# Triarylborane‐Catalyzed Alkenylation Reactions of Aryl Esters with Diazo Compounds

**DOI:** 10.1002/anie.202007176

**Published:** 2020-07-15

**Authors:** Ayan Dasgupta, Katarína Stefkova, Rasool Babaahmadi, Lukas Gierlichs, Alireza Ariafard, Rebecca L. Melen

**Affiliations:** ^1^ Cardiff Catalysis Institute School of Chemistry Cardiff University Main Building, Park Place Cardiff CF10 3AT, Cymru/Wales UK; ^2^ School of Natural Sciences – Chemistry University of Tasmania Private Bag 75 Hobart, Tasmania 7001 Australia

**Keywords:** alkenylation, diazoesters, metal-free catalysis, tris(pentafluorophenyl)borane

## Abstract

Herein we report a facile, mild reaction protocol to form carbon–carbon bonds in the absence of transition metal catalysts. We demonstrate the metal‐free alkenylation reactions of aryl esters with α‐diazoesters to give highly functionalized enyne products. Catalytic amounts of tris(pentafluorophenyl)borane (10–20 mol %) are employed to afford the C=C coupled products (31 examples) in good to excellent yields (36–87 %). DFT studies were used to elucidate the mechanism for this alkenylation reaction.

## Graphical abstract

**2 Become 1**: Lewis acidic boranes are employed in the metal‐free alkenylation reactions of aryl esters with α‐diazoesters to give highly functionalized alkene, enyne, and diene products in good to excellent yields. DFT studies have elucidated the mechanism for the reaction.

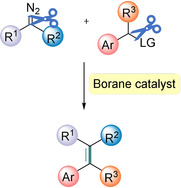

Diazo compounds have found a multitude of applications in synthetic chemistry and are versatile intermediates that can rapidly functionalize organic compounds in a single step.^1^, ^2^ In particular, diazo compounds and *N*‐tosylhydrazones (diazo precursors) have attracted much attention as carbonyl equivalents in the construction of carbon–carbon double bonds.^3^ Transition metal catalysts, such as palladium, are typically used in reactions with diazo compounds to synthesize C=C bonds.^4^ Two mechanistic approaches are generally accepted: 1) migratory insertion of a palladium carbene species (generated from the diazo compound) followed by β‐hydride elimination, or 2) nucleophilic attack of a diazo compound on a transition metal carbene species followed by β‐elimination (Figure 1 a). More recently, more abundant first‐row metals such as copper or iron have been employed with diazo compounds.^5^, ^6^ Of particular relevance is the recent report of FeCl_2_‐catalyzed alkenylation of benzylic C(sp^3^)−H bonds with diazoesters in the presence of an oxidant (Figure 1 b). Nonetheless, metal‐free approaches are comparatively rare. In 2015, the first transition‐metal‐free *gem*‐difluoroolefination of diazo acetates with difluorocarbene reagents was reported (Figure 1 c).^7^ Herein we investigate the alkenylation of benzylic sp^3^ centers using tris(pentafluorophenyl)borane [B(C_6_F_5_)_3_] as the catalyst (Figure 1 d) as a new route to generate conjugated organic compounds.

**Figure 1:**
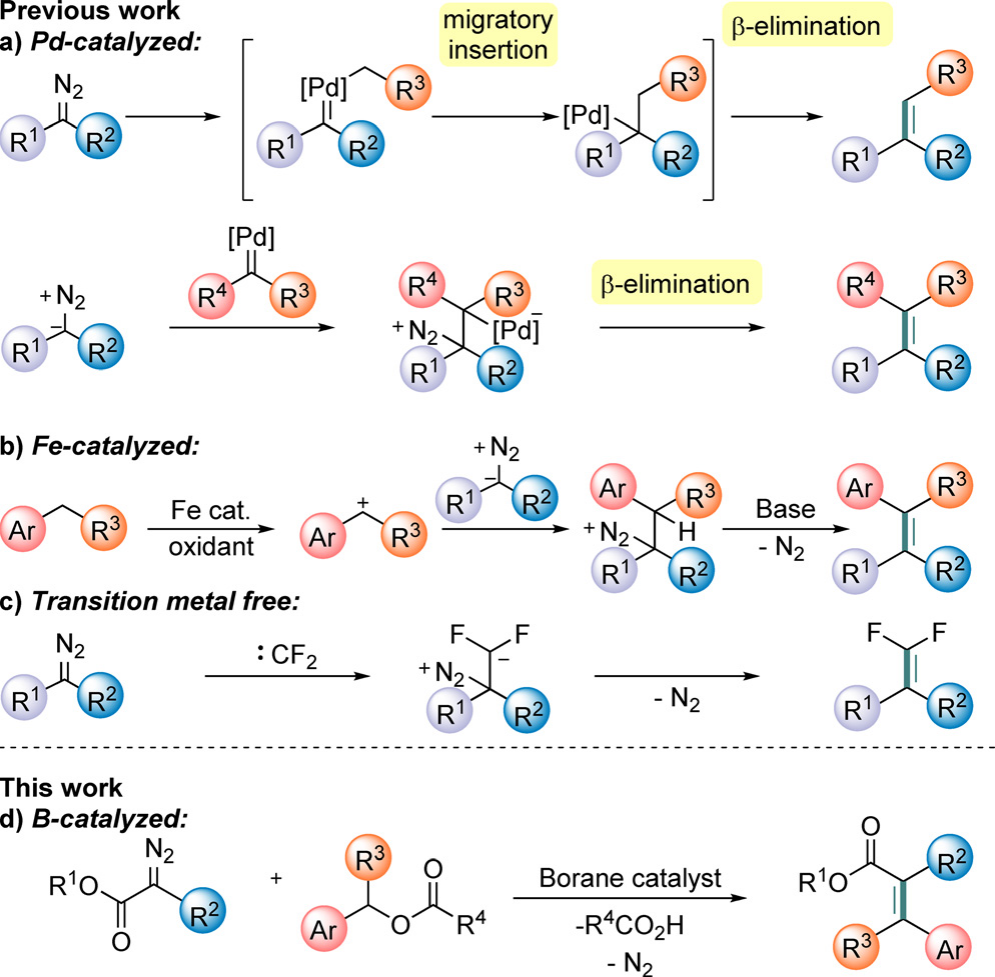
General representation of benzylic alkenylation.

Over the past decade the use of B(C_6_F_5_)_3_ and related triarylboranes have grown in popularity as catalysts for a range of transformations.^8^ Importantly, the ester‐substituted enyne and diene products generated in the reactions reported herein are usually made by metal‐catalyzed Sonogashira cross‐coupling reactions.^9^ These compounds are common starting materials for the synthesis of heterocycles such as pyran‐2‐ones through a simple metal‐ or borane‐catalyzed cyclization reaction.^10^, ^11^ Such pyran‐2‐ones are omnipresent in many bioactive natural products which display inter alia antimicrobial, anti‐HIV, and antitumor activity.^12^

Initially, we reacted dimethyl 2‐diazomalonate (**1 a**) with 1‐(4‐fluorophenyl)‐3‐(trimethylsilyl)prop‐2‐yn‐1‐yl‐4‐fluorobenzoate (**2 a**) in a 1:1.1 ratio in trifluorotoluene (TFT) solvent (Table 1). In the absence of a borane catalyst no reaction occurred after 22 h at 65 °C (Table 1, entry 1). However, the addition of 20 mol % of a fluorinated triarylborane resulted in loss of N_2_ from **1 a** and the benzylic alkenylation of the aryl‐alkynyl ester **2 a** with loss of 4‐fluorobenzoic acid to generate the C=C coupled enyne product dimethyl 2‐(1‐(4‐fluorophenyl)‐3‐(trimethylsilyl)prop‐2‐yn‐1‐ylidene) malonate (**3 a**). A range of borane catalysts were trialed in the reaction, including B(2,4,6‐F_3_C_6_H_2_)_3_, B(3,4,5‐F_3_C_6_H_2_)_3_, and B(C_6_F_5_)_3_, giving the product in 41 %, 25 %, and 78 % isolated yields, respectively when a 20 mol % catalyst loading was used (Table 1, entries 2–4). Other Lewis acids such as BF_3_⋅OEt_2_, on the other hand, showed no conversion after 18 h at 65 °C (Table 1, entry 5). Likewise, the Brønsted acid PTSA (*p*‐toluenesulfonic acid) also gave no desired product (Table 1, entry 6). A reduction in catalyst loading to 10 mol % still gave the product in high yield (81 % after 22 h), whereas reducing the catalytic loading further to 5 mol % resulted in just 42 % yield after 22 h (Table 1, entries 7 and 8).

**Table 1:** Optimization of conditions for the alkenylation reactions of α‐diazoester **1 a** with aryl ester **2 a**. 
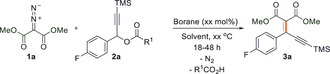

Entry	BAr_3_	Cat. Loading [mol %]	Solv.	*T* [°C]	*t* [h]	Yield [%]^[a]^
1	no cat.	–	TFT	65	22	–
2	B(C_6_F_5_)_3_	20	TFT	65	18	78
3	B(2,4,6‐F_3_C_6_H_2_)_3_	20	TFT	65	18	41
4	B(3,4,5‐F_3_C_6_H_2_)_3_	20	TFT	65	18	25
5	BF_3_⋅OEt	20	TFT	65	18	–
6	PTSA	10	TFT	65	22	–
7	B(C_6_F_5_)_3_	10	TFT	65	22	81
8	B(C_6_F_5_)_3_	5	TFT	65	22	42
9	B(C_6_F_5_)_3_	10	TFT	100	22	48
10	B(C_6_F_5_)_3_	5	TFT	100	22	30
11	B(C_6_F_5_)_3_	20	TFT	RT	48	30
12	B(C_6_F_5_)_3_	10	toluene	65	22	68
13	B(C_6_F_5_)_3_	10	CH_2_Cl_2_	65	22	52
14	B(C_6_F_5_)_3_	10	hexane	65	22	45
15	B(C_6_F_5_)_3_	10	THF	65	22	–

[a] Isolated yield.

Elevating the temperature to 100 °C and using 10 and 5 mol % B(C_6_F_5_)_3_ resulted in side‐products and lowered isolated yields of the product **3 a**, while reducing the temperature to room temperature also gave lower yields when 20 mol % B(C_6_F_5_)_3_ was used (Table 1, entries 9–11). TFT was found to be the best solvent for the reaction, whereas toluene, dichloromethane, and hexane led to reduced yield of the product, and THF resulted in no desired product being formed (Table 1, entries 12–15). Finally, we investigated the effect of different leaving groups on the ester (R^1^, Table 1 scheme) including 4‐FC_6_H_4_, CF_3_, Me, Ph, and ^*t*^Bu. The reaction proceeded in all cases except when R=^*t*^Bu. However, the rate of reaction was faster with electron‐withdrawing groups; therefore we opted to use esters **2** where R^1^=4‐FC_6_H_4_ or CF_3_.

Using the optimized conditions (10 mol % B(C_6_F_5_)_3_, 65 °C, 18–22 h, solvent: TFT) the scope of the reaction was investigated. Reactions of a variety of 2‐diazomalonates (**1 a**–**c**) with aryl‐alkynyl esters (**2 a**–**g**) provided the products **3 a**–**n** in good to very good isolated yields (63–87 %) (Scheme 1). The reaction worked well with several symmetrical diazoesters **1** as well as *p*‐F, *p*‐Cl, *p*‐Br and *p*‐CF_3_ substituents on the aryl group of the aryl‐alkynyl ester **2**. In addition, trimethylsilyl (TMS) and phenyl substituents could be tolerated on the alkyne functionality of **2**. Electron‐releasing (*p*‐OMe) or strongly electron‐withdrawing (2,6‐F_2_) moieties on the aryl group of **2 h** and **2 i** were unsuccessful giving a complex mixture of inseparable products. Likewise, the reaction with di‐*tert*‐butyl 2‐diazomalonate (**1 d**) was unsuccessful showing only decomposition of the diazo compound. Single crystals of **3 a** and **3 n** suitable for X‐ray diffraction could be grown by slow evaporation of a saturated CH_2_Cl_2_ solution (Figure 2).

**Figure 2:**
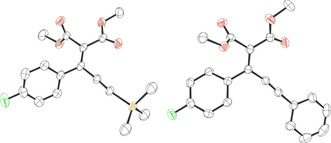
Solid‐state structures of compound **3 a** (left) and **3 n** (right). Thermal ellipsoids drawn at 50 % probability. Carbon: black; oxygen: red; fluorine: green; silicon: yellow.

**Scheme 1:**
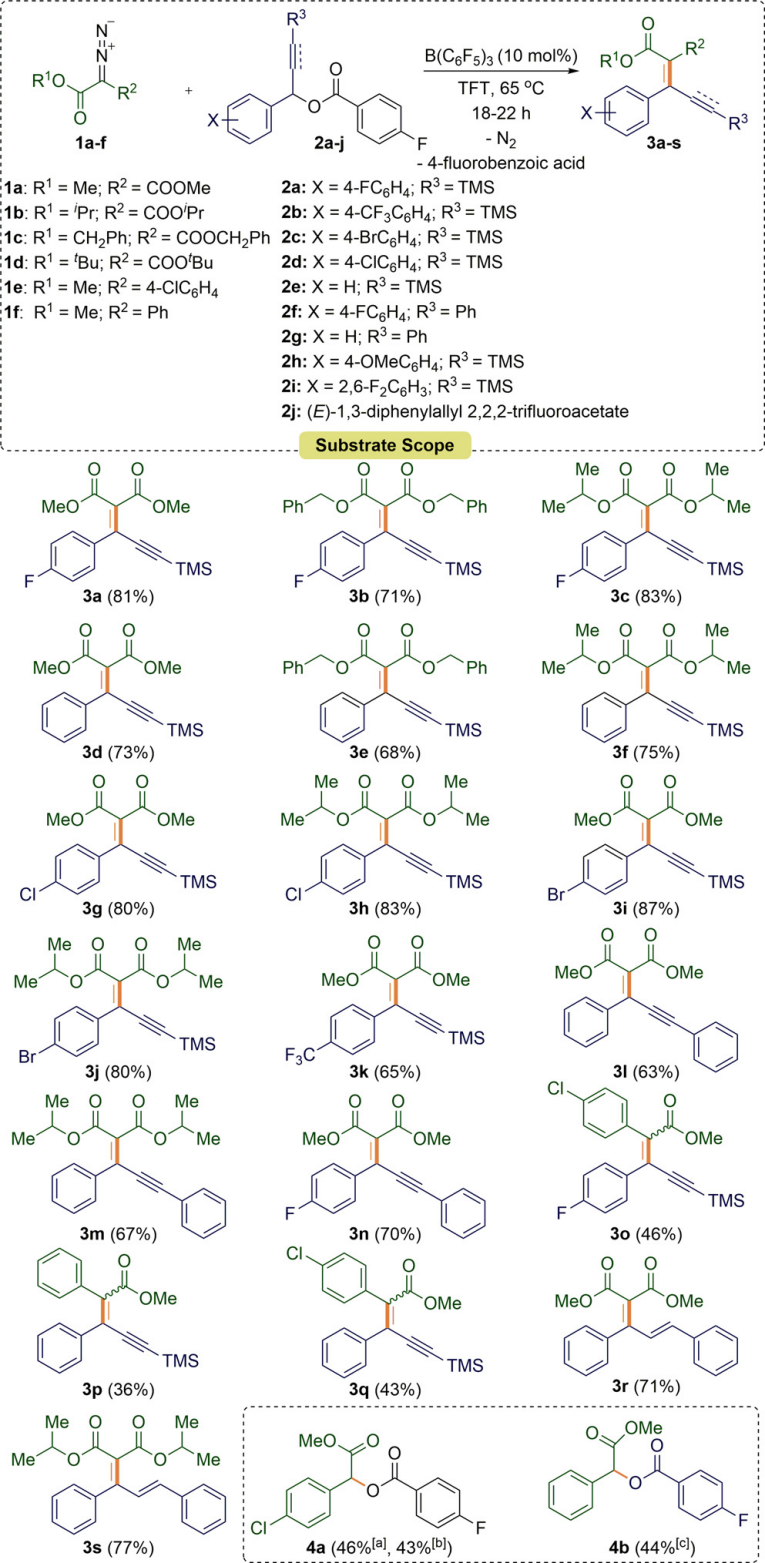
Propargylic alkenylation of aryl‐alkynyl and aryl‐alkenyl esters using diazomalonates. Insert shows the structures of by‐products **4 a** and **4 b**. [a] Yield of by‐product formed in the synthesis of **3 o**. [b] Yield of by‐product formed in the synthesis of **3 q**. [c] Yield of by‐product formed in the synthesis of **3 p**.

We further examined the scope of this methodology in reactions with the unsymmetrical α‐aryl‐diazoesters methyl 2‐(4‐chlorophenyl)‐2‐diazoacetate (**1 e**) and methyl 2‐diazo‐2‐phenylacetate (**1 f**) with **2 a** and **2 e**. The products **3 o–q** were all isolated in yields (36–46 %) that were lower than those obtained for the symmetrical diazoesters. The products were all formed in a 1:0.4 ratio (*E*:*Z*) of diastereoisomers as determined by ^1^H NMR spectroscopy of the crude reaction mixture. One reason for the reduced yield of the C=C coupled product was the observation that new by‐products **4 a**–**b** were formed in an approximate 1:1 ratio with the desired product **3**. We attribute the generation of the by‐products to the increased reactivity of the α‐aryl diazoesters **1 e** and **1 f** which rapidly react with the 4‐fluorobenzoic acid (**5**) generated in the reaction. It should be noted that when diphenyldiazomethane was used, only homocoupling of the diazocompound was observed. Reactions with the aryl‐alkenyl ester **2 j** also proved possible, generating the diene products **3 r** and **3 s** in 71 % and 77 % yields, respectively.

The satisfactory outcome of these reactions led us to extend our work to demonstrate a broader substrate scope for this protocol. Rather than alkynyl or alkenyl esters, diaryl esters were examined for the alkenylation reactions. Under the optimized reaction conditions, diaryl esters **2 k**–**o** reacted with diazoesters **1 a**–**c** to afford the C=C bonded products **6 a**–**l** in good to excellent yields of 67–87 % (Scheme 2). These reactions proved successful with symmetrical diaryl esters containing both electron‐withdrawing (*p*‐F) and electron‐donating (*p*‐OMe) functionalities on the aryl ring. In addition, these reactions were also possible using asymmetrical diaryl esters **2 n** and **2 o**.

**Scheme 2:**
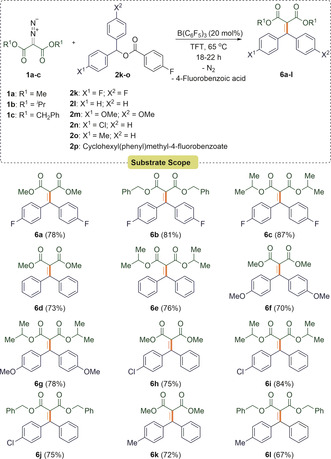
Benzylic alkenylation of diaryl esters using diazomalonates.

Limitations included the reaction of esters bearing a cyclohexyl group such as cyclohexyl(phenyl)methyl 4‐fluorobenzoate (**2 p**). In this case no reaction had occurred even after 32 h. Compound **6 e** was crystallized by vapor diffusion using CH_2_Cl_2_/hexane as solvents and the crystals were measured by X‐ray diffraction (Figure 3).

**Figure 3:**
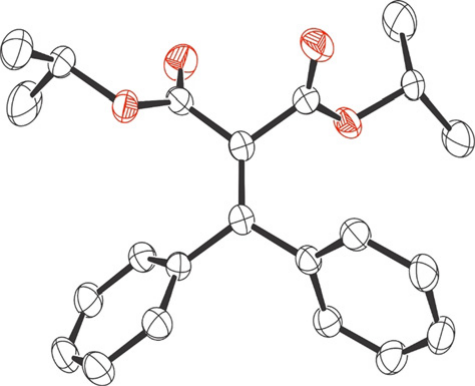
Solid‐state structure of compound **6 e**. Thermal ellipsoids drawn at 50 % probability. Carbon: black; oxygen: red.

Based upon literature precedent, we believe there are two possibilities for substrate activation by the borane catalyst either 1) activation of the carbonyl group on the ester **2** resulting in the generation of a carbenium ion^10^ or 2) activation of the diazo compounds **1** by the borane.^13^, ^14^ In order to determine which pathway is operating, and to investigate the entire reaction mechanism, we undertook DFT calculations at the SMD/M06‐2X‐D3/def2‐TZVP//CPCM/B3LYP/6‐31G(d) level of theory using toluene as the solvent. Figure 4 shows the catalytic cycle proposed by the DFT calculations and Figure 5 shows the free energy profile achieved based on the cycle. It was found that coordination of the borane to the ester **2** is the initial step of the reaction to give the adduct **I1** via a low‐energy transition state (**TS_1_**) with a relative free energy of 3.6 kcal mol^−1^. This was calculated to be a lower energy pathway by 13.0 kcal mol^−1^ than coordination of the borane to the diazo compound, a transformation that was computed to be endergonic by about 13.6 kcal mol^−1^ (Figure S144). **I1** was found to have an elongated C−O bond length of 1.502 Å, which results in bond cleavage and the generation of an electrophilic carbenium ion in salt **I2**, occurring with an activation barrier of 13.4 kcal mol^−1^ through **TS_2_**. The generation of the carbenium ion corroborates the observation that esters less able to stabilize a positive charge such as **2 p** were unsuccessful in these reactions. **I2** exists as a close ion pair and reacts with the diazo substrate **1** as a nucleophile via **TS_3_** (activation barrier 13.8 kcal mol^−1^) to give the salt **I3**. The reaction between **I2** and **1** results in the C−N π‐bond in the diazo substrate being interrupted, as evidenced by the elongated C−N bond in **I3**. The resultant intermediate (**I3**) also exists as a close ion pair with a short O–H contact (1.945 Å). Finally, an E2‐type elimination reaction through **TS4** with an activation barrier as low as 3.7 kcal mol^−1^ releases dinitrogen and 4‐fluorobenzoic acid (**5**) as a borane adduct to generate the C=C double bonded product **3**.

**Figure 4:**
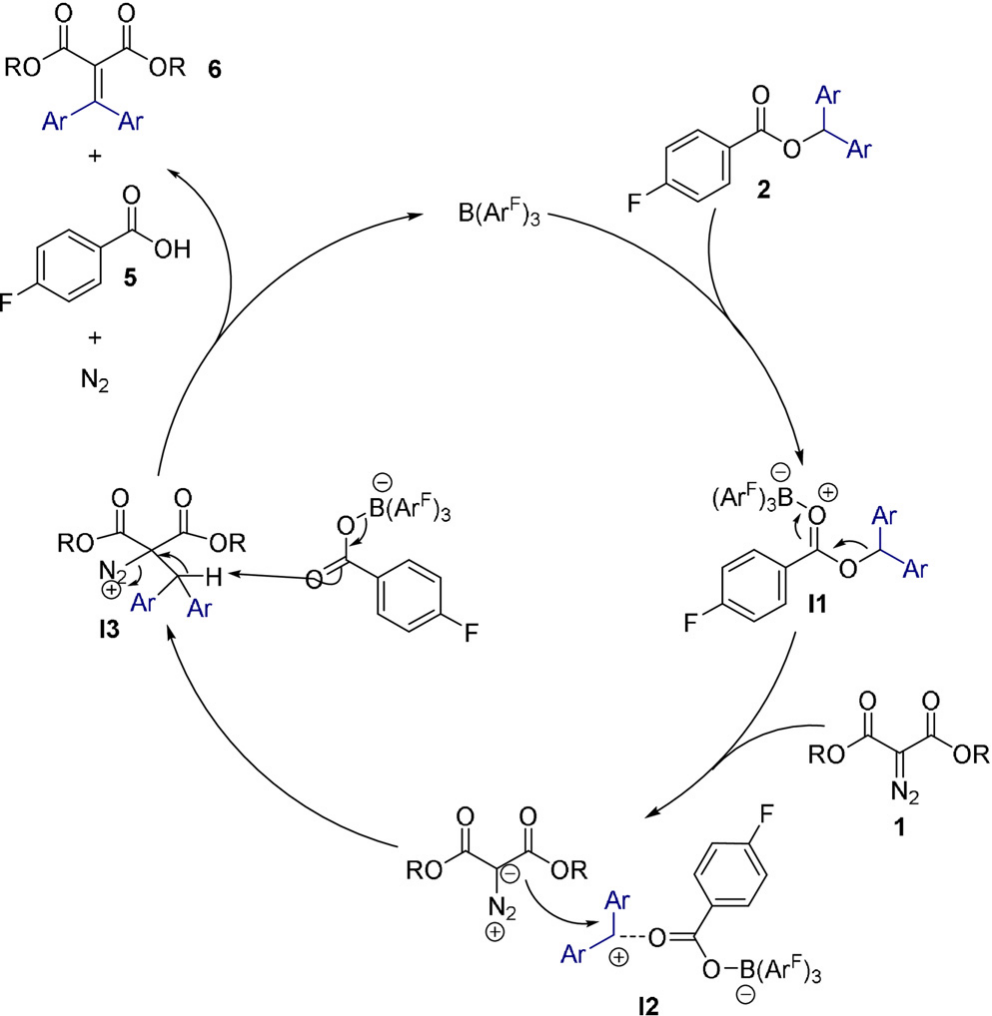
Catalytic cycle proposed by DFT calculations.

**Figure 5:**
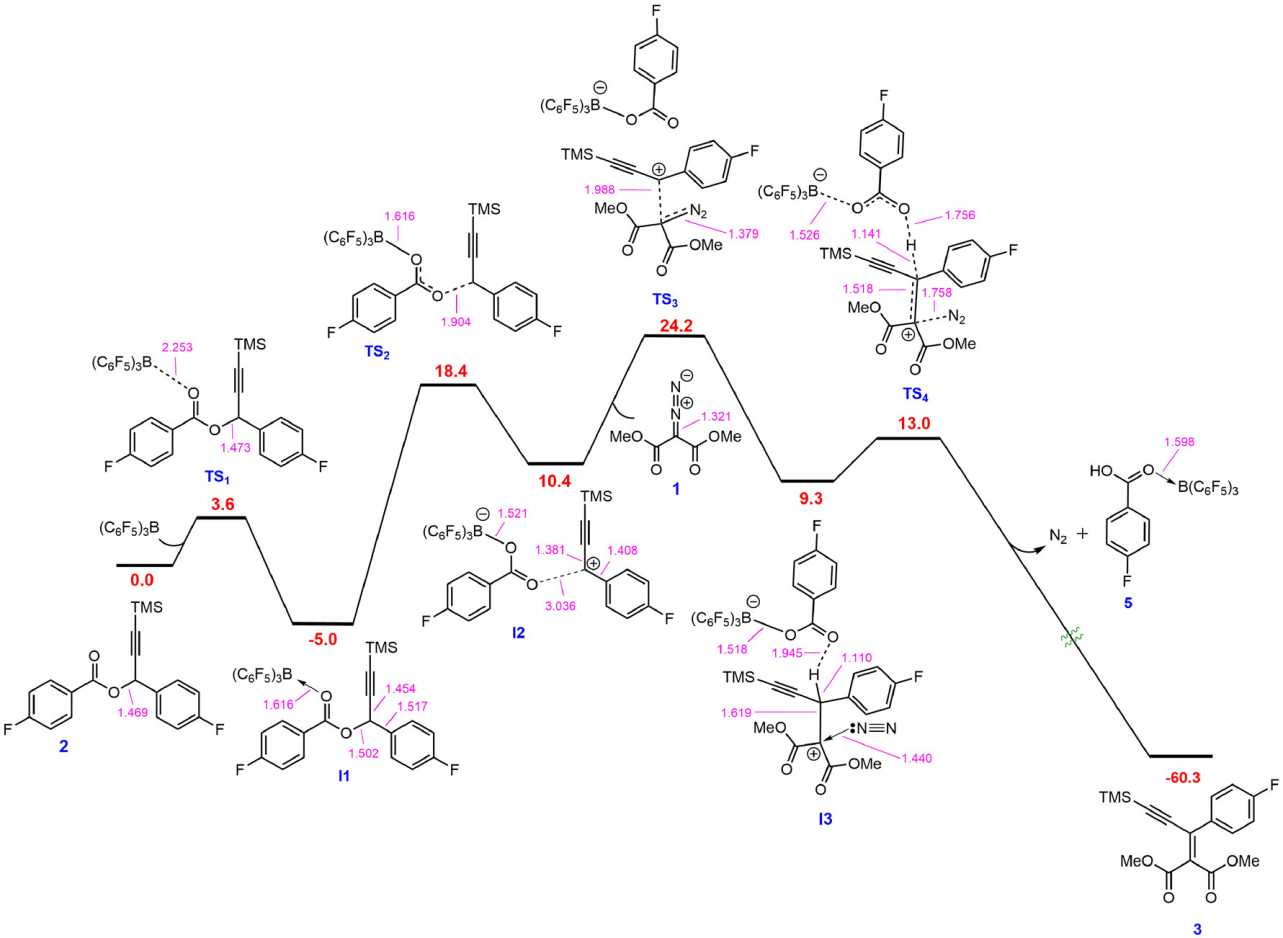
DFT‐calculated free energy profile for the reaction. The relative free energies are given in kcal mol^−1^ and the selected bond distances (in pink) in Å.

In conclusion, we have demonstrated a simple, mild reaction protocol for propargylic, allylic, and benzylic alkenylation to form highly functionalized C=C bonded products in the absence of a metal catalyst. B(C_6_F_5_)_3_ was found to be the most effective catalyst for the reaction, giving the C−C bonded product in good to excellent yields. Comprehensive DFT studies were used to elucidate the reaction mechanism which highlight that the key role of the catalyst is to activate the aryl ester rather than the diazomalonate. Importantly, this simple reaction allows complex ester‐substituted enyne and diene products to be generated in a single step. Notably, the products generated are essential precursors to prepare biologically active heterocyclic molecules.

## Conflict of interest

The authors declare no conflict of interest.

## Supporting information

As a service to our authors and readers, this journal provides supporting information supplied by the authors. Such materials are peer reviewed and may be re‐organized for online delivery, but are not copy‐edited or typeset. Technical support issues arising from supporting information (other than missing files) should be addressed to the authors.

Supplementary
