# Supplementary material for: Triarylborane‐Catalyzed Alkenylation Reactions of Aryl Esters with Diazo Compounds
Source: Angew Chem Int Ed Engl. 2020 Jul 15;59(36):15492–6. doi: 10.1002/anie.202007176 (PMC7497215; doi:10.1002/anie.202007176)
Supplement: Supplementary file 1 — Supplementary [file ANIE-59-15492-s001.pdf]

Angewandte Chemie
Eine Zeitschrift der Gesellschaft Deutscher Chemiker

Supporting Information

Triarylborane-Catalyzed Alkenylation Reactions of Aryl Esters with Diazo Compounds

Ayan Dasgupta[+], Katarína Stefkova[+], Rasool Babaahmadi, Lukas Gierlichs, Alireza Ariafard, and Rebecca L. Melen**

anie_202007176_sm_miscellaneous_information.pdf

Table of Contents

1	Experimental		02
	1.1	General experimental	02
	1.2	General procedure a: synthesis of diazoesters	03
	1.2.1	Synthesis and spectral characterization of diazo compounds	04
	1.3	General procedure b: synthesis of alkyne esters	06
	1.3.1	Synthesis and spectral characterization of alkyne-ester compounds	06
	1.4	General procedure c: synthesis of diaryl esters	12
	1.4.1	Synthesis and spectral characterization of diaryl ester compounds	13
2	Product Characterization		16
	2.1	General procedure d: C–C coupling reaction	16
	2.2	Synthesis and spectral characterization of products	16
3	NMR Spectra		33
4	Crystallographic Data		172
	4.1	Single crystal X-ray diffraction	172
	4.2	Solid-state structures	173
	4.3	X-ray refinement data	176
5	Computational Data		179
	5.1	Additional free energy profiles	179
	5.2	Cartesian coordinates and total energies for the calculated structures	183
	5.3	Computational details	207
6	References		208

1. Experimental

1.1 General experimental

With the exception of the starting materials, all reactions and manipulations were carried out under an atmosphere of dry, O_2-free nitrogen using standard double-manifold techniques with a rotary oil pump. A nitrogen-filled glove box (MBraun) was used to manipulate solids including the storage of starting materials, room temperature reactions, product recovery and sample preparation for analysis. All solvents (toluene, hexane, tetrahydrofuran, chloroform, acetonitrile,

dichloromethane, pentane) were dried by employing a Grubbs-type column system (Innovative Technology) or a solvent purification system MB SPS-800 and stored under a nitrogen atmosphere. Anhydrous (with Sure/Seal) α,α,α-trifluorotoluene (TFT) was purchased from Merck and dried over molecular sieves before use. Deuterated solvents were distilled and/or dried over molecular sieves before use. Chemicals were purchased from commercial suppliers and used as received. All the triarylboranes were prepared as per the standard literature report.[1][2] Thin-layer chromatography (TLC) was performed on pre-coated aluminum sheets of Merck silica gel 60 F254 (0.20 mm) and visualized by UV radiation (254 nm) also a solution of $KMnO_4$ (1.5 g $KMnO_4$, 10 g K_2CO_3, and 1.25 mL 10% NaOH in 200 mL water) were used to develop the stain on TLC plates. ^{1}H, ^{13}C, and ^{19}F NMR spectra were recorded on a Bruker Avance II 400 or Bruker Avance 500 spectrometers. All coupling constants are absolute values and are expressed in Hertz (Hz). ^{13}C NMR was measured as ^{1}H decoupled. Yields are given as isolated yields. Chemical shifts are expressed as parts per million (ppm, δ) downfield of tetramethylsilane (TMS) and are referenced to $CDCl_3$ (7.26/77.16 ppm) as internal standard. NMR spectra were referenced to $CFCl_3$ (^{19}F).[3] The description of signals includes s = singlet, d = doublet, t = triplet, q = quartet, and m = multiplet, br. = broad. All coupling constants are absolute values and are expressed in Hertz (Hz). ^{13}C NMR was measured as ^{1}H decoupled. Yields are given as isolated yields. All spectra were analyzed assuming a first order approximation. IR-Spectra were measured on a Shimadzu IRAffinity-1 photo-spectrometer. Mass spectra were measured on a Waters LCT Premier/XE or a Waters GCT Premier spectrometer. Ions were generated by the Atmospheric Solids, Analysis Probe (ASAP), Electrospray (ES) or Electron Ionisation (EI). The molecular ion peaks values quoted for either molecular ion (M^+), molecular ion plus or minus hydrogen ($M+H^+$, $M-H^–$), molecular ion minus hydride ($M-H^+$), molecular ion plus sodium ($M+Na^+$).

1.2 Synthesis of diazoesters

General Procedure a: 1,8-Diazabicyclo[5.4.0]undec-7-ene (1.5 equiv.) (until otherwise mentioned) was added to a solution of corresponding ester (1 equiv.) and 4-acetamidobenzenesulfonyl azide (1.2 equiv.) in anhydrous CH_3CN (30 mL). The reaction mixture was stirring at room temperature for 14–16 h under the nitrogen atmosphere. The organic compounds were extracted with diethyl ether (3 × 25 mL), the combined organic fractions were washed with brine solution (1 × 30 mL), dried over $MgSO_4$ and concentrated using vacuum. The

crude compound was purified *via* column chromatography using silica gel (Merck, 60 Å, 230–400 mesh particle size) and hexane/ethyl acetate as eluent.

1.2.1 Synthesis and spectral characterization of diazo compounds

*Synthesis of dimethyl 2-diazomalonate (**1a**):*[4]

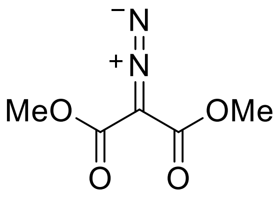

Synthesized in accordance with *General Procedure a* using 1,8-diazabicyclo[5.4.0]undec-7-ene (6.3 mL, 42.0 mmol), 4-acetamidobenzenesulfonyl azide (8.65 g, 36.0 mmol), and dimethyl malonate (3.4 mL, 30.0 mmol). The crude compound was purified *via* column chromatography using hexane/ethyl acetate (80:20 v/v) as eluent. The desired product (**1a**) was obtained as a yellow liquid. Yield: 4.50 g, 95%, 28.5 mmol.

^{1}H NMR (400 MHz, $CDCl_3$, 298 K) δ: 3.81 (s, 6H, OCH_3); ^{13}C NMR (101 MHz, $CDCl_3$, 298 K) δ: 161.5 (C=O), 52.6 (CH_3).

*Synthesis of diisopropyl 2-diazomalonate (**1b**):*

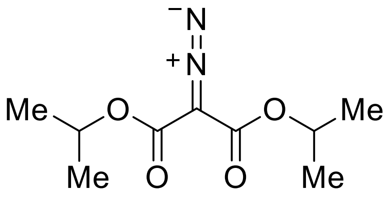

Synthesized in accordance with *General Procedure a* using triethylamine (4.40 mL, 31.9 mmol, 2 equiv.), 4-acetamidobenzenesulfonyl azide (4.60 g, 19.1 mmol), and diisopropyl malonate (3.00 mL, 15.9 mmol). The crude compound was purified *via* column chromatography using hexane/ethyl acetate (80:20 v/v) as eluent. The desired product (**1b**) was obtained as a yellow liquid. Yield: 2.96 g, 87%, 13.8 mmol.

^{1}H NMR (400 MHz, $CDCl_3$, 298 K) δ: 5.15 (hept, J = 6.2 Hz, 2H, CH), 1.29 (d, J = 6.3 Hz, 12H, CH_3); ^{13}C NMR (101 MHz, $CDCl_3$, 298 K) δ: 160.8 (C=O), 69.5 (CH), 22.0 (CH_3); HRMS (ES+) $[M+Na]^+$ $[C_9H_{14}N_2O_4Na]^+$: calculated 237.0851, found 237.0854.

*Synthesis of dibenzyl 2-diazomalonate (**1c**):*[5]

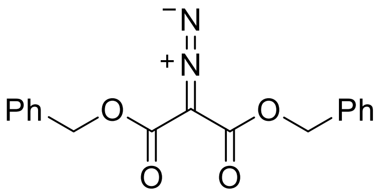

Synthesized in accordance with *General Procedure a* using triethylamine (2.90 mL, 21.1 mmol, 2 equiv.), 4-acetamidobenzenesulfonyl azide (3.04 g, 12.6 mmol), and dibenzyl malonate (2.60 mL, 10.5 mmol). The crude compound

was purified *via* column chromatography using hexane/ethyl acetate (80:20 v/v) as eluent. The desired product (**1c**) was obtained as a white solid. Yield: 2.8 g, 85%, 9.02 mmol.

^{1}H NMR (400 MHz, $CDCl_3$, 298 K) δ: 7.42–7.31 (m, 10H, Ar–H), 5.28 (s, 4H, CH_2); ^{13}C NMR (101 MHz, $CDCl_3$, 298 K) δ: 161.0 (C=O), 135.4, 128.8, 128.6, 128.4, 67.2 (CH_2).

*Synthesis of di-tert-butyl 2-diazomalonate (**1d**):*[6]

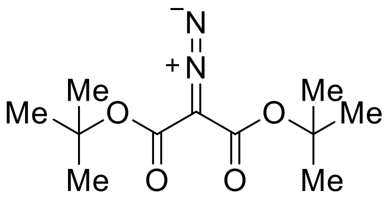

Synthesized in accordance with *General Procedure a* using triethylamine (2.90 mL, 21.1 mmol, 2 equiv.), 4-acetamidobenzenesulfonyl azide (3.04 g, 12.6 mmol), and di-*tert*-butyl malonate (2.3 mL, 10.5 mmol). The crude compound was purified *via* column chromatography using hexane/ethyl acetate (80:20 v/v) as eluent. The desired product (**1d**) was obtained as a colorless oil. Yield: 2.1 g, 8.67 mmol, 83%.

^{1}H NMR (500 MHz, $CDCl_3$, 298 K) δ: 1.48 (s, 18H, CH_3); ^{13}C NMR (126 MHz, $CDCl_3$, 298 K) δ: 160.4 (C=O), 82.8, (CH), 28.3 (CH_3).

*Synthesis of methyl 2-(4-chlorophenyl)-2-diazoacetate (**1e**):*[7]

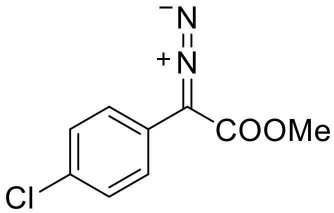

Synthesized in accordance with *General Procedure a* using 1,8-diazabicyclo[5.4.0]undec-7-ene (2.4 mL, 16.2 mmol), 4-acetamidobenzenesulfonyl azide (3.12 g, 13.0 mmol), and methyl 2-(4-chlorophenyl)acetate (2 g, 10.8 mmol). The crude compound was purified *via* column chromatography using hexane/ethyl acetate (20:1 v/v) as eluent. The desired product (**1e**) was obtained as an orange solid. Yield: 2.12 g, 93%, 10.1 mmol.

^{1}H NMR (400 MHz, $CDCl_3$, 298 K) δ: 7.43–7.40 (m, 2H, Ar–H), 7.36–7.33 (m, 2H, Ar–H), 3.87 (s, 3H, $COOCH_3$); ^{13}C NMR (101 MHz, $CDCl_3$, 298 K) δ: 165.4 (C=O), 131.6, 129.2, 125.1, 124.2, 52.2 (CH_3).

*Synthesis of methyl 2-diazo-2-phenylacetate (**1f**):*[7]

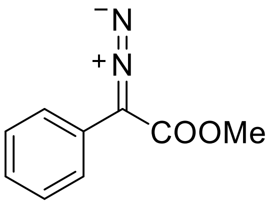

Synthesized in accordance with *General Procedure a* using 1,8-diazabicyclo[5.4.0]undec-7-ene (2.9 mL, 20.0 mmol), 4-acetamidobenzenesulfonyl azide (3.84 g, 16.0 mmol), and methyl 2-phenylacetate (2 g, 13.3 mmol). The crude compound was purified *via*

column chromatography using hexane/ethyl acetate (20:1 v/v) as eluent. The desired product (**1f**) was obtained as a yellow solid. Yield: 1.92 g. 82%, 10.9 mmol.

^{1}H NMR (400 MHz, $CDCl_3$, 298 K) δ: 7.49–7.47 (m, 2H, Ar–H), 7.40–7.37 (m, 1H, Ar–H), 7.21–7.17 (m, 2H, Ar–H), 3.87 (s, 3H, $COOCH_3$); ^{13}C NMR (101 MHz, $CDCl_3$, 298 K) δ: 165.7 (C=O), 129.0, 125.9, 125.6, 124.1, 52.1 (CH_3).

1.3 Synthesis of alkyne-esters

General Procedure b: Corresponding alkyne (1.2 equiv.) was dissolved in dry tetrahydrofuran (25 mL) and the reaction mixture was cooled to 0 °C. *n*-BuLi (2.5 M in hexanes, 1.2 equiv.) was added dropwise to the reaction mixture at 0 °C. The reaction mixture was stirred for 1 h at ambient temperature. The mixture was cooled down to 0 °C and the aldehyde (1 equiv.) was added dropwise, allowed the reaction mixture to warm to room temperature and stirred for additional 2 h at ambient temperature. 4-Fluorobenzoyl chloride (1.2 equiv.) was added to the reaction mixture dropwise at 0 °C. The reaction was stirred at ambient temperature for 15 min. Saturated aqueous NH_4Cl solution was used to quench the reaction. The organic layer was extracted with ethyl acetate (3 × 25 mL). The combined organic fractions were washed with brine solution and dried over $MgSO_4$ and concentrated using vacuum. The crude compound was purified *via* column chromatography using silica gel (Merck, 60 Å, 230–400 mesh particle size) and hexane/ethyl acetate as eluent.

1.3.1 Synthesis and spectral characterization of alkyne-ester compounds

*Synthesis of 1-(4-fluorophenyl)-3-(trimethylsilyl)prop-2-yn-1-yl 4-fluorobenzoate (**2a**):*

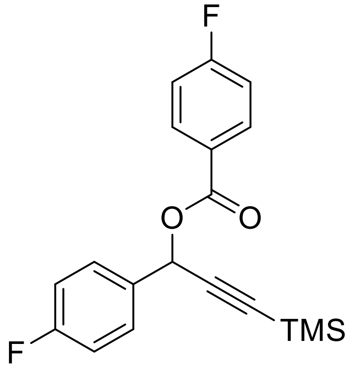

Synthesized in accordance with *General Procedure b* using trimethylsilylacetylene (2.80 mL, 19.3 mmol), *n*-BuLi (7.70 mL, 19.3 mmol), 4-fluorobenzaldehyde (2.00 g, 16.1 mmol), and 4-fluorobenzoyl chloride (2.30 mL, 19.3 mmol). The crude compound was purified *via* column chromatography using hexane/ethyl acetate (95:5 v/v) as eluent. The desired product (**2a**) was obtained as a yellow oil. Yield: 4.33 g, 78%, 12.6 mmol.

^{1}H NMR (400 MHz, $CDCl_3$, 298 K) δ: 8.10–8.07 (m, 2H, Ar–H), 7.61–7.58 (m, 2H, Ar–H), 7.13–7.06 (m, 4H, Ar–H), 6.70 (s, 1H, CH), 0.22 (s, 9H, $Si(CH_3)_3$); ^{13}C NMR (101 MHz, $CDCl_3$, 298

K) δ: 166.1 (d, $J_{C–F}$ = 254.5 Hz), 164.4 (C=O), 163.1 (d, $J_{C–F}$ = 248.2 Hz), 133.0 (d, $J_{C–F}$ = 3.2 Hz), 132.6 (d, $J_{C–F}$ = 9.4 Hz), 129.9 (d, $J_{C–F}$ = 8.5 Hz), 126.0 (d, $J_{C–F}$ = 3.1 Hz), 115.7 (dd, $J_{C–F}$ = 21.9, 3.7 Hz), 101.0 (C≡C), 93.2 (C≡C), 65.9 (CH), -0.1 ($Si(CH_3)_3$); ^{19}F NMR (376 MHz, $CDCl_3$, 298 K) δ: -104.93 (Ar–F), -112.45 (Ar–F); IR ν_{max} (cm^{-1}): 3076, 2964, 2179 (C≡C), 1724 (C=O), 1602, 1506, 1413, 1367, 1251, 1230, 1101, 1083; HRMS (EI+) $[M]^+$ $[C_{19}H_{18}F_2O_2Si]^+$: calculated 344.1044, found 344.1039.

*Synthesis of 1-(4-(trifluoromethyl)phenyl)-3-(trimethylsilyl)prop-2-yn-1-yl 4-fluorobenzoate (**2b**):*

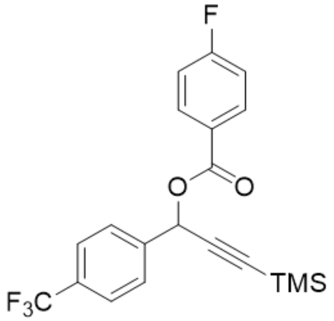

Synthesized in accordance with *General Procedure b* using trimethylsilylacetylene (2 mL, 13.8 mmol), *n*-BuLi (5.5 mL, 13.8 mmol), 4-(trifluoromethyl)benzaldehyde (2 g, 11.5 mmol), and 4-fluorobenzoyl chloride (1.6 mL, 13.8 mmol). The crude compound was purified *via* column chromatography using hexane/ethyl acetate (95:5 v/v) as eluent. The desired product (**2b**) was obtained as off–white solid. Yield: 3.40 g, 75%, 8.61 mmol.

^{1}H NMR (400 MHz, $CDCl_3$, 298 K) δ: 8.11–8.08 (m, 2H, Ar–H), 7.73–7.65 (m, 4H, Ar–H), 7.14–7.10 (m, 2H, Ar–H), 6.75 (s, 1H, CH), 0.21 (s, 9H, $Si(CH_3)_3$); ^{13}C NMR (101 MHz, $CDCl_3$, 298 K) δ: 166.2 (d, $J_{C–F}$ = 254.9 Hz), 164.3 (C=O), 140.9, 132.7 (d, $J_{C–F}$ = 9.4 Hz), 131.2 (q, $J_{C–F}$ = 32.5 Hz), 128.2, 125.9 (d, $J_{C–F}$ = 3.8 Hz), 125.88, 125.84, 122.6, 115.8 (d, $J_{C–F}$ = 22.1 Hz), 100.3 (C≡C), 93.9 (C≡C), 65.9 (CH), -0.1 ($Si(CH_3)_3$); ^{19}F NMR (376 MHz, $CDCl_3$, 298 K) δ: -62.71 (3F, Ar–CF_3), -104.59 (1F, Ar–F); IR ν_{max} (cm^{-1}): 3026, 2986, 2123 (C≡C), 1724 (C=O), 1604, 1506, 1413, 1163, 1045; HRMS (EI+) $[M]^+$ $[C_{20}H_{18}F_4O_2Si]^+$: calculated 394.1012, found 394.1008.

*Synthesis of 1-(4-bromophenyl)-3-(trimethylsilyl)prop-2-yn-1-yl 4-fluorobenzoate (**2c**):*

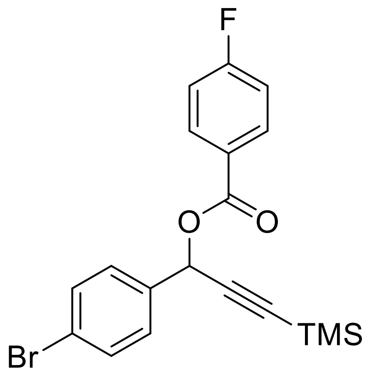

Synthesized in accordance with *General Procedure b* using trimethylsilylacetylene (1.9 mL, 13.0 mmol), *n*-BuLi (5.2 mL, 12.97 mmol), 4-bromobenzaldehyde (2 g, 10.8 mmol), and 4-fluorobenzoyl chloride (1.5 mL, 13.0 mmol). The crude compound was purified *via* column chromatography using hexane/ethyl acetate (95:5 v/v) as eluent. The desired product (**2c**) was obtained as a yellow liquid. Yield: 3.07 g, 70 %, 7.57 mmol.

^{1}H NMR (400 MHz, $CDCl_3$, 298 K) δ: 8.10–8.06 (m, 2H, Ar–H), 7.54–7.47 (m, 4H, Ar–H), 7.13–7.08 (m, 2H, Ar–H), 6.67 (s, 1H, CH), 0.21 (s, 9H, $Si(CH_3)_3$); ^{13}C NMR (101 MHz, $CDCl_3$, 298 K) δ: 166.1 (d, $J_{C–F}$ = 254.7 Hz), 164.4 (C=O), 136.1, 132.6 (d, $J_{C–F}$ = 9.4 Hz), 131.9, 129.6, 125.9 (d, $J_{C–F}$ = 3.0 Hz), 123.3, 115.7 (d, $J_{C–F}$ = 22.0 Hz), 100.7 (C≡C), 93.4 (C≡C), 65.9 (CH), -0.1 ($Si(CH_3)_3$); ^{19}F NMR (376 MHz, $CDCl_3$, 298 K) δ: -104.77 (Ar–F); IR ν_{max} (cm^{-1}): 3053, 2960, 2141 (C≡C), 1722 (C=O), 1602, 1506, 1487, 1315, 1290, 1153, 1153, 1058; HRMS (EI+) $[M]^+$ $[C_{19}H_{18}BrFO_2Si]^+$: calculated 404.0243, found 404.0238.

*Synthesis of 1-(4-chlorophenyl)-3-(trimethylsilyl)prop-2-yn-1-yl 4-fluorobenzoate (**2d**):*

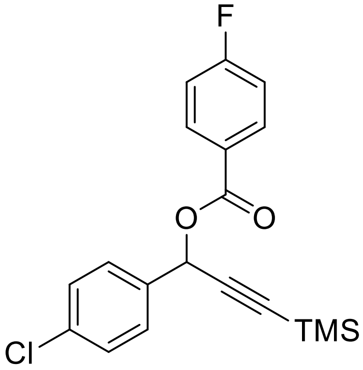

Synthesized in accordance with *General Procedure b* using trimethylsilylacetylene (2.4 mL, 17.1 mmol), *n*-BuLi (6.8 mL, 17.1 mmol), 4-chlorobenzaldehyde (2 g, 14.2 mmol), and 4-fluorobenzoyl chloride (2 mL, 17.1 mmol). The crude compound was purified *via* column chromatography using hexane/ethyl acetate (95:5 v/v) as eluent. The desired product (**2d**) was obtained as a yellow liquid. Yield: 4.01 g, 78%, 11.1 mmol).

^{1}H NMR (500 MHz, $CDCl_3$, 298 K) δ: 8.09–8.07 (m, 2H, Ar–H), 7.55–7.53 (m, 2H, Ar–H), 7.38–7.36 (m, 2H, Ar–H), 7.12–7.09 (m, 2H, Ar–H), 6.68 (s, 1H, CH), 0.21 (s, 9H, $Si(CH_3)_3$); ^{13}C NMR (126 MHz, $CDCl_3$, 298 K) δ: 166.1 (d, $J_{C–F}$ = 254.6 Hz), 164.4 (C=O), 135.6, 135.0, 132.6 (d, $J_{C–F}$ = 9.4 Hz), 129.3, 129.0, 125.9 (d, $J_{C–F}$ = 3.0 Hz), 115.7 (d, $J_{C–F}$ = 22.1 Hz), 100.7 (C≡C), 93.4 (C≡C), 65.9 (CH), -0.1 ($Si(CH_3)_3$); ^{19}F NMR (471 MHz, $CDCl_3$, 298 K) δ: -104.84 (Ar–F); IR ν_{max} (cm^{-1}): 3053, 2960, 2150 (C≡C), 1724 (C=O), 1602, 1506, 1490, 1411, 1315, 1290, 1153, 1043; HRMS (EI+) $[M]^+$ $[C_{19}H_{18}ClFO_2Si]^+$: calculated 360.0749, found 360.0743.

*Synthesis of 1-phenyl-3-(trimethylsilyl)prop-2-yn-1-yl 4-fluorobenzoate (**2e**):*

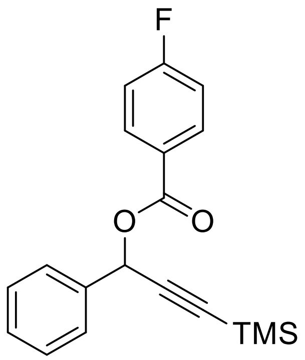

Synthesized in accordance with *General Procedure b* using trimethylsilylacetylene (3.2 mL, 22.6 mmol), *n*-BuLi (9.1 mL, 22.8 mmol), benzaldehyde (2 g, 18.8 mmol), and 4-fluorobenzoyl chloride (2.7 mL, 22.6 mmol). The crude compound was purified *via* column chromatography using hexane/ethyl acetate (95:5 v/v) as eluent. The desired product (**2e**) was obtained as a yellow liquid. Yield: 5.11 g, 83%, 15.6 mmol.

^{1}H NMR (500 MHz, $CDCl_3$, 298 K) δ: 8.11–8.08 (m, 2H, Ar–H), 7.61–7.59 (m, 2H, Ar–H), 7.42–7.37 (m, 3H, Ar–H), 7.12–7.08 (m, 2H, Ar–H), 6.72 (s, 1H, CH), 0.20 (s, 9H, $Si(CH_3)_3$); ^{13}C NMR (126 MHz, $CDCl_3$, 298 K) δ: 166.0 (d, $J_{C–F}$ = 254.3 Hz), 164.5 (C=O), 137.0, 132.6 (d, $J_{C–F}$ = 9.4 Hz), 129.1, 128.8, 127.9, 126.2 (d, $J_{C–F}$ = 3.0 Hz), 115.6 (d, $J_{C–F}$ = 22.0 Hz), 101.2 (C≡C), 93.0 (C≡C), 66.6 (CH), -0.1 (s, 9H, $Si(CH_3)_3$); ^{19}F NMR (471 MHz, $CDCl_3$, 298 K) δ: -105.17 (Ar–F); IR ν_{max} (cm^{-1}): 3062, 2960, 2177 (C≡C), 1720 (C=O), 1602, 1506, 1454, 1411, 1345, 1256, 1125, 1054; HRMS (EI+) $[M]^+$ $[C_{19}H_{19}FO_2Si]^+$: calculated 326.1138, found 326.1133.

*Synthesis of 1-(4-fluorophenyl)-3-phenylprop-2-yn-1-yl 4-fluorobenzoate (**2f**):*

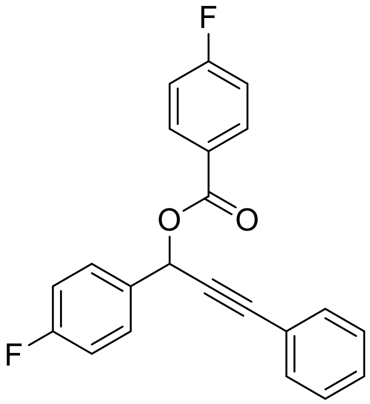

Synthesized in accordance with *General Procedure b* using phenylacetylene (2.1 mL, 19.3 mmol), *n*-BuLi (7.8 mL, 19.3 mmol), 4-fluorobenzaldehyde (2 g, 16.1 mmol), and 4-fluorobenzoyl chloride (2.3 mL, 19.3 mmol). The crude compound was purified *via* column chromatography using hexane/ethyl acetate (95:5 v/v) as eluent. The desired product (**2f**) was obtained as a white solid. Yield: 4.55 g, 81%, 13.1 mmol.

^{1}H NMR (400 MHz, $CDCl_3$, 298 K) δ: 8.13–8.10 (m, 2H, Ar–H), 7.69–7.66 (m, 2H, Ar–H), 7.50 (dt, *J* = 7.7, 1.5 Hz, 2H, Ar–H), 7.38–7.31 (m, 3H, Ar–H), 7.12 (t, *J* = 8.7 Hz, 4H, Ar–H), 6.92 (s, 1H, CH); ^{13}C NMR (101 MHz, $CDCl_3$, 298 K) δ: 166.1 (d, $J_{C–F}$ = 254.6 Hz), 164.6 (C=O), 163.1 (d, $J_{C–F}$ = 248.2 Hz), 133.2 (d, $J_{C–F}$ = 3.2 Hz), 132.6 (d, $J_{C–F}$ = 9.4 Hz), 132.0, 129.9 (d, $J_{C–F}$ = 8.5 Hz), 129.1, 128.4, 126.0 (d, $J_{C–F}$ = 3.0 Hz), 122.0, 87.7 (C≡C), 85.3 (C≡C), 66.2 (CH); ^{19}F NMR (376 MHz, $CDCl_3$, 298 K) δ: -104.85 (Ar–F), -112.34 (Ar–F);

IR ν_{max} (cm^{-1}): 3076, 2964, 2179 (C≡C), 1724 (C=O), 1651, 1523, 1454, 1413, 1367, 1251, 1153, 1083; HRMS (EI+) $[M]^+$ $[C_{22}H_{14}F_2O_2]^+$: calculated 348.0962, found 348.0954.

*Synthesis of 1,3-diphenylprop-2-yn-1-yl 4-fluorobenzoate (**2g**):*

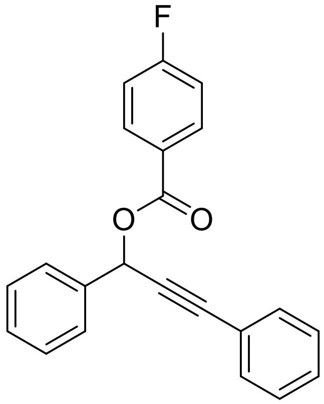

Synthesized in accordance with *General Procedure b* using phenylacetylene (2.5 mL, 22.6 mmol), *n*-BuLi (9.1 mL, 22.6 mmol), benzaldehyde (2 g, 18.8 mmol), and 4-fluorobenzoyl chloride (2.7 mL, 22.6 mmol). The crude compound was purified *via* column chromatography using hexane/ethyl acetate (95:5 v/v) as eluent. The desired product (**2g**) was obtained as a yellow solid. Yield: 4.98 g, 80%, 15.1 mmol.

^{1}H NMR (400 MHz, $CDCl_3$, 298 K) δ: 8.16–8.11 (m, 2H, Ar–H), 7.69 (dt, J = 6.1, 1.7 Hz, 2H, Ar–H), 7.51 (dt, J = 6.1, 2.2 Hz, 2H, Ar–H), 7.47–7.40 (m, 3H, Ar–H), 7.35–7.30 (m, 3H, Ar–H), 7.12 (td, J = 8.6, 1.6 Hz, 2H, Ar–H), 6.95 (s, 1H, CH); ^{13}C NMR (101 MHz, $CDCl_3$, 298 K) δ: 166.0 (d, $J_{C–F}$ = 254.4 Hz), 164.6 (C=O), 137.2, 132.6 (d, $J_{C–F}$ = 9.4 Hz), 132.0, 129.1, 129.0, 128.8, 128.4, 127.9, 126.2 (d, $J_{C–F}$ = 3.0 Hz), 122.2, 115.7 (d, $J_{C–F}$ = 22.0 Hz), 87.5 (C≡C), 85.6 (C≡C), 66.9 (CH); ^{19}F NMR (376 MHz, $CDCl_3$, 298 K) δ: -105.03 (Ar–F); IR ν_{max} (cm^{-1}): 3053, 2983, 2156 (C≡C), 1720 (carbonyl C=O stretching), 1602, 1508, 1490, 1442, 1321, 1232, 1153, 1062; HRMS (EI+) $[M]^+$ calculated for $[C_{22}H_{15}FO_2]^+$: calculated 330.1056, found 330.1050.

*Synthesis of 1-(4-methoxyphenyl)-3-(trimethylsilyl)prop-2-yn-1-yl 4-fluorobenzoate (**2h**):*

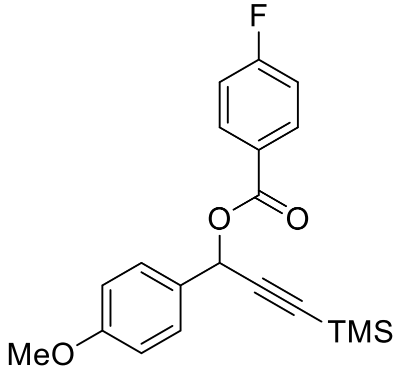

Synthesized in accordance with *General Procedure b* using trimethylsilylacetylene (2.5 mL, 17.6 mmol), *n*-BuLi (7.0 mL, 17.6 mmol), 4-methoxybenzaldehyde (2 g, 14.7 mmol), and 4-fluorobenzoyl chloride (2.8 mL, 17.6 mmol). The crude compound was purified *via* column chromatography using hexane/ethyl acetate (95:5 v/v) as eluent. The desired product (**2h**) was obtained as a yellow liquid. Yield: 4.0 g, 76%, 11.2 mmol.

^1H NMR (400 MHz, $CDCl_3$, 298 K) δ: 8.10–8.06 (m, 2H, Ar–H), 7.56–7.53 (m, 2H, Ar–H), 7.11–7.07 (m, 2H, Ar–H), 6.93 – 6.91 (m, 2H, Ar–H), 6.69 (s, 1H, CH), 3.82 (s, 3H, OCH_3), 0.21 (s, 9H, $Si(CH_3)_3$); ^{13}C NMR (101 MHz, $CDCl_3$, 298 K) δ: 166.0 (d, $J_{C–F}$ = 254.2 Hz), 164.5 (C=O), 160.2, 132.6 (d, $J_{C–F}$ = 9.3 Hz), 129.5, 129.3, 126.3 (d, $J_{C–F}$ = 3.0 Hz), 115.6 (d, $J_{C–F}$ = 22.1 Hz), 114.1, 101.5 (C≡C), 92.6 (C≡C), 66.4 (CH_3), 55.4 (CH), -0.1 ($Si(CH_3)_3$); ^{19}F NMR (376 MHz, $CDCl_3$, 298 K) δ: -105.31 (Ar–F); IR ν_{max} (cm^{-1}): 3062, 2960, 2177 (C≡C), 1720 (C=O), 1602, 1506, 1454, 1411, 1345, 1256, 1125, 1054; HRMS (ES+) $[M+Na]^+$ $[C_{20}H_{21}O_3FNaSi]^+$: calculated 379.1142, found 379.1155.

*Synthesis of 1-(2,6-difluorophenyl)-3-phenylprop-2-yn-1-yl 4-fluorobenzoate (**2i**):*

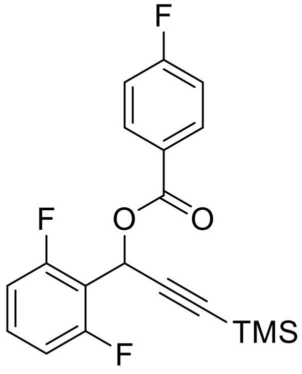

Synthesized in accordance with *General Procedure b* using trimethylsilylacetylene (2.4 mL, 16.9 mmol), *n*-BuLi (6.8 mL, 16.9 mmol), 2,6-difluorobenzaldehyde (2 g, 14.1 mmol), and 4-fluorobenzoyl chloride (2.7 mL, 16.9 mmol). The crude compound was purified *via* column chromatography using hexane/ethyl acetate (95:5 v/v) as eluent. The desired product (**2i**) was obtained as a yellow solid. Yield: 3.6 g, 70%, 9.8 mmol.

^1H NMR (400 MHz, $CDCl_3$, 298 K) δ: 8.10 (dd, J = 8.3, 5.6 Hz, 2H, Ar–H), 7.36–7.29 (m, 1H, Ar–H), 7.10 (t, J = 8.6 Hz, 2H, Ar–H), 7.01 – 7.00 (m, 1H, Ar–H), 6.94 (t, J = 8.2 Hz, 2H, Ar–H), 0.18 (s, 9H, $Si(CH_3)_3$); ^{13}C NMR (101 MHz, $CDCl_3$, 298 K) δ: 166.1 (d, $J_{C–F}$ = 254.5 Hz), 164.1 (C=O), 161.1 (dd, $J_{C–F}$ = 253.8, 6.8 Hz), 132.7 (d, $J_{C–F}$ = 9.5 Hz), 131.0 (t, $J_{C–F}$ = 10.3 Hz), 125.9 (d, $J_{C–F}$ = 3.0 Hz), 115.7 (d, $J_{C–F}$ = 21.9 Hz), 114.1 (t, $J_{C–F}$ = 16.5 Hz), 112.0 (d, $J_{C–F}$ = 25.3 Hz), 99.3 (C≡C), 92.1 (C≡C), 56.9 (CH), -0.2 ($Si(CH_3)_3$); ^{19}F NMR (376 MHz, $CDCl_3$, 298 K) δ: -104.91 (1F, Ar–F), -112.30 (2F, Ar–F); IR ν_{max} (cm^{-1}): 3062, 2960, 2177 (C≡C), 1720 (C=O), 1602, 1506, 1454, 1411, 1345, 1256, 1125, 1054; HRMS (ES+) $[M+Na]^+$ $[C_{20}H_{17}O_2F_3NaSi]^+$: calculated 385.0839, found 385.0848.

*Synthesis of (E)-1,3-diphenylallyl 2,2,2-trifluoroacetate (**2j**):*

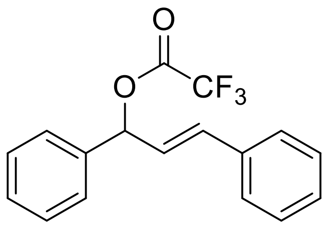

Pyridine (1.4 mL, 14.3 mmol, 1.5 equiv.) was added to a stirred CH_2Cl_2 (25 mL) solution of (*E*)-1,3-diphenylprop-2-en-1-ol (2 g) at 0 °C. The reaction mixture was allowed to stir for 15 min under nitrogen at same temperature. Trifluoroacetic anhydride (2 mL, 14.3 mmol, 1.5 equiv.) was added to the reaction mixture dropwise at 0 °C. The reaction mixture was allowed to stir over night at ambient temperature and quenched the reaction with saturated aq. $NaHCO_3$ solution (1 × 30 mL). The organic compounds were extracted with ethyl acetate (3 × 25 mL), the combined organic fractions were washed with brine solution (1 × 30 mL), dried over $MgSO_4$ and concentrated using vacuum. The crude compound was purified *via* column chromatography using silica gel (Merck, 60 Å, 230–400 mesh particle size) and hexane/ethyl acetate as eluent. The desired compound was obtained as thick liquid which was recrystallized using pentane at -30 °C. White solid was obtained as pure compound. Yield: 2 g, 71%, 6.7 mmol. ^{1}H NMR (500 MHz, $CDCl_3$, 298 K) δ: 7.46–7.43 (m, 2H, Ar–H), 7.41–7.36 (m, 4H, Ar–H), 7.33–7.29 (m, 3H, Ar–H), 7.26–7.23 (m, 1H, Ar–H), 6.61 (dd, *J* = 15.8, 5.0 Hz, 1H, CH), 6.36 (ddd, *J* = 26.2, 15.9, 7.1 Hz, 1H, CH), 5.11 (dd, *J* = 9.6, 7.2 Hz, 1H, CH); ^{13}C NMR (126 MHz, $CDCl_3$, 298 K) δ: 141.39, 141.31, 136.7 (d, $J_{C–F}$ = 2.1 Hz), 131.6 (d, $J_{C–F}$ = 22.7 Hz), 130.5 (d, $J_{C–F}$ = 21.1 Hz), 128.69, 128.67, 127.87 (d, $J_{C–F}$ = 1.9 Hz), 127.82, 127.2, 126.7 (d, $J_{C–F}$ = 2.3 Hz), 79.3, 79.2; ^{19}F NMR (471 MHz, $CDCl_3$, 298 K) δ: -75.15 (CF_3); IR ν_{max} (cm^{-1}): 3061, 3026, 1651, 1598, 1492, 1448, 1296, 1093, 1068, 1024.

1.4 Synthesis of diaryl esters

General Procedure c: Diaryl alcohol (1 equiv.) was dissolved in pyridine at 0 °C. Acyl chloride (1.2 equiv.) was added to the reaction mixture drop wise at 0 °C The mixture was allowed to stir at ambient temperature overnight. The reaction was quenched with water and extracted with ethyl acetate (3 × 25 mL). The combined organic fractions were washed with saturated brine solution (1 × 25 mL) and dried over $MgSO_4$. All volatiles were removed *in vacuo* and the crude compound was purified *via* column chromatography using silica gel (Merck, 60 Å, 230–400 mesh particle size) and hexane/ethyl acetate as eluent.

1.4.1 Synthesis and spectral characterization of diaryl ester compounds

*Synthesis of bis(4-fluorophenyl)methyl 4-fluorobenzoate (**2k**):*

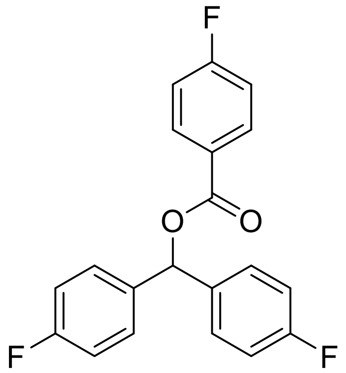

Synthesized in accordance with *General Procedure c* using 4-fluorobenzoyl chloride (2.7 mL, 23.2 mmol.), bis(4-fluorophenyl)methanol (4.4 g, 20.0 mmol), and pyridine (25 mL). All volatiles were removed *in vacuo* and the crude compound was purified *via* column chromatography using silica gel and hexane/ethyl acetate (20:1 v/v) as eluent: The desired product (**2k**) was obtained as a white solid. Yield: 5.81 g, 85%, 17.0 mmol.

^{1}H NMR (400 MHz, $CDCl_3$, 298 K) δ: 8.15–8.12 (m, 2H, Ar–H), 7.39–7.36 (m, 4H, Ar–H), 7.16–7.12 (m, 2H, Ar–H), 7.08–7.04 (m, 5H, Ar–H and CH, not able to detect distinct singlet peak for CH); ^{13}C NMR (101 MHz, $CDCl_3$, 298 K) δ: 166.1 (d, $J_{C\text{–}F}$ = 254.7 Hz), 164.8 (C=O), 162.6 (d, $J_{C\text{–}F}$ = 247.3 Hz), 135.8 (d, $J_{C\text{–}F}$ = 3.3 Hz), 132.4 (d, $J_{C\text{–}F}$ = 9.4 Hz), 129.0 (d, $J_{C\text{–}F}$ = 8.3 Hz), 126.2 (d, $J_{C\text{–}F}$ = 3.0 Hz), 115.8 (d, $J_{C\text{–}F}$ = 22.2 Hz), 115.7 (d, $J_{C\text{–}F}$ = 21.6 Hz), 76.4 (CH); ^{19}F NMR (376 MHz, $CDCl_3$, 298 K) δ: -104.80 (1F, Ar–F), -113.67 (2F, Ar–F); IR ν_{max} (cm^{-1}): 3116, 3074, 1724 (C=O), 1602, 1504, 1413, 1340, 1301, 1265, 1186, 1099; HRMS (EI+) $[M]^+$ $[C_{20}H_{13}O_2F_3]^+$: calculated 342.0868, found 342.0871.

*Synthesis of benzhydryl 4-fluorobenzoate (**2l**):*[8]

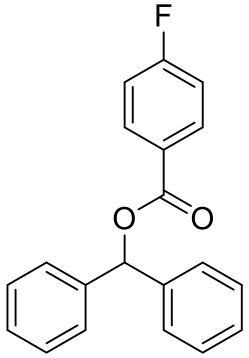

Synthesized in accordance with *General Procedure c* using 4-fluorobenzoyl chloride (2.7 ml, 23.2 mmol), diphenylmethanol (3.68 g, 20.0 mmol), and pyridine (25 ml). All volatiles were removed *in vacuo* and the crude product was purified *via* column chromatography using silica gel and hexane/ethyl acetate (20:1 v/v) as eluent. The desired product (**2l**) was obtained as a white solid. Yield: 5.49 g, 89%, 17.8 mmol.

^{1}H NMR (500 MHz, $CDCl_3$, 298 K) δ: 8.18–8.15 (m, 2H, Ar–H), 7.44–7.42 (m, 4H, Ar–H), 7.38–7.35 (m, 4H, Ar–H), 7.32–7.30 (m, 2H, Ar–H), 7.15–7.11 (m, 3H, Ar–H and CH); ^{13}C NMR (126 MHz, $CDCl_3$, 298 K) δ: 166.0 (d, $J_{C\text{–}F}$ = 254.3 Hz), 164.7 (C=O), 143.9, 140.2, 132.49 (d, $J_{C\text{–}F}$ = 9.3 Hz), 128.7, 128.6, 127.7, 127.2, 126.68, 126.61, 126.5, 115.7 (d, $J_{C\text{–}F}$ = 22.0 Hz), 77.7 (CH); ^{19}F NMR (471 MHz, $CDCl_3$, 298 K) δ: -105.19 (Ar–F); IR ν_{max} (cm^{-1}):

3026, 3030, 1716 (C=O), 1598, 1504, 1454, 1411, 1361, 1294, 1184, 1105, 1089, 1014; HRMS (EI+) $[M]^+$ $[C_{20}H_{15}O_2F]^+$: calculated 306.1056, found: 306.1056.

*Synthesis of bis(4-methoxyphenyl)methyl 4-fluorobenzoate (**2m**):*

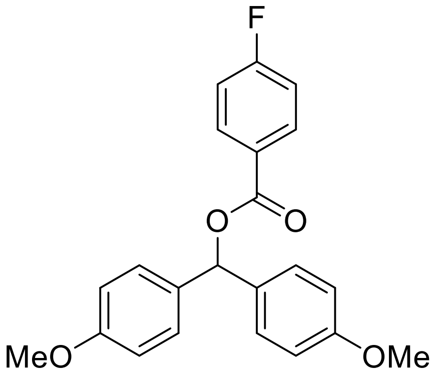

Synthesized in accordance with *General Procedure c* using 4-fluorobenzoyl chloride (2.74 mL, 23.2 mmol), bis(4-methoxyphenyl)methanol (4.86 g, 20.0 mmol), and pyridine (25 ml). All volatiles were removed *in vacuo* and the crude product was purified *via* column chromatography using silica gel and hexane/ethyl acetate (90:10 v/v) as eluent. The desired product (**2m**) was obtained as a colorless oil. Yield: 5.46 g, 76%, 14.9 mmol.

^{1}H NMR (500 MHz, $CDCl_3$, 298 K) δ: 8.13 (ddd, J = 8.9, 5.4, 1.4 Hz, 2H, Ar–H), 7.34–7.32 (m, 4H, Ar–H), 7.12 (td, J = 8.5, 1.4 Hz, 2H, Ar–H), 7.04 (s, 1H, CH), 6.90–6.87 (m, 4H, Ar–H), 3.80 (s, 6H, OCH_3); ^{13}C NMR (126 MHz, $CDCl_3$, 298 K) δ: 165.9 (d, $J_{C–F}$ = 254.0 Hz), 164.8 (C=O), 159.4, 132.6, 132.4 (d, $J_{C–F}$ = 9.3 Hz), 128.6, 126.7 (d, $J_{C–F}$ = 3.0 Hz), 115.6 (d, $J_{C–F}$ = 22.0 Hz), 114.0, 77.1 (CH), 55.4 (OCH_3); ^{19}F NMR (376 MHz, $CDCl_3$, 298 K) δ: -105.46 (Ar–F); IR ν_{max} (cm^{-1}): 3116, 3074, 1724 (C=O), 1002, 1413, 1340, 1301, 1263, 1186, 1099, 1014; HRMS (EI+) $[M+Na]^+$ $[C_{22}H_{19}O_4FNa]^+$: calculated 389.1165, found 389.1166.

*Synthesis of (4-chlorophenyl)(phenyl)methyl 4-fluorobenzoate (**2n**):*

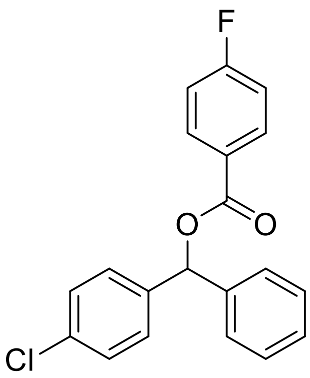

Synthetized in accordance with *General Procedure c* using 4-fluorobenzoyl chloride (2.7 mL, 23.2 mmol, 1.16 equiv.), 4-chlorobenzohydrol (4.37 g, 20.0 mmol, 1 equiv.), and pyridine (25 ml). All volatiles were removed *in vacuo* and the crude product was purified *via* column chromatography using silica gel and hexane/ethyl acetate (90:10 v/v) as eluent. The desired product (**2n**) was obtained as a white solid. Yield 5.59 g, 82%, 16.4 mmol.

^{1}H NMR (500 MHz, $CDCl_3$, 298 K) δ: 8.17–8.11 (m, 2H, Ar–H), 7.42–7.30 (m, 9H, Ar–H), 7.16–7.11 (m, 2H, Ar–H), 7.07 (s, 1H, CH); ^{13}C NMR (126 MHz, $CDCl_3$, 298 K) δ: 166.1 (d, $J_{C–F}$ = 254.6 Hz), 164.6 (C=O), 139.7, 138.8, 134.1, 132.4 (d, $J_{C–F}$ = 9.4 Hz), 128.9, 128.8, 128.6, 128.4,

127.2, 126.3 (d, $J_{C–F}$ = 3.0 Hz), 115.8 (d, $J_{C–F}$ = 22.1 Hz), 77.0 (CH); ^{19}F NMR (471 MHz, $CDCl_3$, 298 K) δ: -104.96 (Ar–F); IR ν_{max} (cm^{-1}): 3053, 2970, 1722 (C=O), 1602, 1506, 1490, 1415, 1367, 1263, 1232, 1151, 1105, 1087, 1012; HRMS (EI+) $[M]^+$ $[C_{20}H_{14}ClFO_2]^+$: calculated 340.0666, found 340.0661.

*Synthesis of phenyl(p-tolyl)methyl 4-fluorobenzoate (**2o**):*

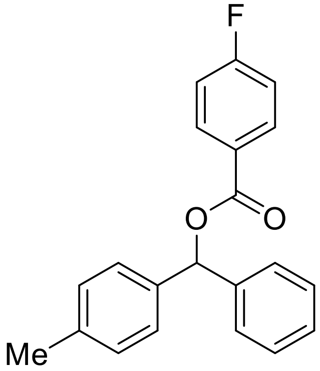

Synthetized in accordance with *General Procedure c* using 4-fluorobenzoyl chloride (2.7 mL, 23.2 mmol, 1.16 equiv.), (4-methylphenyl)(phenyl)methanol (3.97 g, 20 mmol, 1 equiv.), and pyridine (25 ml). All volatiles were removed *in vacuo* and the crude product was purified *via* column chromatography using silica gel and hexane/ethyl acetate (90:10 v/v) as eluent. The desired product (**2o**) was obtained as a white solid. Yield: 4.99 g. 78%, 15.6 mmol.

^{1}H NMR (500 MHz, $CDCl_3$, 298 K) δ: 8.18–8.13 (m, 2H, Ar–H), 7.42 (d, J = 7.1 Hz, 2H, Ar–H), 7.36 (t, J = 7.5 Hz, 2H, Ar–H), 7.31 (t, J = 6.8 Hz, 3H, Ar–H), 7.17 (d, J = 7.7 Hz, 2H, Ar–H), 7.13 (t, J = 8.7 Hz, 2H, Ar–H), 7.08 (s, 1H, CH), 2.34 (s, 3H, CH_3); ^{13}C NMR (126 MHz, $CDCl_3$, 298 K) δ: 166.0 (d, $J_{C–F}$ = 254.1 Hz), 164.8 (C=O), 140.4, 138.0, 137.3, 132.5 (d, $J_{C–F}$ = 9.3 Hz), 129.4, 128.7, 128.1, 127.3, 127.1, 126.7 (d, $J_{C–F}$ = 3.0 Hz), 115.7 (d, $J_{C–F}$ = 22.0 Hz), 77.7 (CH), 21.3 (CH_3); ^{19}F NMR (471 MHz, $CDCl_3$, 298 K) δ: -105.39 (Ar–F); IR ν_{max} (cm^{-1}): 3053, 1718 (C=O), 1602, 1506, 1450, 1411, 1309, 1261, 1236, 1151, 1107, 1087, 1014; HRMS (EI+) $[M]^+$ $[C_{21}H_{17}FO_2]^+$: calculated 320.1213, found 320.1207.

*Synthesis of cyclohexyl(phenyl)methyl 4-fluorobenzoate (**2p**):*

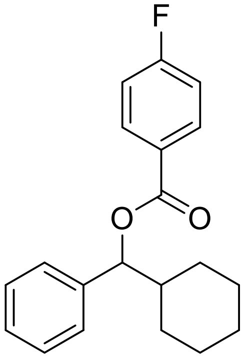

Synthetized in accordance with *General Procedure c* using 4-fluorobenzoyl chloride (3.4 mL, 28.9 mmol, 1.1 equiv.), cyclohexyl(phenyl)methanol (5 g, 26.3 mmol, 1 equiv.), and pyridine (18 mL). All volatiles were removed *in vacuo* and the crude compound was purified *via* column chromatography using silica gel and hexane/ethyl acetate (95:5 v/v) as eluent. The desired product (**2p**) was obtained as a white solid. Yield: 5.4 g, 17.4 mmol, 66%.

^{1}H NMR (500 MHz, $CDCl_3$, 298 K) δ: 8.12–8.08 (m, 2H, Ar–H), 7.37–7.32 (m, 4H, Ar–H), 7.29–7.26 (m, 1H, Ar–H), 7.13–7.10 (m, 2H, Ar–H), 5.73 (d, J = 7.5 Hz, 1H, CH), 1.95–1.88 (m, 2H, CH_2), 1.78–1.65 (m, 3H, CH_2), 1.50–1.48 (m, 1H, CH), 1.29–1.12 (m, 4H, CH_2), 1.02 (qd, J = 12.3, 3.8 Hz, 1H, CH); ^{13}C NMR (126 MHz, $CDCl_3$, 298 K) δ: 165.8 (d, $J_{C–F}$ = 254.5 Hz), 165.0, 139.7, 132.2 (d, $J_{C–F}$ = 10.0 Hz), 128.3, 127.9, 127.1, 126.9 (d, $J_{C–F}$ = 3.0 Hz), 115.6 (d, $J_{C–F}$ = 22.6 Hz), 81.1, 43.3, 29.2, 29.1, 26.4, 26.05, 26.00; ^{19}F NMR (471 MHz, $CDCl_3$, 298 K) δ: -105.88 (Ar–F); IR ν_{max} (cm^{-1}): 2939, 2848, 1720 (C=O), 1600, 1504, 1448, 1292, 1253, 1238, 1219, 1153, 1107, 1087, 1053, 1012; HRMS (EI+) $[M]^+$ $[C_{20}H_{21}O_2F]^+$: calculated 312.1526, found 312.1519.

2. Product Characterization

2.1 General procedure d for C-C coupling reactions: Tris(pentafluorophenyl)borane ($B(C_6F_5)_3$) (10–20 mol%) was dissolved in TFT (0.5 mL) and added to a TFT solution (0.5 mL) of the α-diazoester (1 equiv.). The aryl ester (0.2 mmol, 1.1 equiv.) was also dissolved in TFT (0.5 mL) and then added to the reaction mixture dropwise. The reaction tube was sealed in the glove box under nitrogen atmosphere and heated at 65 °C for 18–24 h. All volatiles were removed *in vacuo* and the crude compound was purified *via* preparative thin layer chromatography using hexane/ethyl acetate as eluent.

2.2 Synthesis and spectral characterization of products

*Synthesis of dimethyl 2-(1-(4-fluorophenyl)-3-(trimethylsilyl)prop-2-yn-1-ylidene)malonate (**3a**):*

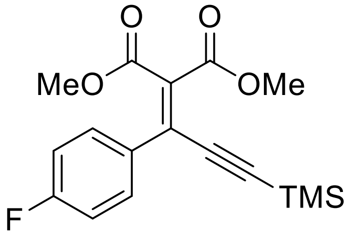

Synthesized in accordance with *General Procedure d* using $B(C_6F_5)_3$ (5 mg, 0.01 mmol), diazoester **1a** (16 mg, 0.10 mmol), and alkynyl aryl ester **2a** (38 mg, 0.11 mmol) in TFT to afford **3a**. The crude reaction mixture was purified *via* preparative thin layer chromatography using hexane/ethyl acetate (90:10 v/v) as eluent. The desired compound **3a** was obtained as a pale-yellow solid. Yield: 27 mg. 81%, 0.08 mmol.

^{1}H NMR (400 MHz, $CDCl_3$, 298 K) δ: 7.43–7.39 (m, 2H, Ar–H), 7.07–7.02 (m, 2H, Ar–H), 3.85 (s, 3H, $COOCH_3$), 3.61 (s, 3H, $COOCH_3$), 0.23 (s, 9H, $Si(CH_3)_3$); ^{13}C NMR (101 MHz, $CDCl_3$, 298 K) δ: 165.5 (C=O), 164.1 (C=O), 163.5 (d, $J_{C–F}$ = 251.4 Hz), 135.7, 132.8 (d, $J_{C–F}$ = 3.4 Hz), 131.6, 130.2 (d, $J_{C–F}$ = 8.6 Hz), 115.6 (d, $J_{C–F}$ = 21.9 Hz), 111.9 (C≡C), 102.0 (C≡C), 52.6 (OCH_3),

-0.3 ($Si(CH_3)_3$); ^{19}F NMR (376 MHz, $CDCl_3$, 298 K) δ: -110.80 (Ar–F); IR ν_{max} (cm^{-1}): 3027, 2954, 2232 (C≡C), 1724 (C=O), 1600, 1575, 1508, 1436, 1313, 1301, 1249, 1217, 1161, 1056; HRMS (EI+) $[M]^+$ $[C_{17}H_{19}FO_4Si]^+$: calculated 334.1037, found 334.1031.

*Synthesis of dibenzyl 2-(1-(4-fluorophenyl)-3-(trimethylsilyl)prop-2-yn-1-ylidene)malonate (**3b**):*

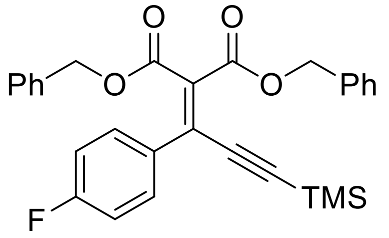

Synthesized in accordance with *General Procedure d* using $B(C_6F_5)_3$ (5 mg, 0.01 mmol), diazoester **1c** (31 mg, 0.10 mmol), and alkynyl aryl ester **2a** (38 mg, 0.11 mmol) in TFT to afford **3b** The crude reaction mixture was purified *via* preparative thin layer chromatography using hexane/ethyl acetate (90:10 v/v) as eluent. The desired compound **3b** was obtained as a pale-yellow liquid. Yield: 34 mg, 71%, 0.07 mmol.

^{1}H NMR (400 MHz, $CDCl_3$, 298 K) δ: 7.16–7.14 (m, 6H, Ar–H), 7.08–7.06 (m, 4H, Ar–H), 6.89 –6.86 (m, 2H, Ar–H), 6.72–6.69 (m, 2H, Ar–H), 5.10 (s, 2H, CH_2), 4.83 (s, 2H, CH_2), 0.00 (s, 9H, $Si(CH_3)_3$); ^{13}C NMR (101 MHz, $CDCl_3$, 298 K) δ: 164.9 (C=O), 163.5 (d, $J_{C–F}$ = 251.4 Hz), 163.3 (C=O), 136.0, 135.5, 134.8, 132.8 (d, $J_{C–F}$ = 3.4 Hz), 131.7, 130.3 (d, $J_{C–F}$ = 8.6 Hz), 128.7, 128.6, 128.6, 128.5, 128.3, 128.1, 115.5 (d, $J_{C–F}$ = 22.0 Hz), 112.4 (C≡C), 102.1 (C≡C), 67.4 (CH_2), 67.2 (CH_2), -0.4 ($Si(CH_3)_3$); ^{19}F NMR (376 MHz, $CDCl_3$, 298 K) δ: -110.81 (Ar–F); IR ν_{max} (cm^{-1}): 3016, 2933, 2210 (C≡C), 1721 (C=O), 1603, 1565, 1525, 1416, 1353, 1310, 1275, 1235, 1155, 1035; HRMS (ES+) $[M+Na]^+$ $[C_{29}H_{27}FO_4SiNa]^+$: calculated 509.1560, found 509.1562.

*Synthesis of diisopropyl 2-(1-(4-fluorophenyl)-3-(trimethylsilyl)prop-2-yn-1-ylidene)malonate (**3c**):*

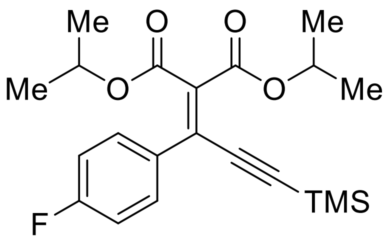

Synthesized in accordance with *General Procedure d* using $B(C_6F_5)_3$ (5 mg, 0.01 mmol), diazoester **1b** (22 mg, 0.10 mmol), and alkynyl aryl ester **2a** (38 mg, 0.11 mmol) in TFT to afford **3c**. The crude reaction mixture was purified *via* preparative thin layer chromatography using hexane/ethyl acetate (90:10 v/v) as eluent. The desired compound **3c** was obtained as a pale-yellow liquid. Yield: 32 mg, 83%, 0.08 mmol.

^{1}H NMR (500 MHz, $CDCl_3$, 298 K) δ: 7.44–7.40 (m, 2H, Ar–H), 7.04–7.01 (m, 2H, Ar–H), 5.18 (hept, J = 6.3 Hz, 1H, CH), 4.94 (hept, J = 6.3 Hz, 1H, CH), 1.33 (d, J = 6.3 Hz, 6H, CH_3), 1.08

(d, J = 6.3 Hz, 6H, CH_3), 0.22 (s, 9H, $Si(CH_3)_3$); ^{13}C NMR (126 MHz, $CDCl_3$, 298 K) δ: 164.5 (C=O), 163.4 (d, $J_{C–F}$ = 249.4 Hz), 163.3 (C=O), 134.1, 133.2 (d, $J_{C–F}$ = 3.3 Hz), 130.3 (d, $J_{C–F}$ = 8.4 Hz), 115.4 (d, $J_{C–F}$ = 21.8 Hz), 110.9 (C≡C), 102.2 (C≡C), 69.48 (CH), 69.41 (CH), 21.8 (CH_3), 21.4 (CH_3), −0.3 ($Si(CH_3)_3$); ^{19}F NMR (471 MHz, $CDCl_3$, 298 K) δ: -111.47 (Ar–F); IR ν_{max} (cm^{-1}): 2981, 2897, 2254 (C≡C), 1720 (C=O), 1610, 1541, 1456, 1412, 1325, 1247, 1217, 1135, 1051; HRMS (ES+) $[M+Na]^+$ $[C_{21}H_{27}FO_4SiNa]^+$: calculated 413.1560, found 413.1560.

*Synthesis of dimethyl 2-(1-phenyl-3-(trimethylsilyl)prop-2-yn-1-ylidene)malonate (**3d**):*

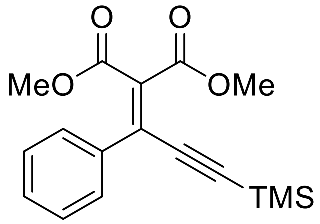

Synthesized in accordance with *General Procedure d* using $B(C_6F_5)_3$ (5 mg, 0.01 mmol), diazoester **1a** (16 mg, 0.10 mmol), and alkynyl aryl ester **2e** (36 mg, 0.11 mmol) in TFT to afford **3d**. The crude reaction mixture was purified *via* preparative thin layer chromatography using hexane/ethyl acetate (90:10 v/v) as eluent. The desired compound **3d** was obtained as a pale-yellow solid, Yield: 23 mg, 73%, 0.07 mmol.

^{1}H NMR (400 MHz, $CDCl_3$, 298 K) δ: 7.43–7.41 (m, 2H, Ar–H), 7.38–7.35 (m, 3H, Ar–H), 3.86 (s, 3H, $COOCH_3$), 3.58 (s, 3H, $COOCH_3$), 0.23 (s, 9H, $Si(CH_3)_3$); ^{13}C NMR (101 MHz, $CDCl_3$, 298 K) δ: 165.7 (C=O), 164.2 (C=O), 136.9, 131.6, 129.7, 128.4, 128.1, 111.8 (C≡C), 102.1 (C≡C), 52.6 (CH_3), 52.5 (CH_3), -0.3 ($Si(CH_3)_3$); IR ν_{max} (cm^{-1}): 3010, 2958, 2254 (C≡C), 1732 (C=O), 1651, 1558, 1516, 1433, 1410, 1372, 1321, 1247, 1150, 1052; HRMS (ES+) $[M+Na]^+$ $[C_{17}H_{20}O_4SiNa]^+$: calculated 339.1029, found 339.1032.

*Synthesis of dibenzyl 2-(1-phenyl-3-(trimethylsilyl)prop-2-yn-1-ylidene)malonate (**3e**):*

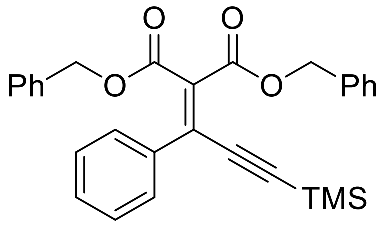

Synthesized in accordance with *General Procedure d* using $B(C_6F_5)_3$ (5 mg, 0.01 mmol), diazoester **1c** (31 mg, 0.10 mmol), and alkynyl aryl ester **2e** (36 mg, 0.11 mmol) in TFT to afford **3e**. The crude reaction mixture was purified *via* preparative thin layer chromatography using hexane/ethyl acetate (90:10 v/v) as eluent. The desired compound **3e** was obtained as a light-yellow solid. Yield: 32 mg, 68%, 0.07 mmol.

^{1}H NMR (400 MHz, $CDCl_3$, 298 K) δ: 7.22–7.12 (m, 7H, Ar–H), 7.10–7.01 (m, 6H, Ar–H), 6.83–6.81 (m, 2H, Ar–H), 5.10 (s, 2H, CH_2), 4.80 (s, 2H, CH_2), 0.00 (s, 9H, $Si(CH_3)_3$); ^{13}C NMR (101 MHz, $CDCl_3$, 298 K) δ: 165.1 (C=O), 163.4 (C=O), 137.2, 137.0, 135.6, 134.9, 131.6, 129.7,

128.6, 128.5, 128.46, 128.45, 128.35, 128.32, 128.2, 128.1, 112.3 (C≡C), 102.3 (C≡C), 67.4 (CH_3), 67.2 (CH_3), -0.3 ($Si(CH_3)_3$); IR ν_{max} (cm^{-1}): 2981, 2925, 2254 (C≡C), 1740 (C=O), 1651, 1532, 1512, 1492, 1456, 1417, 1355, 1249, 1201, 1162, 1048; HRMS (ES+) $[M+Na]^+$ $[C_{29}H_{28}O_4SiNa]^+$: calculated 491.1655, found 491.1660.

*Synthesis of diisopropyl 2-(1-phenyl-3-(trimethylsilyl)prop-2-yn-1-ylidene)malonate (**3f**):*

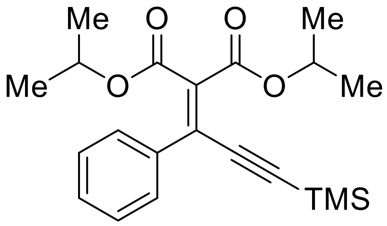

Synthesized in accordance with *General Procedure d* using $B(C_6F_5)_3$ (5 mg, 0.01 mmol), diazoester **1b** (22 mg, 0.10 mmol), and alkynyl aryl ester **2e** (36 mg, 0.11 mmol) in TFT to afford **3f**. The crude reaction mixture was purified *via* preparative thin layer chromatography using hexane/ethyl acetate (90:10 v/v) as eluent. The desired compound **3f** was obtained as a pale colorless liquid. Yield: 28 mg, 75%, 0.07 mmol. ^{1}H NMR (400 MHz, $CDCl_3$, 298 K) δ: 7.44–7.41 (m, 2H, Ar–H), 7.34 (dt, *J* = 3.7, 1.4 Hz, 3H, Ar–H), 5.19 (hept, *J* = 6.5 Hz, 1H, CH), 4.92 (hept, *J* = 6.5 Hz, 1H, CH), 1.33 (d, *J* = 6.3 Hz, 6H, CH_3), 1.04 (d, *J* = 6.3 Hz, 6H, CH_3), 0.22 (s, 9H, $Si(CH_3)_3$); ^{13}C NMR (101 MHz, $CDCl_3$, 298 K) δ: 164.7 (C=O), 163.4 (C=O), 137.3, 135.2, 133.2, 129.3, 128.3, 110.7 (C≡C), 102.3 (C≡C), 69.3 (CH), 69.2 (CH), 21.8 (CH_3), 21.3 (CH_3), -0.2 ($Si(CH_3)_3$; IR ν_{max} (cm^{-1}): 3030, 2981, 2250 (C≡C), 1714 (C=O), 1651, 1541, 1512, 1492, 1391, 1303, 1246, 1180, 1145, 1049; HRMS (ES+) $[M+Na]^+$ $[C_{21}H_{28}O_4SiNa]^+$: calculated 395.1655, found 395.1667.

*Synthesis of dimethyl 2-(1-(4-chlorophenyl)-3-(trimethylsilyl)prop-2-yn-1-ylidene)malonate (**3g**):*

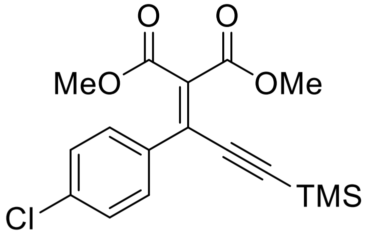

Synthesized in accordance with *General Procedure d* using $B(C_6F_5)_3$ (5 mg, 0.01 mmol), diazoester **1a** (16 mg, 0.10 mmol), and alkynyl aryl ester **2d** (40 mg, 0.11 mmol) in TFT to afford **3g** The crude reaction mixture was purified *via* preparative thin layer chromatography using hexane/ethyl acetate (90:10 v/v) as eluent. The desired compound **3g** was obtained as an off-white solid. Yield: 28 mg, 80%, 0.08 mmol.
^{1}H NMR (400 MHz, $CDCl_3$, 298 K) δ: 7.37–7.31 (m, 4H, Ar–H), 3.85 (s, 3H, $COOCH_3$), 3.61 (s, 3H, $COOCH_3$), 0.23 (s, 9H, $Si(CH_3)_3$); ^{13}C NMR (101 MHz, $CDCl_3$, 298 K) δ: 165.3 (C=O), 164.1 (C=O), 135.8, 135.6, 135.3, 131.9, 129.5, 128.7, 112.1 (C≡C), 101.8 (C≡C), 52.7 (OCH_3), 52.6 (OCH_3), –0.3 ($Si(CH_3)_3$); IR ν_{max} (cm^{-1}): 3033, 2915, 2215 (C≡C), 1722 (C=O), 1640, 1530, 1525,

1465, 1409, 1376, 1314, 1255, 1221, 1162, 1039; HRMS (EI+) $[M]^+$ $[C_{17}H_{19}O_4ClSi]^+$: calculated 350.0741, found 350.0738.

*Synthesis of diisopropyl 2-(1-(4-chlorophenyl)-3-(trimethylsilyl)prop-2-yn-1-ylidene)malonate (**3h**):*

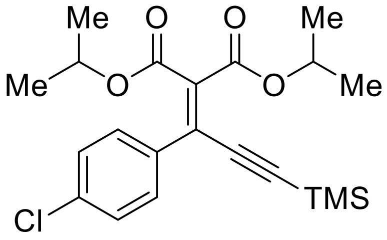

Synthesized in accordance with *General Procedure d* using $B(C_6F_5)_3$ (5 mg, 0.01 mmol), diazoester **1b** (22 mg, 0.10 mmol), and alkynyl aryl ester **2d** (40 mg, 0.11 mmol) in TFT to afford **3h**. The crude reaction mixture was purified *via* preparative thin layer chromatography using hexane/ethyl acetate (90:10 v/v) as eluent. The desired compound **3h** was obtained as a pale-yellow solid. Yield: 34 mg, 83%, 0.07 mmol.

^{1}H NMR (400 MHz, $CDCl_3$, 298 K) δ: 7.38–7.35 (m, 2H, Ar–H), 7.33–7.30 (m, 2H, Ar–H), 5.18 (hept, *J* = 6.4 Hz, 1H, CH), 4.94 (hept, *J* = 6.4 Hz, 1H, CH), 1.33 (d, *J* = 6.3 Hz, 6H, CH_3), 1.09 (d, *J* = 6.3 Hz, 6H, CH_3), 0.21 (s, 9H, $Si(CH_3)_3$); ^{13}C NMR (101 MHz, $CDCl_3$, 298 K) δ: 164.3 (C=O), 163.2 (C=O), 135.6, 135.5, 134.0, 133.4, 129.7, 128.5, 111.0 (C≡C), 101.9 (C≡C), 69.54 (CH) 69.50 (CH), 21.8 (CH_3), 21.4 (CH_3), -0.3 ($Si(CH_3)_3$); IR ν_{max} (cm^{-1}): 3010, 2915, 2230 (C≡C), 1720 (C=O), 1643, 1560, 1526, 1461, 1423, 1350, 1322, 1241, 1221, 1152, 1065; HRMS (ES+) $[M+Na]^+$ $[C_{21}H_{27}ClO_4SiNa]^+$: calculated 429.1265, found 429.1265.

*Synthesis of dimethyl 2-(1-(4-bromophenyl)-3-(trimethylsilyl)prop-2-yn-1-ylidene)malonate (**3i**):*

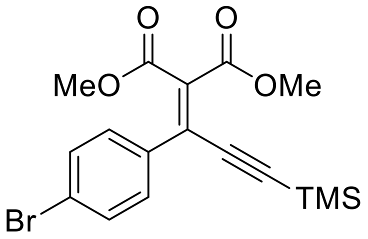

Synthesized in accordance with *General Procedure d* using $B(C_6F_5)_3$ (5 mg, 0.01 mmol), diazoester **1a** (16 mg, 0.10 mmol), and alkynyl aryl ester **2c** (45 mg, 0.11 mmol) in TFT to afford **3i**. The crude reaction mixture was purified *via* preparative thin layer chromatography using hexane/ethyl acetate (90:10 v/v) as eluent. The desired compound **3i** was obtained as a light-yellow solid. Yield: 34 mg, 87%, 0.08 mmol.

^{1}H NMR (400 MHz, $CDCl_3$, 298 K) δ: 7.53–7.50 (m, 2H, Ar–H), 7.33–7.28 (m, 2H, Ar–H), 3.88 (s, 3H, $COOCH_3$), 3.64 (s, 3H, $COOCH_3$), 0.25 (s, 9H, $Si(CH_3)_3$); ^{13}C NMR (101 MHz, $CDCl_3$, 298 K) δ: 165.3 (C=O), 164.1 (C=O), 135.79, 135.70, 131.9, 131.7, 129.8, 124.1, 112.2, 101.7, 52.73 (CH_3), 52.70 (CH_3), -0.3 ($Si(CH_3)_3$); IR ν_{max} (cm^{-1}): 3025, 2920, 2210 (C≡C), 1725 (C=O),

1650, 1545, 1512, 1478, 1412, 1382, 1325, 1245, 1201, 1182, 1047; HRMS (ES+) $[M+Na]^+$ $[C_{17}H_{19}BrO_4SiNa]^+$: calculated 417.0134, found 417.0139.

*Synthesis of diisopropyl 2-(1-(4-bromophenyl)-3-(trimethylsilyl)prop-2-yn-1-ylidene)malonate (**3j**):*

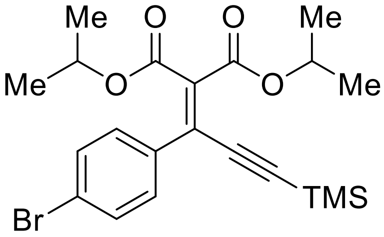

Synthesized in accordance with *General Procedure d* using $B(C_6F_5)_3$ (5 mg, 0.01 mmol), diazoester **1b** (22 mg, 0.10 mmol), and alkynyl aryl ester **2c** (45 mg, 0.11 mmol) in TFT to afford **3j**. The crude reaction mixture was purified *via* preparative thin layer chromatography using hexane/ethyl acetate (90:10 v/v) as eluent. The desired compound **3j** was obtained as a yellow liquid. Yield: 36 mg, 80%, 0.07 mmol.

^{1}H NMR (500 MHz, $CDCl_3$, 298 K) δ: 7.49–7.46 (m, 2H, Ar–H), 7.31–7.28 (m, 2H, Ar–H), 5.18 (hept, J = 6.3 Hz, 1H, CH), 4.94 (hept, J = 6.3 Hz, 1H, CH), 1.33 (d, J = 6.3 Hz, 6H, CH_3), 1.09 (d, J = 6.3 Hz, 6H, CH_3), 0.21 (s, 9H, $Si(CH_3)_3$); ^{13}C NMR (126 MHz, $CDCl_3$, 298 K) δ: 164.3 (C=O), 163.2 (C=O), 136.1, 134.0, 133.4, 131.5, 129.9, 123.7, 111.1 (C≡C), 101.9 (C≡C), 69.55 (CH), 69.51 (CH), 21.8 (CH_3), 21.4 (CH_3), -0.3 ($Si(CH_3)_3$); IR ν_{max} (cm^{-1}): 3030, 2970, 2252 (C≡C), 1722 (C=O), 1620, 1558, 1539, 1487, 1456, 1375, 1296, 1217, 1105, 1049; HRMS (ES+) $[M+Na]^+$ $[C_{21}H_{27}O_4BrSiNa]^+$: calculated 473.0760, found 473.0759.

*Synthesis of dimethyl 2-(1-(4-(trifluoromethyl)phenyl)-3-(trimethylsilyl)prop-2-yn-1-ylidene) malonate (**3k**):*

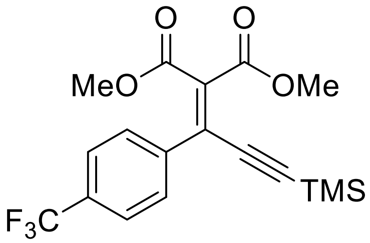

Synthesized in accordance with *General Procedure d* using $B(C_6F_5)_3$ (5 mg, 0.01 mmol), diazoester **1a** (16 mg, 0.10 mmol), and alkynyl aryl ester **2b** (43 mg, 0.11 mmol) in TFT to afford **3k**. The crude reaction mixture was purified *via* preparative thin layer chromatography using hexane/ethyl acetate (90:10 v/v) as eluent. The desired compound **3k** was obtained as a pale-yellow liquid. Yield: 25 mg, 65%, 0.06 mmol.

^{1}H NMR (400 MHz, $CDCl_3$, 298 K) δ: 7.63–7.61 (m, 2H, Ar–H), 7.53–7.51 (m, 2H, Ar–H), 3.87 (s, 3H, $COOCH_3$), 3.60 (s, 3H, $COOCH_3$), 0.23 (s, 9H, $Si(CH_3)_3$); ^{13}C NMR (101 MHz, $CDCl_3$, 298 K) δ: 164.9 (C=O), 164.0 (C=O), 140.3, 135.4, 132.8, 129.0, 128.5, 125.4 (q, J_{C-F} = 3.7 Hz), 112.7 (C≡C), 101.4 (C≡C), 52.8 (CH_3), 52.7 (CH_3), -0.3 ($Si(CH_3)_3$); ^{19}F NMR (376 MHz, $CDCl_3$,

298 K) δ: -62.83 (Ar–CF_3); IR ν_{max} (cm^{-1}): 3025, 2920, 2215 (C≡C), 1725 (C=O), 1650, 1545, 1512, 1478, 1412, 1382, 1325, 1245, 1201, 1182, 1047; HRMS (ES+) $[M+Na]^+$ $[C_{18}H_{19}F_3O_4SiNa]^+$: calculated 407.0902, found 407.0901.

*Synthesis of dimethyl 2-(1,3-diphenylprop-2-yn-1-ylidene)malonate (**3l**):*

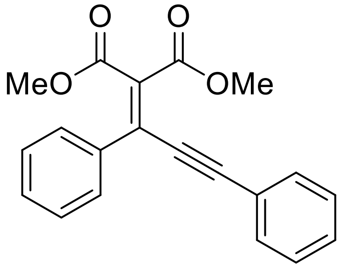

Synthesized in accordance with *General Procedure d* using $B(C_6F_5)_3$ (5 mg, 0.01 mmol), diazoester **1a** (16 mg, 0.10 mmol), and alkynyl aryl ester **2h** (36 mg, 0.11 mmol) in TFT to afford **3l**. The crude reaction mixture was purified *via* preparative thin layer chromatography using hexane/ethyl acetate (90:10 v/v) as eluent. The desired compound **3l** was obtained as a pale-yellow solid. Yield: 20 mg, 63%, 0.07 mmol.

^{1}H NMR (400 MHz, $CDCl_3$, 298 K) δ: 7.54–7.50 (m, 4H, Ar–H), 7.41–7.35 (m, 6H, Ar–H), 3.89 (s, 3H, $COOCH_3$), 3.60 (s, 3H, $COOCH_3$); ^{13}C NMR (101 MHz, $CDCl_3$, 298 K) δ: 166.1 (C=O), 164.1 (C=O), 137.7, 137.3, 132.3, 130.4, 129.8, 129.7, 128.59, 128.57, 128.0, 122.3, 105.0 (C≡C), 88.1 (C≡C), 52.65 (CH_3), 52.61 (CH_3); IR ν_{max} (cm^{-1}): 3014, 2970, 2252 (C≡C), 1734 (C=O), 1633, 1558, 1489, 1435, 1365, 1234, 1205, 1095, 1043; HRMS (EI+) $[M]^+$ $[C_{20}H_{16}O_4]^+$: calculated 320.1049, found 320.1040.

*Synthesis of diisopropyl 2-(1,3-diphenylprop-2-yn-1-ylidene)malonate (**3m**):*

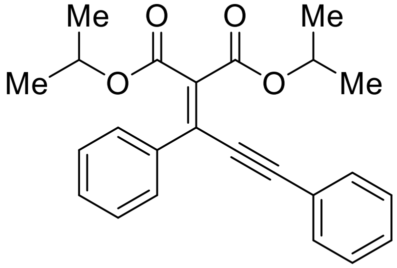

Synthesized in accordance with *General Procedure d* using $B(C_6F_5)_3$ (5 mg, 0.01 mmol), diazoester **1b** (22 mg, 0.10 mmol), and alkynyl aryl ester **2h** (36 mg, 0.11 mmol) in TFT to afford **3m**. The crude reaction mixture was purified *via* preparative thin layer chromatography using hexane/ethyl acetate (90:10 v/v) as eluent. The desired compound **3m** was obtained as an off-white solid. Yield: 25 mg, 67%, 0.07 mmol.

^{1}H NMR (500 MHz, $CDCl_3$, 298 K) δ: 7.52–7.49 (m, 4H, Ar–H), 7.39–7.32 (m, 6H, Ar–H), 5.22 (hept, J = 6.4 Hz, 1H, CH), 4.95 (hept, J = 6.4 Hz, 1H, CH), 1.34 (d, J = 6.3 Hz, 6H, CH_3), 1.08 (d, J = 6.3 Hz, 6H, CH_3); ^{13}C NMR (126 MHz, $CDCl_3$, 298 K) δ: 165.0 (C=O), 163.3 (C=O), 137.6, 136.0, 132.2, 132.1, 129.5, 129.4, 128.5, 128.4, 128.2, 122.5, 103.9 (C≡C), 88.2 (C≡C), 69.28 (CH), 69.25 (CH), 21.9 (CH_3), 21.4 (CH_3); IR ν_{max} (cm^{-1}): 2981, 2920, 2200 (C≡C), 1720

(C=O), 1651, 1558, 1512, 1490, 1412, 1396, 1247, 1211, 1178, 1037; HRMS (ES+) $[M+Na]^+$ $[C_{24}H_{24}O_4Na]^+$: calculated 399.1572, found 399.1581.

*Synthesis of dimethyl 2-(1-(4-fluorophenyl)-3-phenylprop-2-yn-1-ylidene)malonate (**3n**):*

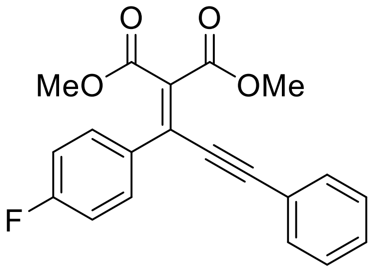

Synthesized in accordance with *General Procedure d* using $B(C_6F_5)_3$ (5 mg, 0.01 mmol), diazoester **1a** (16 mg, 0.10 mmol), and alkynyl aryl ester **2i** (38 mg, 0.11 mmol) in TFT to afford **3n** The crude reaction mixture was purified *via* preparative thin layer chromatography using hexane/ethyl acetate (90:10 v/v) as eluent. The desired compound **3n** was obtained as an off-white solid. Yield: 24 mg, 70%, 0.07 mmol.

^{1}H NMR (400 MHz, $CDCl_3$, 298 K) δ: 7.53–7.48 (m, 4H, Ar–H), 7.39–7.33 (m, 3H, Ar–H), 7.10–7.05 (m, 2H, Ar–H), 3.89 (s, 3H, $COOCH_3$), 3.63 (s, 3H, $COOCH_3$); ^{13}C NMR (101 MHz, $CDCl_3$, 298 K) δ: 165.8 (C=O), 164.1 (C=O), 163.6 (d, $J_{C–F}$ = 251.4 Hz), 136.3, 133.4 (d, $J_{C–F}$ = 3.5 Hz), 132.3, 130.8, 130.2 (d, $J_{C–F}$ = 8.5 Hz), 129.9, 128.6, 122.4, 115.6 (d, $J_{C–F}$ = 22.0 Hz), 104.9 (C≡C), 88.1 (C≡C), 52.57 (CH_3), 52.53 (CH_3); ^{19}F NMR (376 MHz, $CDCl_3$, 298 K) δ: -110.96 (Ar–F); IR ν_{max} (cm^{-1}): 3051, 2945, 2198 (C≡C), 1734 (C=O), 1637, 1598, 1541, 1506, 1489, 1408, 1375, 1332, 1278, 1203, 1190, 1089; HRMS (EI+) $[M]^+$ $[C_{20}H_{15}FO_4]^+$: calculated 338.0954, found 338.0949.

*Synthesis of methyl 2-(4-chlorophenyl)-3-(4-fluorophenyl)-5-(trimethylsilyl)pent-2-en-4-ynoate (**3o**):*

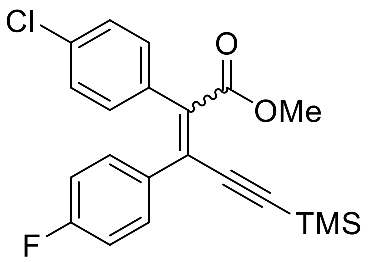

Synthetized in accordance with *General Procedure d* using $B(C_6F_5)_3$ (5 mg, 0.01 mmol), diazoester **1e** (21 mg, 0.10 mmol), and alkynyl aryl ester **2a** (38 mg, 0.11 mmol) in TFT to afford **3o**. The crude reaction mixture was purified *via* preparative thin layer chromatography using hexane/ethyl acetate (92:8 v/v) as eluent. The desired compound **3o** was obtained as a mixture of isomers (1:0.4) which appeared as a yellow liquid. Yield: 18 mg, 46%, 0.46 mmol.

^{1}H NMR (500 MHz, $CDCl_3$, 298 K) δ: 7.62–7.59 (m, Ar–H), 7.46–7.42 (m, Ar–H), 7.37–7.34 (m, Ar–H), 7.19–7.16 (m, Ar–H), 7.08–7.01 (m, Ar–H), 6.91–6.86 (m, Ar–H), 3.85 (s, CH_3, minor isomer), 3.56 (s, 3H, CH_3, major isomer), 0.24 (s, $Si(CH_3)_3$, minor isomer), 0.12 (s, 9H, $Si(CH_3)_3$,

major isomer); ^{13}C NMR (126 MHz, $CDCl_3$, 298 K) δ: 169.1 (C=O), 168.5 (C=O), 163.0 (d, $J_{C–F}$ = 239.4 Hz), 162.5 (d, $J_{C–F}$ = 252 Hz), 139.6, 139.4, 134.7, 134.4, 134.39, 134.38, 134.35, 133.4, 132.49, 132.46, 131.6 (d, $J_{C–F}$ = 8.3 Hz), 131.0, 130.4, 129.9 (d, $J_{C–F}$ = 8.3 Hz), 128.8, 128.4, 126.5, 126.3, 115.5 (d, $J_{C–F}$ = 25.2 Hz), 115.4 (d, $J_{C–F}$ = 25.2 Hz), 105.4, 104.3, 103.6, 103.5, 52.6 (CH_3), 52.4 (CH_3), -0.1 ($Si(CH_3)_3$, minor isomer), -0.4 ($Si(CH_3)_3$ major isomer); ^{19}F NMR (471 MHz, $CDCl_3$, 298 K) δ: -112.38 (Ar–F, minor isomer), -112.54 (Ar–F, major isomer); IR ν_{max} (cm^{-1}): 3053, 2954, 1742 (C=O), 1720 (C=O), 1598, 1504, 1431, 1319, 1263, 1215, 1157, 1089, 1068, 1012; HRMS (ES+) $[M+H]^+$ $[C_{21}H_{21}O_2FSiCl]^+$: calculated 387.0983, found 387.0985.

*Synthesis of methyl 2,3-diphenyl-5-(trimethylsilyl)pent-2-en-4-ynoate (**3p**):*

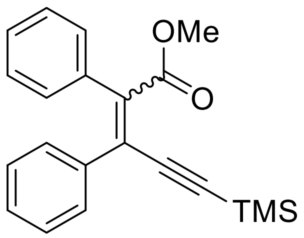

Synthetized in accordance with *General Procedure d* using $B(C_6F_5)_3$ (5 mg, 0.01 mmol), diazoester **1f** (18 mg, 0.10 mmol), and alkynyl aryl ester **2e** (36 mg, 0.11 mmol) in TFT to afford **3p**. The crude reaction mixture was purified *via* preparative thin layer chromatography using hexane/ethyl acetate (92:8 v/v) as eluent. The desired compound **3p** was obtained as a mixture of isomers (1:0.4) which appeared as a yellow liquid. Yield: 12 mg, 36%, 0.12 mmol.

^{1}H NMR (500 MHz, $CDCl_3$, 298 K) δ: 7.67–7.64 (m, Ar–H), 7.48–7.44 (m, Ar–H), 7.40–7.33 (m, Ar–H), 7.21–7.16 (m, Ar–H), 7.11–7.08 (m, Ar–H), 7.07–7.03 (m, Ar–H), 6.88–6.83 (m, Ar–H), 3.86 (s, $COOCH_3$, minor isomer), 3.57 (s, 3H, $COOCH_3$, major isomer), 0.24 (s, $Si(CH_3)_3$, minor isomer), 0.10 (s, 9H, $Si(CH_3)_3$, major isomer); ^{13}C NMR (126 MHz, $CDCl_3$, 298 K) δ: 169.5 (C=O), 168.9 (C=O), 163.8, 163.4, 161.8, 161.4, 141.0, 140.9, 135.9, 134.9, 134.6, 131.66, 131.60, 129.5, 129.4, 129.0, 128.9, 128.5, 128.4, 128.9, 125.56, 125.50, 115.5, 115.2, 115.1, 104.4, 103.8, 103.7, 103.3, 52.5 (CH_3, minor isomer), 52.3 (CH_3, major isomer), -0.1 ($Si(CH_3)_3$, minor isomer)$_3$), -0.4 ($Si(CH_3)_3$, major isomer); IR ν_{max} (cm^{-1}): 3055, 2956, 1759 (C=O), 1720 (C=O), 1600, 1504, 1433, 1263, 1217, 1157, 1070, 1035, 1018.

*Synthesis of methyl (E)-2-(4-chlorophenyl)-3-phenyl-5-(trimethylsilyl)pent-2-en-4-ynoate (**3q**):*

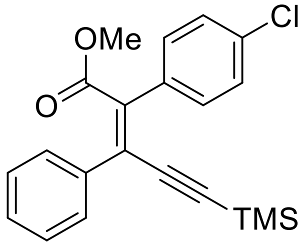

Synthetized in accordance with *General Procedure d* using $B(C_6F_5)_3$ (5 mg, 0.01 mmol), diazoester **1e** (21 mg, 0.10 mmol), and alkynyl aryl ester **2e** (36 mg, 0.11 mmol) in TFT to afford **3q** as major isomer. The crude reaction mixture was purified *via* preparative thin layer chromatography using hexane/ethyl acetate (92:8 v/v) as eluent. The desired compound **3q** (major isomer) was obtained as a yellow liquid. Yield: 11 mg, 30%, 0.30 mmol.

^{1}H NMR (500 MHz, $CDCl_3$, 298 K) δ: 7.64–7.61 (m, 2H, Ar–H), 7.47–7.44 (m, 2H, Ar–H), 7.39–7.33 (m, 5H, Ar–H), 3.53 (s, 3H, $COOCH_3$), 0.12 (s, 9H, $Si(CH_3)_3$); ^{13}C NMR (126 MHz, $CDCl_3$, 298 K) δ: 169.3, 139.5, 138.3, 134.6, 134.5, 130.5, 128.7, 128.5, 128.3, 128.0, 127.4, 105.1, 103.6, 52.3 (CH_3), -0.4 $Si(CH_3)_3$; IR ν_{max} (cm^{-1}): 2933, 2848, 1718 (C=O), 1656, 1492, 1444, 1433, 1323, 1263, 1249, 1213, 1091, 1068, 1029, 1012; HRMS (ES+) $[M+H]^+$ $[C_{21}H_{22}O_2SiCl]^+$: calculated 369.1078, found 369.1075.

*Synthesis of methyl (Z)-2-(4-chlorophenyl)-3-phenyl-5-(trimethylsilyl)pent-2-en-4-ynoate (**3q'**):*

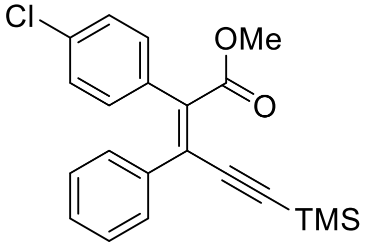

Synthetized in accordance with *General Procedure d* using $B(C_6F_5)_3$ (5 mg, 0.01 mmol), diazoester **1e** (21 mg, 0.10 mmol), and alkynyl aryl ester **2e** (36 mg, 0.11 mmol) in TFT to afford **3q'** as minor isomer. The crude reaction mixture was purified *via* preparative thin layer chromatography using hexane/ethyl acetate (92:8 v/v) as eluent. The desired compound **3q'** (minor isomer) was obtained as a yellow liquid. Yield: 5 mg, 13%, 0.13 mmol. Isomeric ratio: 1:0.4.

^{1}H NMR (500 MHz, $CDCl_3$, 298 K) δ: 7.22–7.18 (m, 5H, Ar–H), 7.16–7.13 (m, 2H, Ar–H), 7.03–7.00 (m, 2H, Ar–H), 3.86 (s, 3H, $COOCH_3$), 0.23 (s, 9H, $Si(CH_3)_3$); ^{13}C NMR (126 MHz, $CDCl_3$, 298 K) δ: 168.7 (C=O), 139.4, 136.5, 134.3, 133.6, 131.0, 129.7, 128.6, 128.4, 128.3, 127.5, 104.0, 103.8, 52.6 (CH_3), -0.1 ($Si(CH_3)_3$); IR ν_{max} (cm^{-1}): 2954, 2899, 1720 (C=O), 1489, 1433, 1263, 1249, 1209, 1091; HRMS (ES+) $[M+H]^+$ $[C_{21}H_{22}O_2SiCl]^+$: calculated 369.1078, found 369.1078.

*Synthesis of dimethyl (E)-2-(1,3-diphenylallylidene)malonate (**3r**):*

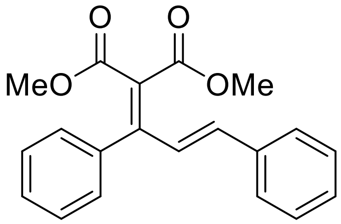

Synthesized in accordance with *General Procedure d* using $B(C_6F_5)_3$ (5 mg, 0.01 mmol), diazoester **1a** (16 mg, 0.10 mmol), and alkene ester **2j** (34 mg, 0.11 mmol) in TFT to afford **3r**. The crude reaction mixture was purified *via* preparative thin layer chromatography using hexane/diethyl ether (90:10 v/v) as eluent. The desired compound **3r** was obtained as a colorless oil. Yield: 23 mg, 71%, 0.07 mmol.

^1H NMR (500 MHz, $CDCl_3$, 298 K) δ: 8.14 (d, J = 15.9 Hz, 1H, CH), 7.44–7.39 (m, 5H, Ar–H), 7.33–7.29 (m, 4H, Ar–H), 7.25–7.24 (m, 1H, Ar–H), 6.45 (d, J = 15.9 Hz, 1H, CH), 3.87 (s, 3H, CH_3), 3.44 (s, 3H, CH_3); ^{13}C NMR (126 MHz, $CDCl_3$, 298 K) δ: 166.8 (C=O), 164.9 (C=O), 154.6, 142.8, 136.9, 136.2, 129.5, 128.9, 128.8, 128.6, 128.2, 127.8, 126.7, 124.0, 52.4 (CH_3), 52.1 (CH_3); IR ν_{max} (cm^{-1}): 3331, 2927, 1704 (C=O), 1604, 1558, 1485, 1394, 1342, 1263, 1232, 1184, 1101, HRMS (ES+) $[M+Na]^+$ $[C_{20}H_{18}O_4Na]^+$: calculated 345.1103, found 345.1107.

*Synthesis of diisopropyl (E)-2-(1,3-diphenylallylidene)malonate (**3s**):*

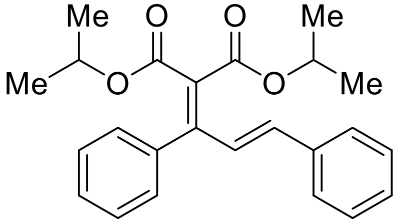

Synthesized in accordance with *General Procedure d* using $B(C_6F_5)_3$ (5 mg, 0.01 mmol), diazoester **1b** (21 mg, 0.10 mmol), and alkene ester **2j** (34 mg, 0.11 mmol) in TFT to afford **3s**. The crude reaction mixture was purified *via* preparative thin layer chromatography using hexane/diethyl ether (90:10 v/v) as eluent. The desired compound **3s** was obtained as a colorless oil. Yield: 29 mg, 77%, 0.08 mmol.

^1H NMR (500 MHz, $CDCl_3$, 298 K) δ: 8.15 (d, J = 16.0 Hz, 1H, CH), 7.42–7.37 (m, 5H, Ar–H), 7.33–7.26 (m, 5H, Ar–H), 6.40 (d, J = 16.0 Hz, 1H, CH), 5.20 (hept, J = 6.3 Hz, 1H, CH), 4.80 (hept, J = 6.3 Hz, 1H, CH), 1.34 (d, J = 6.3 Hz, 6H, CH_3), 0.98 (d, J = 6.3 Hz, 6H, CH_3); ^{13}C NMR (126 MHz, $CDCl_3$, 298 K) δ: 165.7 (C=O), 164.1 (C=O), 152.9, 141.8, 137.0, 136.4, 129.28, 129.24, 128.8, 128.4, 128.1, 127.7, 127.0, 125.7, 69.0 (CH), 68.5 (CH), 21.9 (CH_3), 21.3 (CH_3); IR ν_{max} (cm^{-1}): 2981, 1703 (C=O), 1610, 1573, 1467, 1386, 1323, 1228, 1105, 1051; HRMS (ES+) $[M+Na]^+$ $[C_{24}H_{26}O_4Na]^+$: calculated 401.1729, found 401.1730.

*Synthesis of 1-(4-chlorophenyl)-2-methoxy-2-oxoethyl 4-fluorobenzoate (**4a**):*[9]

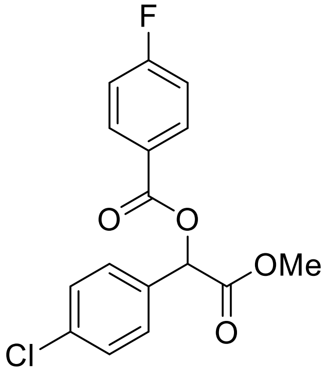

^{1}H NMR (500 MHz, $CDCl_3$, 298 K) δ: 8.15–8.11 (m, 2H, Ar–H), 7.52–7.49 (m, 2H, Ar–H), 7.42–7.39 (m, 2H, Ar–H), 7.16–7.11 (m, 2H, Ar–H), 6.12 (s, 1H, CH), 3.76 (s, 3H, $COOCH_3$); ^{13}C NMR (126 MHz, $CDCl_3$, 298 K) δ: 169.0 (C=O), 166.3 (d, $J_{C–F}$ = 255.1 Hz), 164.9 (C=O), 135.6, 132.7 (d, $J_{C–F}$ = 9.5 Hz), 132.5, 129.3, 129.1, 125.4 (d, $J_{C–F}$ = 3.0 Hz), 115.9 (d, $J_{C–F}$ = 22.1 Hz), 74.3 (CH), 53.0 (CH_3); ^{19}F NMR (471 MHz, $CDCl_3$, 298 K) δ: -104.28 (Ar–F).

*Synthesis of 2-methoxy-2-oxo-1-phenylethyl 4-fluorobenzoate (**4b**):*[9]

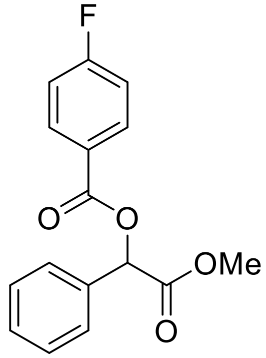

^{1}H NMR (500 MHz, $CDCl_3$, 298 K) δ: 8.17–8.13 (m, 2H, Ar–H), 7.59–7.55 (m, 2H, Ar–H), 7.46–7.40 (m, 3H, Ar–H), 7.16–7.11 (m, 2H, Ar–H), 6.15 (s, 1H, CH), 3.76 (s, 3H, $COOCH_3$); ^{13}C NMR (126 MHz, $CDCl_3$, 298 K) δ: 169.4 (C=O), 166.3 (d, $J_{C–F}$ = 254.8 Hz), 165.0 (C=O), 134.0, 132.7 (d, $J_{C–F}$ = 9.5 Hz), 129.5, 129.0, 127.8, 125.6 (d, $J_{C–F}$ = 3.0 Hz), 115.8 (d, $J_{C–F}$ = 22.1 Hz), 75.1 (CH), 52.9 (CH_3); ^{19}F NMR (471 MHz, $CDCl_3$, 298 K) δ: -104.58 (Ar–F).

*Synthesis of dimethyl 2-(bis(4-fluorophenyl)methylene)malonate (**6a**):*[10]

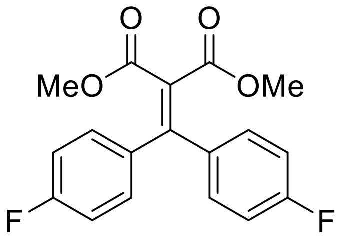

Synthesized in accordance with *General Procedure d* using $B(C_6F_5)_3$ (10 mg, 0.02 mmol), diazoester **1a** (16 mg, 0.10 mmol), and diaryl ester **2k** (35 mg, 0.11 mmol) in TFT to afford **6a**. The crude reaction mixture was purified *via* preparative thin layer chromatography using hexane/diethyl ether (87:13 v/v) as eluent. The desired compound **6a** was obtained as a pale-yellow solid. Yield: 26 mg, 78%, 0.08 mmol.

^{1}H NMR (500 MHz, $CDCl_3$, 298 K) δ: 7.17–7.13 (m, 4H, Ar–H), 7.05–7.01 (m, 4H, Ar–H), 3.63 (s, 6H, $COOCH_3$); ^{13}C NMR (126 MHz, $CDCl_3$, 298 K) δ: 166.3 (C=O), 163.6 (d, $J_{C–F}$ = 250.5 Hz), 154.4, 135.8 (d, $J_{C–F}$ = 3.5 Hz), 131.3 (d, $J_{C–F}$ = 8.5 Hz), 125.7, 115.6 (d, $J_{C–F}$ = 21.9 Hz), 52.5 (CH_3); ^{19}F NMR (471 MHz, $CDCl_3$, 298 K) δ: -110.81 (Ar–F).

*Synthesis of dibenzyl 2-(bis(4-fluorophenyl)methylene)malonate (**6b**):*

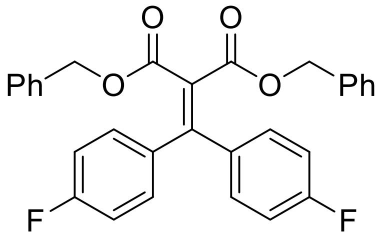

Synthesized in accordance with *General Procedure d* using $B(C_6F_5)_3$ (10 mg, 0.02 mmol), diazoester **1c** (31 mg, 0.10 mmol), and diaryl ester **2k** (35 mg, 0.11 mmol) in TFT to afford **6b**. The crude reaction mixture was purified *via* preparative thin layer chromatography using hexane/diethyl ether (85:15 v/v) as eluent. The desired compound **6b** was obtained as a colorless oil. Yield: 39 mg, 81%, 0.08 mmol.

^{1}H NMR (500 MHz, $CDCl_3$, 298 K) δ: 7.34–7.27 (m, 6H, Ar–H), 7.13–7.11 (m, 4H, Ar–H), 7.07–7.03 (m, 4H, Ar–H), 6.90–6.85 (m, 4H, Ar–H), 5.07 (s, 4H, CH_2); ^{13}C NMR (126 MHz, $CDCl_3$, 298 K) δ: 165.6 (C=O), 163.6 (d, $J_{C–F}$ = 250.5 Hz), 154.5, 135.7 (d, $J_{C–F}$ = 3.3 Hz), 135.1, 131.3 (d, $J_{C–F}$ = 8.5 Hz), 128.7, 128.6, 128.5, 115.5 (d, $J_{C–F}$ = 21.8 Hz), 67.3 (CH_2); ^{19}F NMR (471 MHz, $CDCl_3$, 298 K) δ: -110.84 (Ar–F); IR ν_{max} (cm^{-1}): 3066, 3035, 1718 (C=O), 1600, 1504, 1454, 1408, 1375, 1319, 1259, 1220, 1157, 1066. HRMS (ES+) $[M+Na]^+$ $[C_{30}H_{22}F_2\ O_4Na]^+$: calculated 507.1384, found 507.1385.

*Synthesis of diisopropyl 2-(bis(4-fluorophenyl)methylene) malonate (**6c**):*

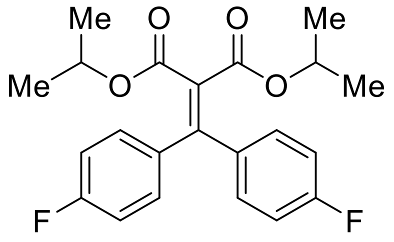

Synthesized in accordance with *General Procedure d* using $B(C_6F_5)_3$ (10 mg, 0.02 mmol), diazoester **1b** (22 mg, 0.10 mmol), and diaryl ester **2k** (35 mg, 0.11 mmol) in TFT to afford **6c**. The crude reaction mixture was purified *via* preparative thin layer chromatography using hexane/diethyl ether (87:13 v/v) as eluent. The desired compound **6c** was obtained as a while solid. Yield: 34 mg, 87%, 0.09 mmol.

^{1}H NMR (500 MHz, $CDCl_3$, 298 K) δ: 7.19–7.15 (m, 4H, Ar–H), 7.04–6.99 (m, 4H, Ar–H), 4.97 (hept, J = 6.3 Hz, 2H, CH), 1.12 (d, J = 6.3 Hz, 12H, CH_3); ^{13}C NMR (126 MHz, $CDCl_3$, 298 K) δ: 165.3 (C=O), 163.4 (d, $J_{C–F}$ = 249.9 Hz), 152.5, 136.0 (d, $J_{C–F}$ = 3.3 Hz), 131.3 (d, $J_{C–F}$ = 8.4 Hz), 127.7, 115.5 (d, $J_{C–F}$ = 21.8 Hz), 69.2 (CH), 21.5 (CH_3); ^{19}F NMR (471 MHz, $CDCl_3$, 298 K) δ: -111.51 (Ar–F); IR ν_{max} (cm^{-1}): 2983, 2937, 1720 (C=O), 1600, 1506, 1373, 1315, 1226, 1161, 1105, 1070; HRMS (ES+) $[M+Na]^+$ $[C_{22}H_{22}F_2O_4Na]^+$: calculated 411.1384, found 411.1385.

*Synthesis of dimethyl 2-(diphenylmethylene) malonate (**6d**):*[10]

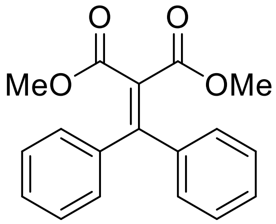

Synthesized in accordance with *General Procedure d* using $B(C_6F_5)_3$ (10 mg, 0.02 mmol), diazoester **1a** (16 mg, 0.10 mmol), and diaryl ester **2l** (31 mg, 0.11 mmol) in TFT to afford **6d**. The crude reaction mixture was purified *via* preparative thin layer chromatography using hexane/diethyl ether (87:13 v/v) as eluent. The desired compound **6d** was obtained as a pale-yellow oil. Yield: 21 mg, 73%, 0.07 mmol.

^{1}H NMR (500 MHz, $CDCl_3$, 298 K) δ: 7.38–7.31 (m, 6H, Ar–H), 7.19–7.17 (m, 4H, Ar–H), 3.61 (s, 6H, $COOCH_3$); ^{13}C NMR (126 MHz, $CDCl_3$, 298 K) δ: 166.5 (C=O), 156.7, 140.0, 129.5, 129.2, 128.3, 125.6, 52.4 (CH_3).

*Synthesis of diisopropyl 2-(diphenylmethylene) malonate (**6e**):* [10]

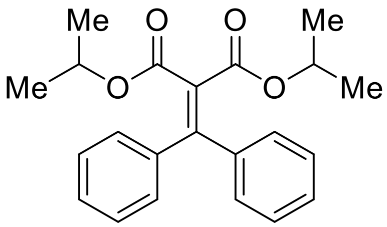

Synthesized in accordance with *General Procedure d* using $B(C_6F_5)_3$ (10 mg, 0.02 mmol), diazoester **1b** (22 mg, 0.10 mmol), and diaryl ester **2l** (31 mg, 0.11 mmol) in TFT to afford **6e**. The crude reaction mixture was purified *via* preparative thin layer chromatography using hexane/diethyl ether (87:13 v/v) as eluent. The desired compound **6e** was obtained as a white solid. Yield: 27 mg, 76%, 0.07 mmol.

^{1}H NMR (500 MHz, $CDCl_3$, 298 K) δ: 7.36–7.29 (m, 6H, Ar–H), 7.21–7.19 (m, 4H, Ar–H), 4.94 (hept, J = 6.3 Hz, 2H, CH), 1.08 (d, J = 6.3 Hz, 12H, CH_3); ^{13}C NMR (126 MHz, $CDCl_3$, 298 K) δ: 165.6 (C=O), 154.8, 140.3, 129.2, 129.1, 128.2, 127.4, 69.0 (CH), 21.4 (CH_3).

*Synthesis of dimethyl 2-(bis(4-methoxyphenyl)methylene) malonate (**6f**):*[10]

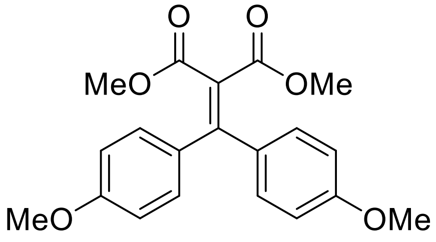

Synthesized in accordance with *General Procedure d* using $B(C_6F_5)_3$ (10 mg, 0.02 mmol), diazoester **1a** (16 mg, 0.10 mmol), and diaryl ester **2m** (37 mg, 0.11 mmol) in TFT to afford **6f**. The crude reaction mixture was purified *via* preparative thin layer chromatography using hexane/diethyl ether (70:30 v/v) as eluent. The desired compound **6f** was obtained as a white solid. Yield: 25 mg, 70%, 0.07 mmol.

^{1}H NMR (500 MHz, $CDCl_3$, 298 K) δ: 7.12–7.09 (m, 4H, Ar–H), 6.86–6.83 (m, 4H, Ar–H), 3.82 (s, 6H, $COOCH_3$), 3.62 (s, 6H, OCH_3); ^{13}C NMR (126 MHz, $CDCl_3$, 298 K) δ: 167.2 (C=O), 160.9 (C=O), 156.8, 132.6, 131.3, 123.0, 113.7, 55.4 (CH_3), 52.3 (CH_3).

*Synthesis of diisopropyl 2-(bis(4-methoxyphenyl)methylene) malonate (**6g**):*

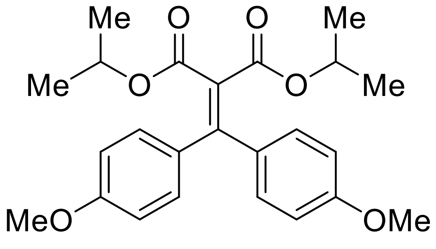

Synthesized in accordance with *General Procedure d* using $B(C_6F_5)_3$ (10 mg, 0.02 mmol), diazoester **1b** (22 mg, 0.10 mmol), and diaryl ester **2m** (37 mg, 0.11 mmol) in TFT to afford **6g**. The crude reaction mixture was purified *via* preparative thin layer chromatography using hexane/diethyl ether (75:25 v/v) as eluent. The desired compound **6g** was obtained as a white solid. Yield: 32 mg, 78%, 0.08 mmol.

^{1}H NMR (500 MHz, $CDCl_3$, 298 K) δ: 7.13–7.11 (m, 4H, Ar–H), 6.84–6.81 (m, 4H, Ar–H), 4.97 (hept, J = 6.3 Hz, 2H, CH), 3.81 (s, 6H, OCH_3), 1.13 (d, J = 6.3 Hz, 12H, CH_3); ^{13}C NMR (126 MHz, $CDCl_3$, 298 K) δ: 166.2 (C=O), 160.6 (C=O), 154.9, 132.9, 131.3, 125.1, 113.6, 68.7(CH), 55.4 (CH_3), 21.6 (CH_3); IR ν_{max} (cm^{-1}): 2981, 2935, 1705 (C=O), 1602, 1508, 1463, 1246, 1166, 1107, 1074, 1031; HRMS (ES+) $[M+Na]^+$ $[C_{24}H_{28}O_6Na]^+$: calculated 435. 1784, found 435.1785.

*Synthesis of dimethyl 2-((4-chlorophenyl)(phenyl)methylene) malonate (**6h**):*[10]

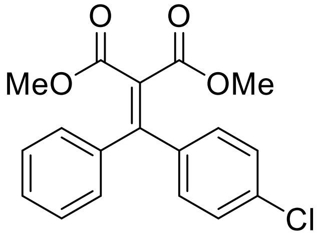

Synthesized in accordance with *General Procedure d* using $B(C_6F_5)_3$ (10 mg, 0.02 mmol), diazoester **1a** (16 mg, 0.10 mmol), and diaryl ester **2n** (34 mg, 0.11 mmol) in TFT to afford **6h**. The crude reaction mixture was purified *via* preparative thin layer chromatography using hexane/diethyl ether (85:15 v/v) as eluent. The desired compound **6h** was obtained as a colorless oil. Yield: 25 mg, 75%, 0.07 mmol.

^{1}H NMR (500 MHz, $CDCl_3$, 298 K) δ: 7.40–7.29 (m, 5H, Ar–H), 7.17–7.15 (m, 2H, Ar–H), 7.13–7.10 (m, 2H, Ar–H), 3.64 (s, 3H, $COOCH_3$), 3.60 (s, 3H, $COOCH_3$); ^{13}C NMR (126 MHz, $CDCl_3$, 298 K) δ: 166.3 (C=O), 166.2 (C=O), 155.3, 139.6, 138.4, 135.7, 130.6, 129.7, 129.2, 128.7, 128.5, 125.9, 52.5 (CH_3), 52.4 (CH_3).

*Synthesis of diisopropyl 2-((4-chlorophenyl)(phenyl)methylene) malonate (**6i**):*

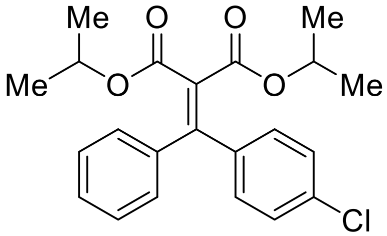

Synthesized in accordance with *General Procedure d* using $B(C_6F_5)_3$ (10 mg, 0.02 mmol), diazoester **1b** (22 mg, 0.10 mmol), and diaryl ester **2n** (34 mg, 0.11 mmol) in TFT to afford **6i**. The crude reaction mixture was purified *via* preparative thin layer chromatography using hexane/diethyl ether (87:13 v/v) as eluent. The desired compound **6i** was obtained as a white solid. Yield: 32 mg, 84%, 0.08 mmol.

^{1}H NMR (500 MHz, $CDCl_3$, 298 K) δ: 7.37–7.28 (m, 5H, Ar–H), 7.19–7.16 (m, 2H, Ar–H), 7.15–7.12 (m, 2H, Ar–H), 5.01–4.90 (m, 2H, CH), 1.12 (d, J = 6.3 Hz, 6H, CH_3), 1.08 (d, J = 6.3 Hz, 6H, CH_3); ^{13}C NMR (126 MHz, $CDCl_3$, 298 K) δ: 165.4 (C=O), 165.2 (C=O), 153.5, 139.8, 138.6, 135.3, 130.6, 129.4, 129.2, 128.5, 128.4, 127.8, 69.2 (CH), 69.1 (CH), 21.5 (CH_3), 21.4 (CH_3); IR ν_{max} (cm^{-1}): 2980, 2935, 1718 (C=O), 1598, 1506, 1487, 1448, 1371, 1355, 1315, 1236, 1166, 1105, 1070, 1014; HRMS (ES+) $[M+Na]^+$ $[C_{22}H_{23}ClO_4Na]^+$: calculated 409.1183, found 409.1184.

*Synthesis of dibenzyl 2-((4-chlorophenyl)(phenyl)methylene)malonate (**6j**):*

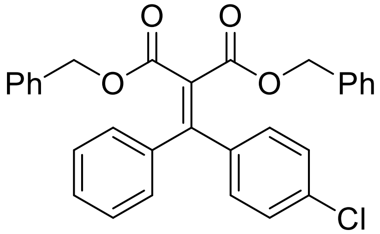

Synthesized in accordance with *General Procedure d* using $B(C_6F_5)_3$ (10 mg, 0.02 mmol), diazoester **1c** (31 mg, 0.10 mmol), and diaryl ester **2n** (34 mg, 0.11 mmol) in TFT to afford **6j**. The crude reaction mixture was purified *via* preparative thin layer chromatography using hexane/diethyl ether (87:13 v/v) as eluent. The desired compound **6j** was obtained as a colorless oil. Yield: 36 mg, 75%, 0.07 mmol.

^{1}H NMR (500 MHz, $CDCl_3$, 298 K) δ: 7.30–7.18 (m, 9H, Ar–H), 7.12–7.09 (m, 2H, Ar–H), 7.07–7.04 (m, 4H, Ar–H), 7.02–7.00 (m, 2H, Ar–H), 6.98–6.96 (m, 2H, Ar–H), 5.02 (s, 2H, CH_2), 4.99 (s, 2H, CH_2); ^{13}C NMR (126 MHz, $CDCl_3$, 298 K) δ: 165.7 (C=O), 165.6 (C=O), 155.4, 139.6, 138.4, 135.6, 135.1, 135.0, 130.6, 129.7, 129.3, 128.7, 128.59, 128.57, 128.53, 128.50, 128.4, 128.3, 67.35 (CH_2), 67.34 (CH_2); IR ν_{max} (cm^{-1}): 3034, 1718 (C=O), 1591, 1489, 1452, 1375, 1323, 1261, 1224, 1161, 1066, 1014; HRMS (ES+) $[M+Na]^+$ $[C_{30}H_{23}ClO_4Na]^+$: calculated 505.1183, found 505.1183.

*Synthesis of dimethyl 2-(phenyl(p-tolyl)methylene) malonate (**6k**):*[10]

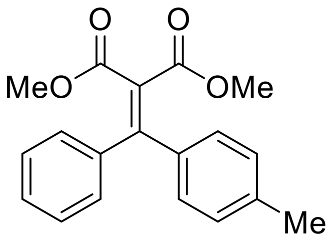

Synthesized in accordance with *General Procedure d* using $B(C_6F_5)_3$ (10 mg, 0.02 mmol), diazoester **1a** (16 mg, 0.10 mmol), and diaryl ester **2o** (32 mg, 0.11 mmol) in TFT to afford **6k**. The crude reaction mixture was purified *via* preparative thin layer chromatography using hexane/diethyl ether (87:13 v/v) as eluent. The desired compound **6k** was obtained as a colorless liquid. Yield: 22 mg, 72%, 0.07 mmol.

^{1}H NMR (500 MHz, $CDCl_3$, 298 K) δ: 7.38–7.31 (m, 3H, Ar–H), 7.19–7.16 (m, 2H, Ar–H), 7.14–7.12 (m, 2H, Ar–H), 7.07–7.05 (m, 2H, Ar–H), 3.64 (s, 3H, $COOCH_3$), 3.59 (s, 3H, $COOCH_3$), 2.36 (s, 3H, CH_3); ^{13}C NMR (126 MHz, $CDCl_3$, 298 K) δ: 166.7 (C=O), 166.6 (C=O), 140.3, 139.8, 137.1, 129.4, 129.3, 129.2, 129.1, 128.3, 125.0, 52.4 (CH_3), 52.3 (CH_3), 21.5 (CH_3).

*Synthesis of dibenzyl 2-(phenyl(p-tolyl)methylene)malonate (**6l**):*

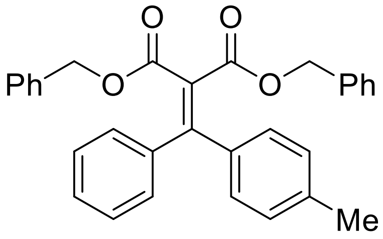

Synthesized in accordance with *General Procedure d* using $B(C_6F_5)_3$ (10 mg, 0.02 mmol), diazoester **1c** (31 mg, 0.10 mmol), and diaryl ester **2o** (32 mg, 0.11 mmol) in TFT to afford **6l**. The crude reaction mixture was purified *via* preparative thin layer chromatography using hexane/diethyl ether (87:13 v/v) as eluent. The desired compound **6l** was obtained as a colorless oil. Yield: 31 mg, 67%, 0.07 mmol.

^{1}H NMR (500 MHz, $CDCl_3$, 298 K) δ: 7.29–7.18 (m, 9H, Ar–H), 7.10–7.08 (m, 2H, Ar–H), 7.03–6.95 (m, 8H, Ar–H), 5.02 (s, 2H, CH_2), 4.98 (s, 2H, CH_2), 2.28 (s, 3H, CH_3); ^{13}C NMR (126 MHz, $CDCl_3$, 298 K) δ: 166.1 (C=O), 166.0 (C=O), 157.1, 140.3, 139.7, 137.2, 135.3, 135.2, 129.4, 129.0, 128.5, 128.47, 128.46, 128.43, 128.3, 128.24, 128.23, 125.1, 67.16 (CH_2), 67.14 (CH_2), 21.5 (CH_3); IR ν_{max} (cm^{-1}): 2976, 2872, 1718 (C=O), 1450, 1379, 1325, 1303, 1259, 1224, 1159, 1109, 1070; HRMS (ES+) $[M+Na]^+$ $[C_{31}H_{26}O_4Na]^+$: calculated 485.1729, found 485.1729.

3. NMR Spectra

Figure S1: ^{1}**H NMR** (400 MHz, $CDCl_3$, 298 K) spectrum of compound **1a**

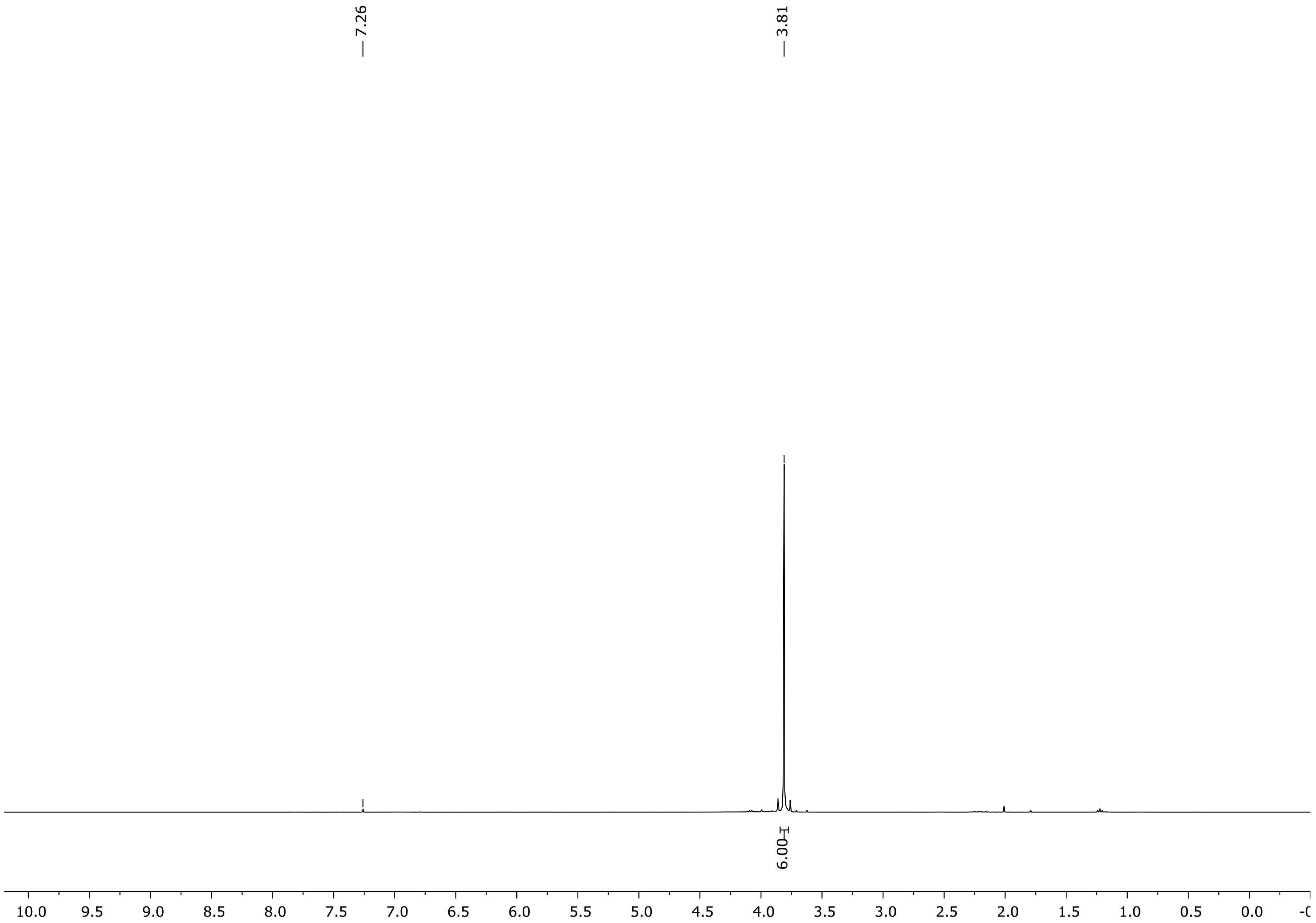

Figure S2: ^{13}**C NMR** (101 MHz, $CDCl_3$, 298 K) spectrum of compound **1a**

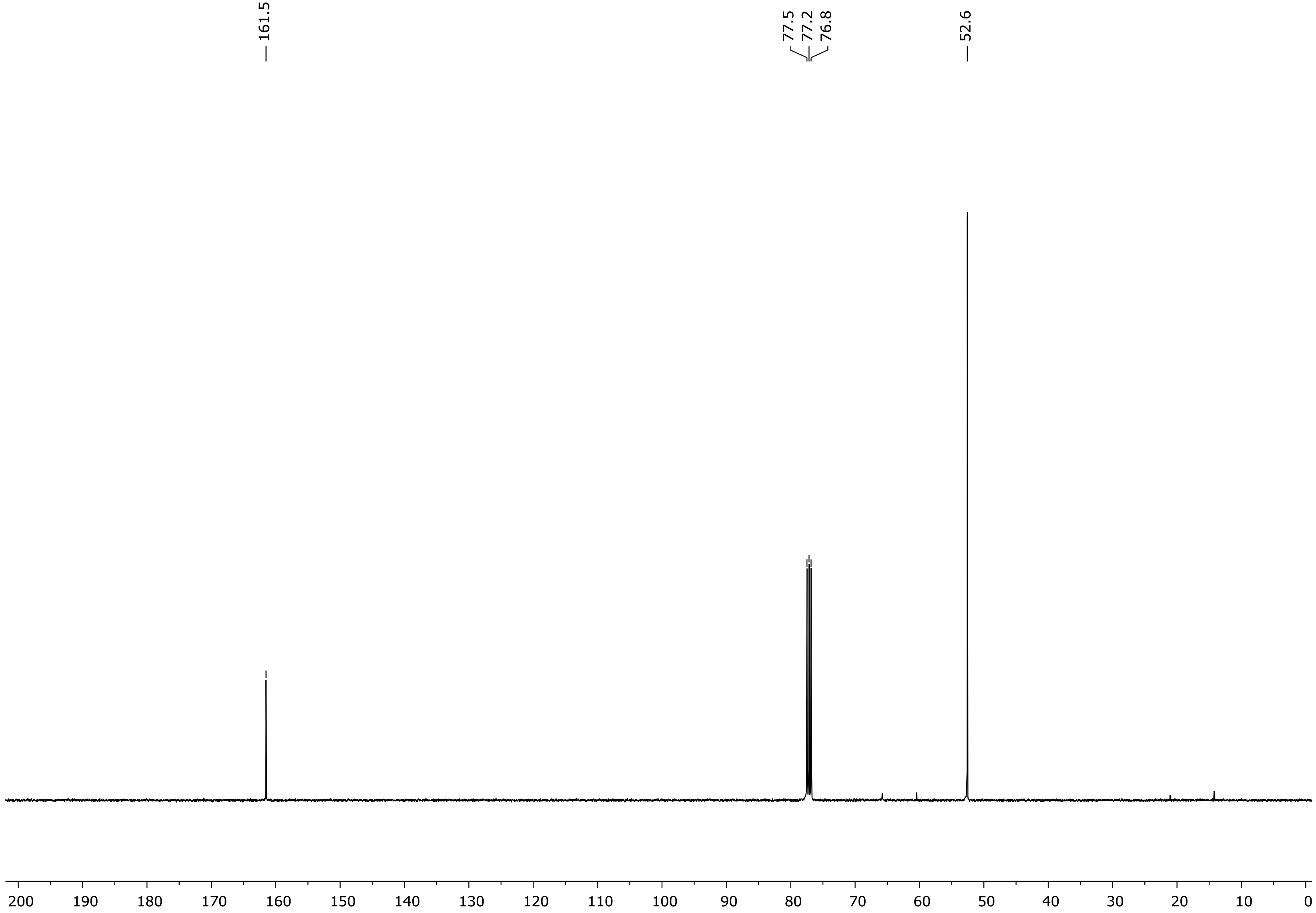

Figure S3: ^{1}**H NMR** (400 MHz, $CDCl_3$, 298 K) spectrum of compound **1b**

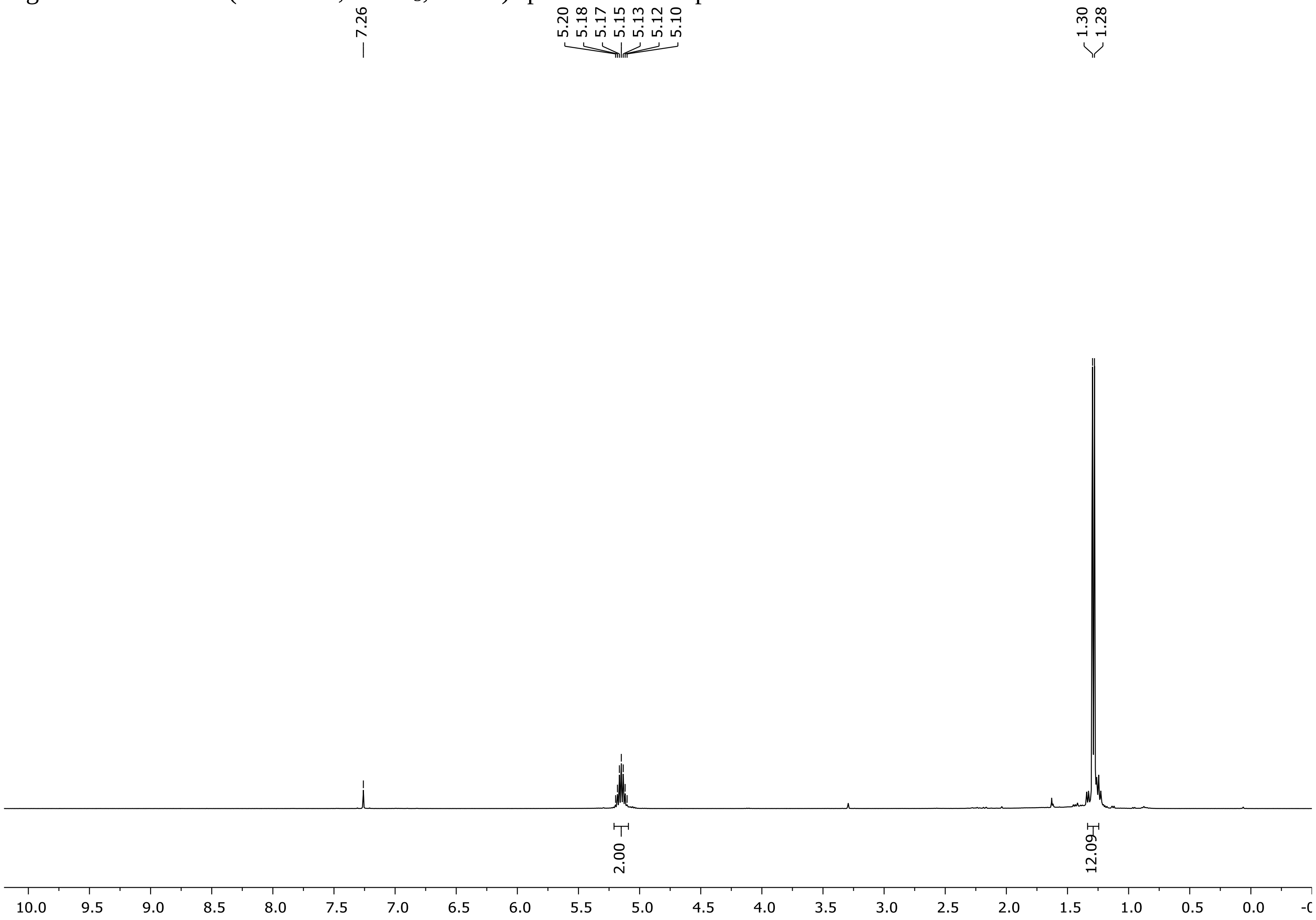

Figure S4: **^{13}C NMR** (101 MHz, $CDCl_3$, 298 K) spectrum of compound **1b**

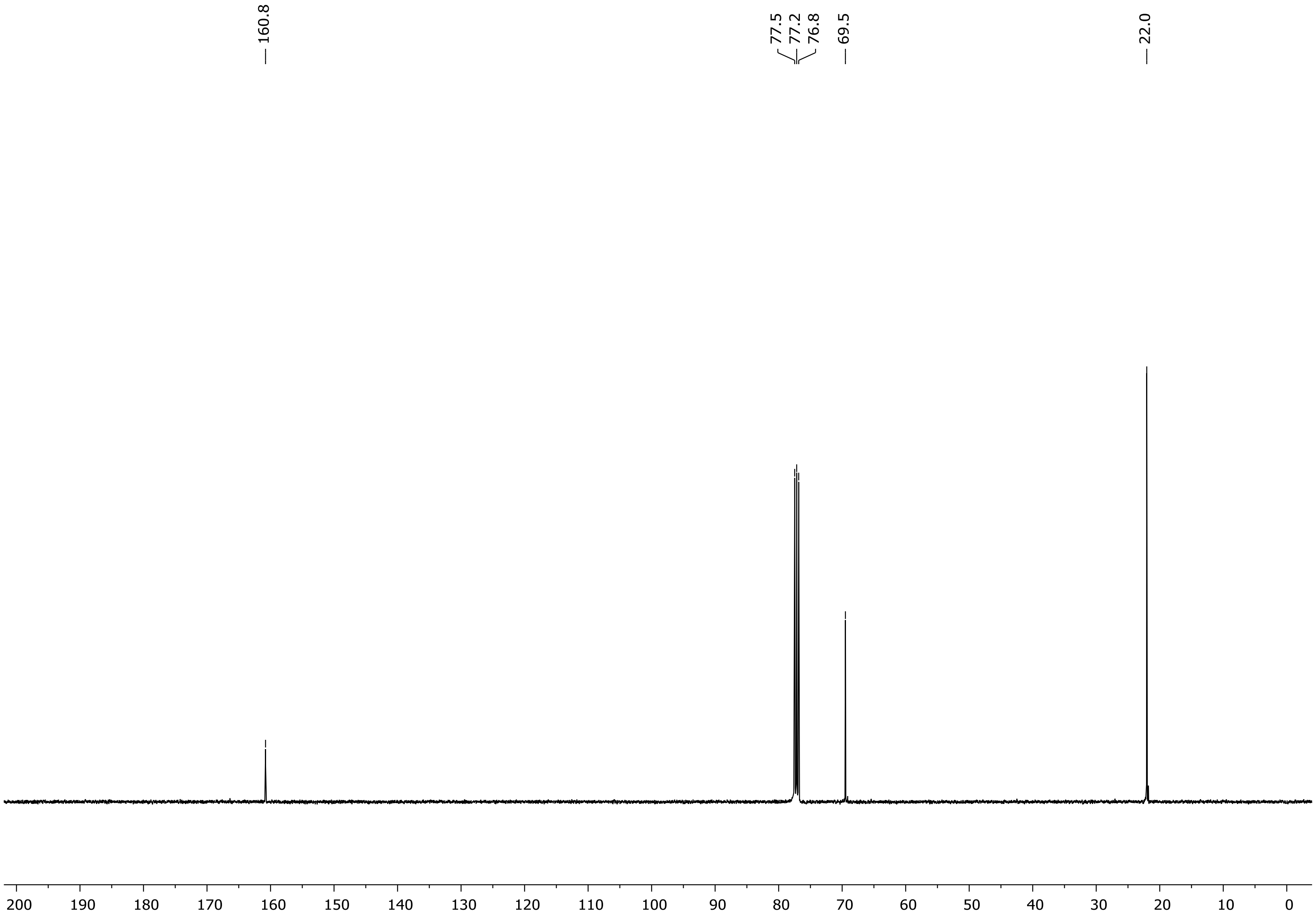

Figure S5: **^{1}H NMR** (400 MHz, $CDCl_3$, 298 K) spectrum of compound **1c**

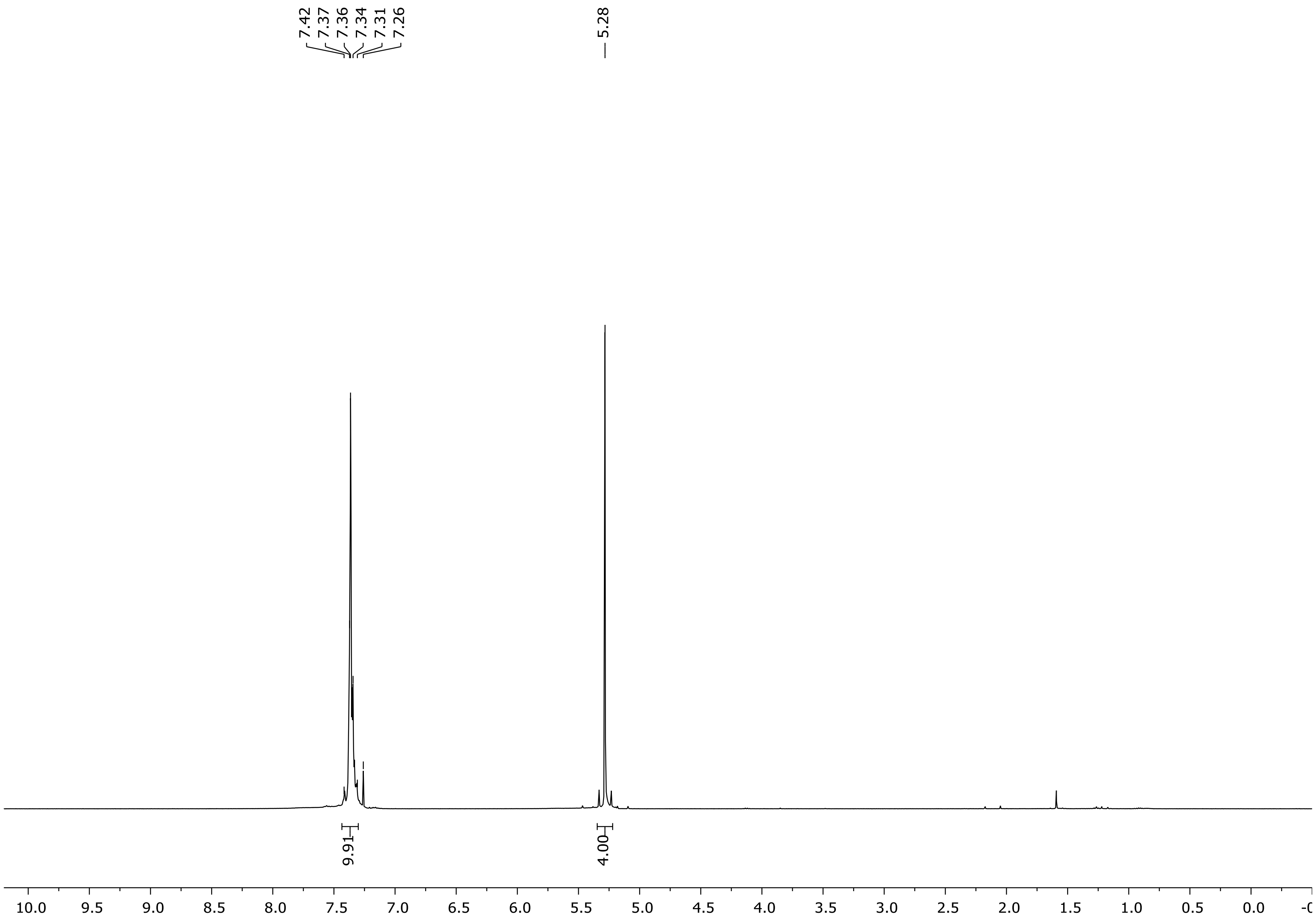

Figure S6: **^{13}C NMR** (101 MHz, $CDCl_3$, 298 K) spectrum of compound **1c**

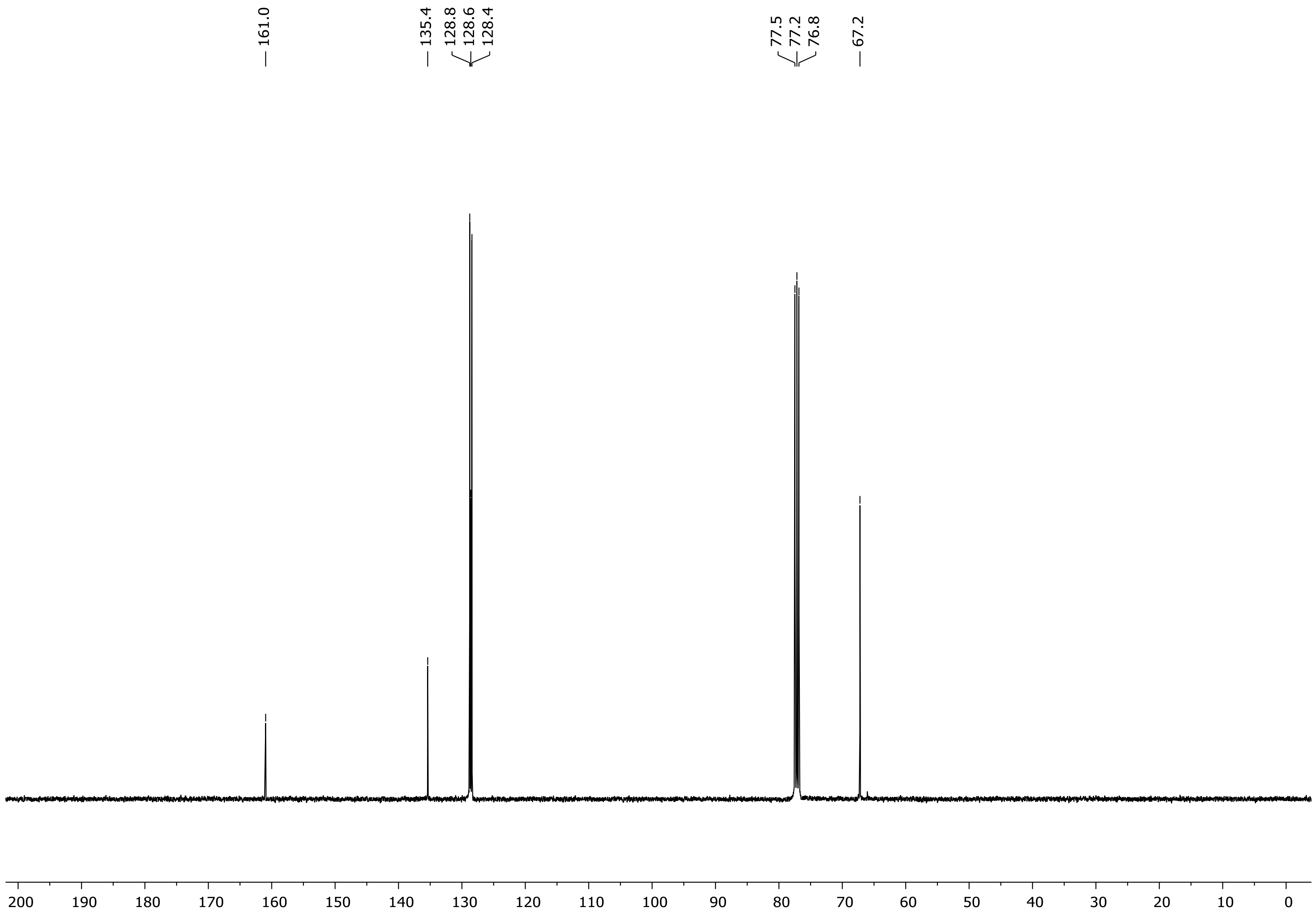

Figure S7: ^{1}**H NMR** (500 MHz, $CDCl_3$, 298 K) spectrum of compound **1d**

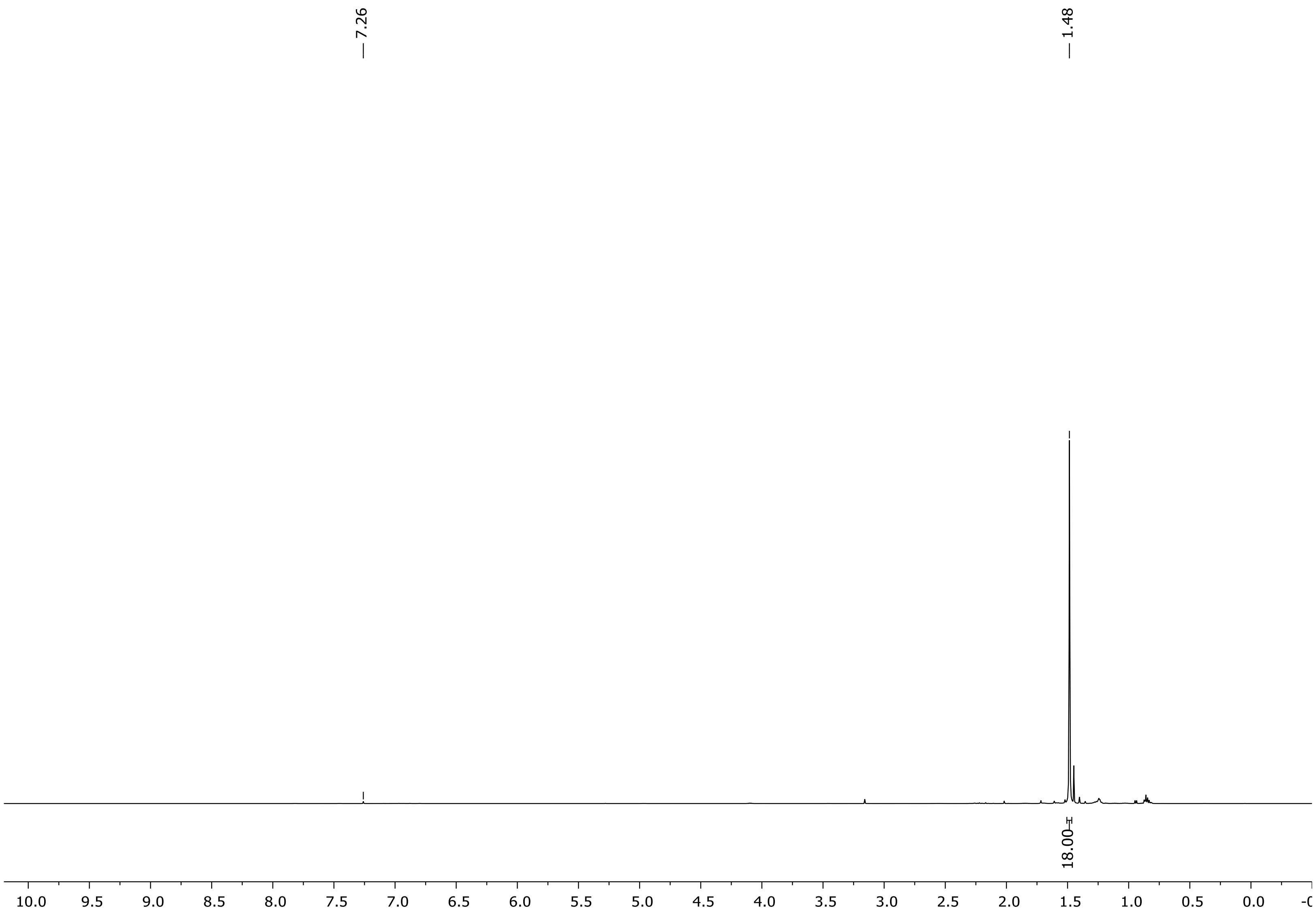

Figure S8: ^{13}**C NMR** (101 MHz, $CDCl_3$, 298 K) spectrum of compound **1d**

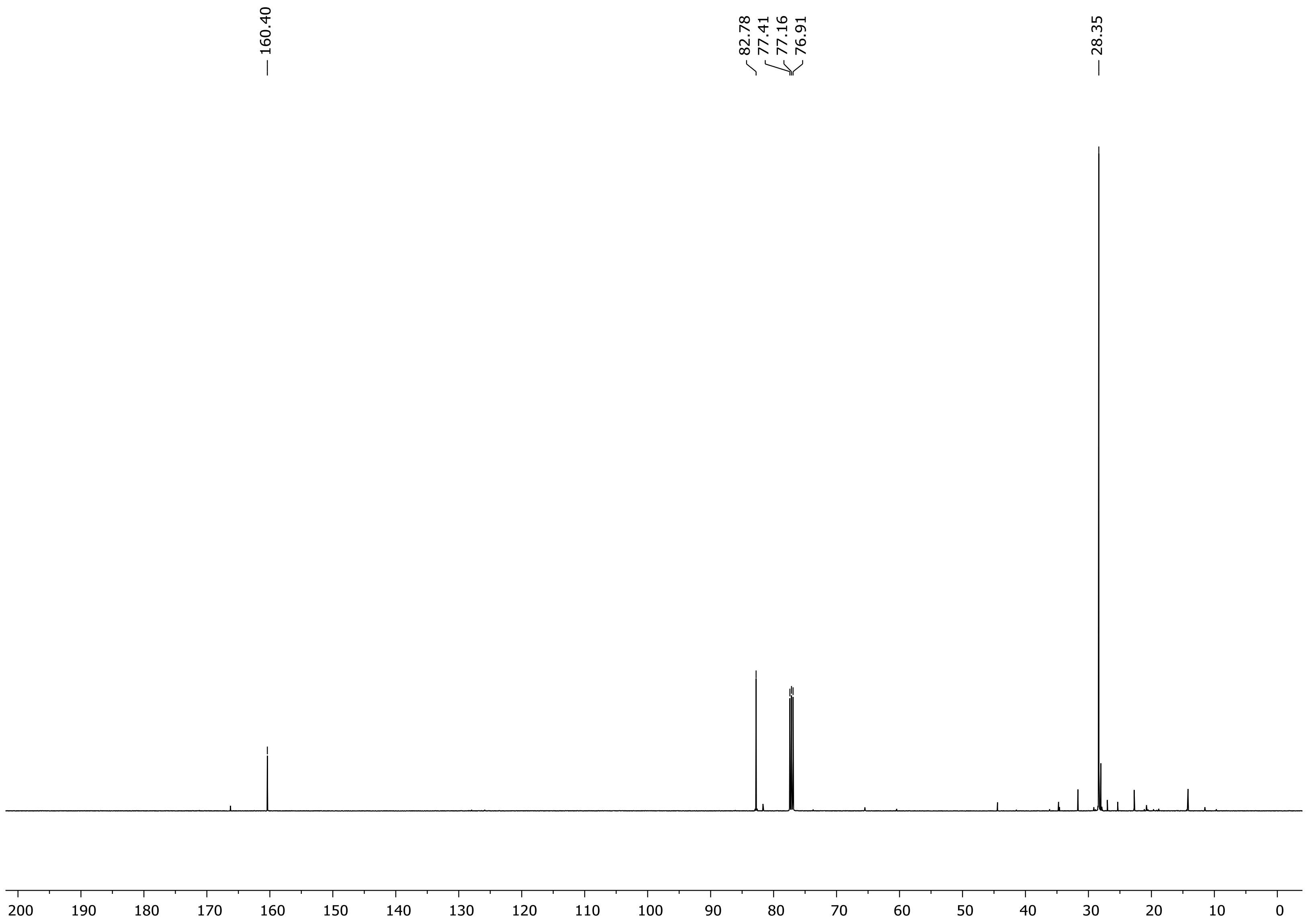

Figure S9: **^{1}H NMR** (400 MHz, $CDCl_3$, 298 K) spectrum of compound **1e**

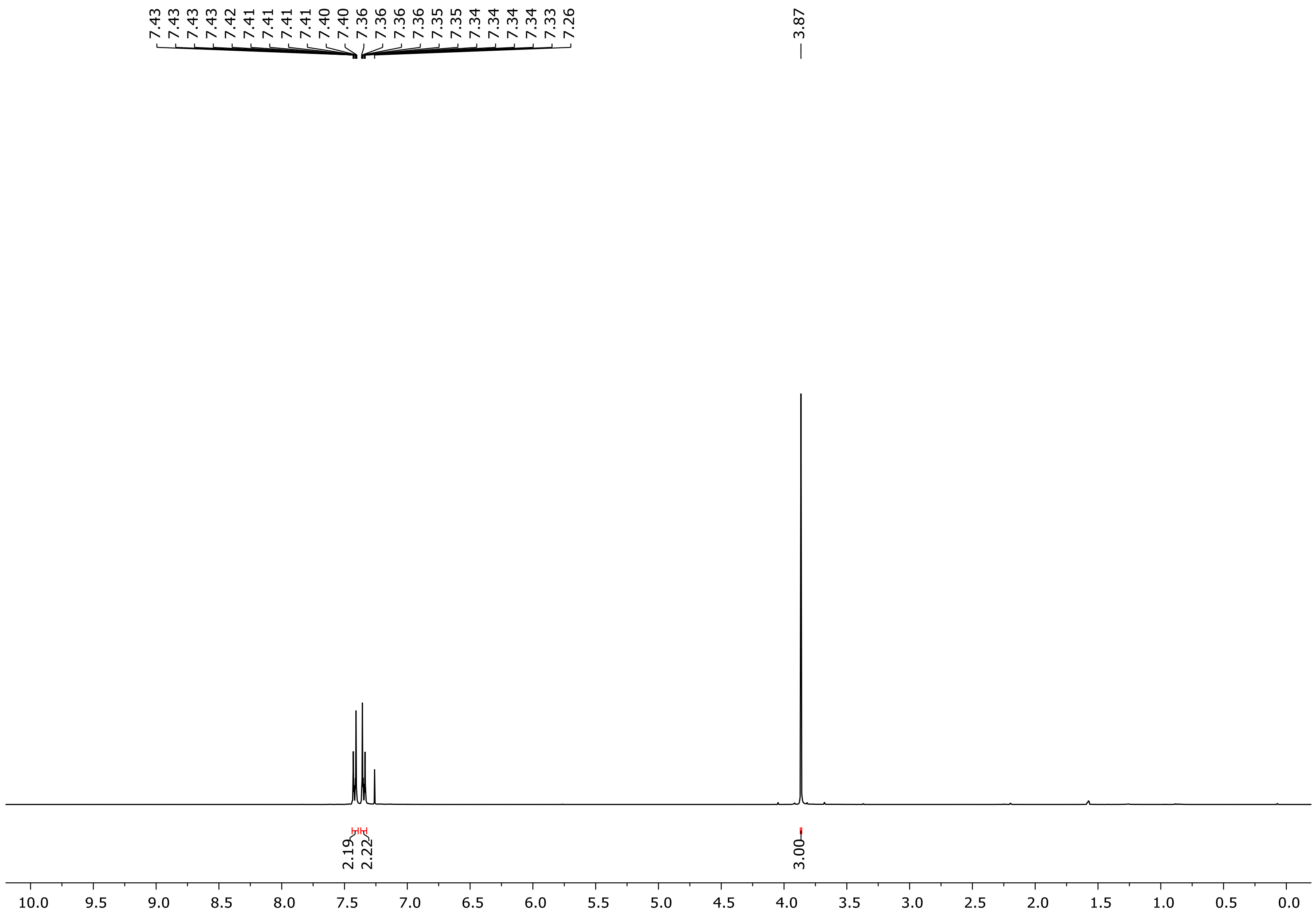

Figure S10: **^{13}C NMR** (101 MHz, $CDCl_3$, 298 K) spectrum of compound **1e**

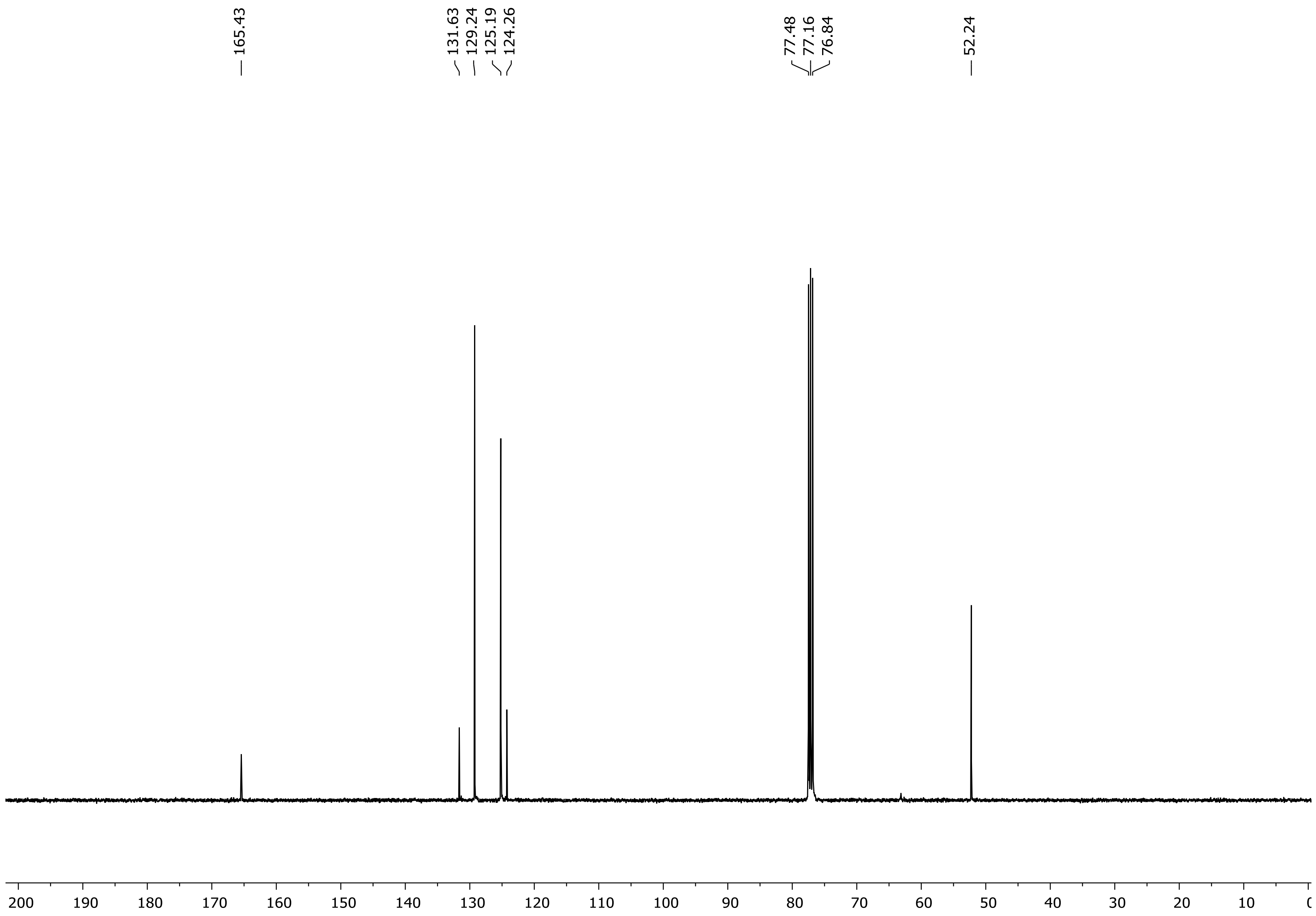

Figure S11: **^{1}H NMR** (500 MHz, $CDCl_3$, 298 K) spectrum of compound **1f**

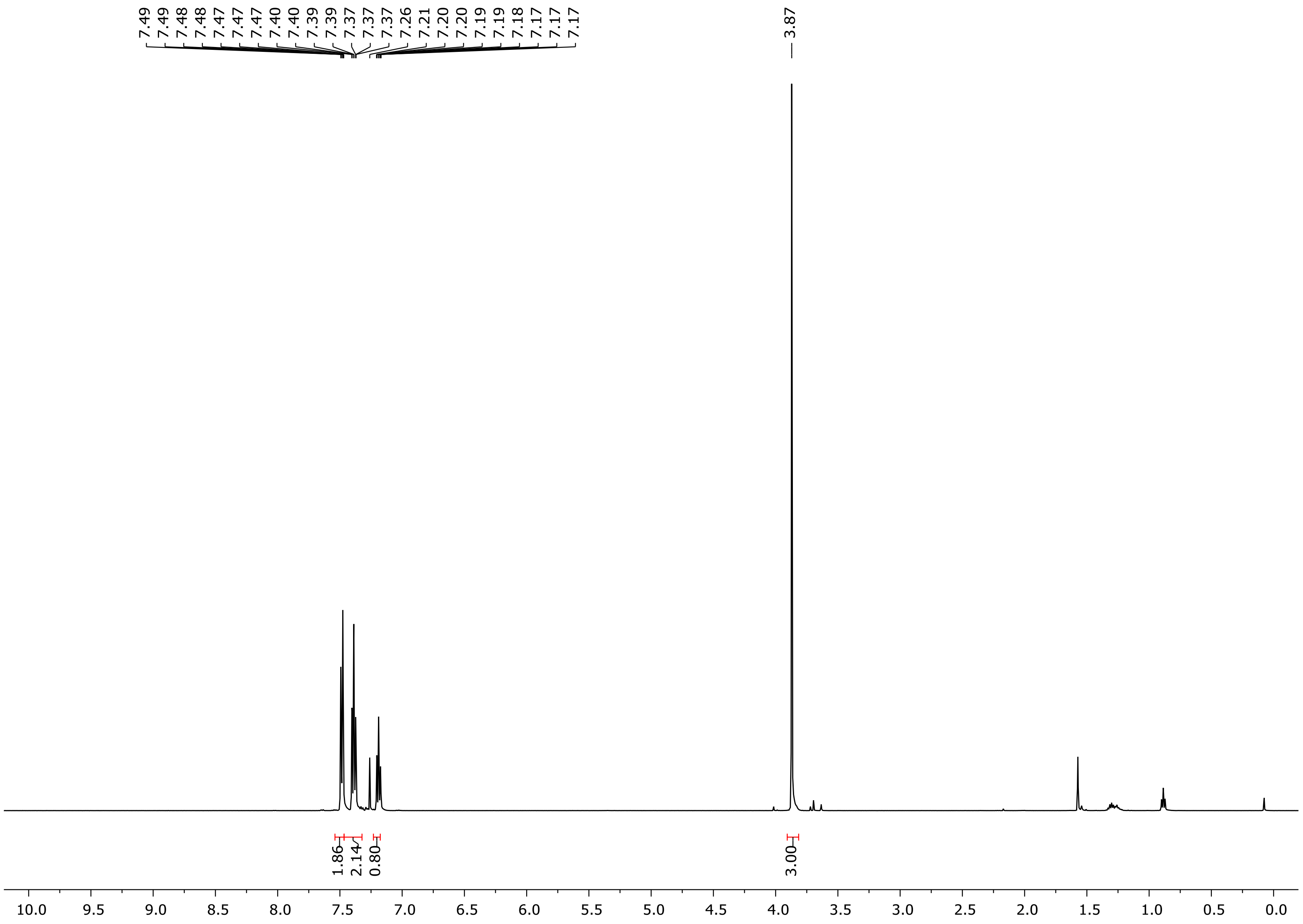

Figure S12: ^{13}**C NMR** (126 MHz, $CDCl_3$, 298 K) spectrum of compound **1f**

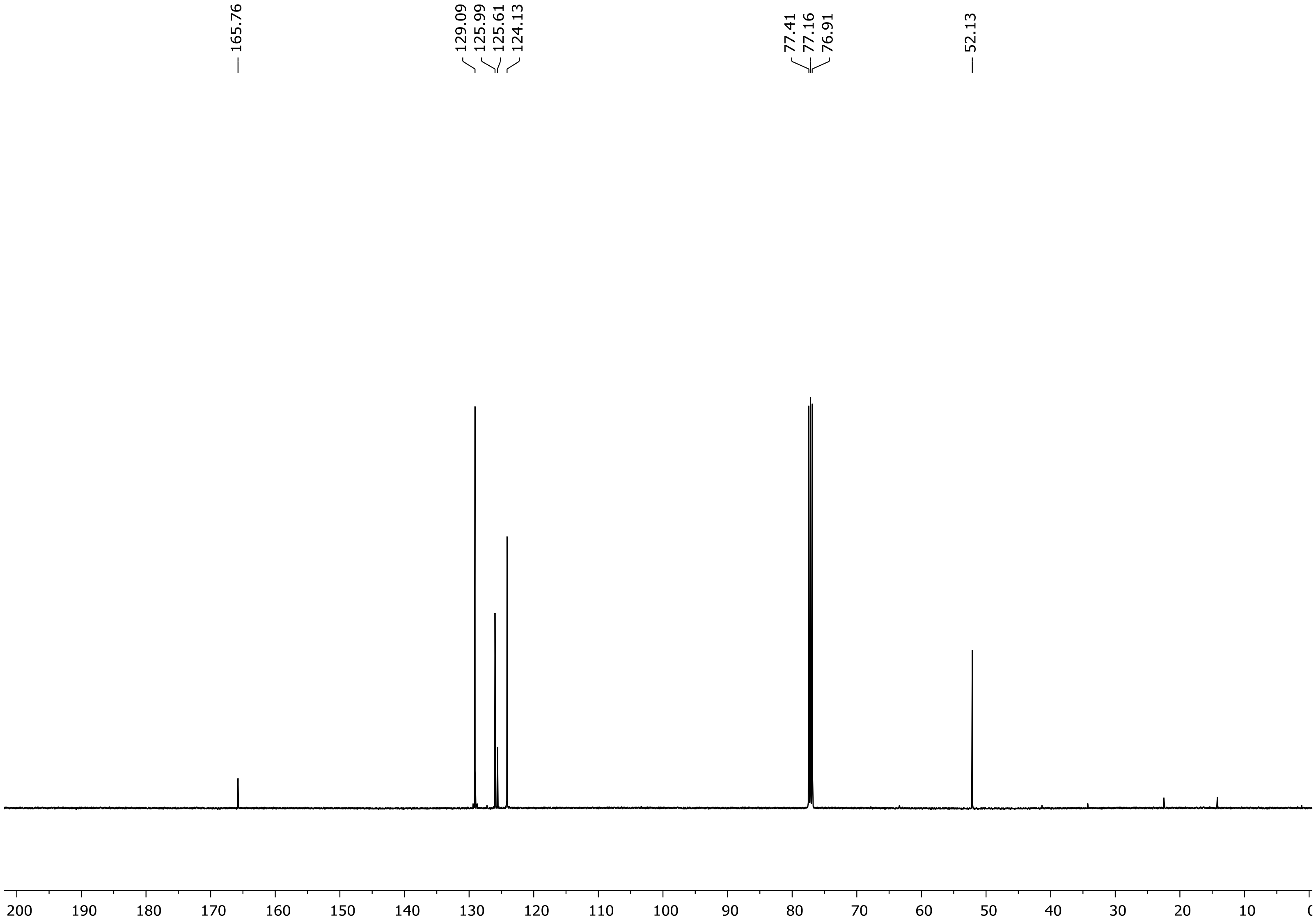

Figure S13: **^{1}H NMR** (400 MHz, $CDCl_3$, 298 K) spectrum of compound **2a**

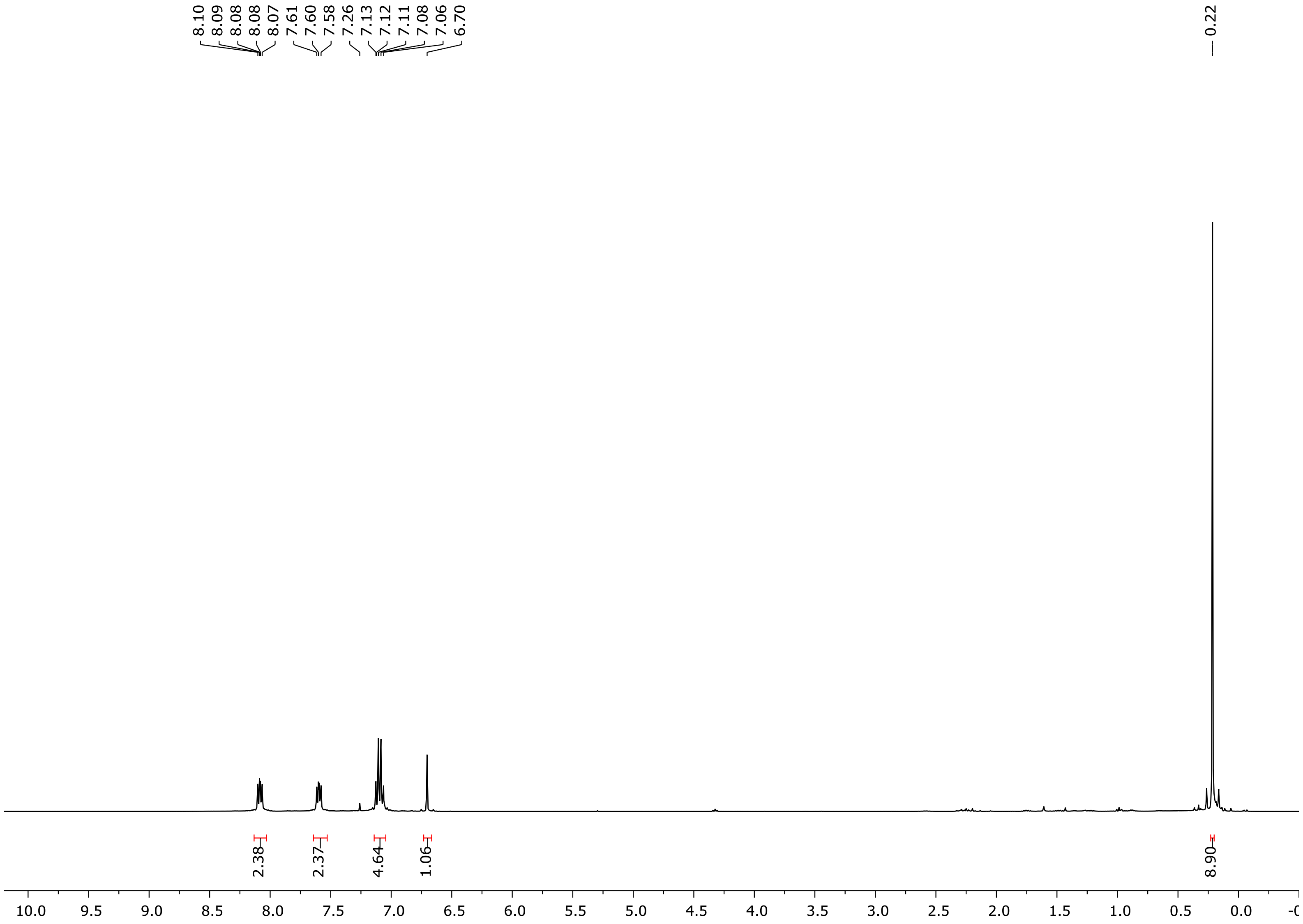

Figure S14: **^{13}C NMR** (101 MHz, $CDCl_3$, 298 K) spectrum of compound **2a**

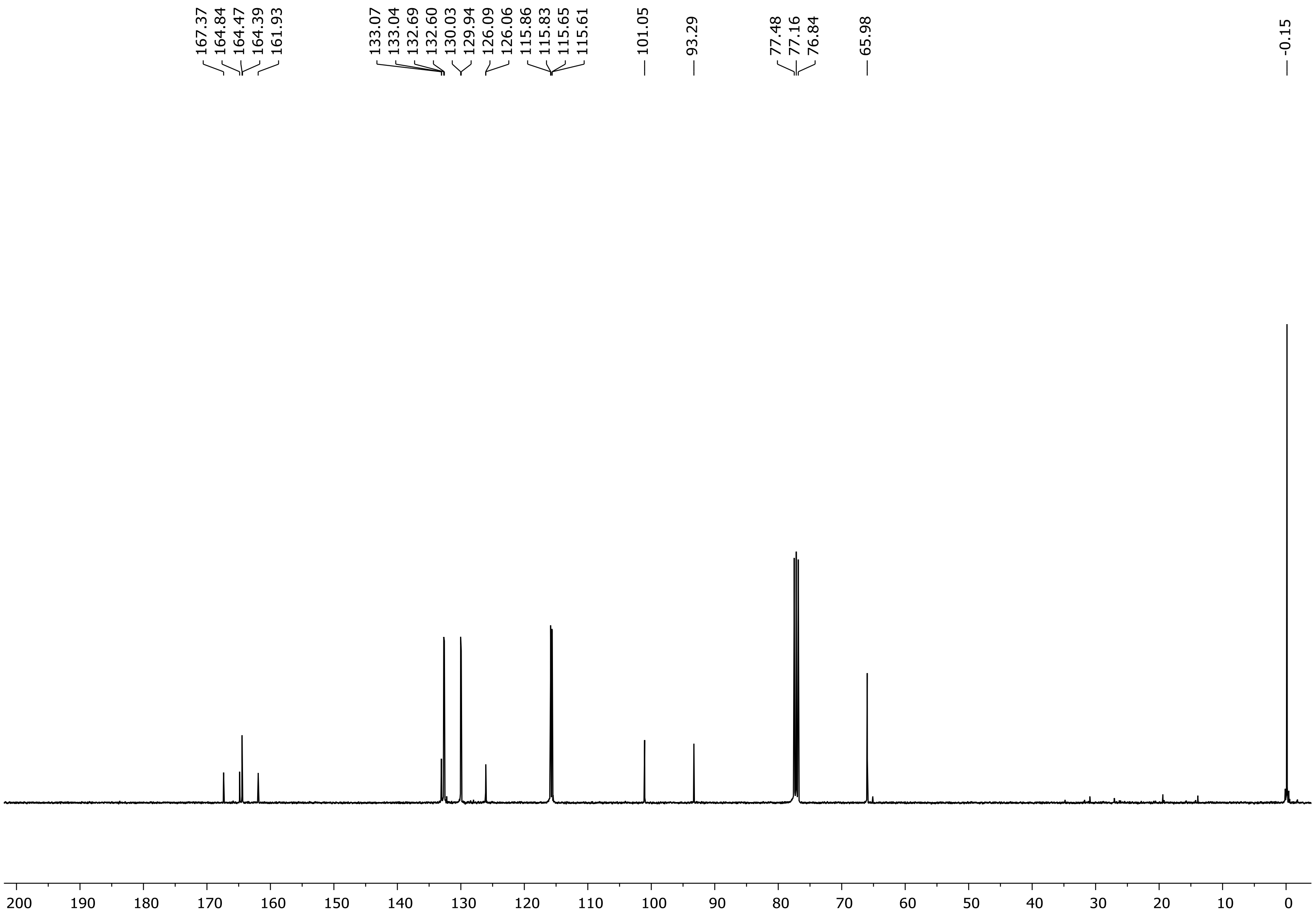

Figure S15: **^{19}F NMR** (376 MHz, $CDCl_3$, 298 K) spectrum of compound **2a**

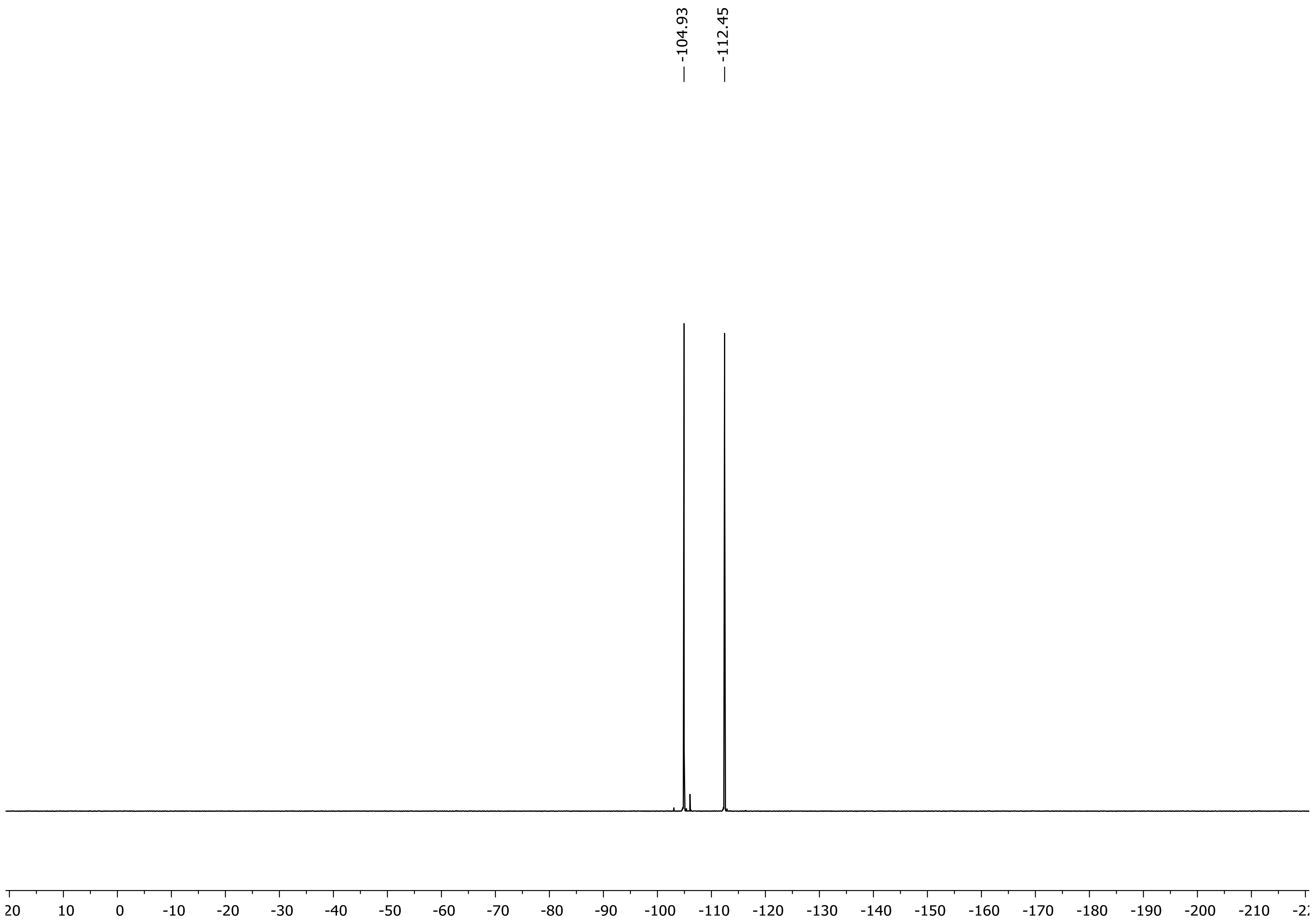

Figure S16: **^{1}H NMR** (400 MHz, $CDCl_3$, 298 K) spectrum of compound **2b**

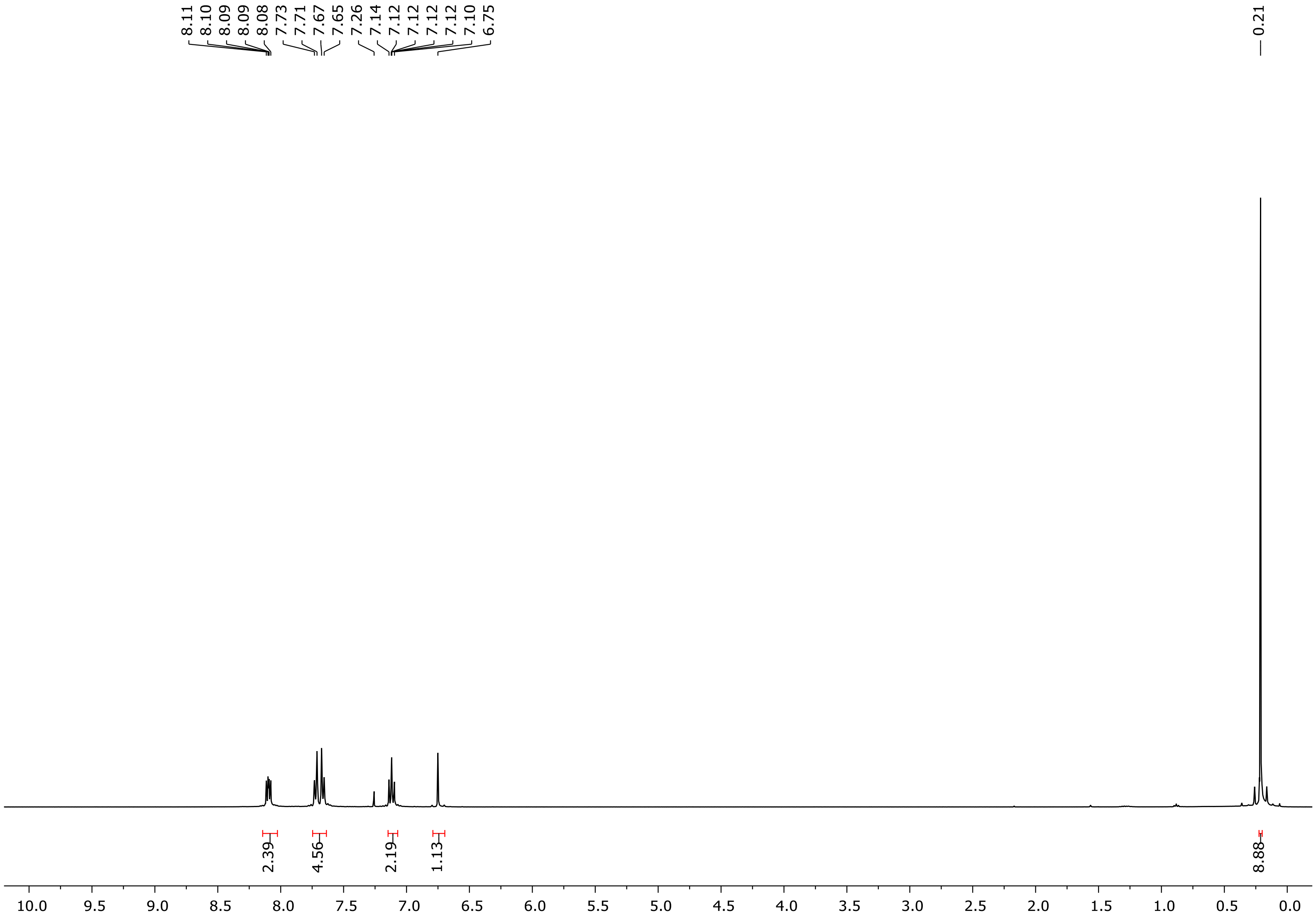

Figure S17: **^{13}C NMR** (101 MHz, $CDCl_3$, 298 K) spectrum of compound **2b**

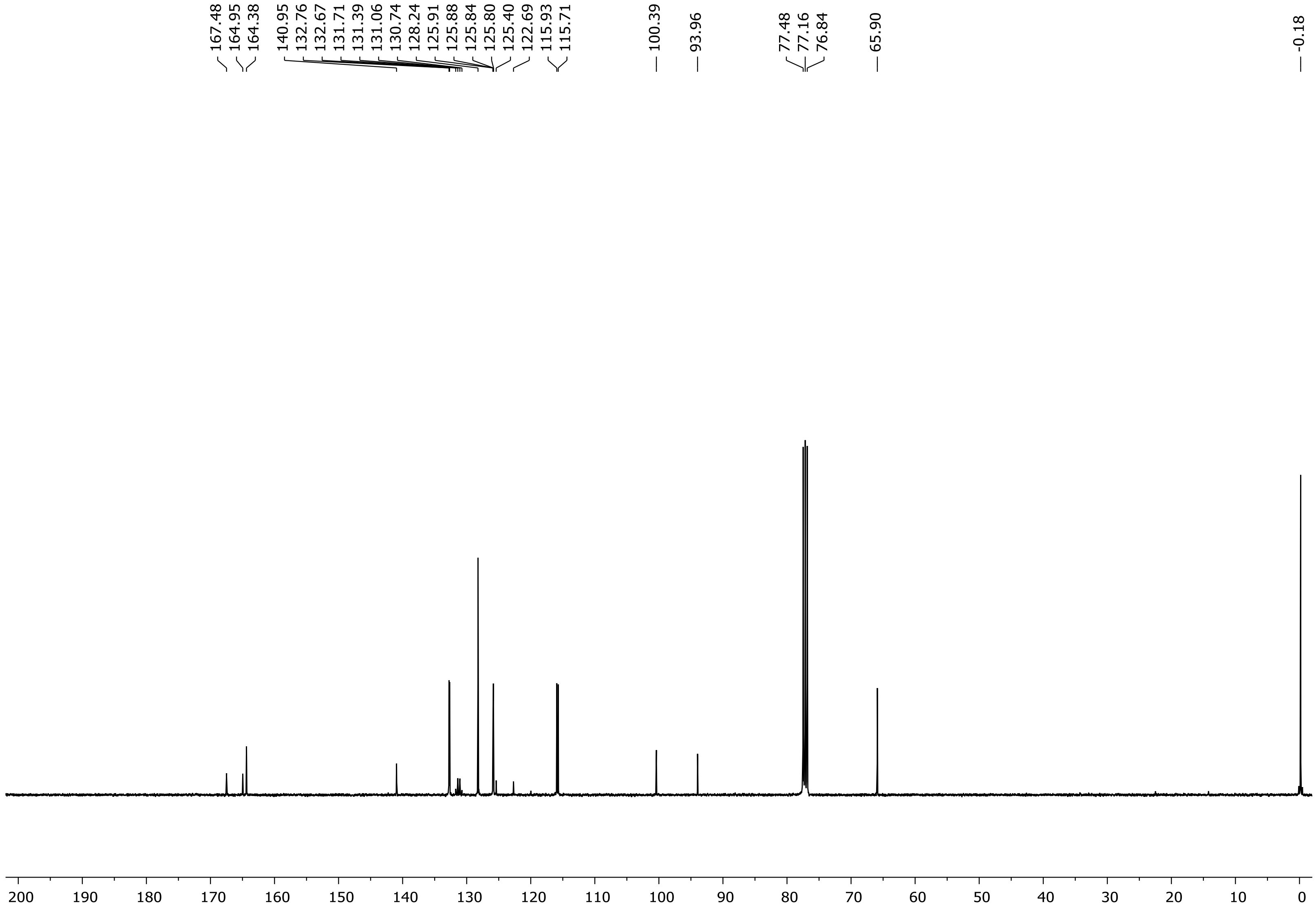

Figure S18: **^{19}F NMR** (376 MHz, $CDCl_3$, 298 K) spectrum of compound **2b**

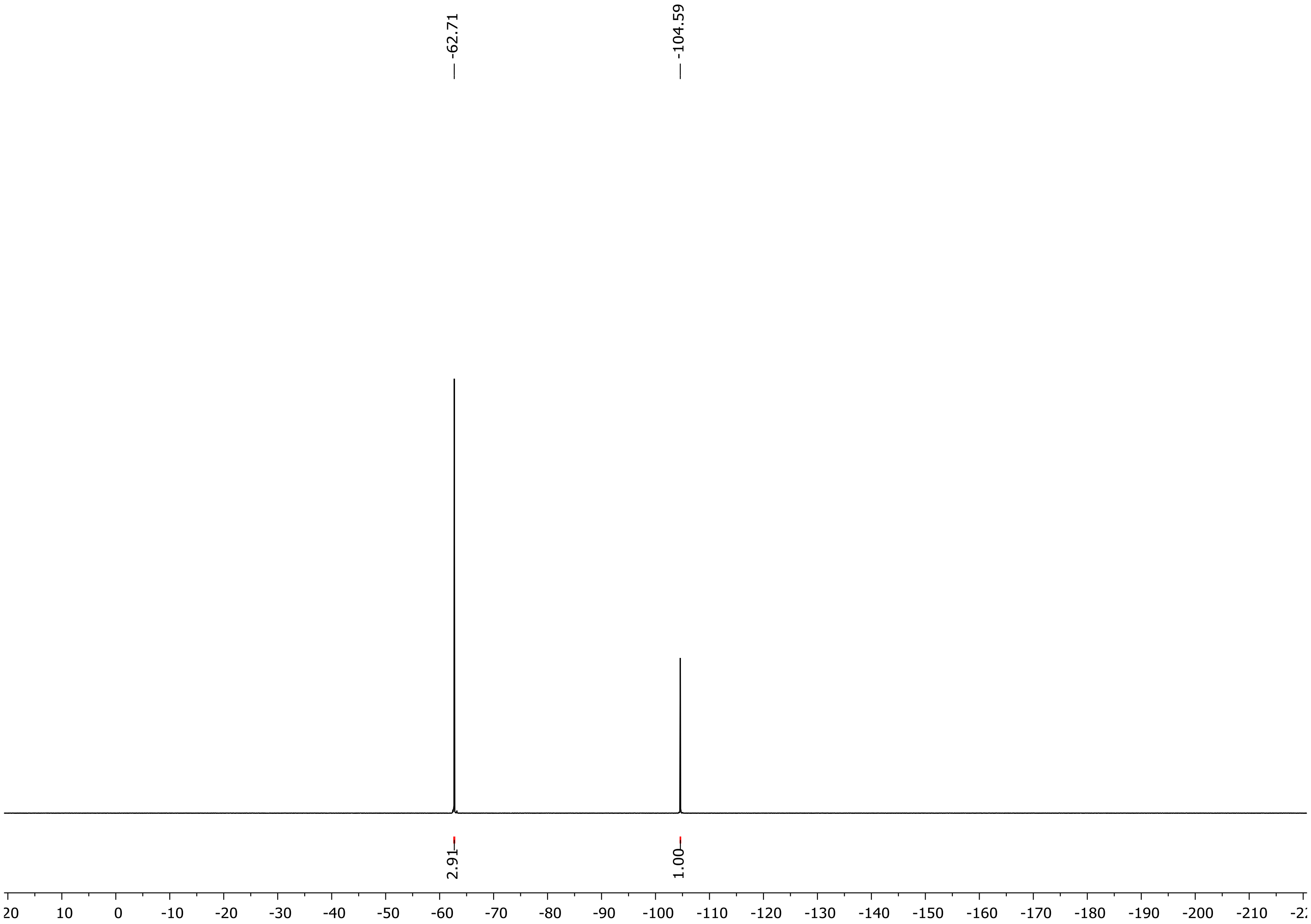

Figure S19: **^{1}H NMR** (400 MHz, $CDCl_3$, 298 K) spectrum of compound **2c**

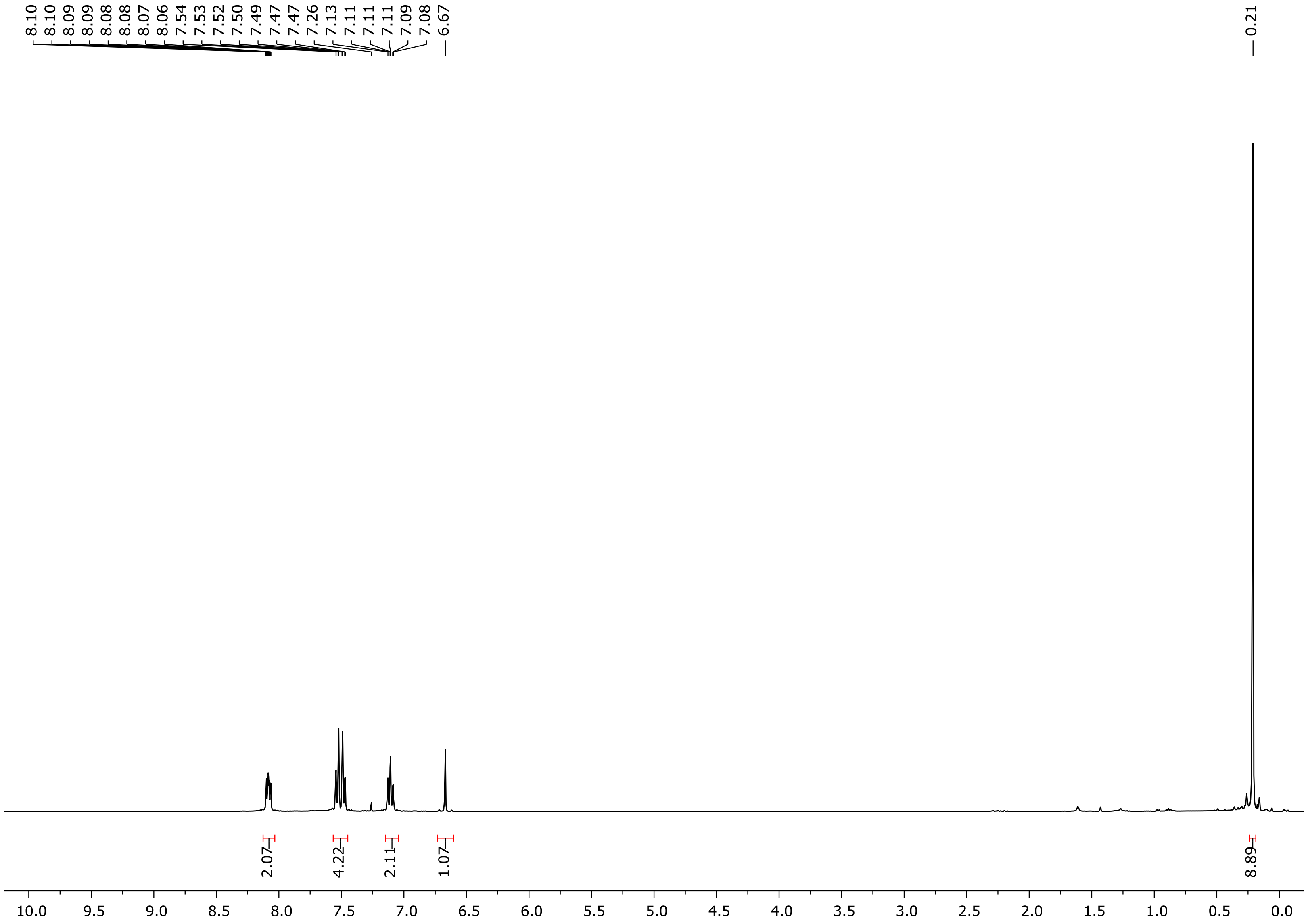

Figure S20: ^{13}**C NMR** (101 MHz, $CDCl_3$, 298 K) spectrum of compound **2c**

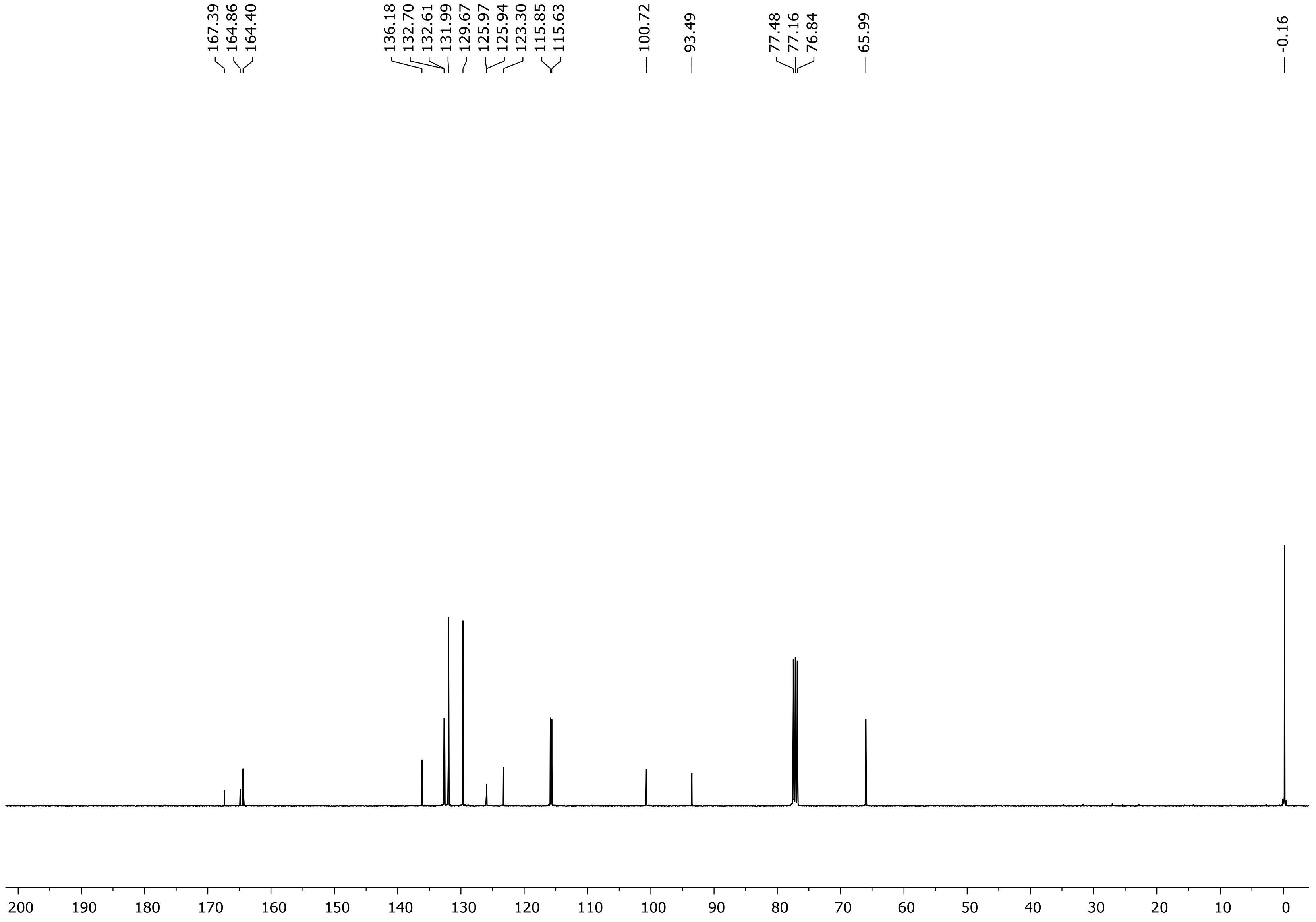

Figure S21: ^{19}**F NMR** (376 MHz, $CDCl_3$, 298 K) spectrum of compound **2c**

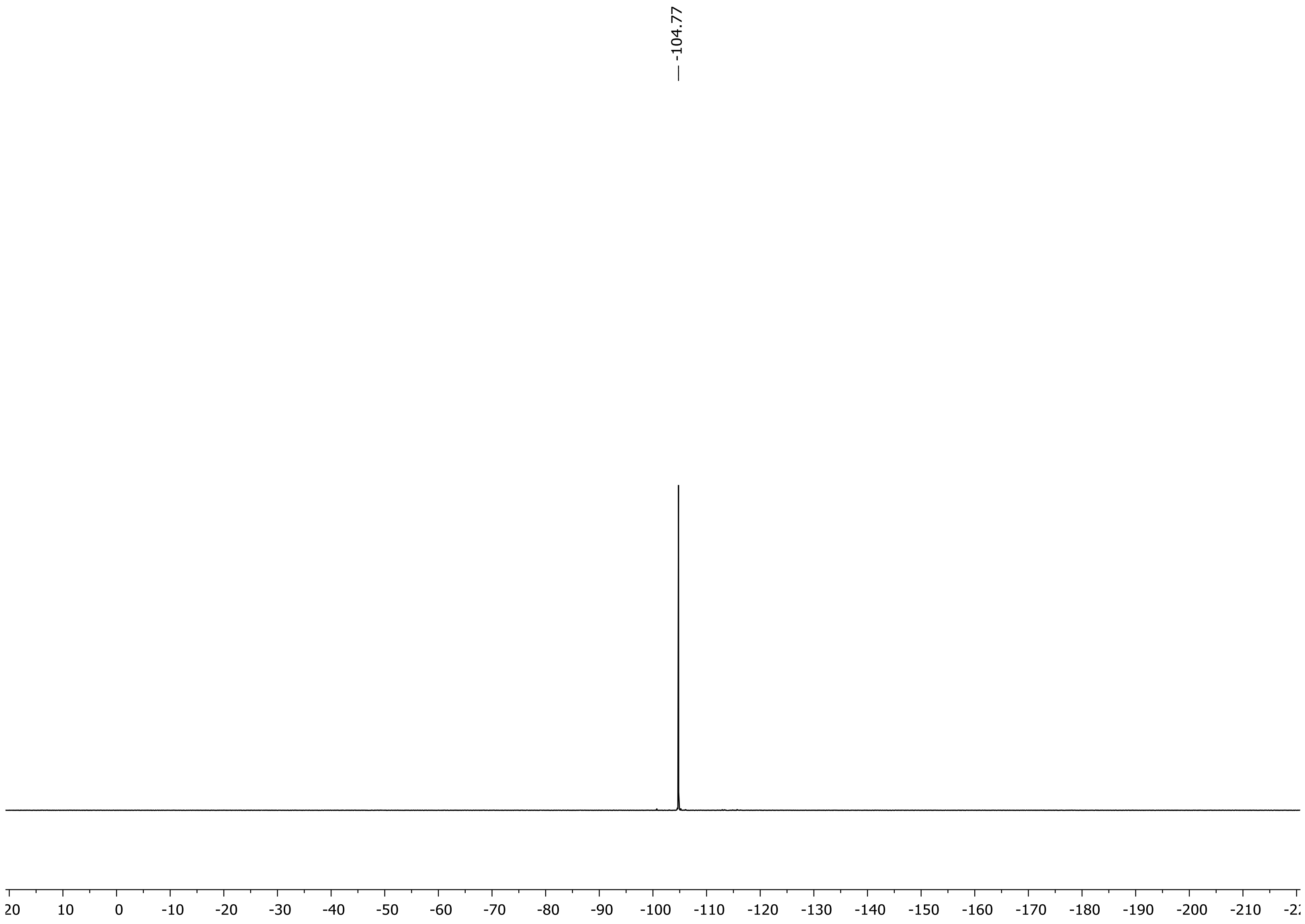

Figure S22: ^{1}**H NMR** (500 MHz, $CDCl_3$, 298 K) spectrum of compound **2d**

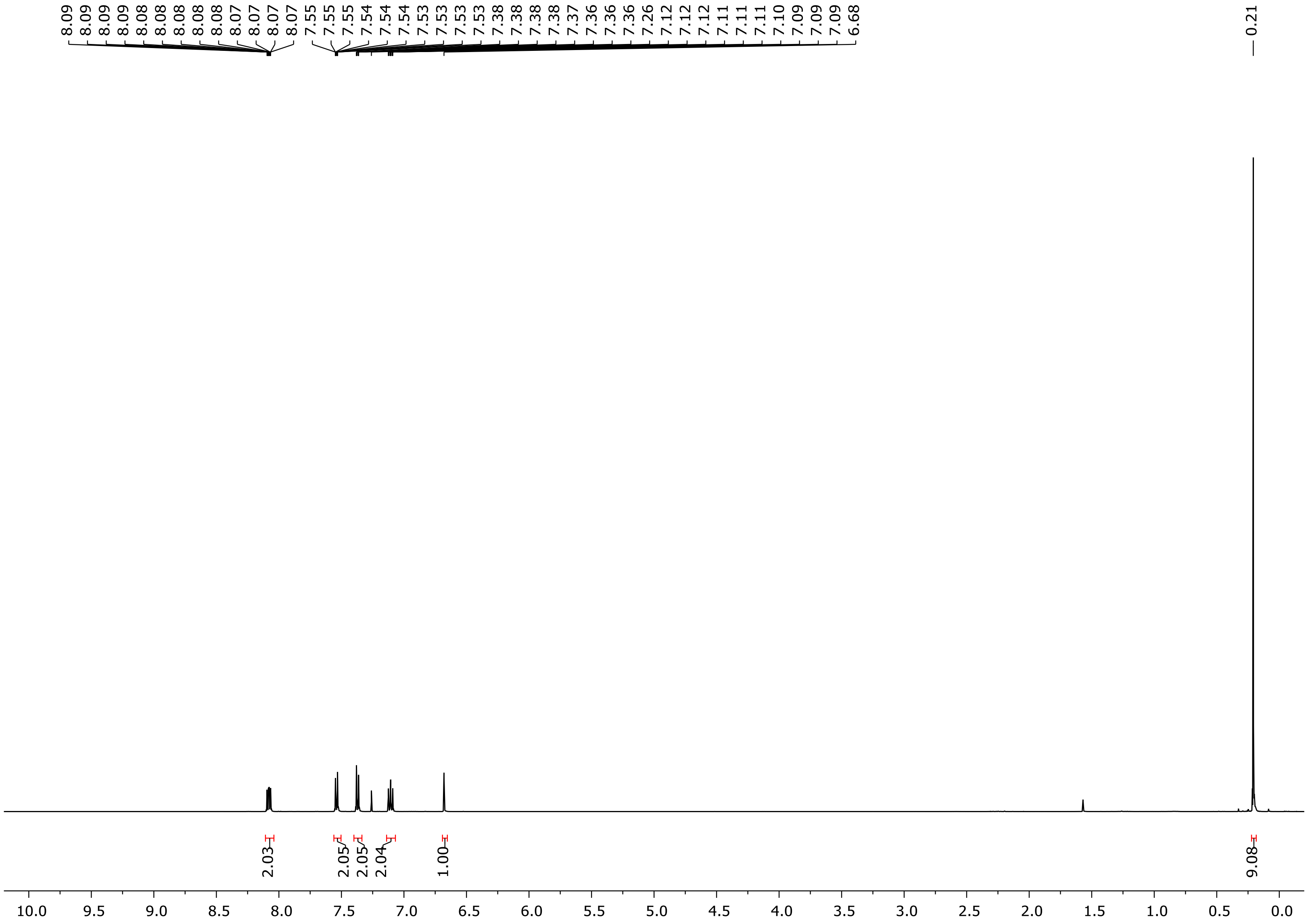

Figure S23: **^{13}C NMR** (126 MHz, $CDCl_3$, 298 K) spectrum of compound **2d**

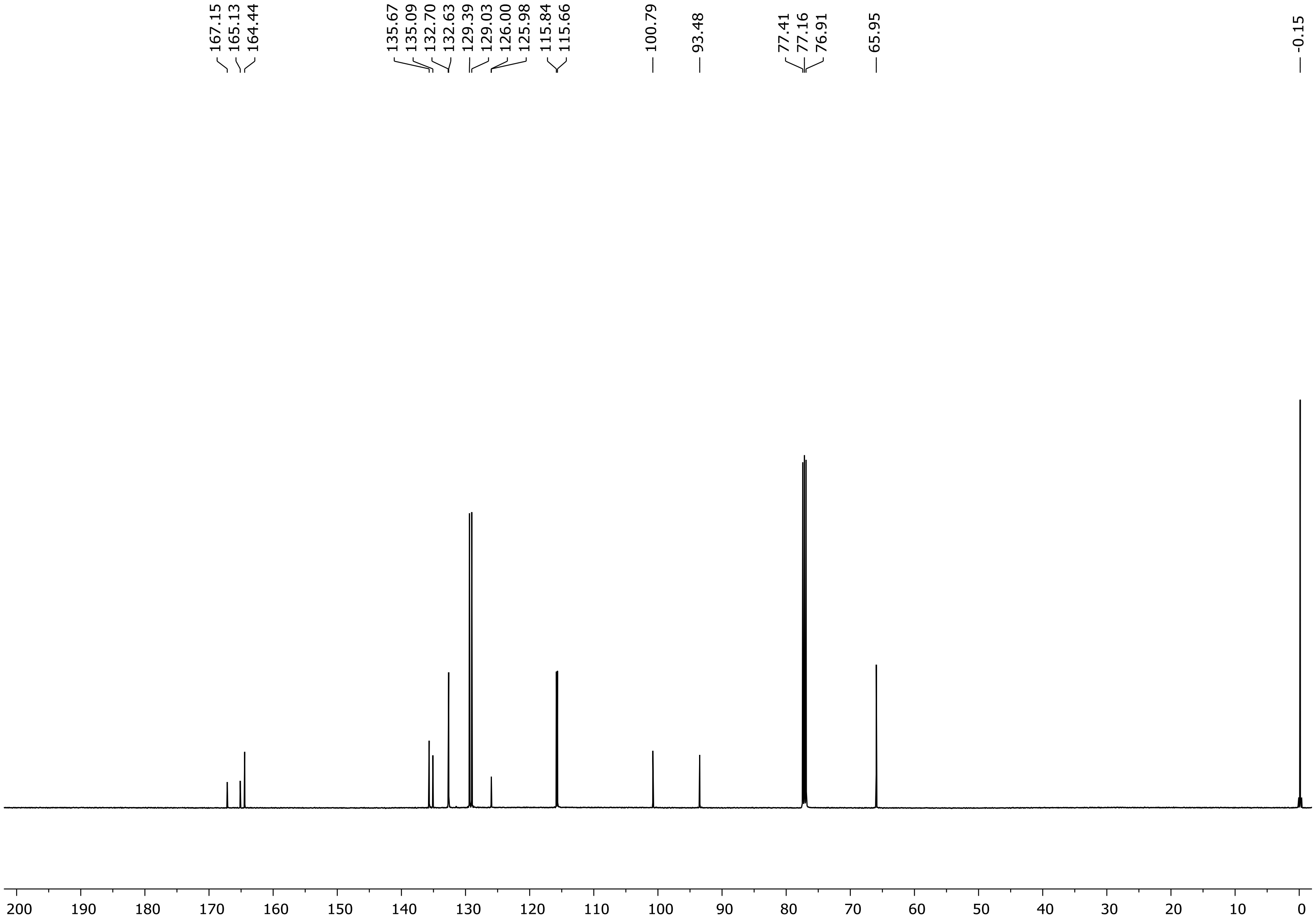

Figure S24: **^{19}F NMR** (471 MHz, $CDCl_3$, 298 K) spectrum of compound **2d**

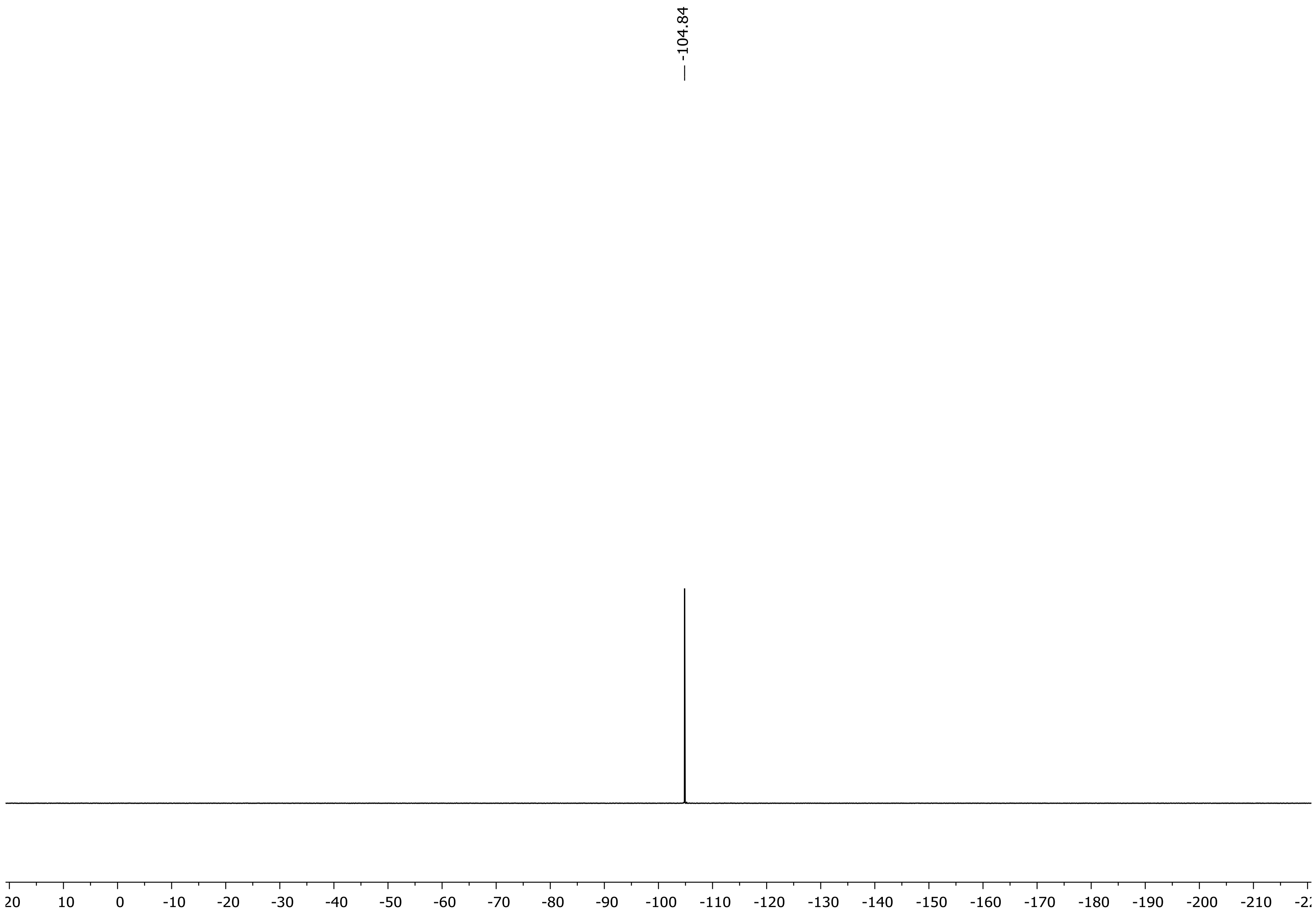

Figure S25: **^{1}H NMR** (500 MHz, $CDCl_3$, 298 K) spectrum of compound **2e**

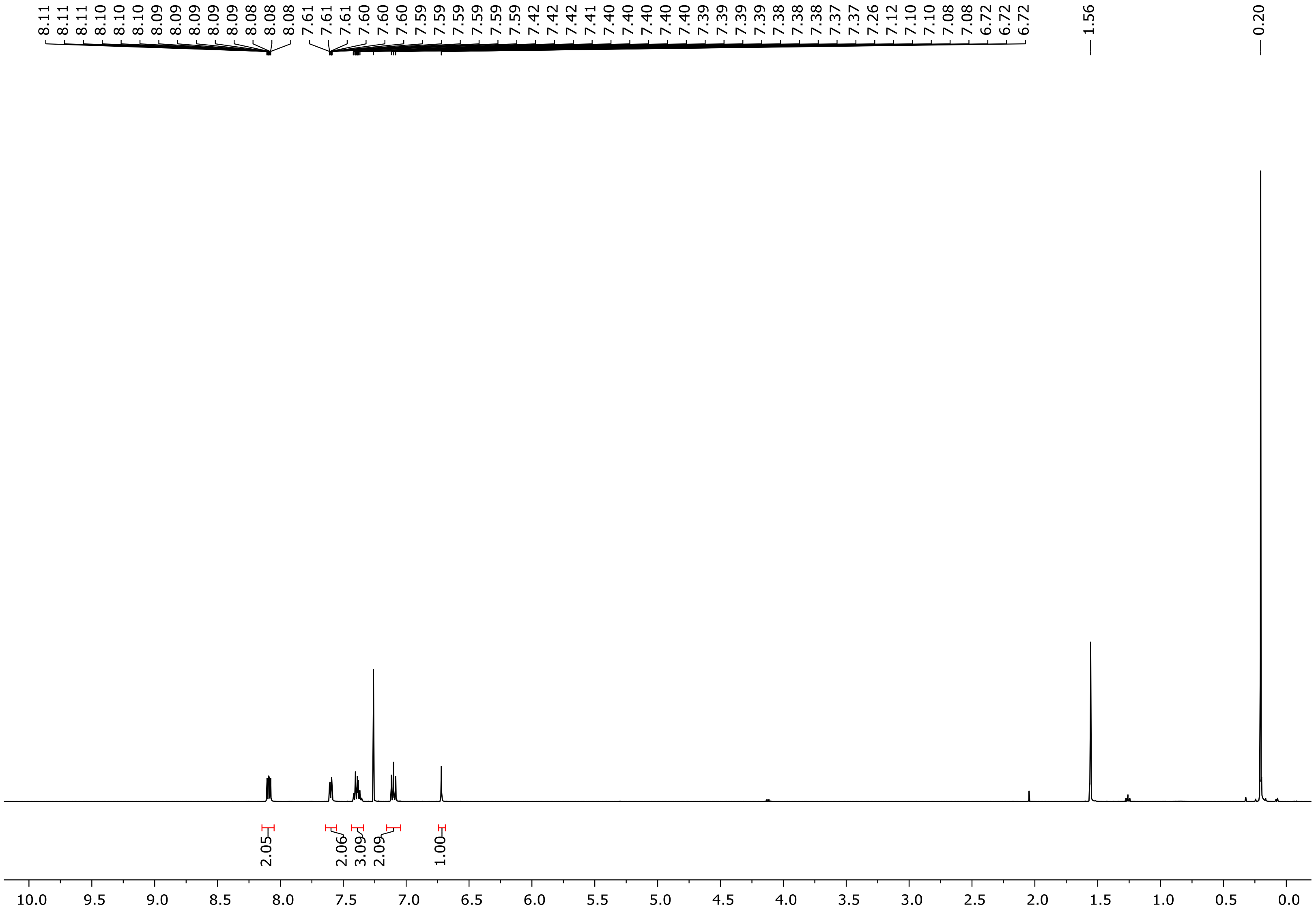

Figure S26: **^{13}C NMR** (126 MHz, $CDCl_3$, 298 K) spectrum of compound **2e**

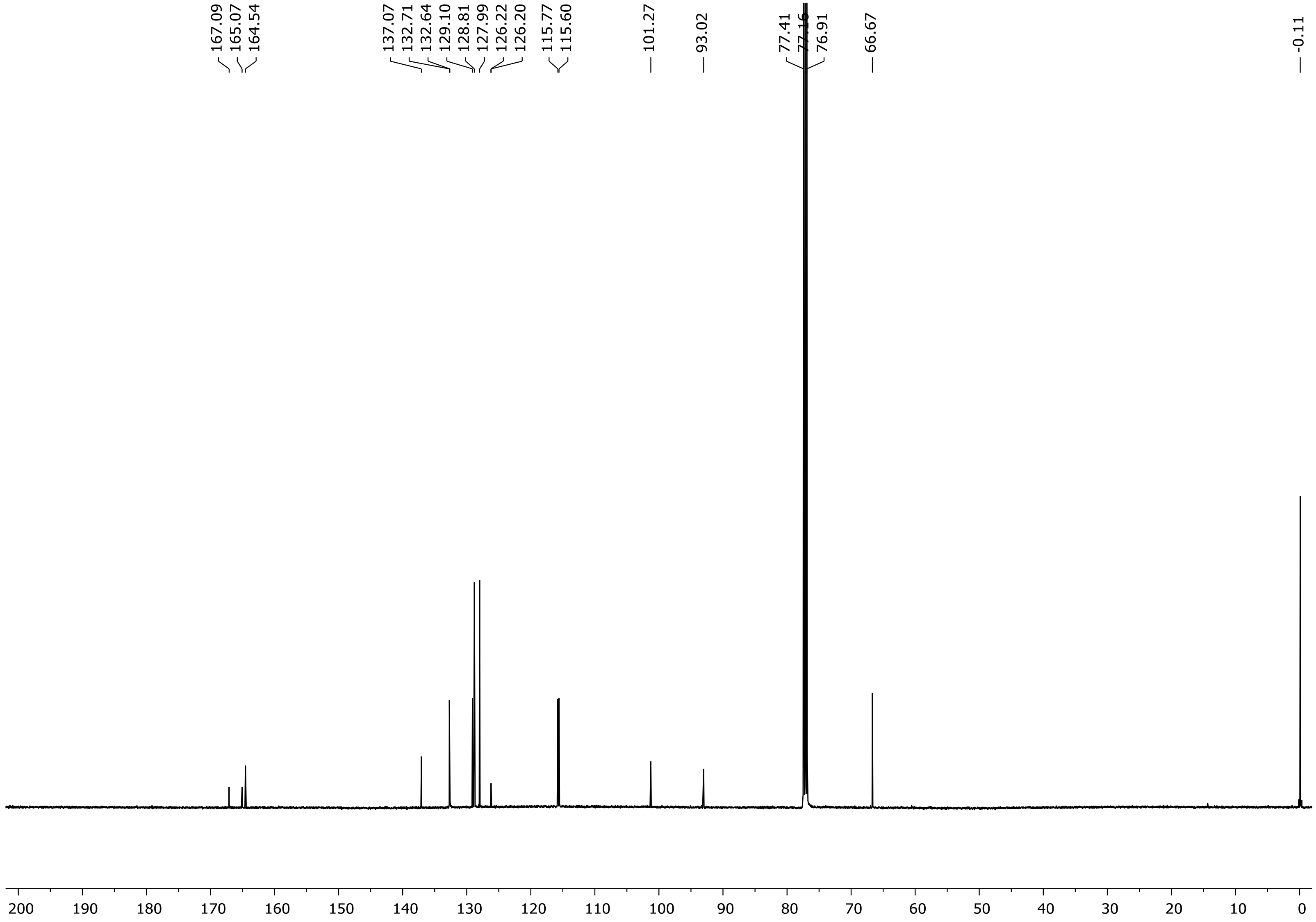

Figure S27: ^{19}**F NMR** (471 MHz, $CDCl_3$, 298 K) spectrum of compound **2e**

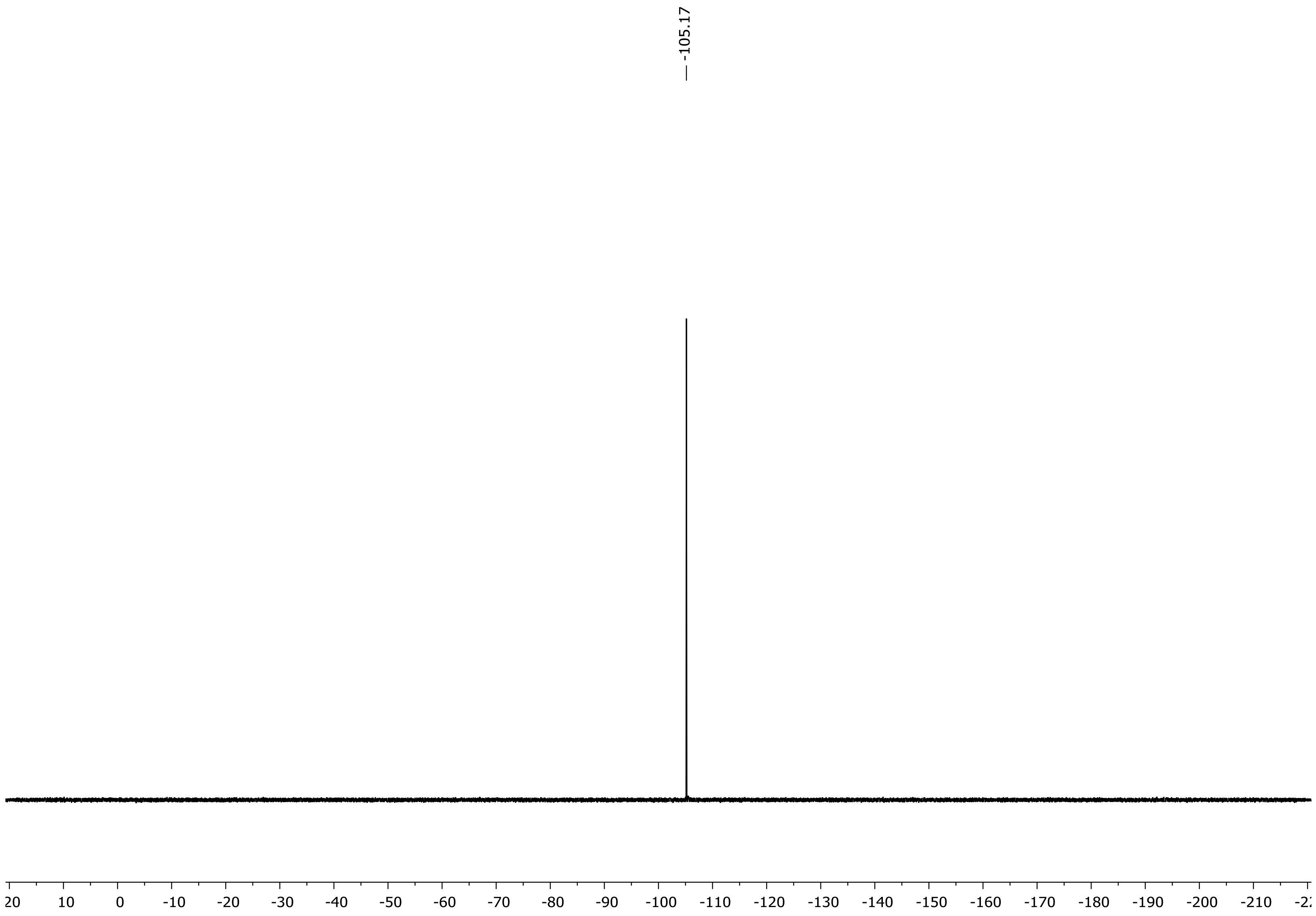

Figure S28: **^{1}H NMR** (500 MHz, $CDCl_3$, 298 K) spectrum of compound **2f**

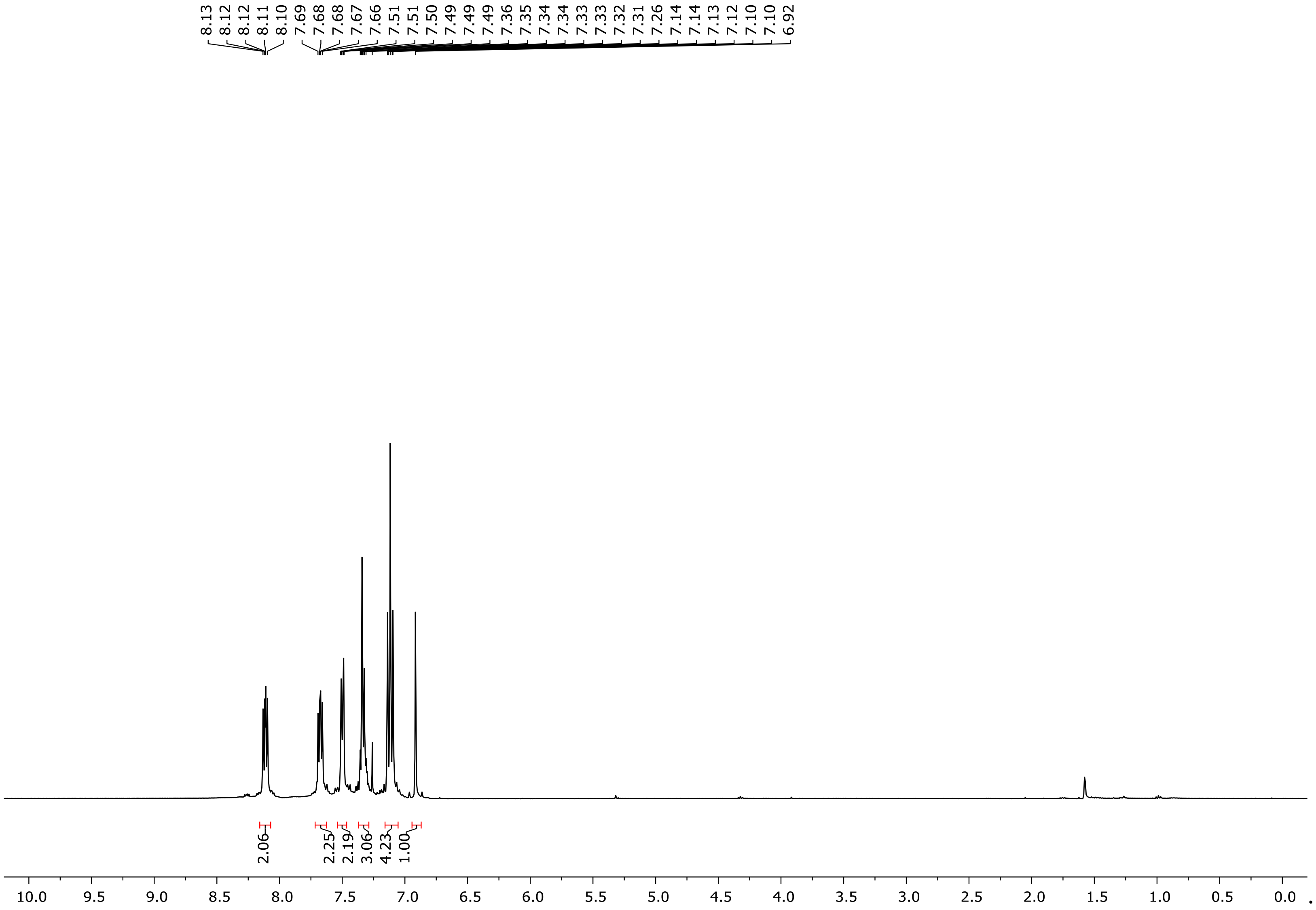

Figure S29: **^{13}C NMR** (126 MHz, $CDCl_3$, 298 K) spectrum of compound **2f**

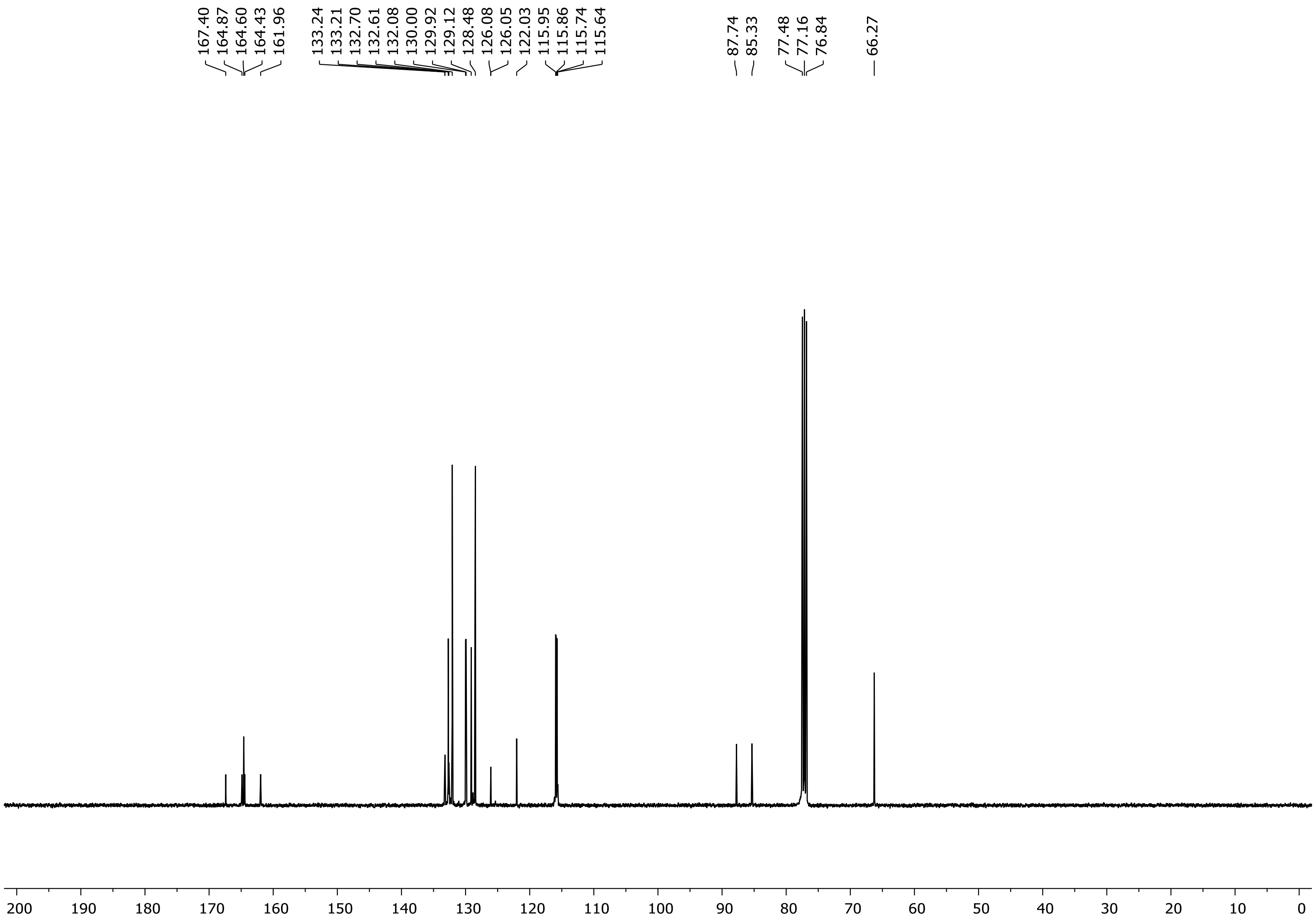

Figure S30: ^{19}**F NMR** (471 MHz, $CDCl_3$, 298 K) spectrum of compound **2f**

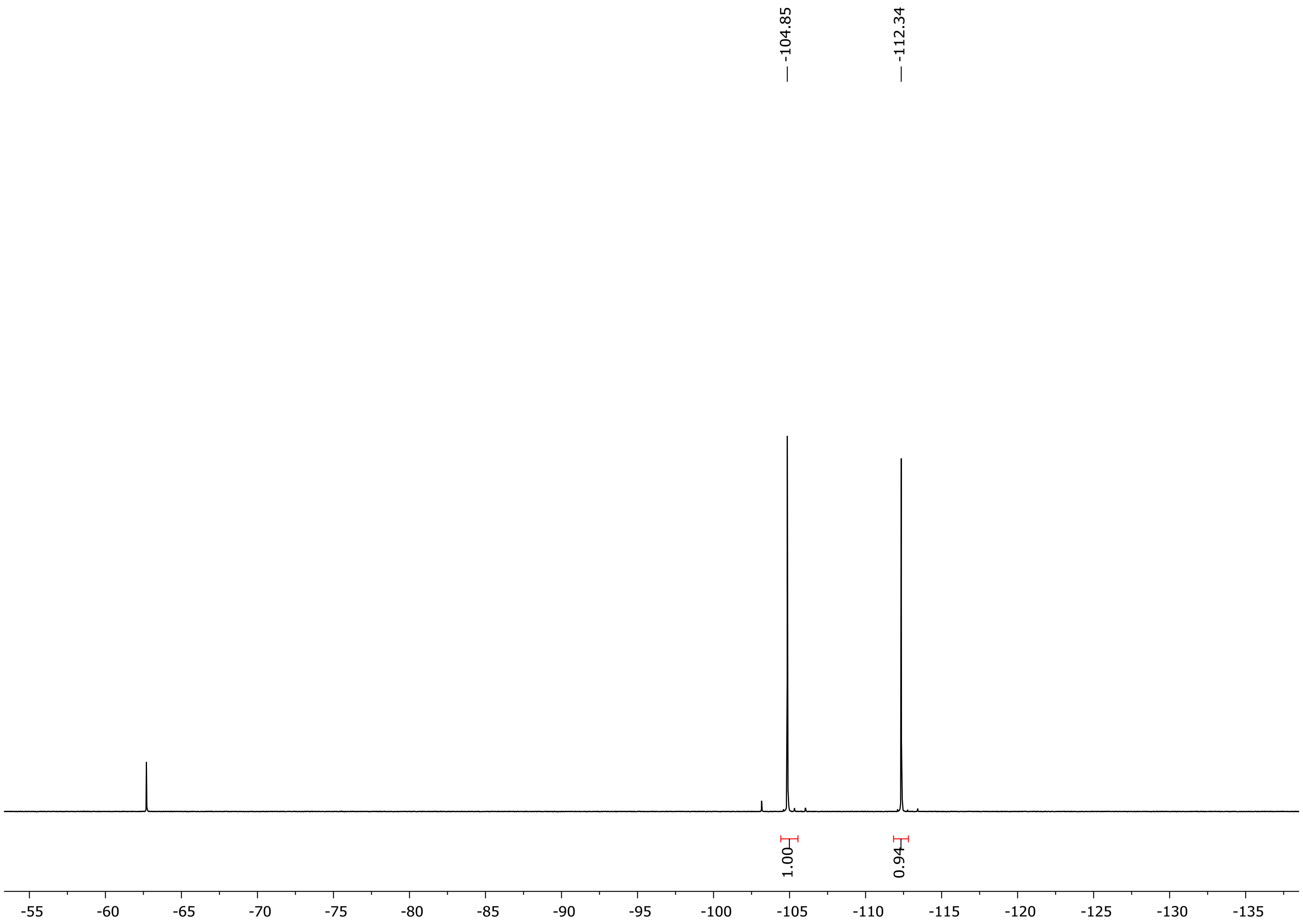

Figure S31: ^{1}**H NMR** (500 MHz, $CDCl_3$, 298 K) spectrum of compound **2g**

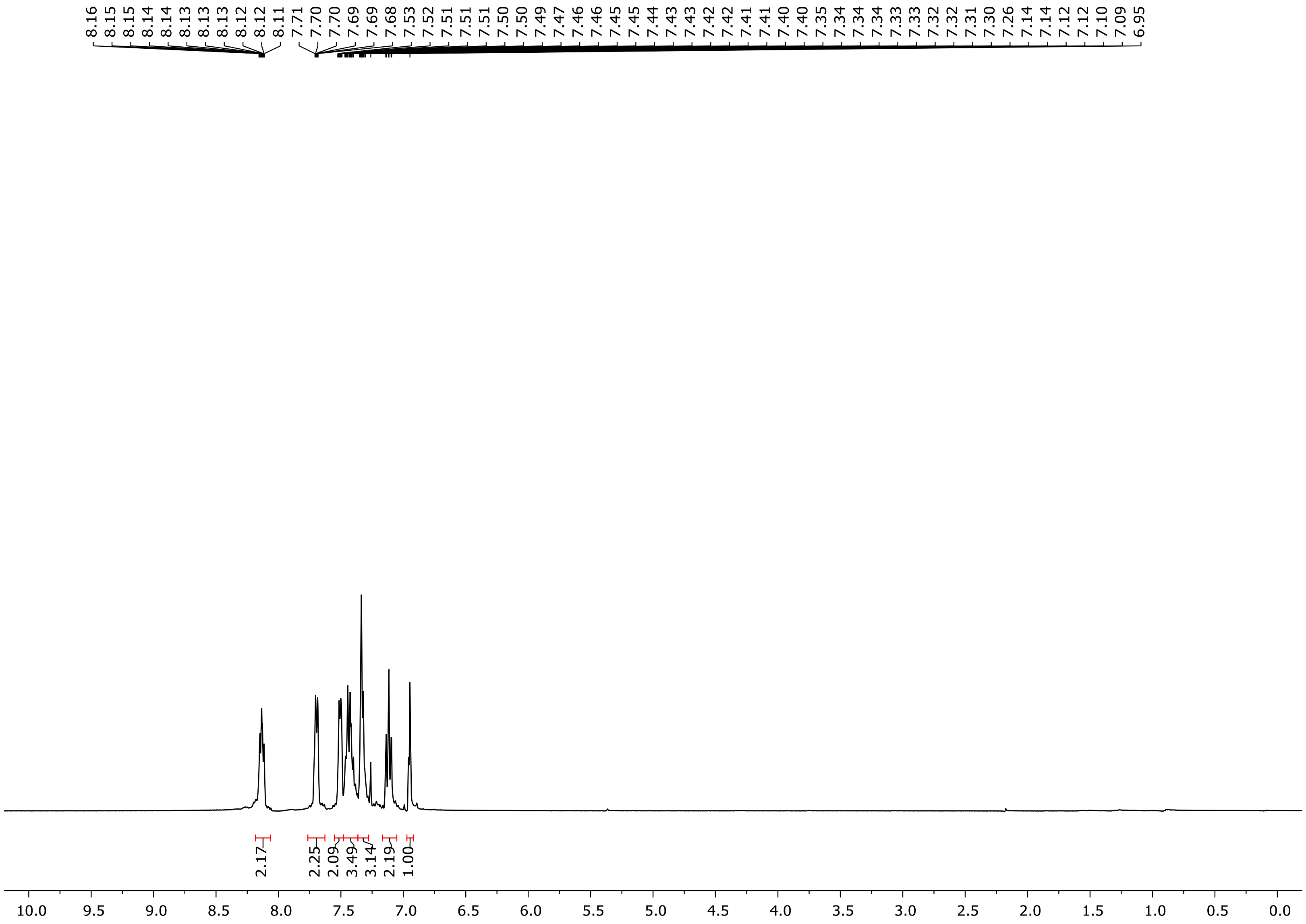

Figure S32: ^{13}**C NMR** (126 MHz, $CDCl_3$, 298 K) spectrum of compound **2g**

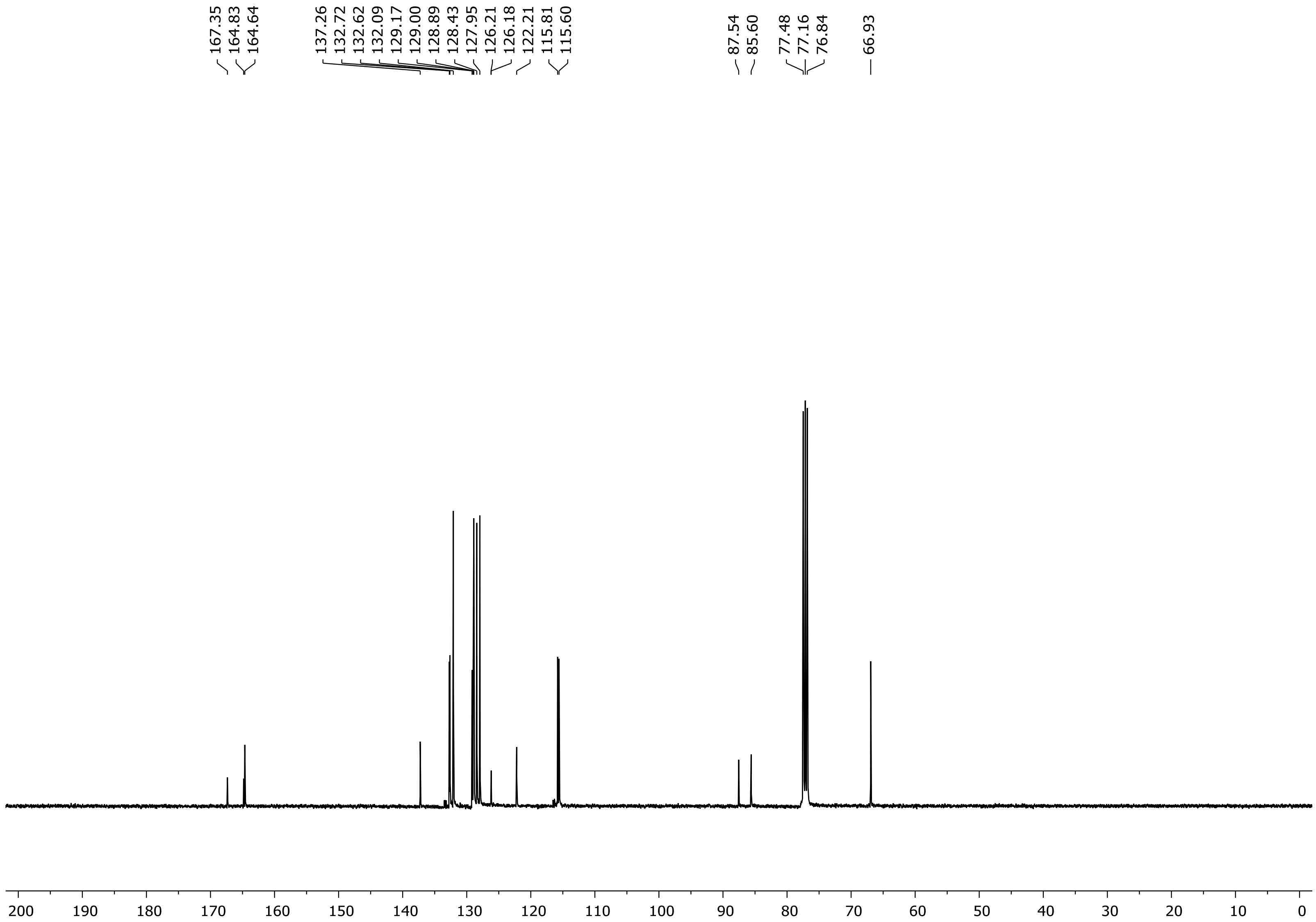

Figure S33: **^{19}F NMR** (471 MHz, $CDCl_3$, 298 K) spectrum of compound **2g**

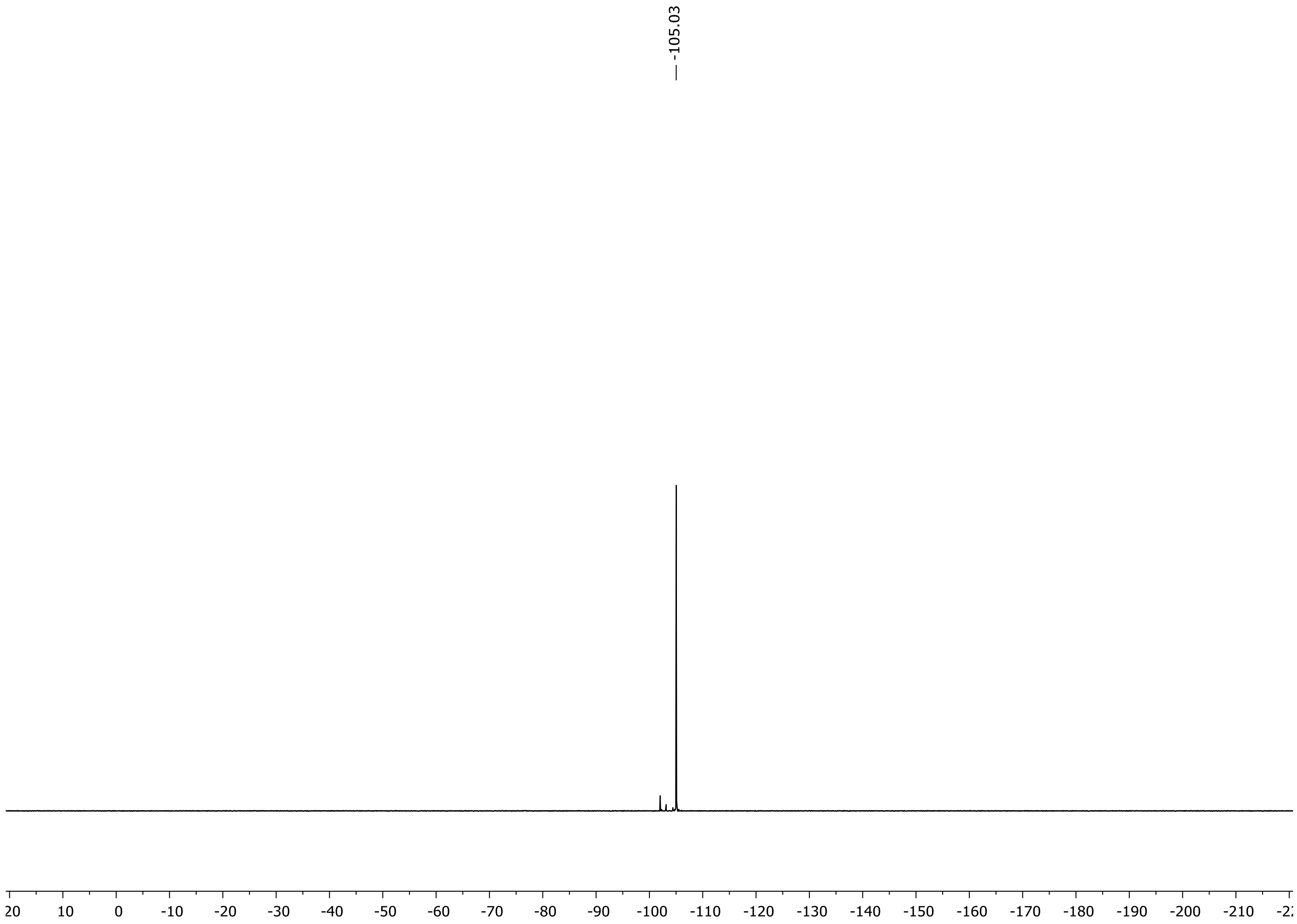

Figure S34: **^{1}H NMR** (400 MHz, $CDCl_3$, 298 K) spectrum of compound **2h**

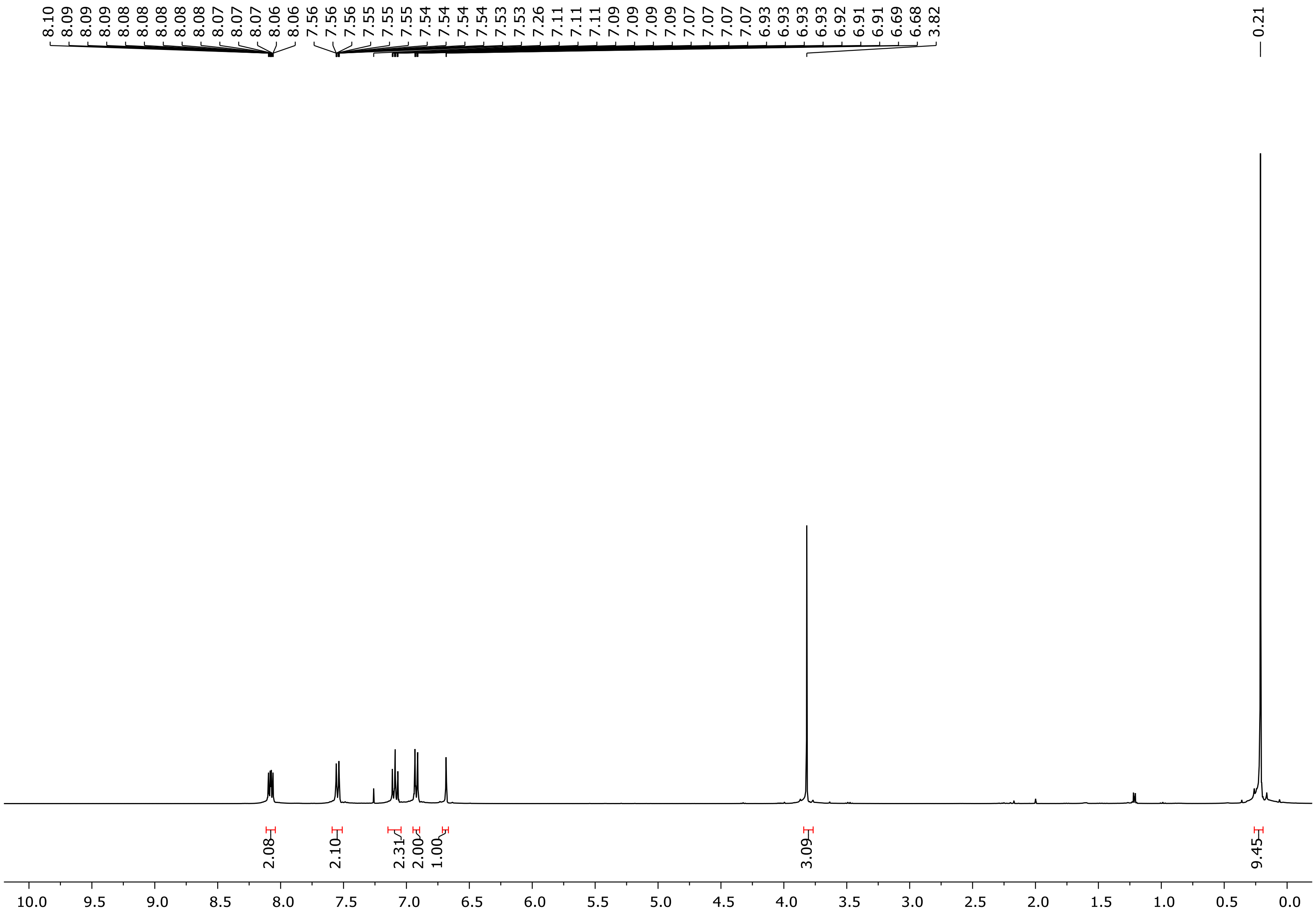

Figure S35: ^{13}**C NMR** (101 MHz, $CDCl_3$, 298 K) spectrum of compound **2h**

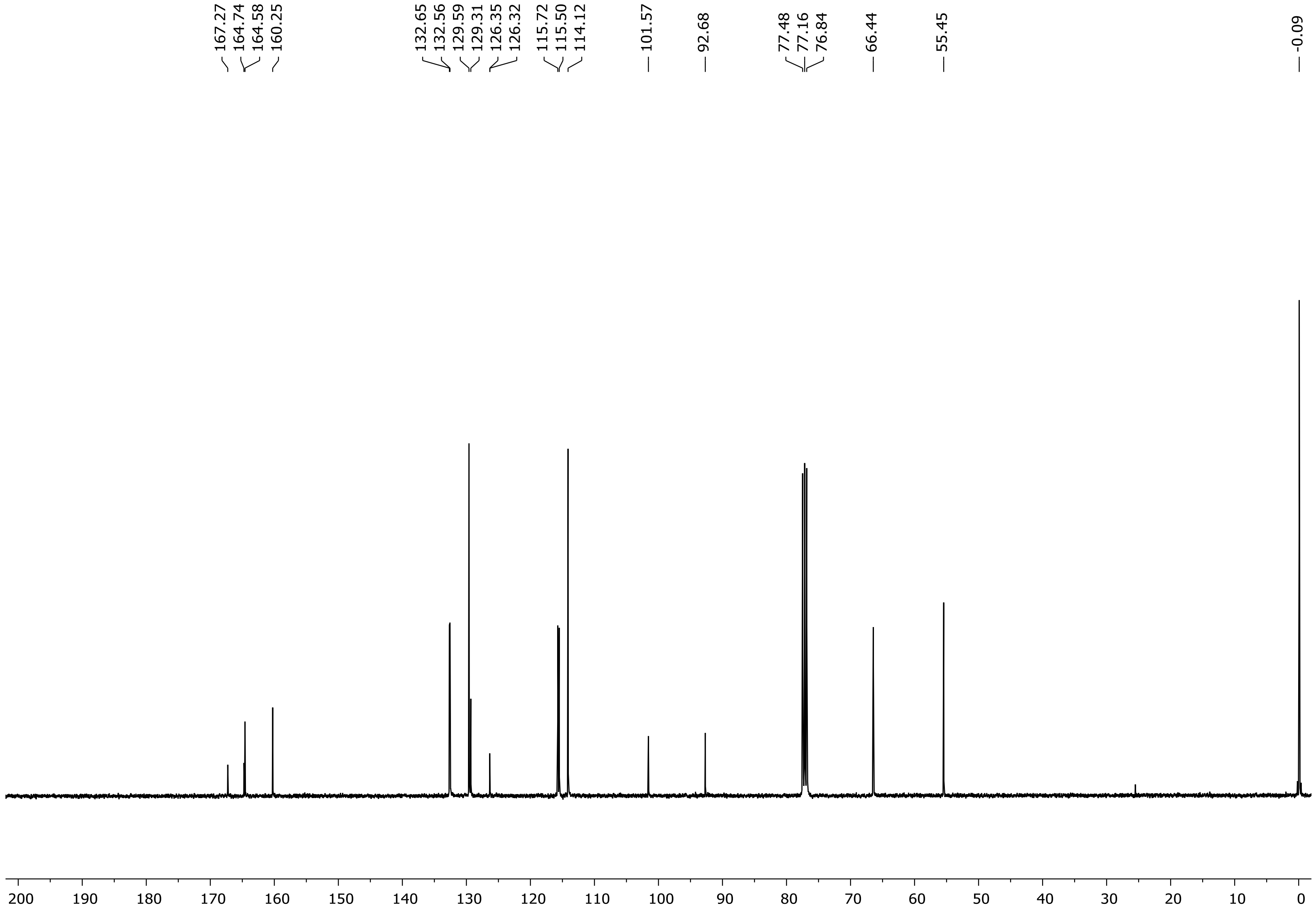

Figure S36: **^{19}F NMR** (376 MHz, $CDCl_3$, 298 K) spectrum of compound **2h**

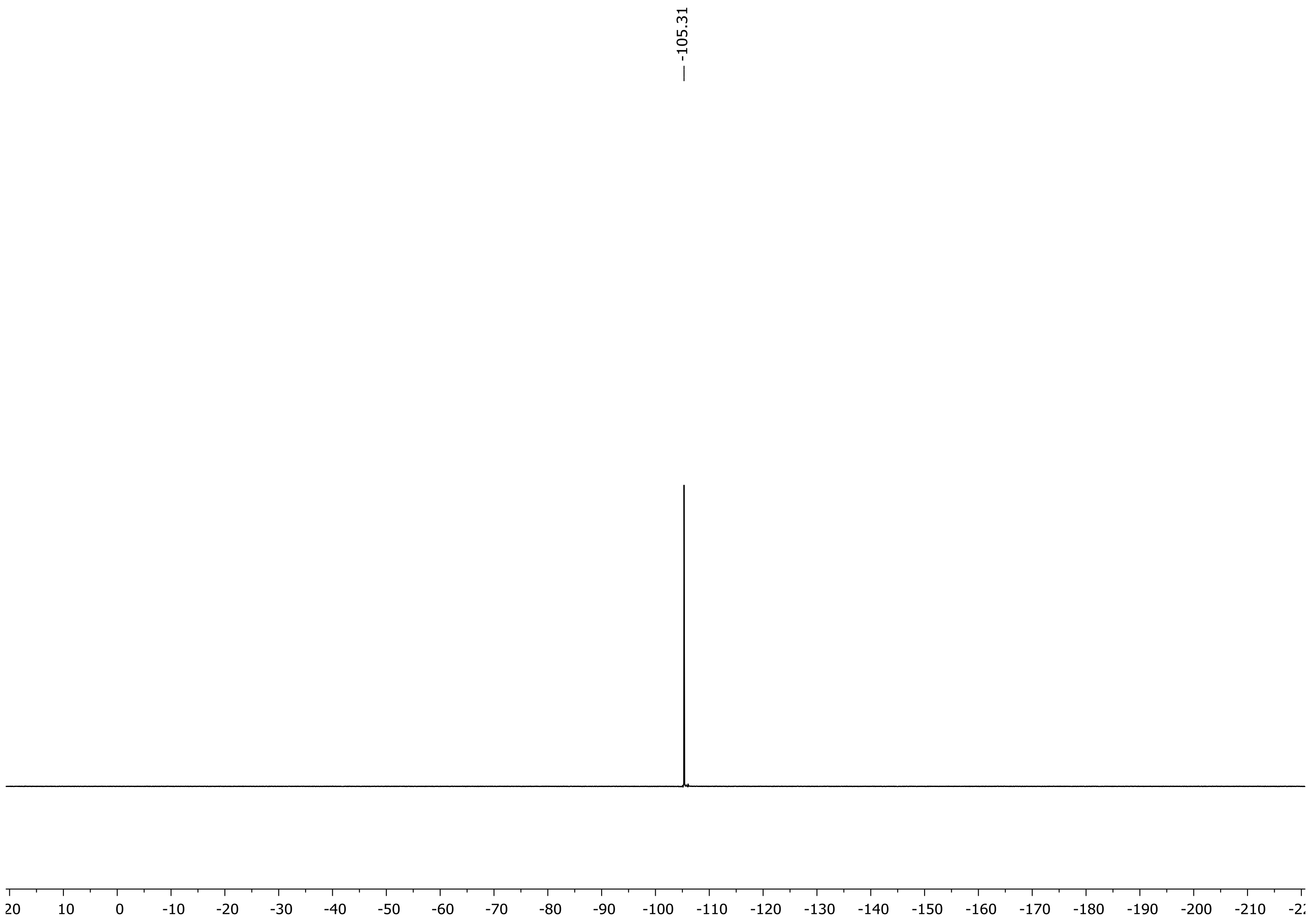

Figure S37: **^{1}H NMR** (400 MHz, $CDCl_3$, 298 K) spectrum of compound **2i**

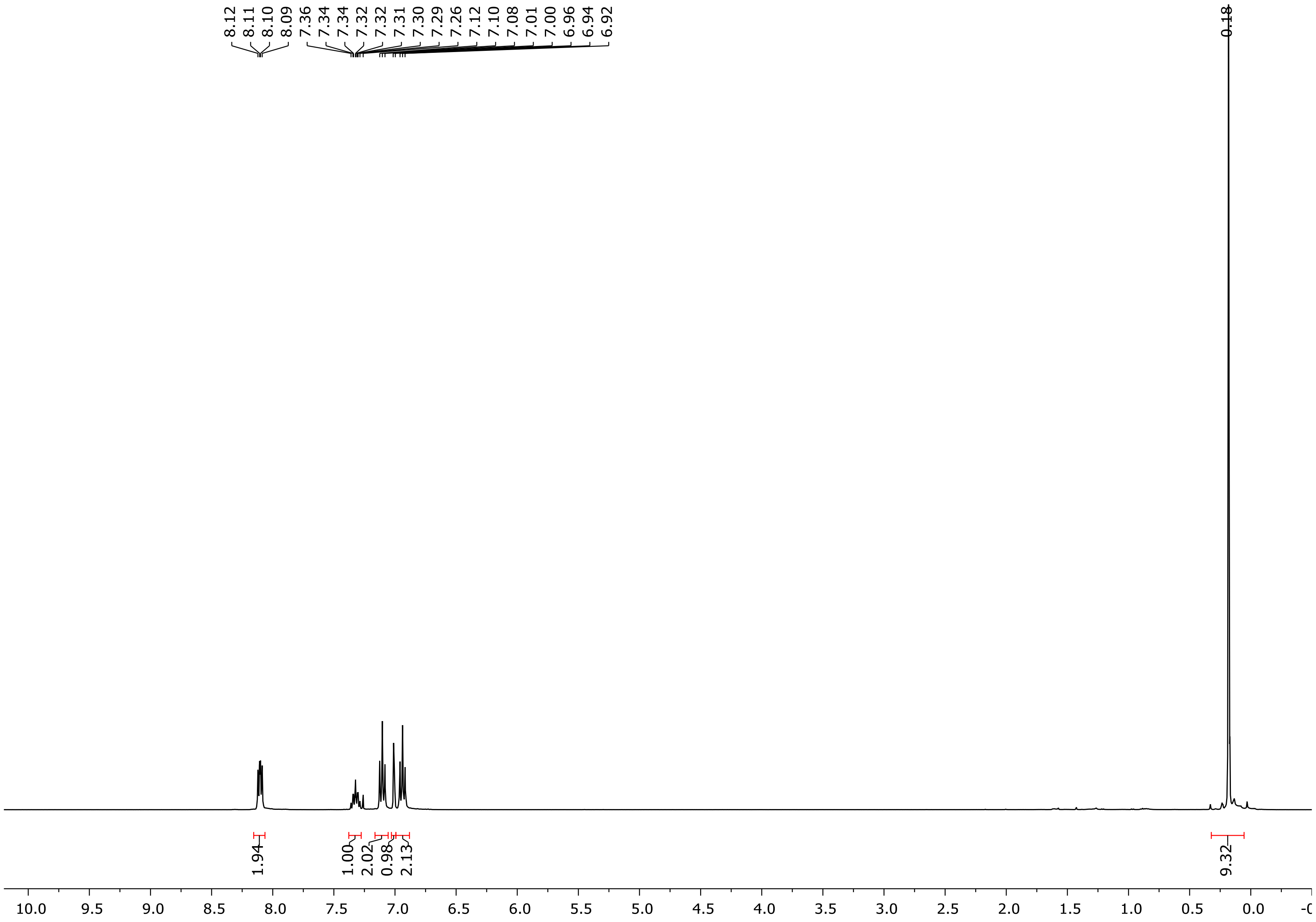

Figure S38: **^{13}C NMR** (101 MHz, $CDCl_3$, 298 K) spectrum of compound **2i**

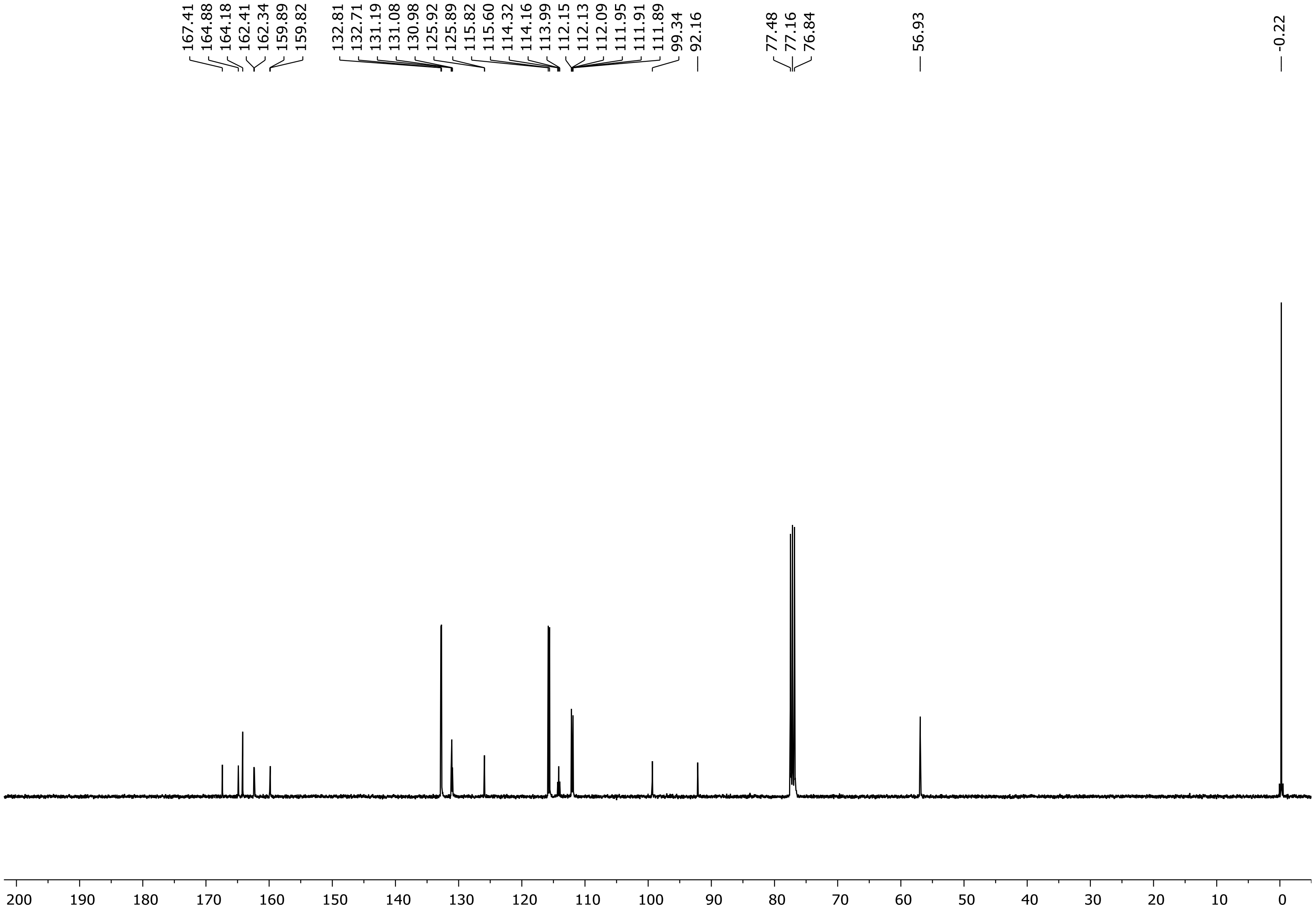

Figure S39: ^{19}**F NMR** (376 MHz, $CDCl_3$, 298 K) spectrum of compound **2i**

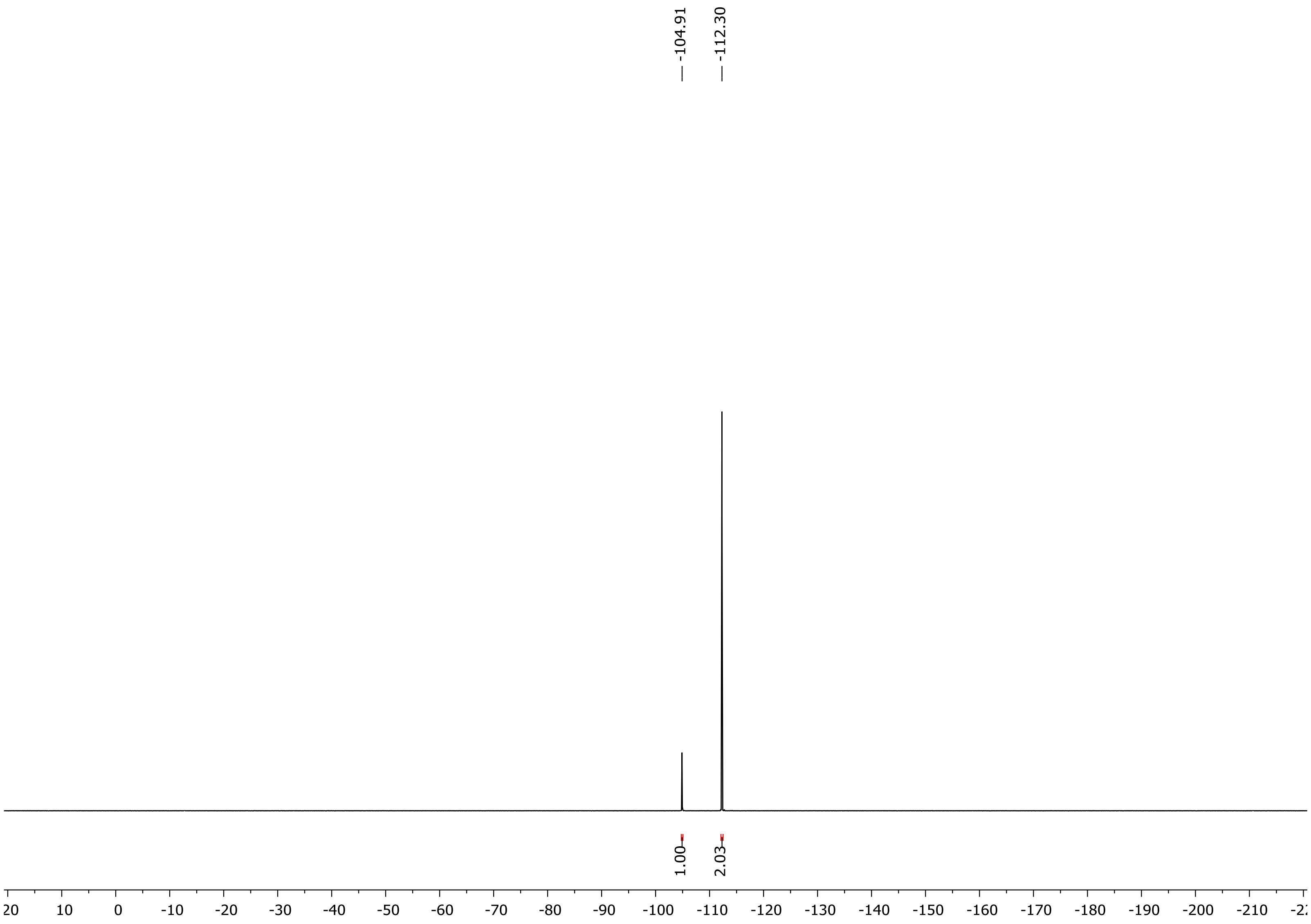

Figure S40: **^{1}H NMR** (500 MHz, $CDCl_3$, 298 K) spectrum of compound **2j**

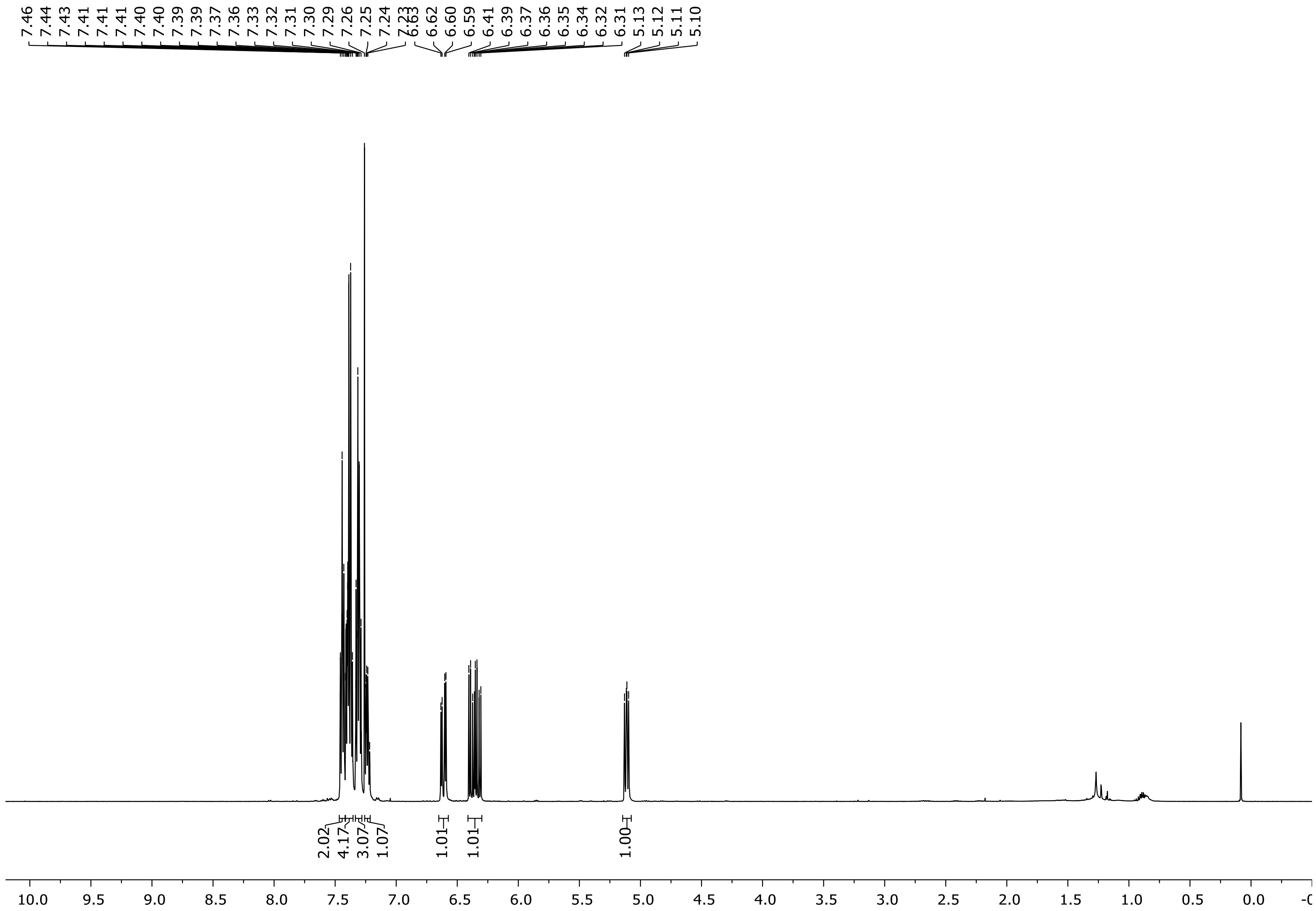

Figure S41: ^{13}**C NMR** (126 MHz, $CDCl_3$, 298 K) spectrum of compound **2j**

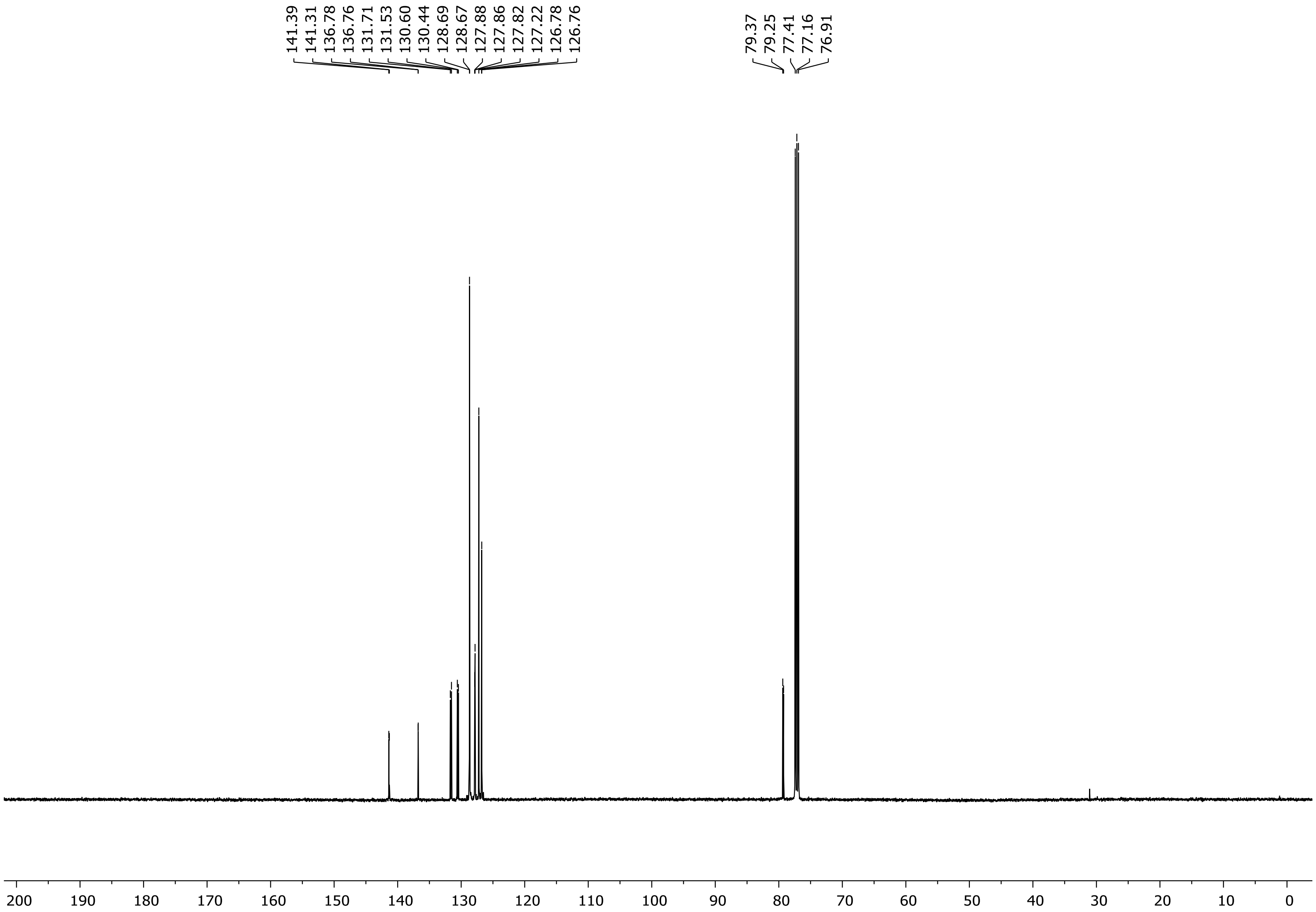

Figure S42: ^{19}**F NMR** (471 MHz, $CDCl_3$, 298 K) spectrum of compound **2j**

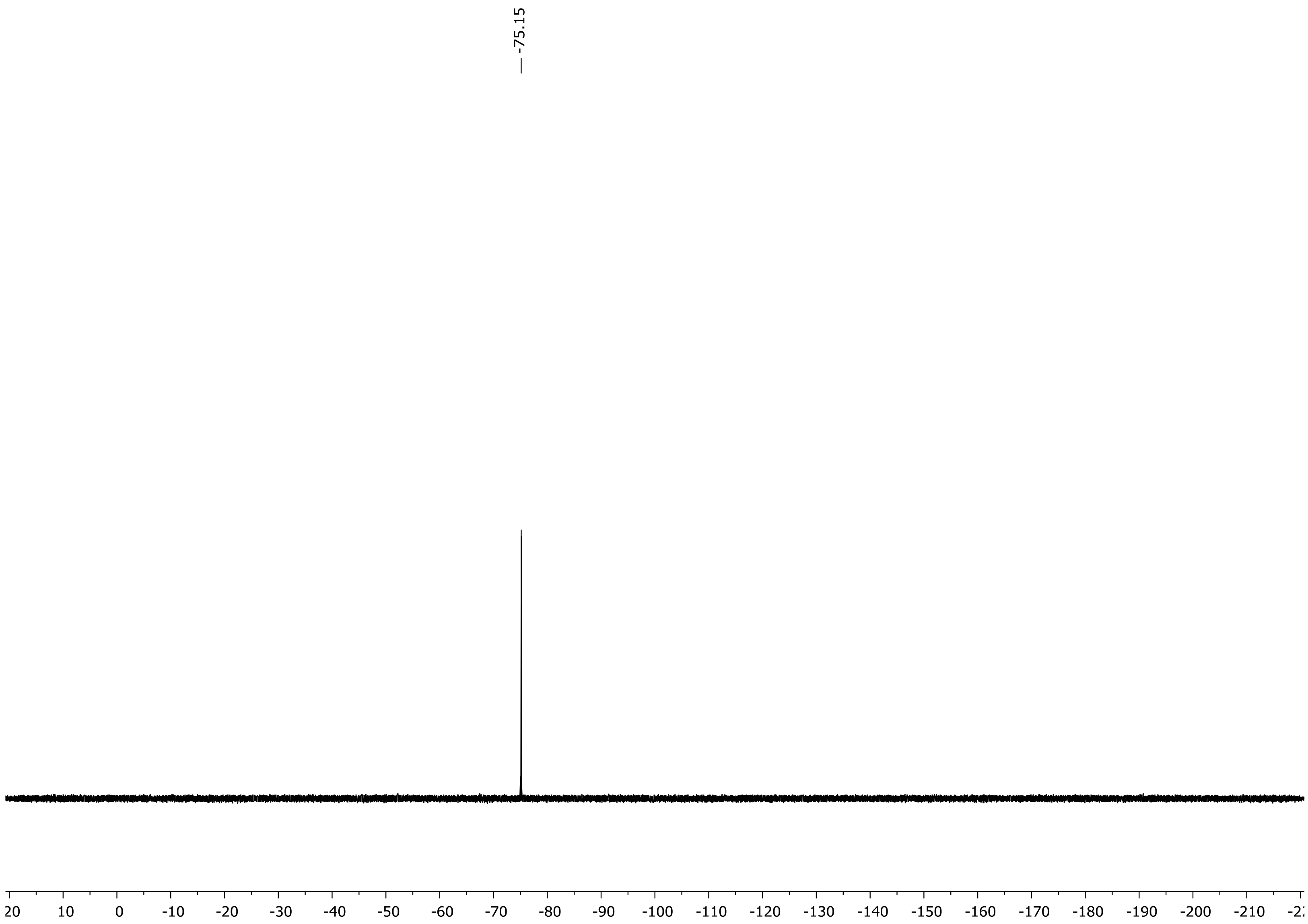

Figure S43: ^{1}**H NMR** (400 MHz, $CDCl_3$, 298 K) spectrum of compound **2k**

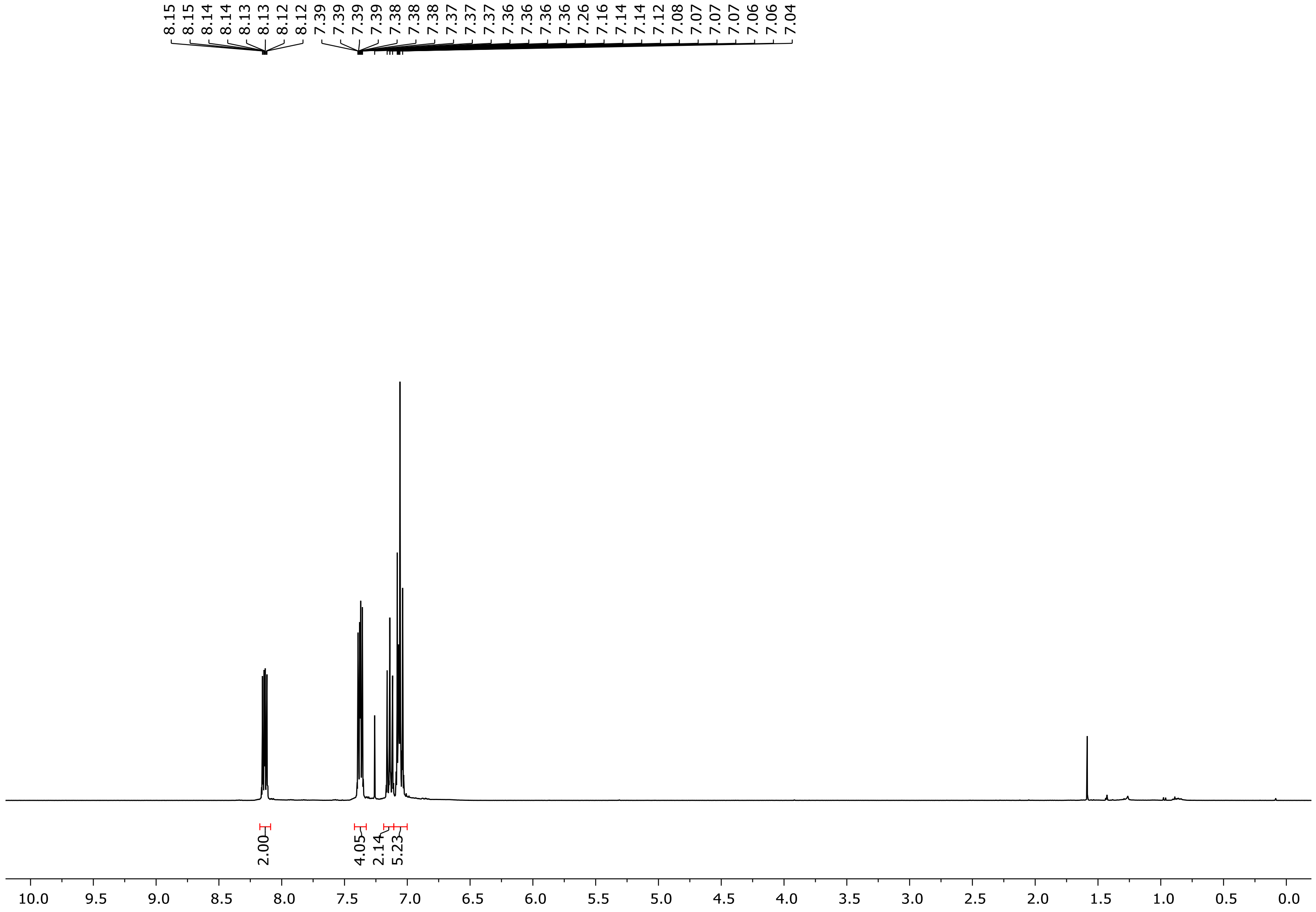

Figure S44: ^{13}**C NMR** (101 MHz, $CDCl_3$, 298 K) spectrum of compound **2k**

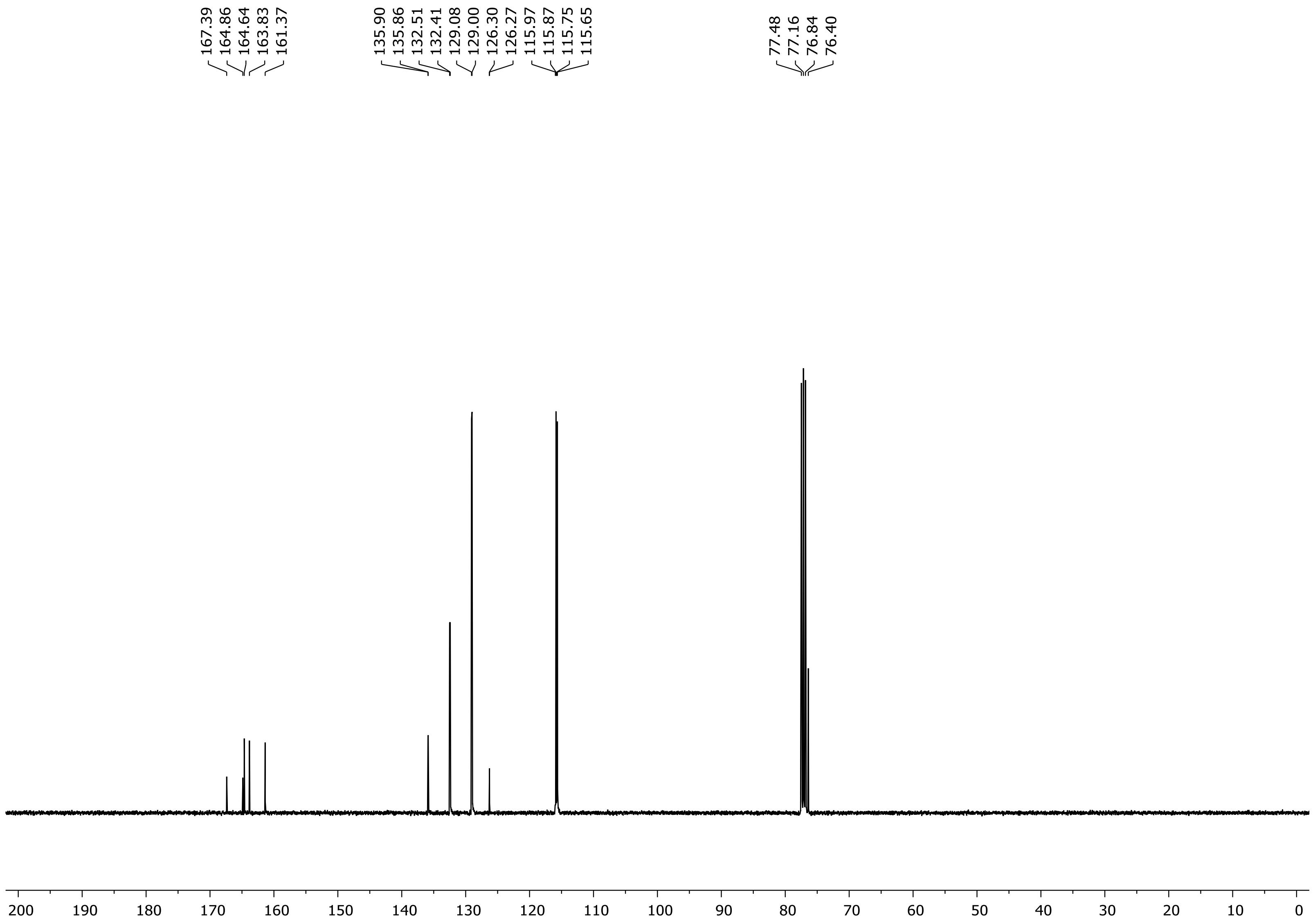

Figure S45: **^{19}F NMR** (376 MHz, $CDCl_3$, 298 K) spectrum of compound **2k**

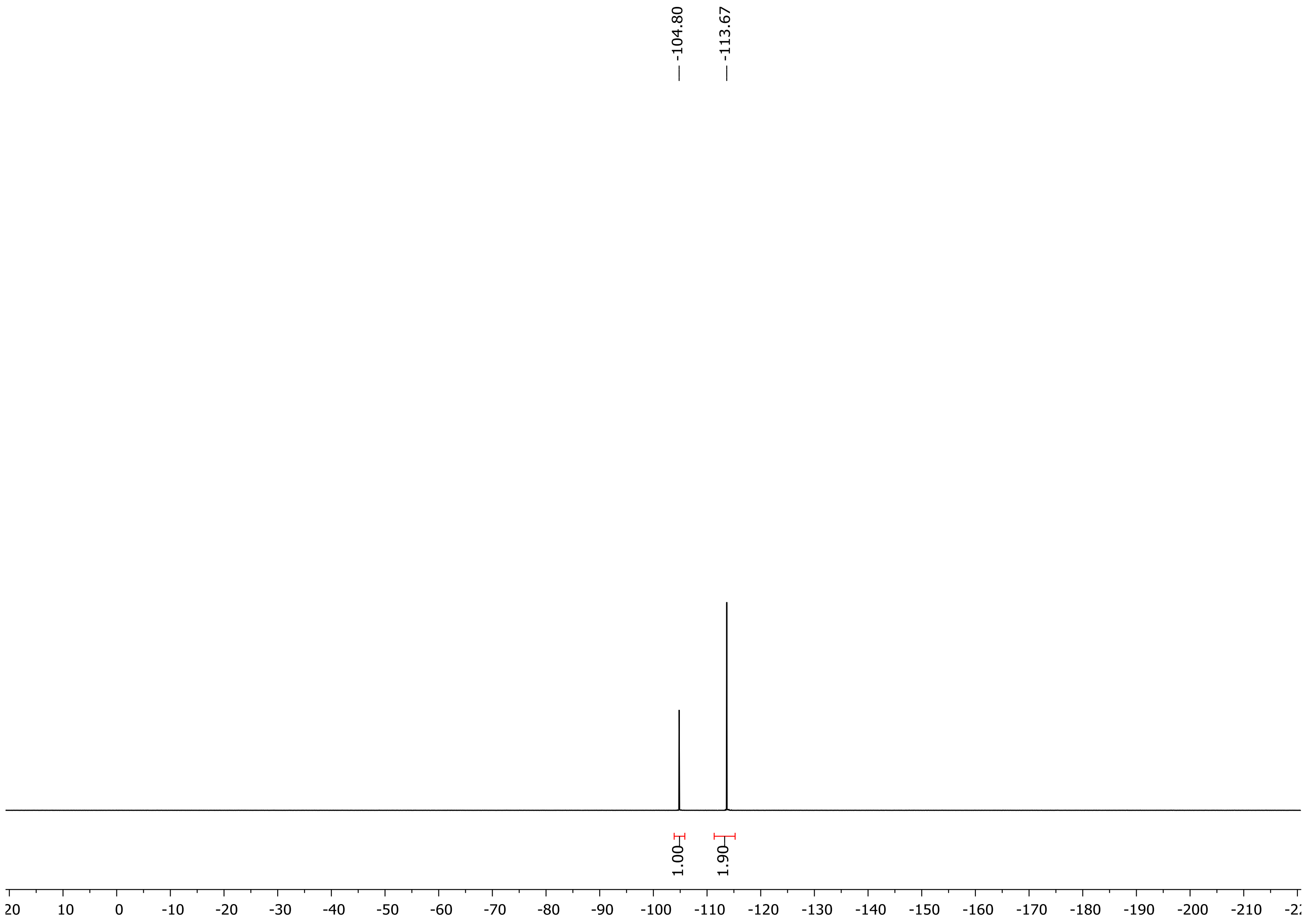

Figure S46: ^{1}**H NMR** (500 MHz, $CDCl_3$, 298 K) spectrum of compound **2l**

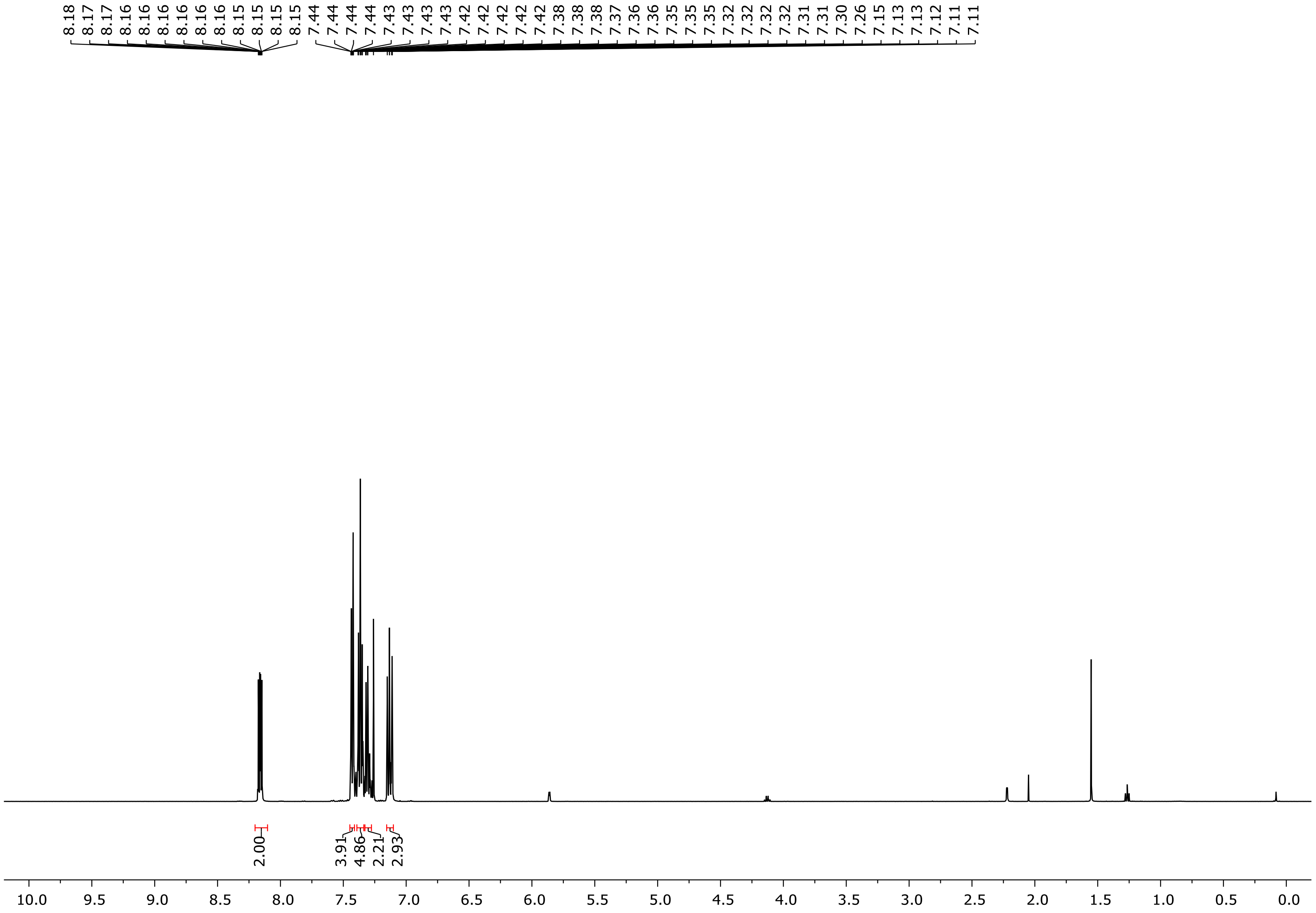

Figure S47: **^{13}C NMR** (126 MHz, $CDCl_3$, 298 K) spectrum of compound **2l**

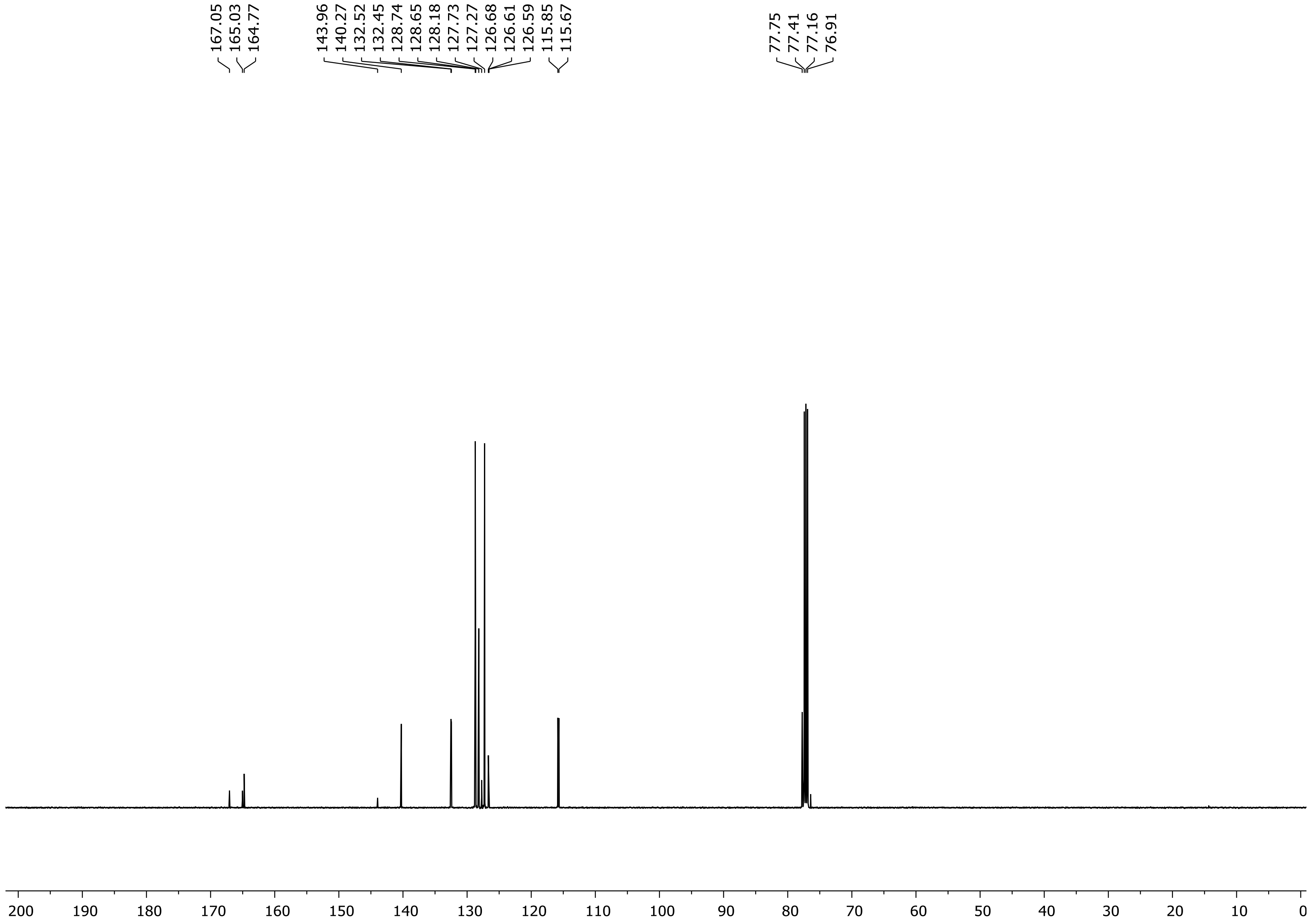

Figure S48: **^{19}F NMR** (471 MHz, $CDCl_3$, 298 K) spectrum of compound **2l**

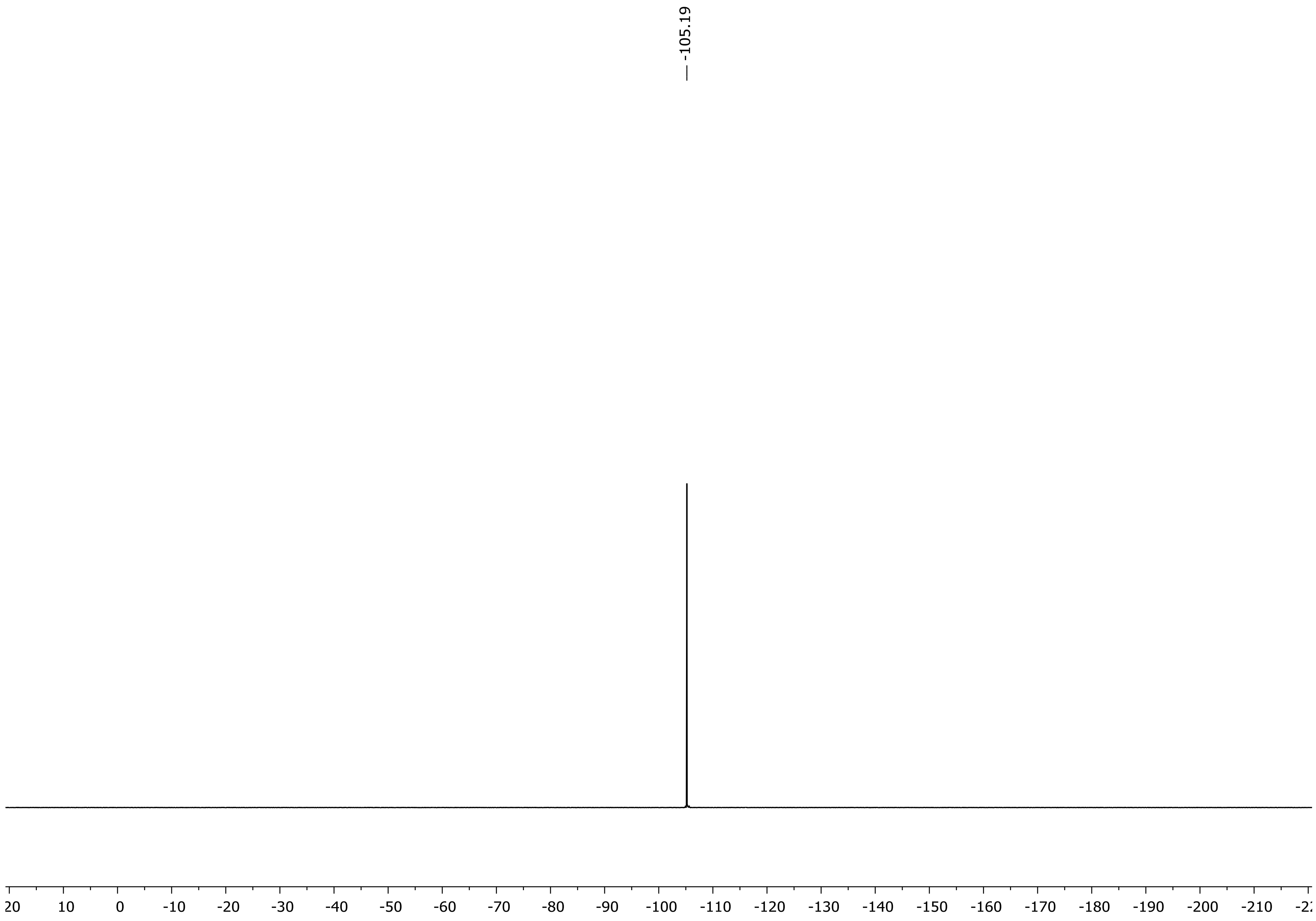

Figure S49: **^{1}H NMR** (500 MHz, $CDCl_3$, 298 K) spectrum of compound **2m**

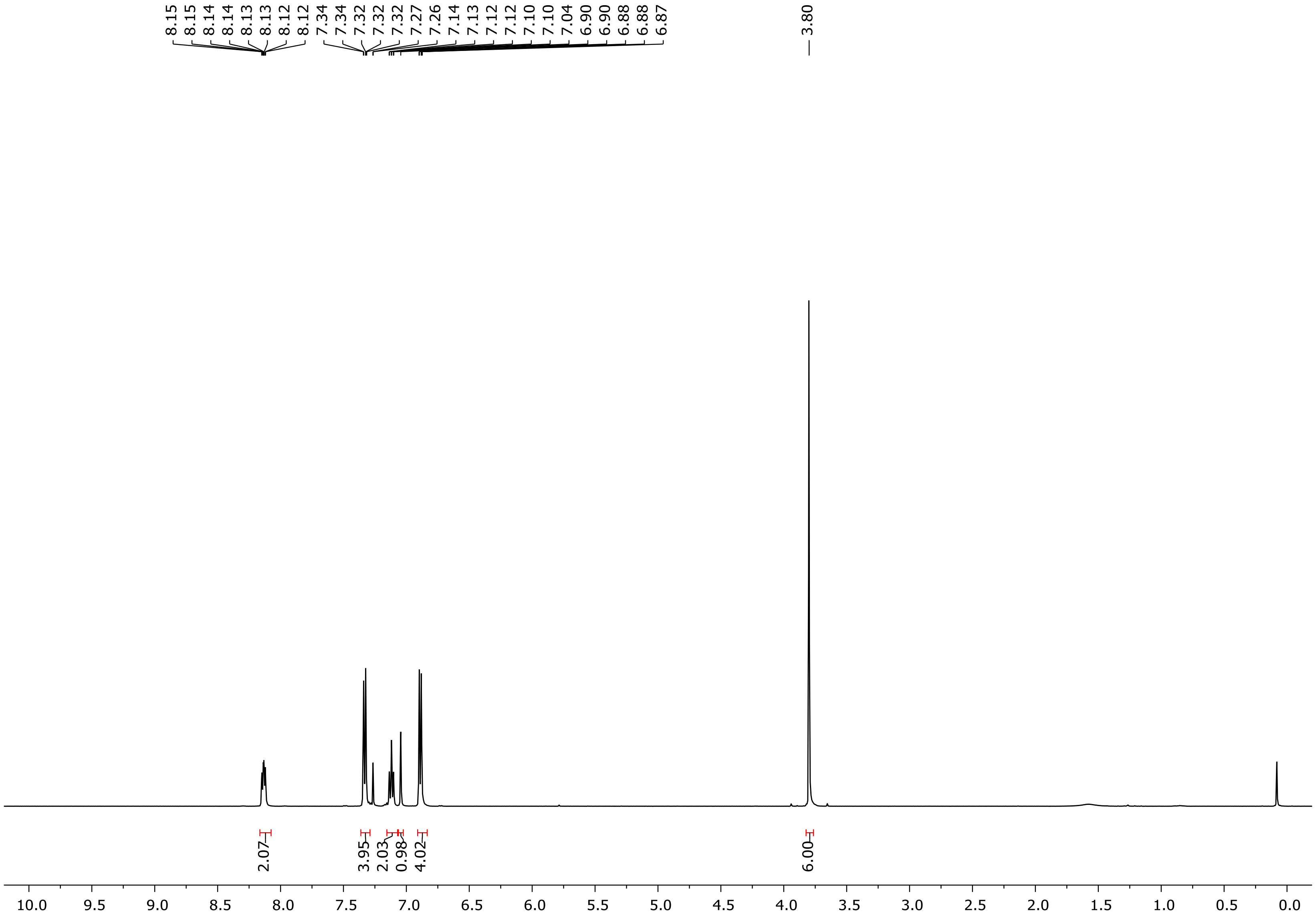

Figure S50: ^{13}**C NMR** (126 MHz, $CDCl_3$, 298 K) spectrum of compound **2m**

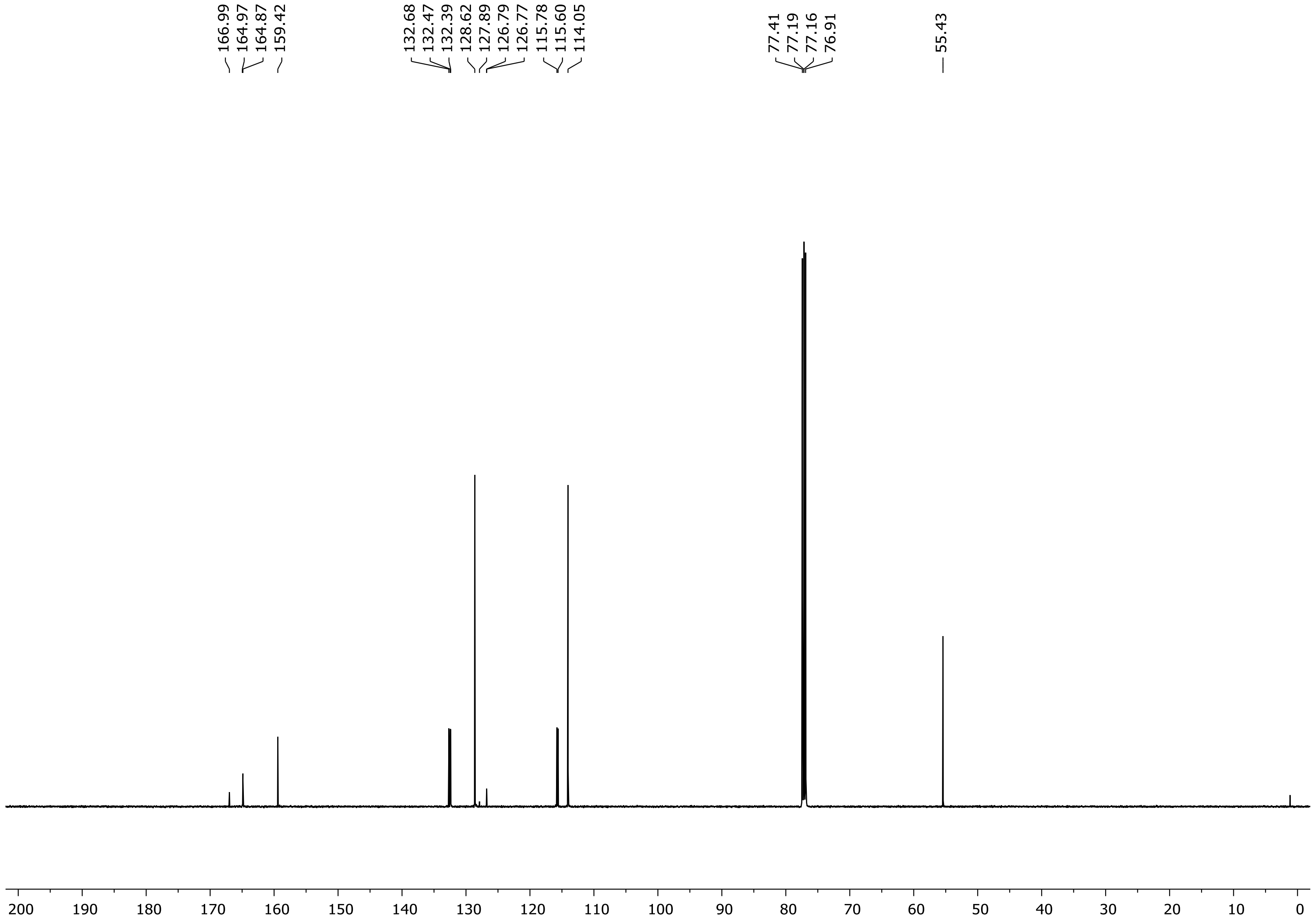

Figure S51: **^{19}F NMR** (471 MHz, $CDCl_3$, 298 K) spectrum of compound **2m**

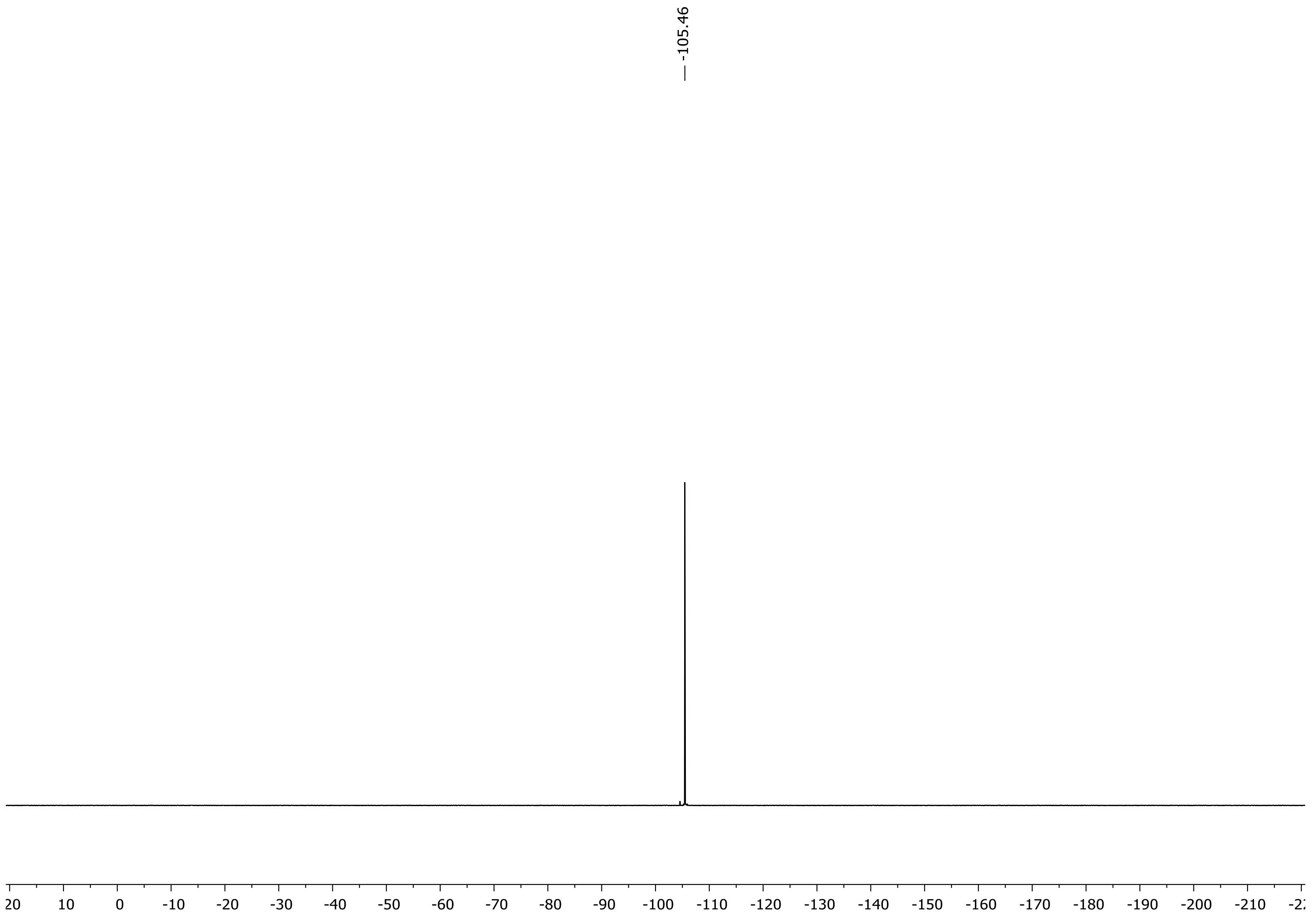

Figure S52: **^{1}H NMR** (500 MHz, $CDCl_3$, 298 K) spectrum of compound **2n**

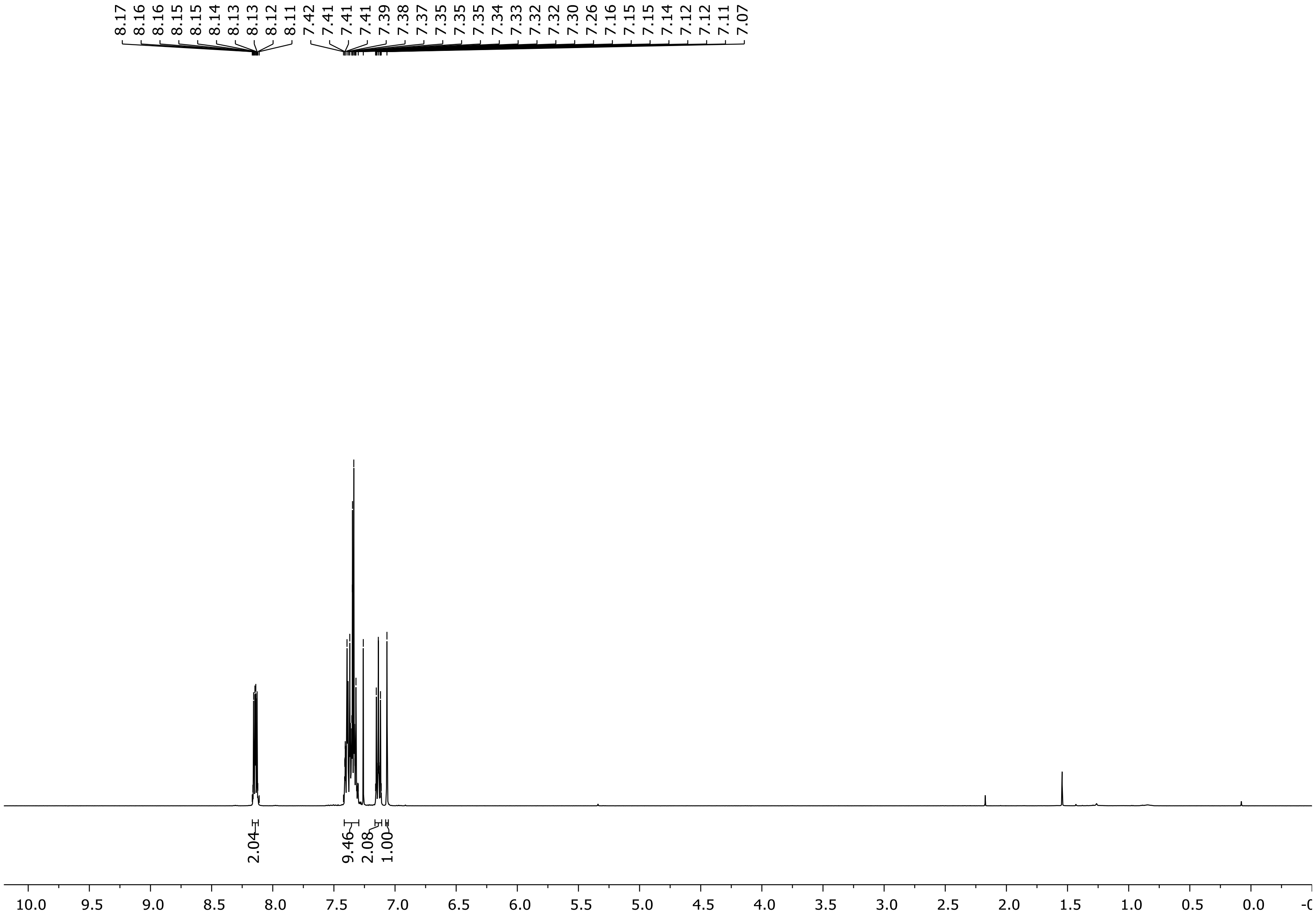

Figure S53: **^{13}C NMR** (126 MHz, $CDCl_3$, 298 K) spectrum of compound **2n**

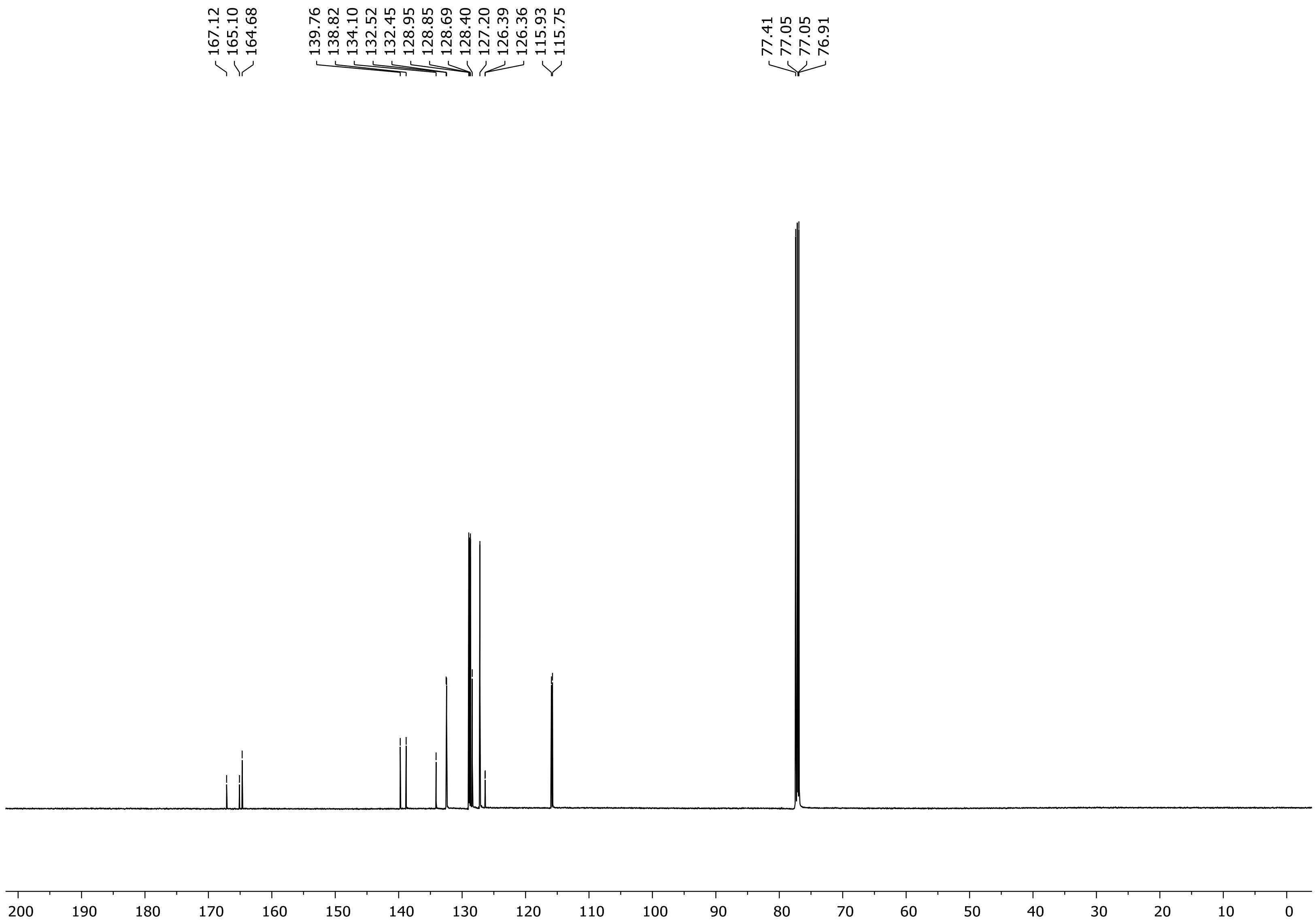

Figure S54: **^{19}F NMR** (471 MHz, $CDCl_3$, 298 K) spectrum of compound **2n**

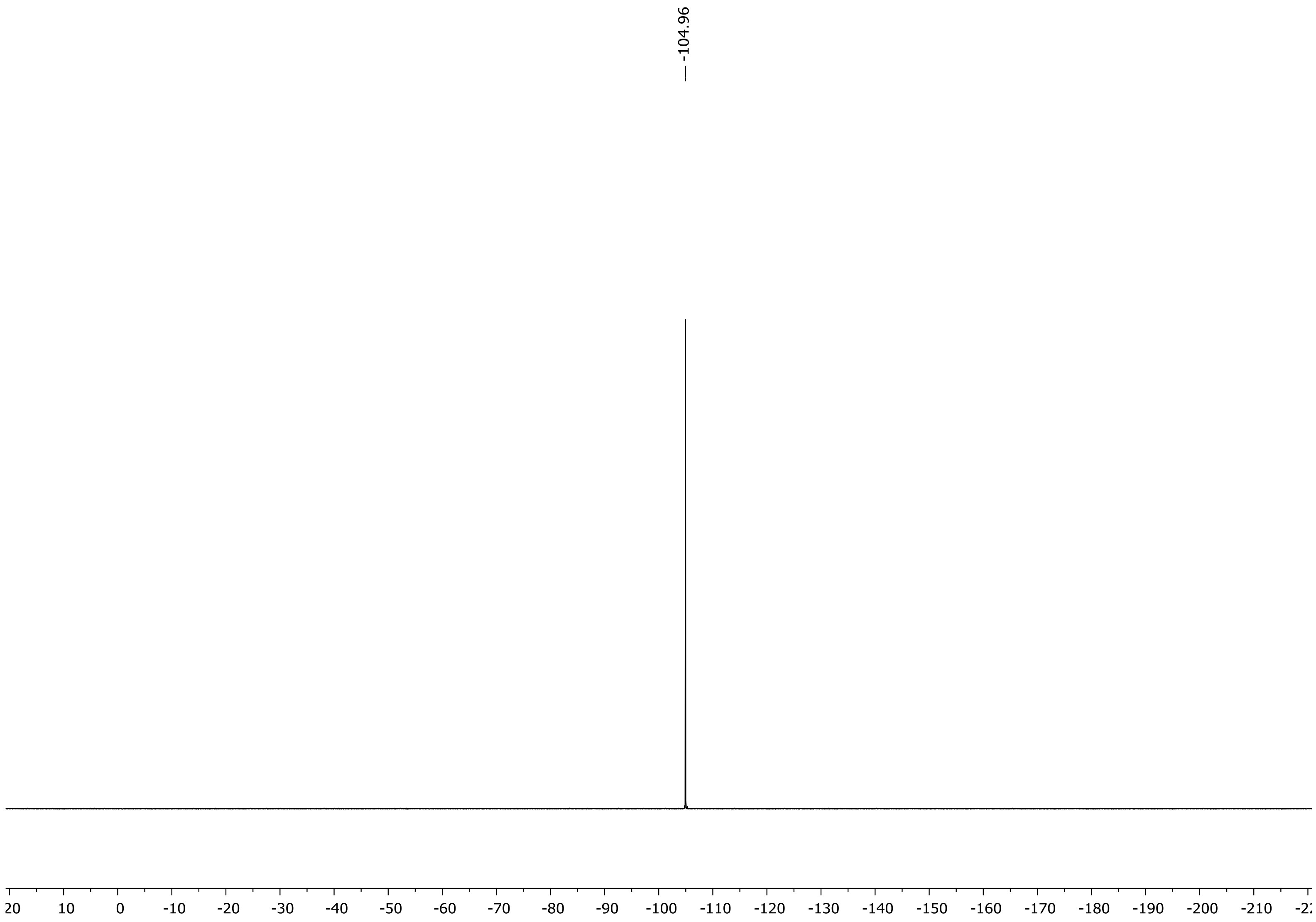

Figure S55: **^{1}H NMR** (500 MHz, $CDCl_3$, 298 K) spectrum of compound **2o**

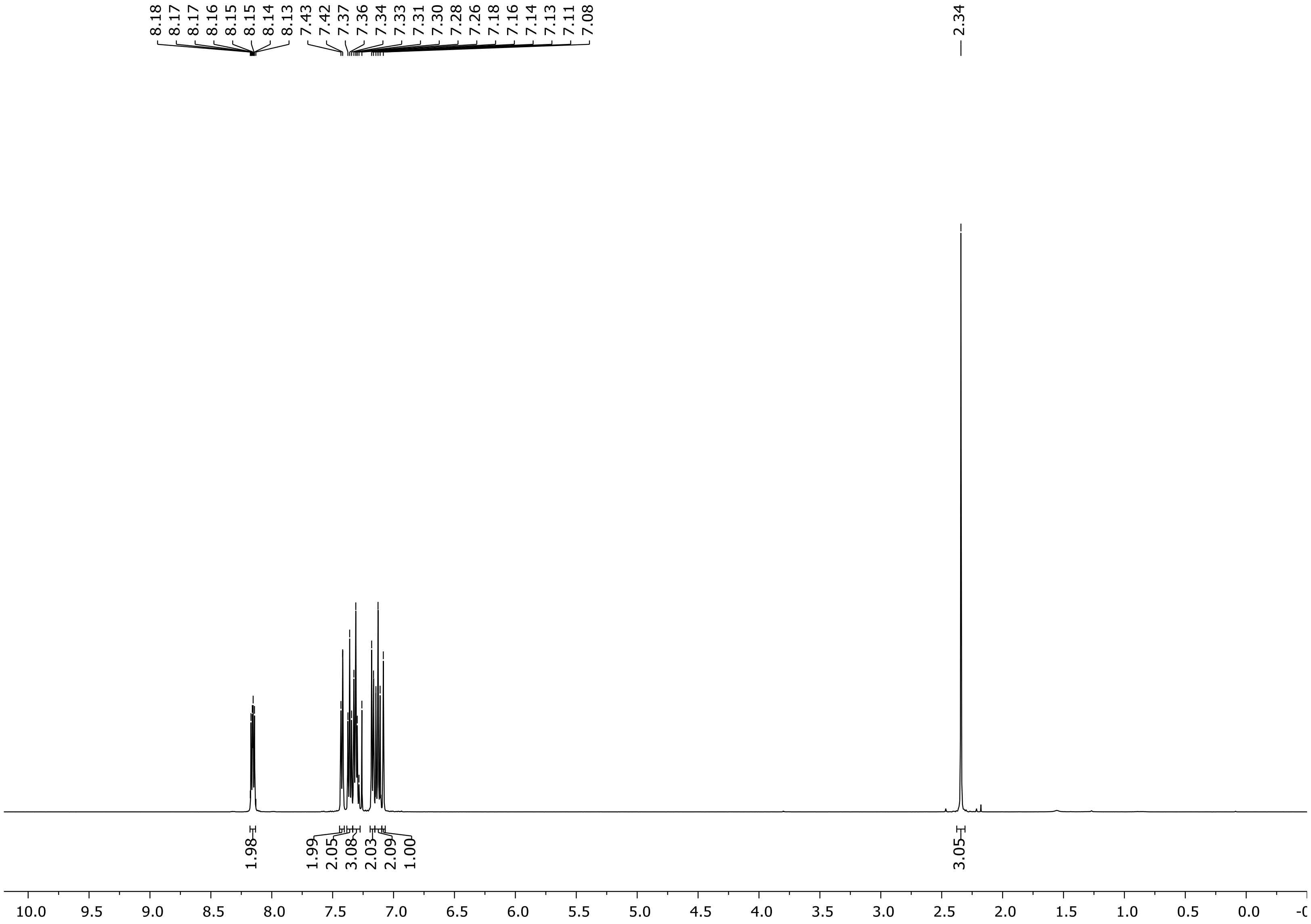

Figure S56: **13C NMR** (126 MHz, $CDCl_3$, 298 K) spectrum of compound **2o**

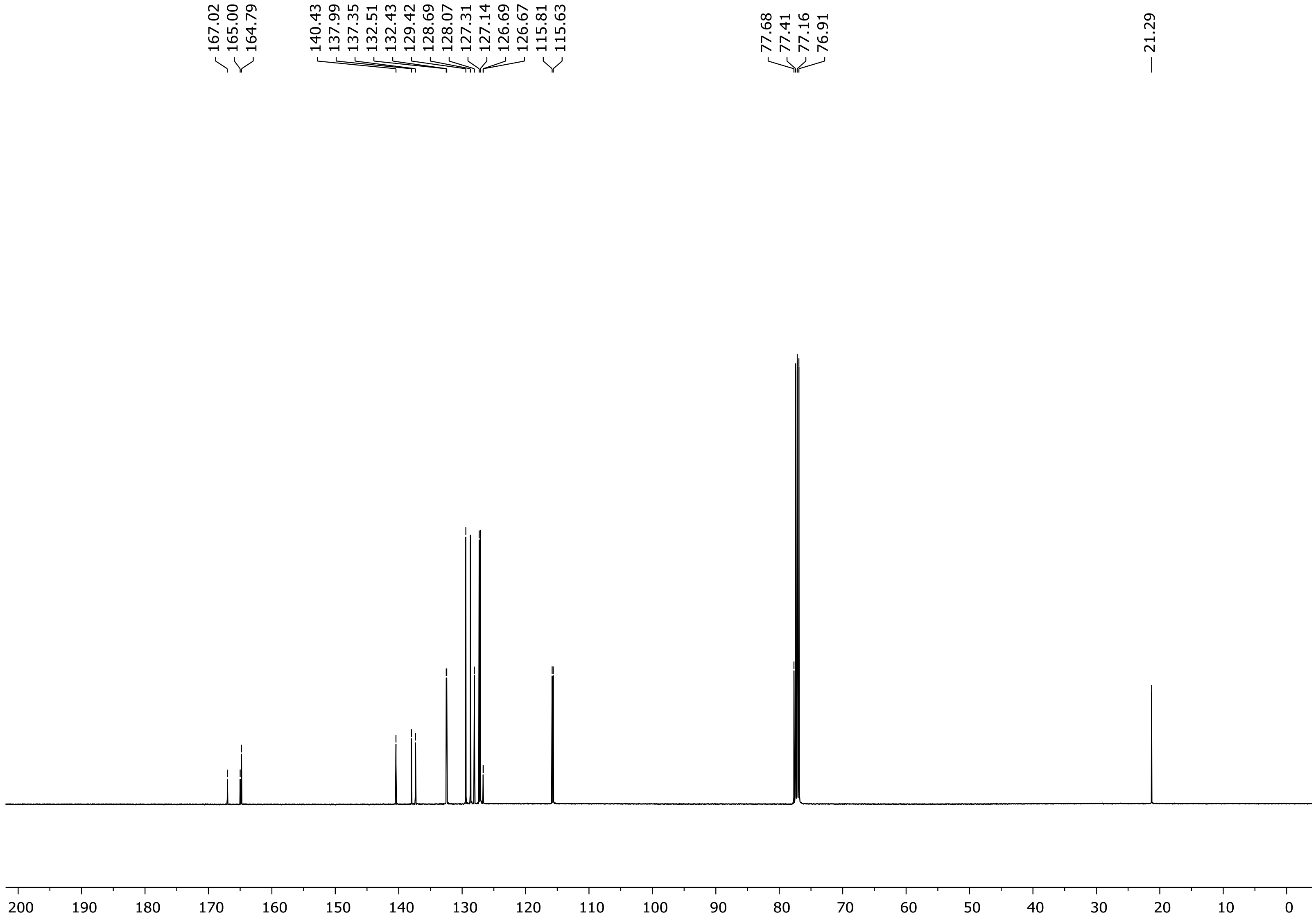

Figure S57: **^{19}F NMR** (471 MHz, $CDCl_3$, 298 K) spectrum of compound **2o**

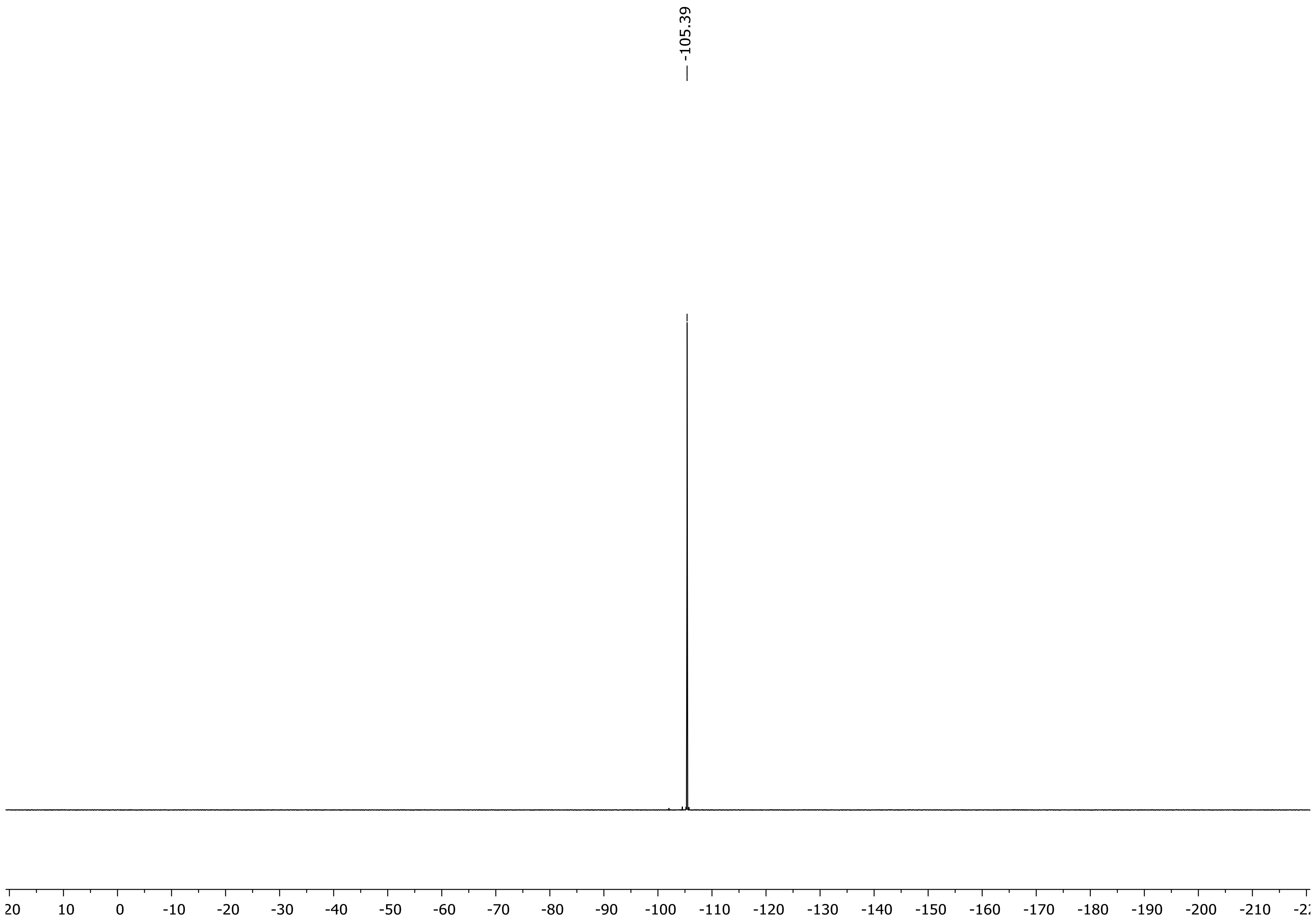

Figure S58: **^{1}H NMR** (500 MHz, $CDCl_3$, 298 K) spectrum of compound **2p**

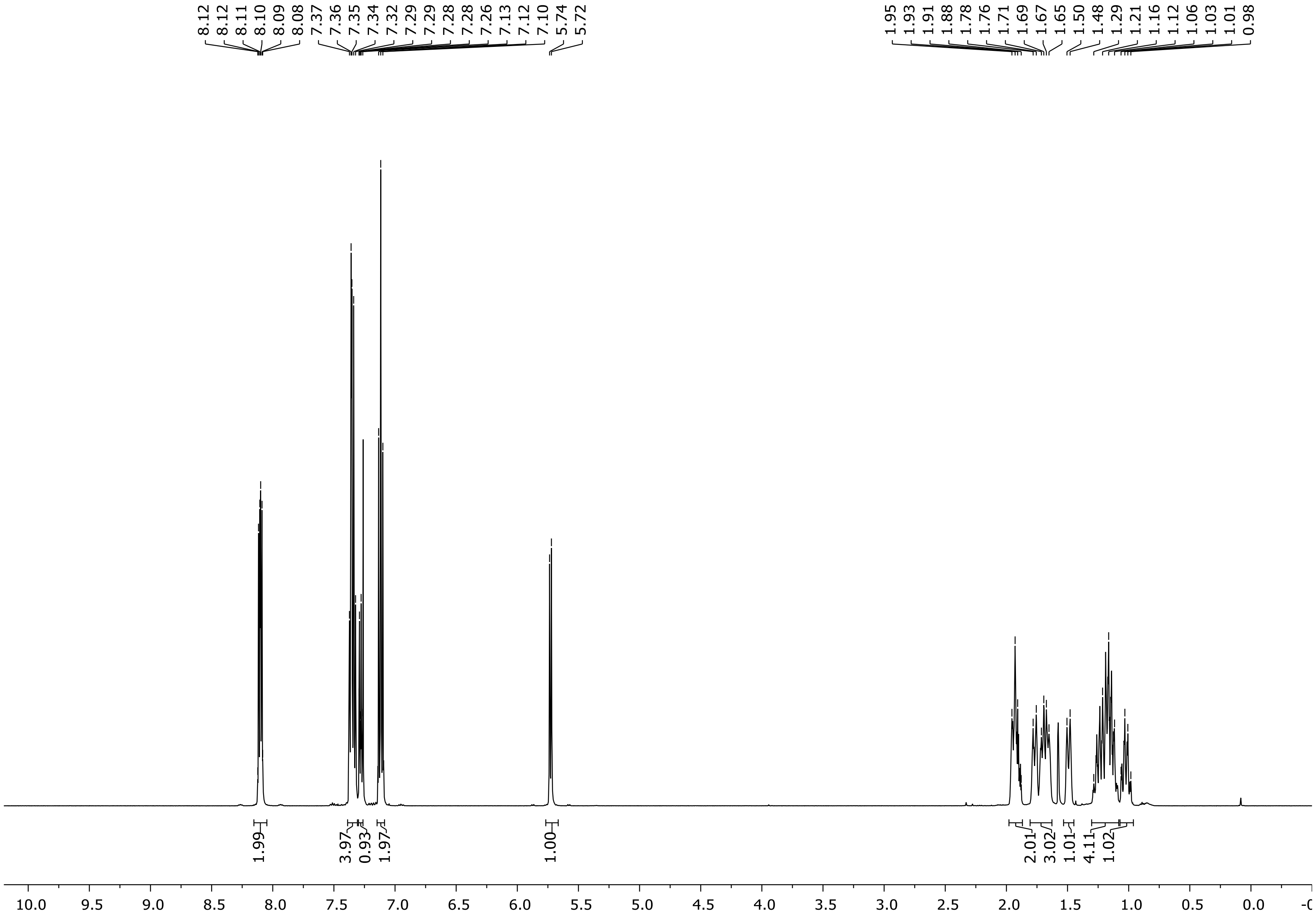

Figure S59: **^{13}C NMR** (126 MHz, $CDCl_3$, 298 K) spectrum of compound **2p**

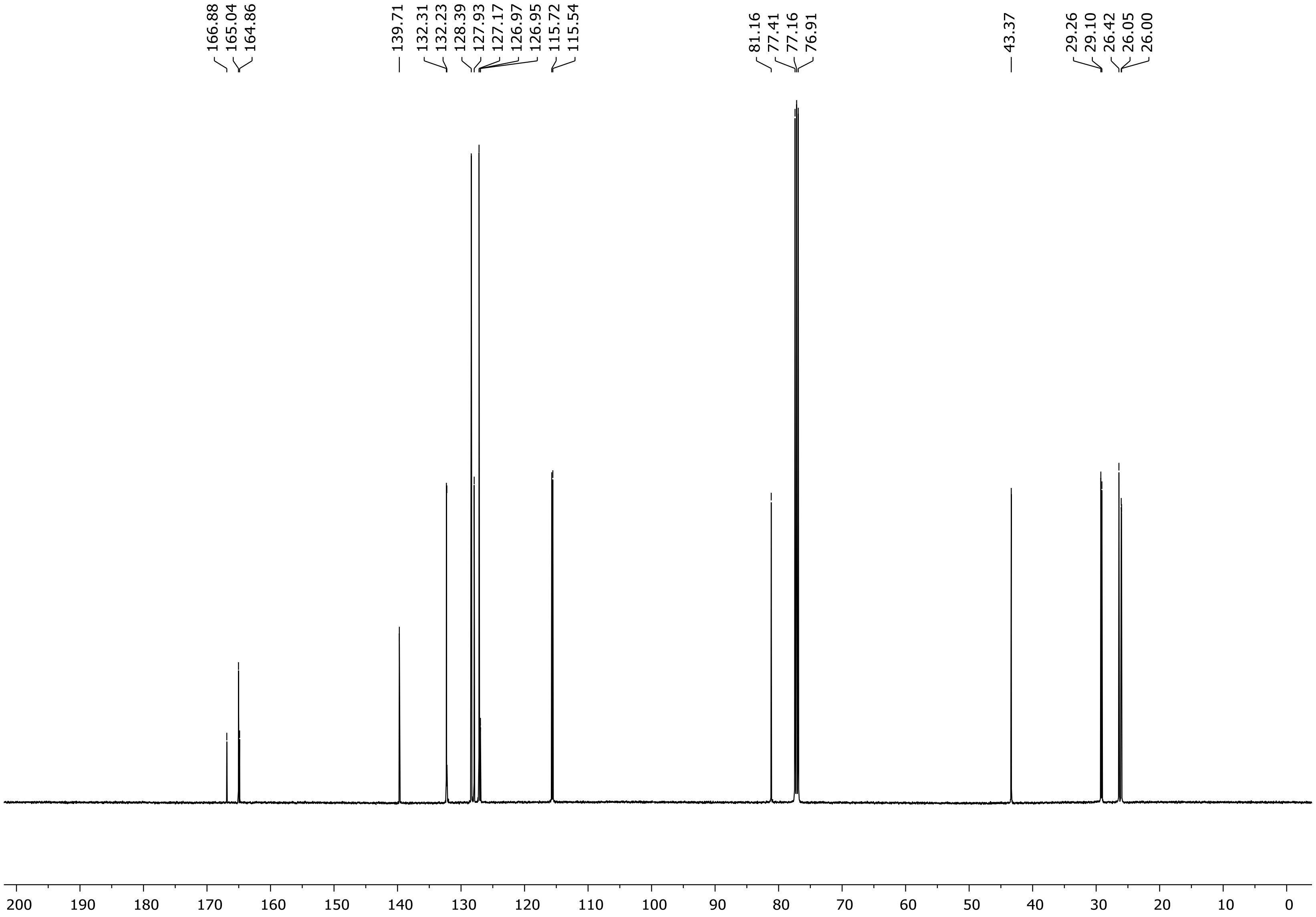

Figure S60: **^{19}F NMR** (471 MHz, $CDCl_3$, 298 K) spectrum of compound **2p**

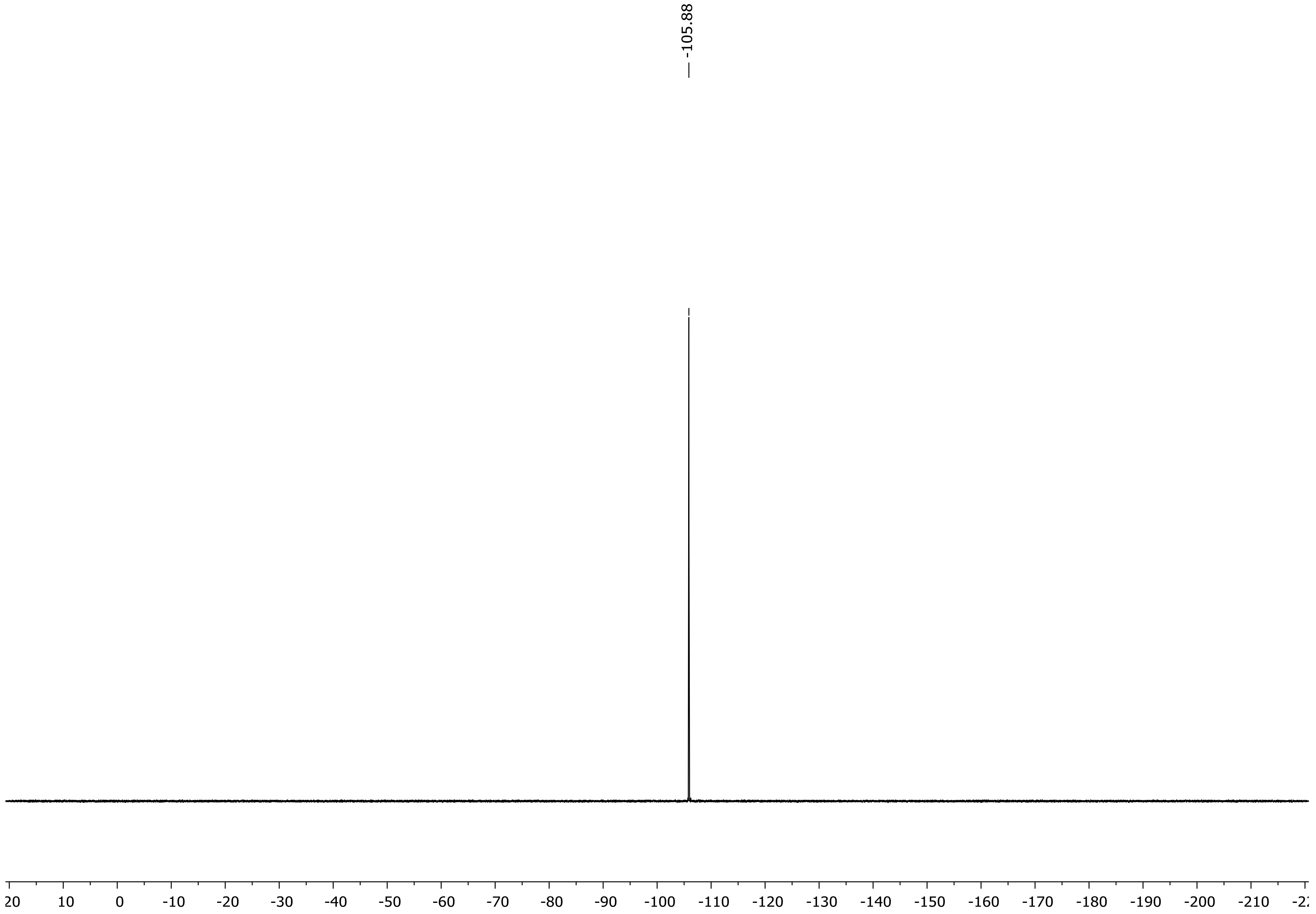

Figure S61: [1]**H NMR** (400 MHz, $CDCl_3$, 298 K) spectrum of compound **3a**

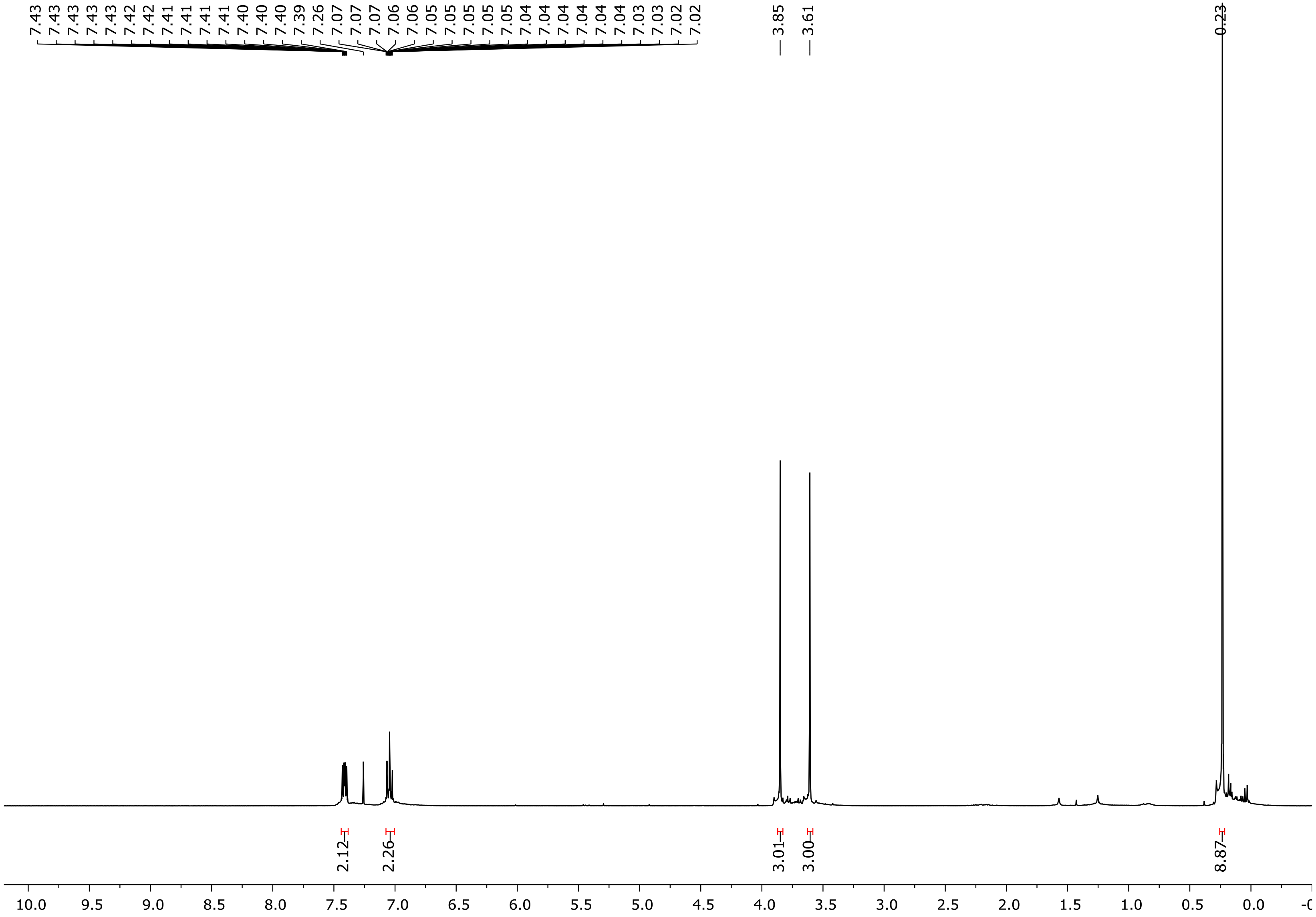

Figure S62: **^{13}C NMR** (101 MHz, $CDCl_3$, 298 K) spectrum of compound **3a**

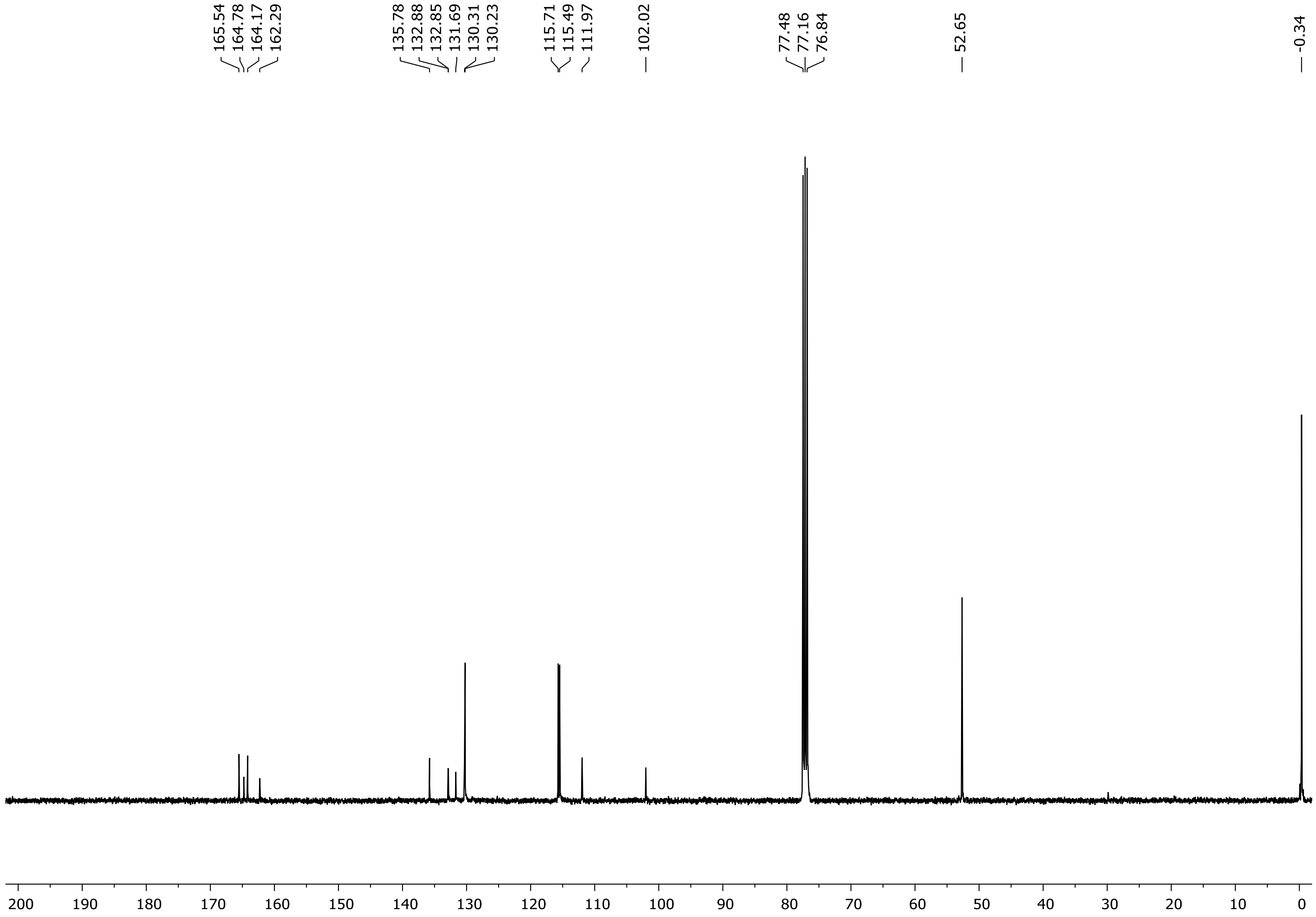

Figure S63: **^{19}F NMR** (376 MHz, $CDCl_3$, 298 K) spectrum of compound **3a**

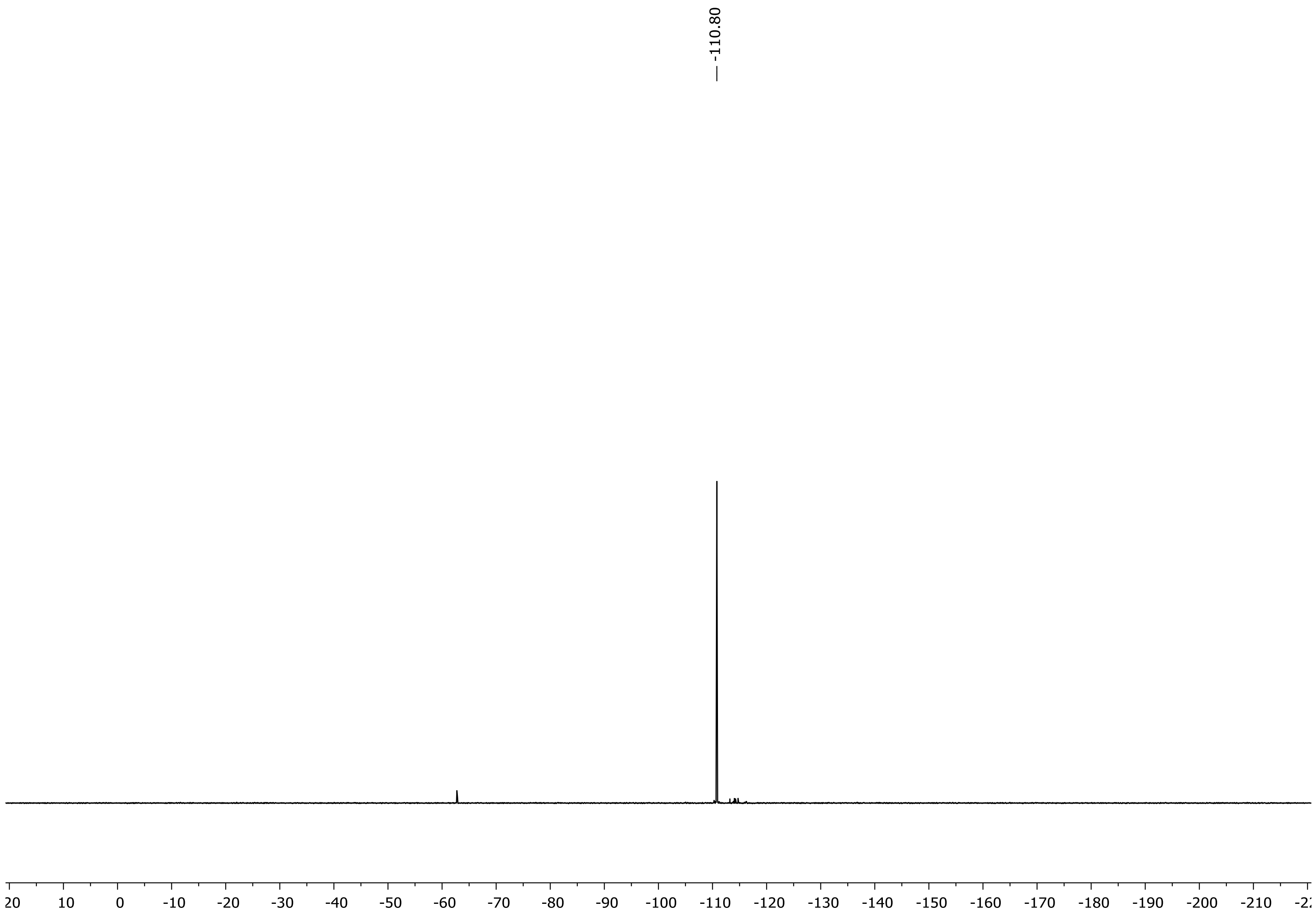

Figure S64: **^{1}H NMR** (400 MHz, $CDCl_3$, 298 K) spectrum of compound **3b**

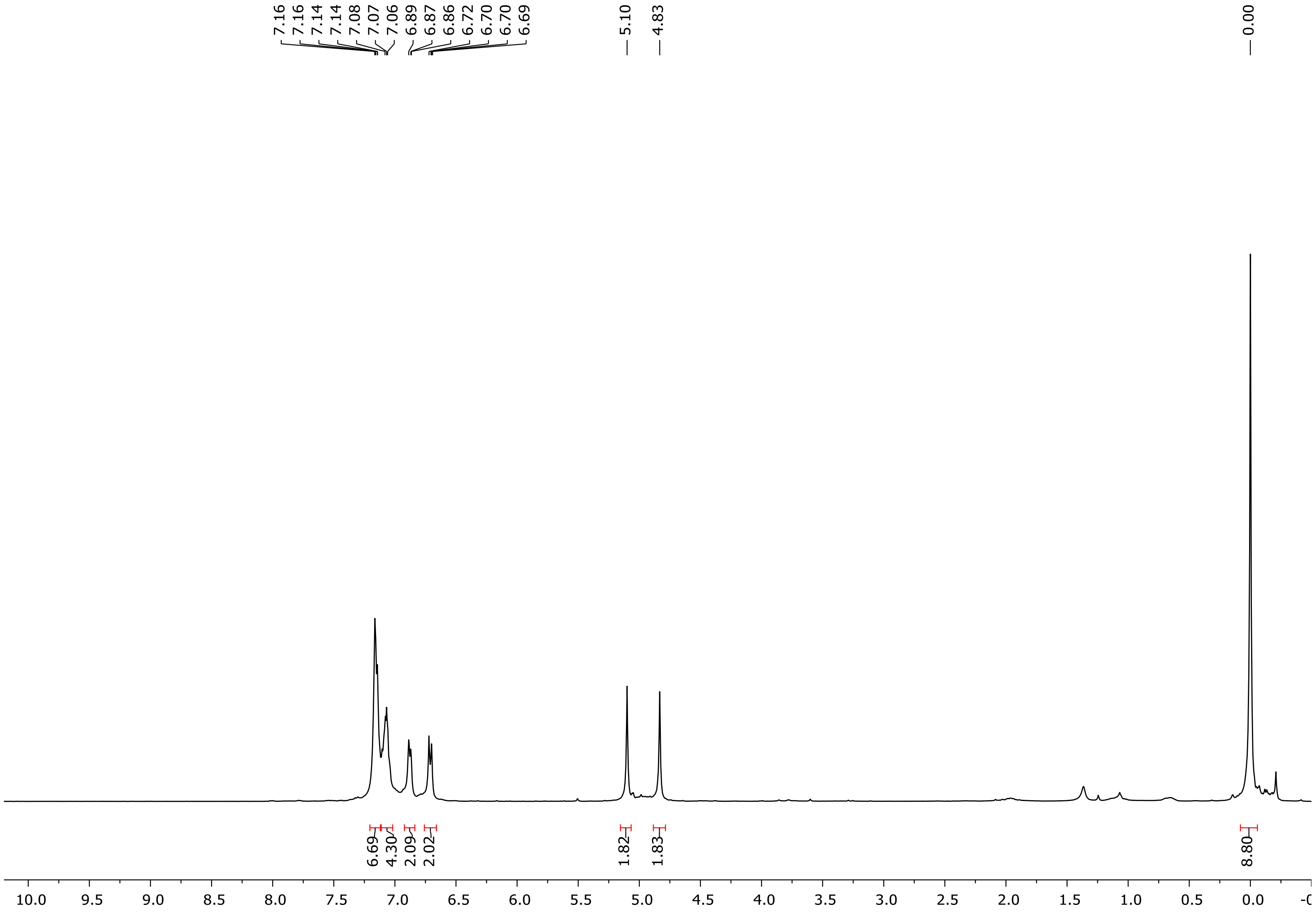

Figure S65: **^{13}C NMR** (101 MHz, $CDCl_3$, 298 K) spectrum of compound **3b**

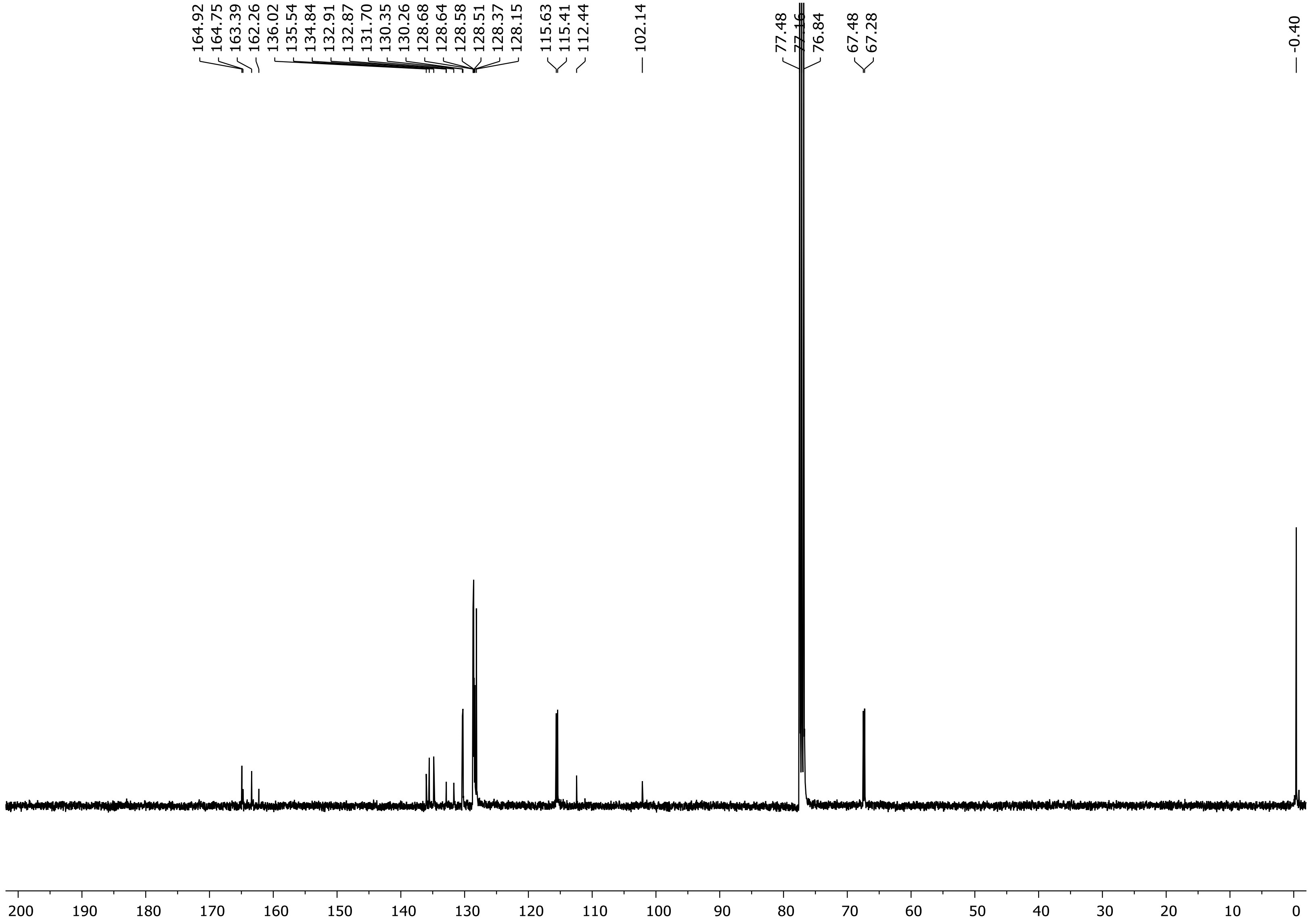

Figure S66: **^{19}F NMR** (376 MHz, $CDCl_3$, 298 K) spectrum of compound **3b**

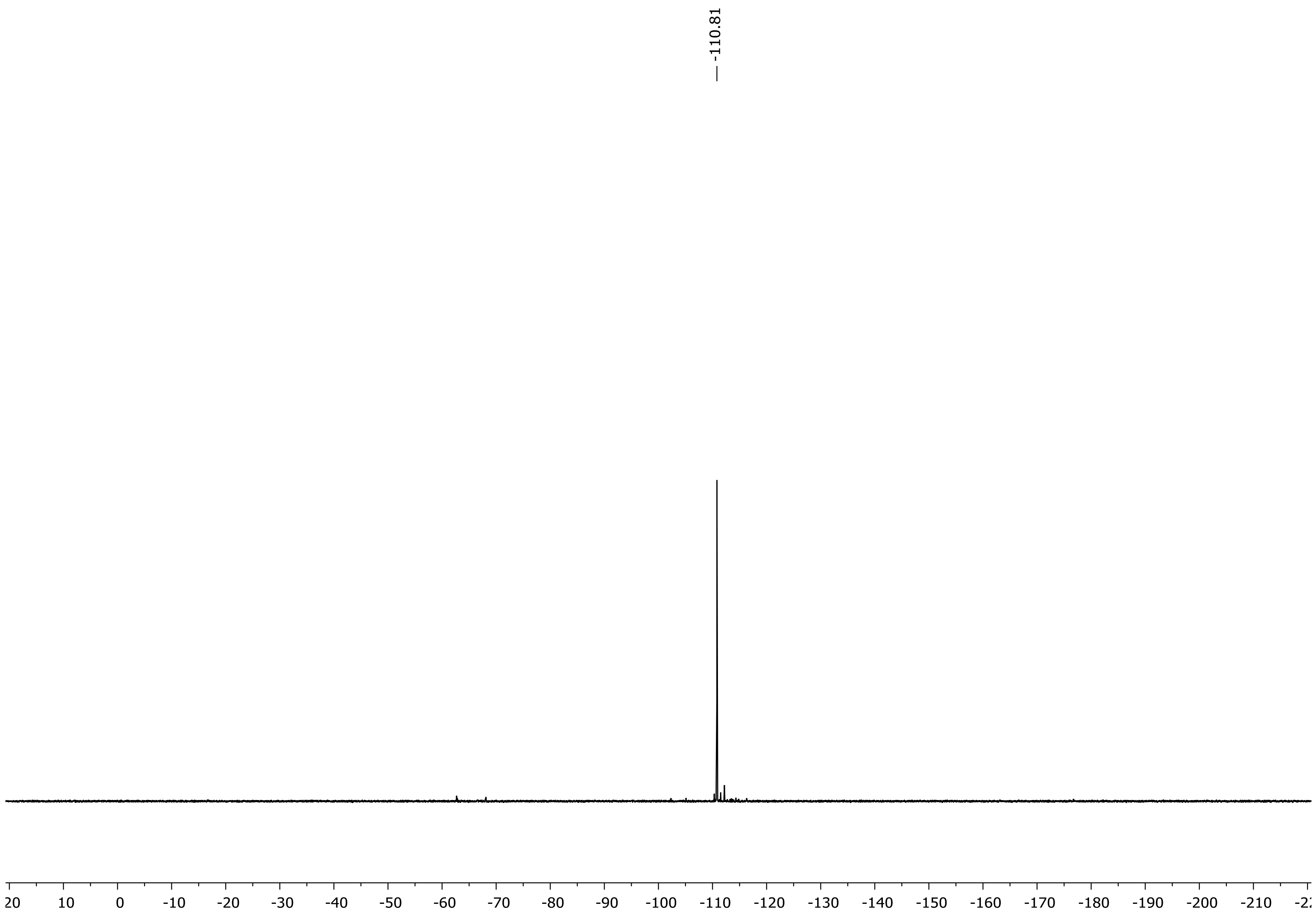

Figure S67: **^{1}H NMR** (500 MHz, $CDCl_3$, 298 K) spectrum of compound **3c**

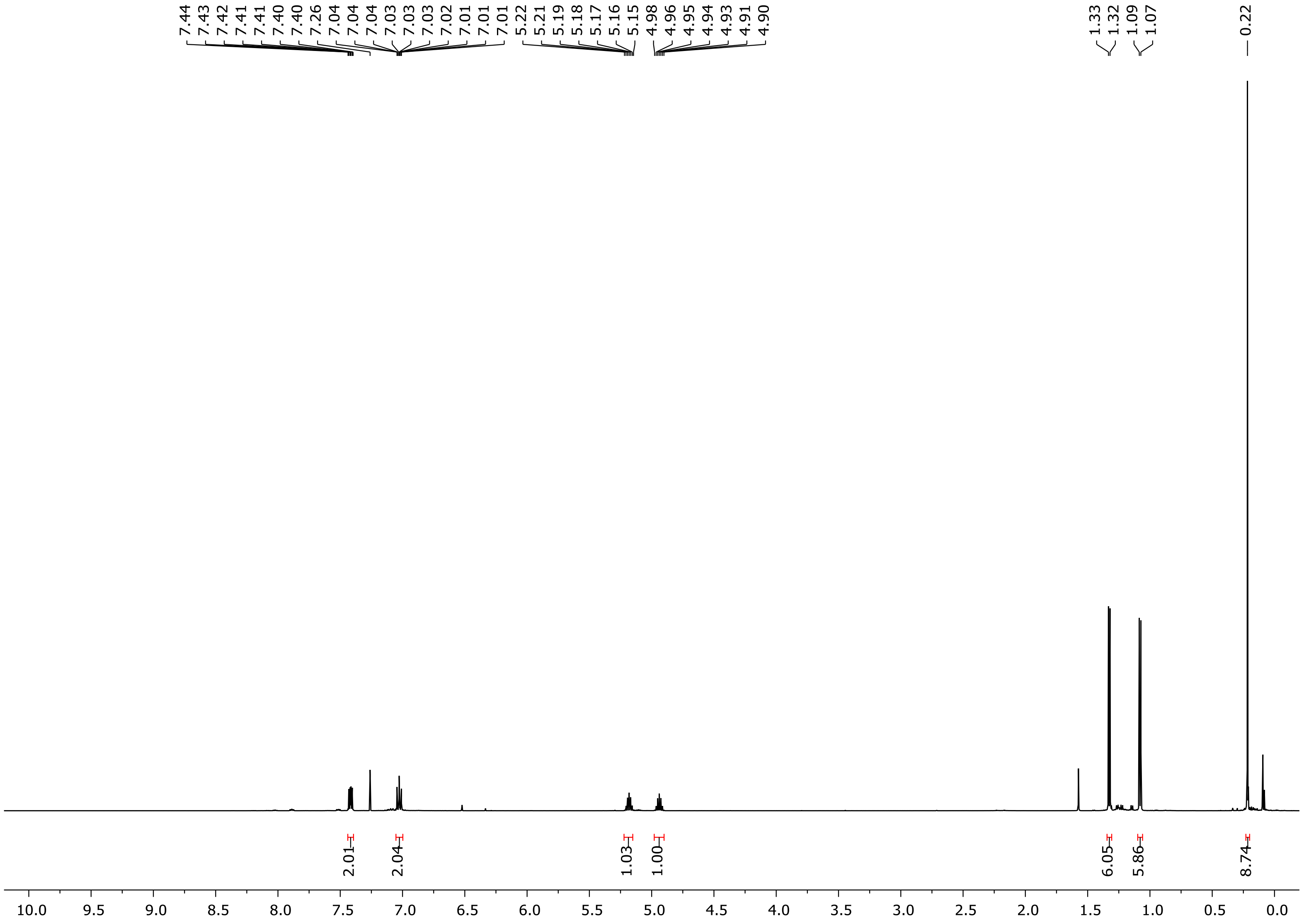

Figure S68: **^{13}C NMR** (126 MHz, $CDCl_3$, 298 K) spectrum of compound **3c**

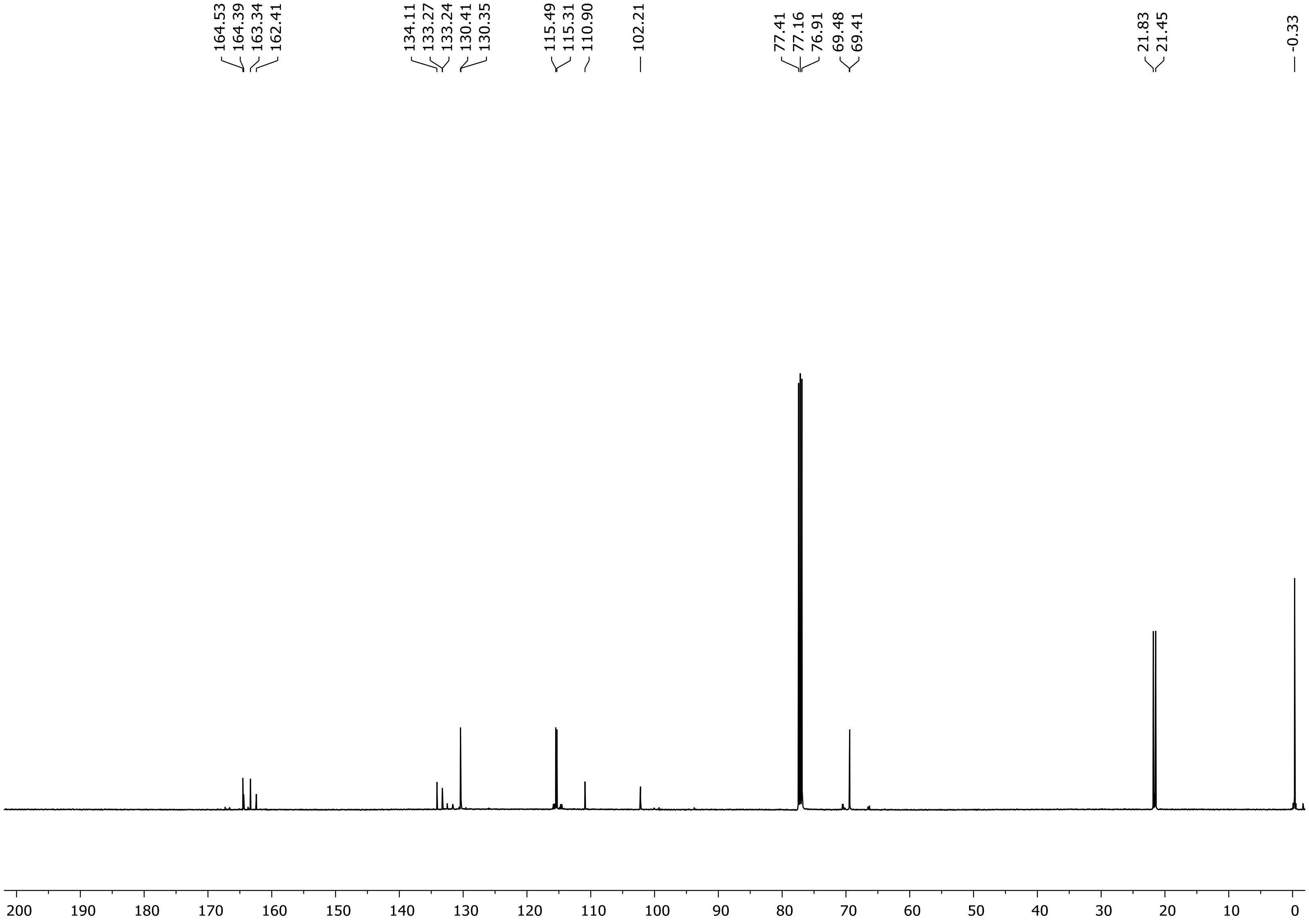

Figure S69: **^{19}F NMR** (471 MHz, $CDCl_3$, 298 K) spectrum of compound **3c**

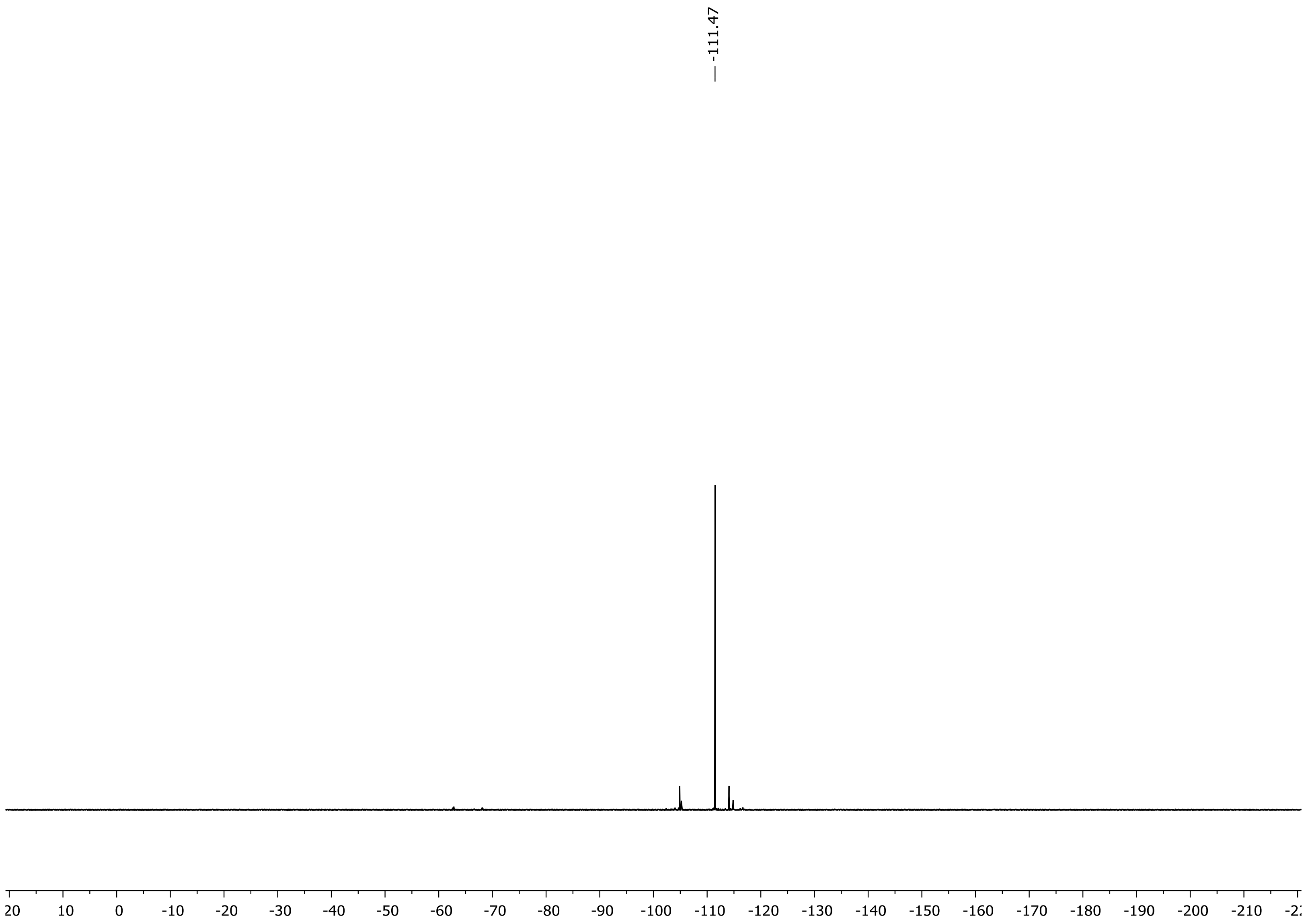

Figure S70: ^{1}**H NMR** (400 MHz, $CDCl_3$, 298 K) spectrum of compound **3d**

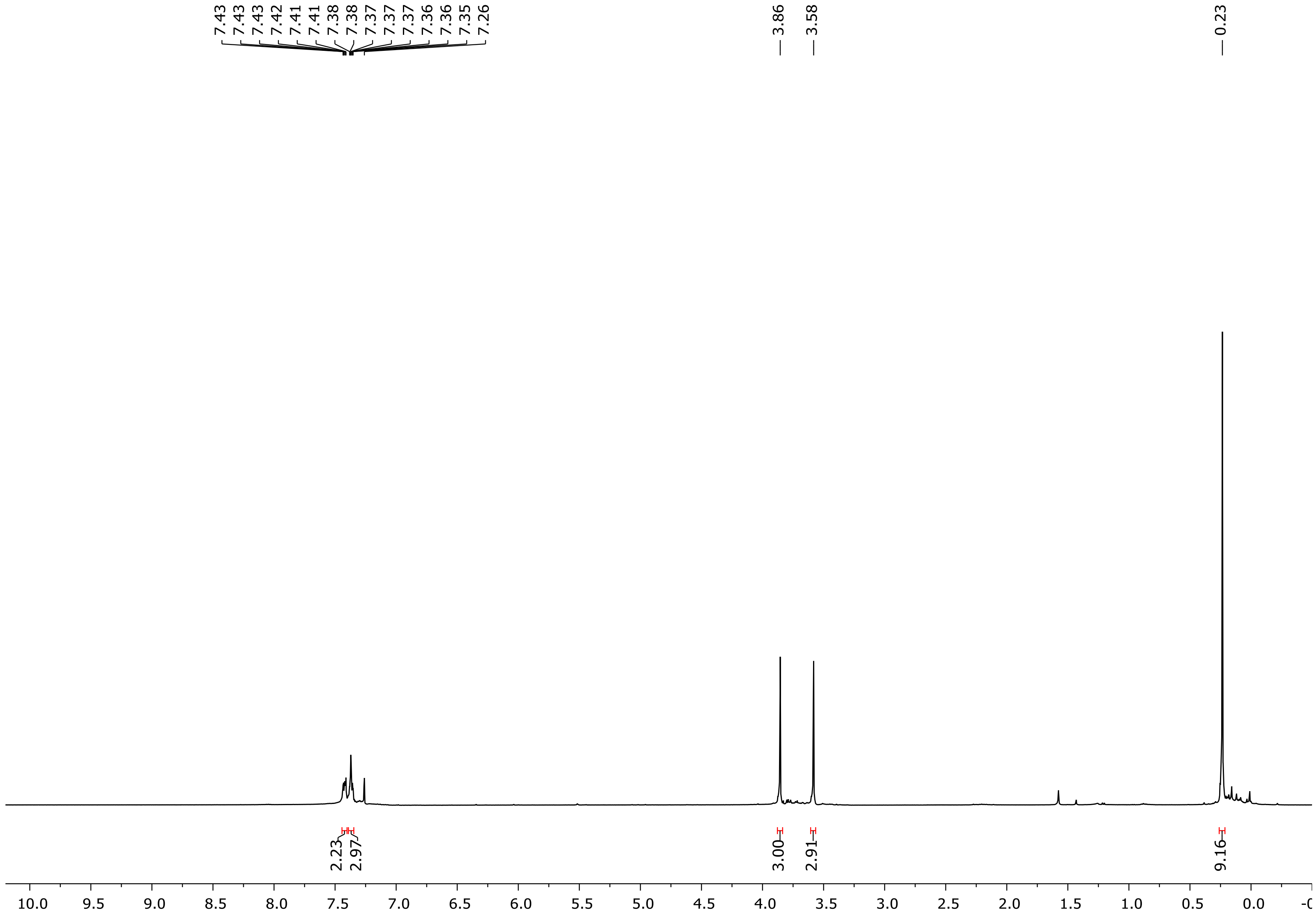

Figure S71: **^{13}C NMR** (101 MHz, $CDCl_3$, 298 K) spectrum of compound **3d**

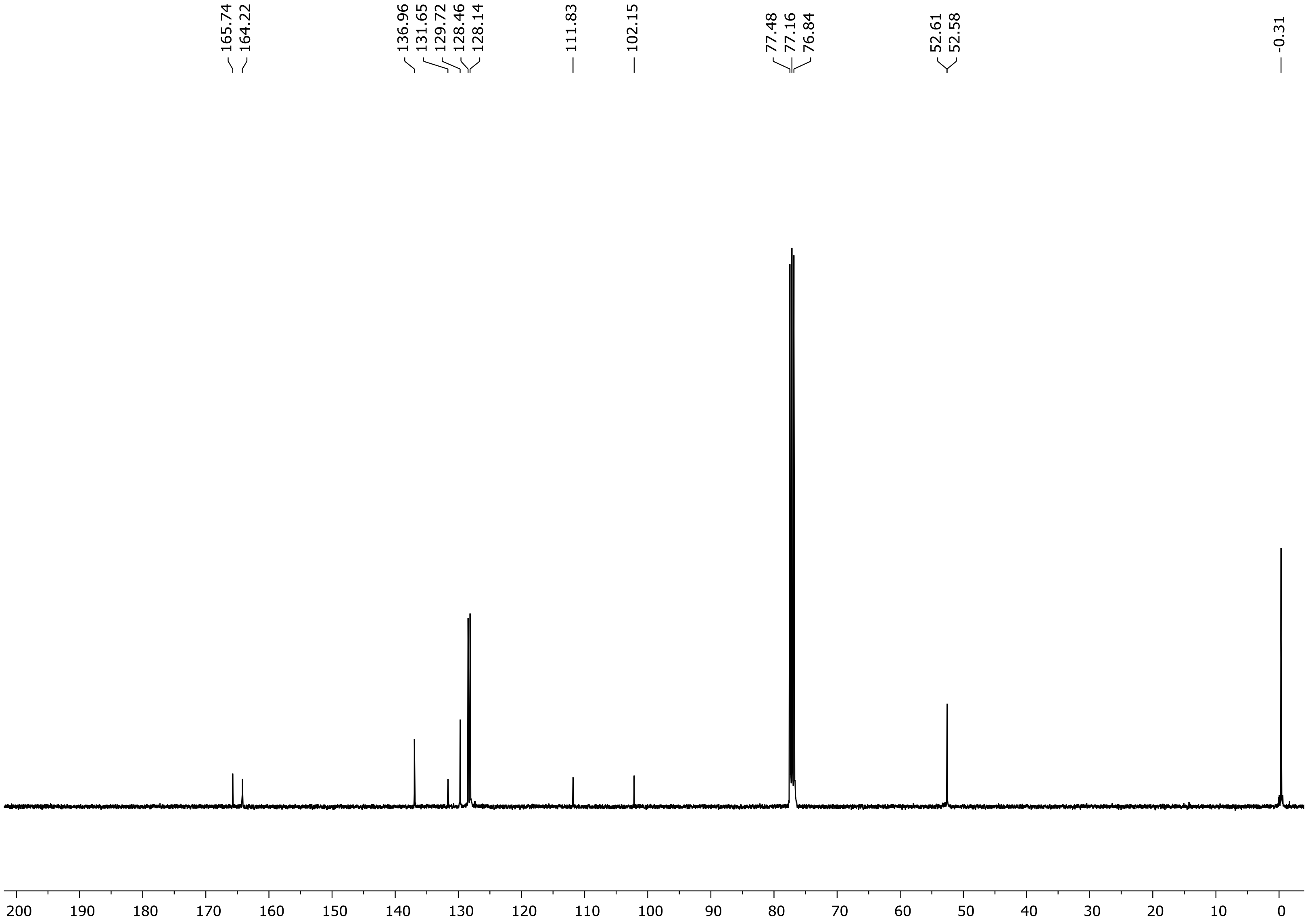

Figure S72: ^{1}**H NMR** (400 MHz, $CDCl_3$, 298 K) spectrum of compound **3e**

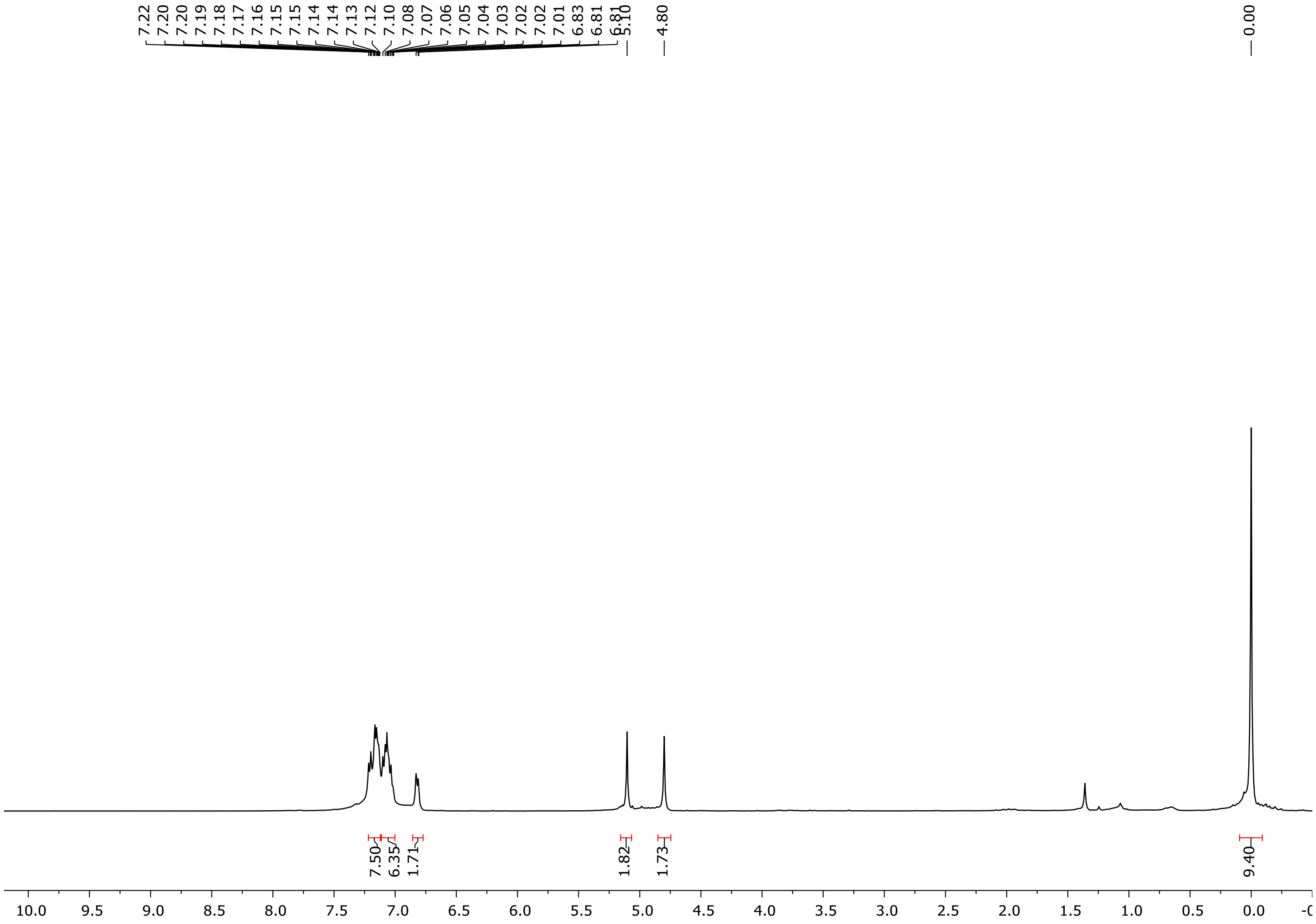

Figure S73: **^{13}C NMR** (101 MHz, $CDCl_3$, 298 K) spectrum of compound **3e**

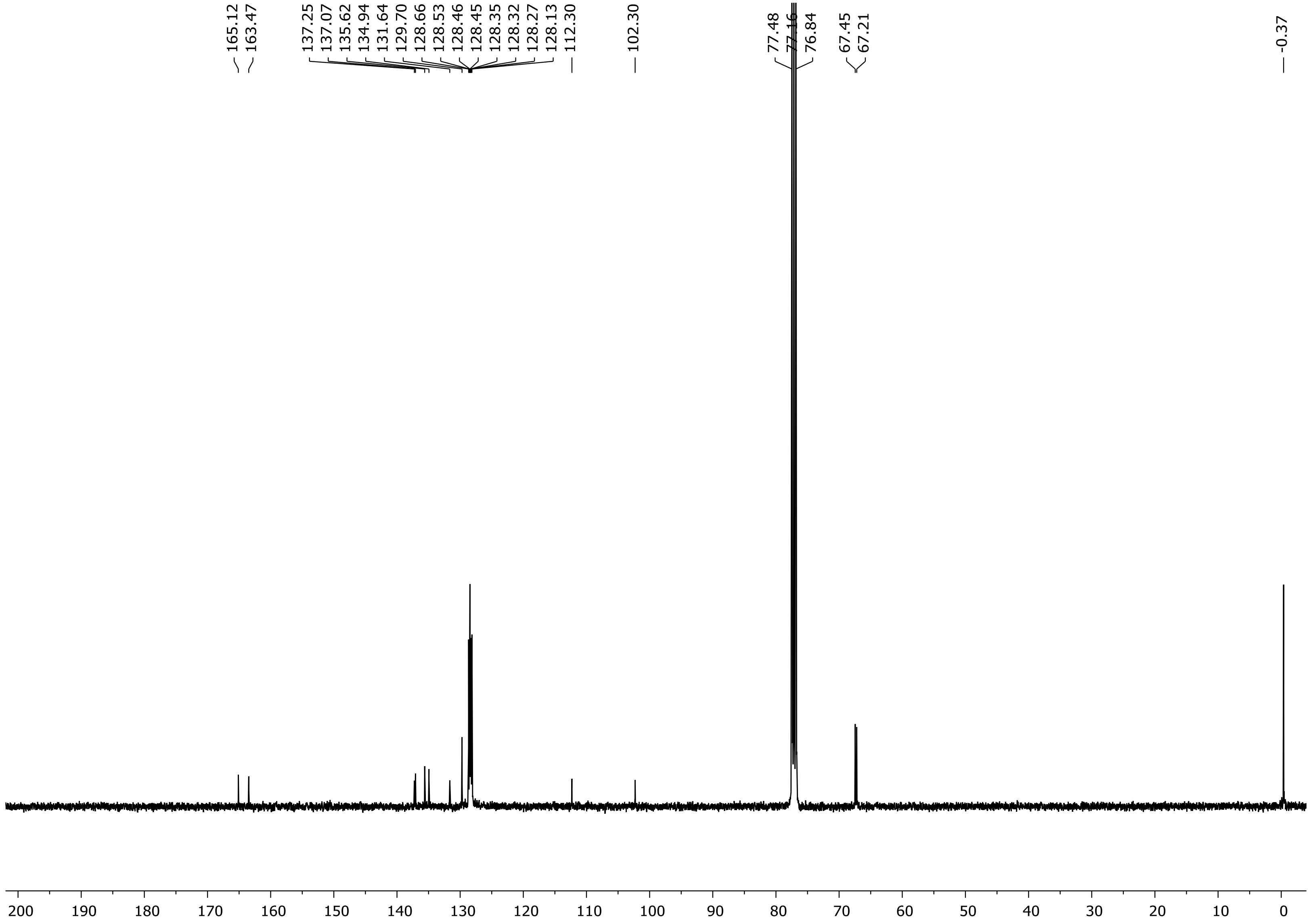

Figure S74: ^{1}**H NMR** (400 MHz, $CDCl_3$, 298 K) spectrum of compound **3f**

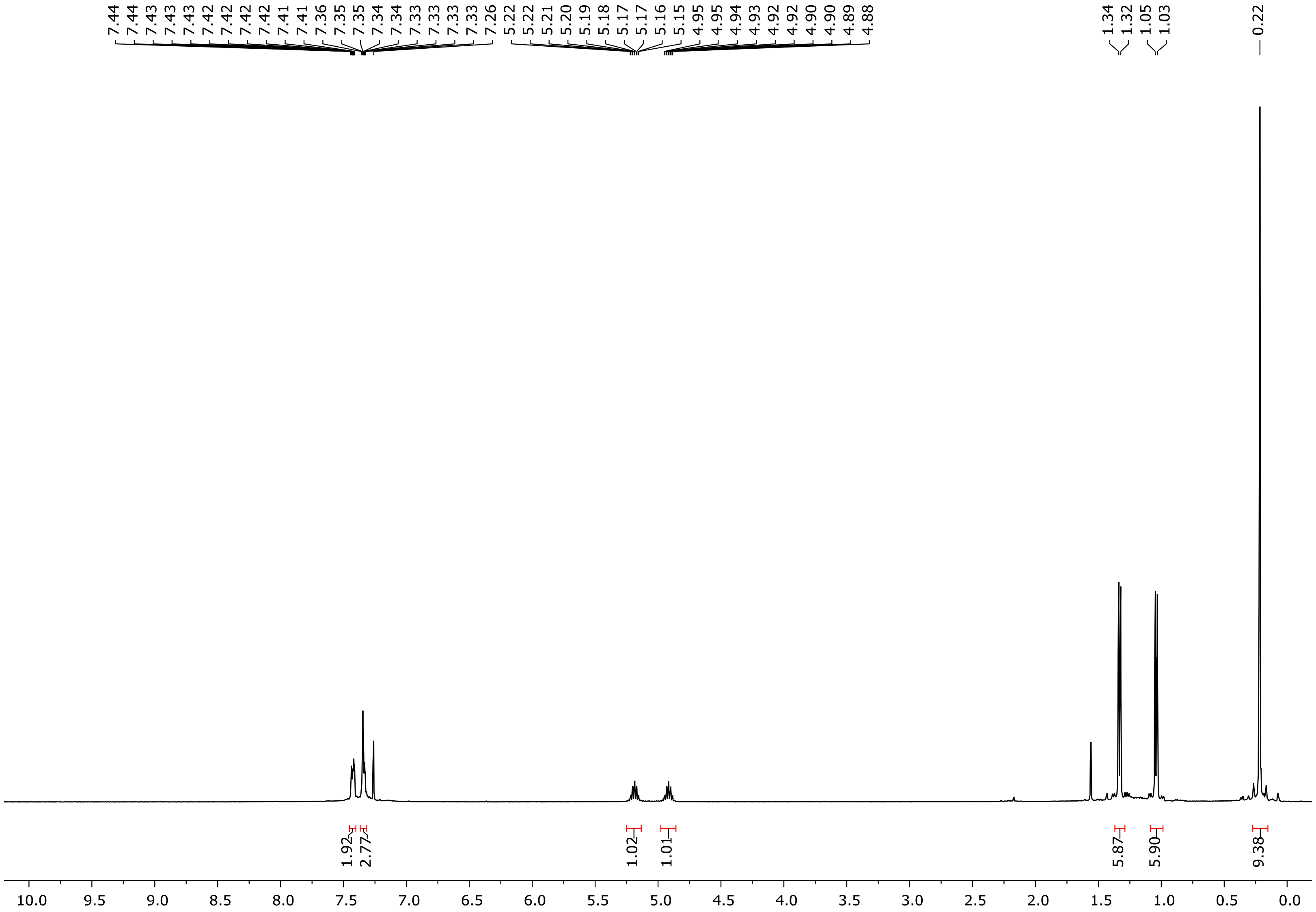

Figure S75: **^{13}C NMR** (101 MHz, $CDCl_3$, 298 K) spectrum of compound **3f**

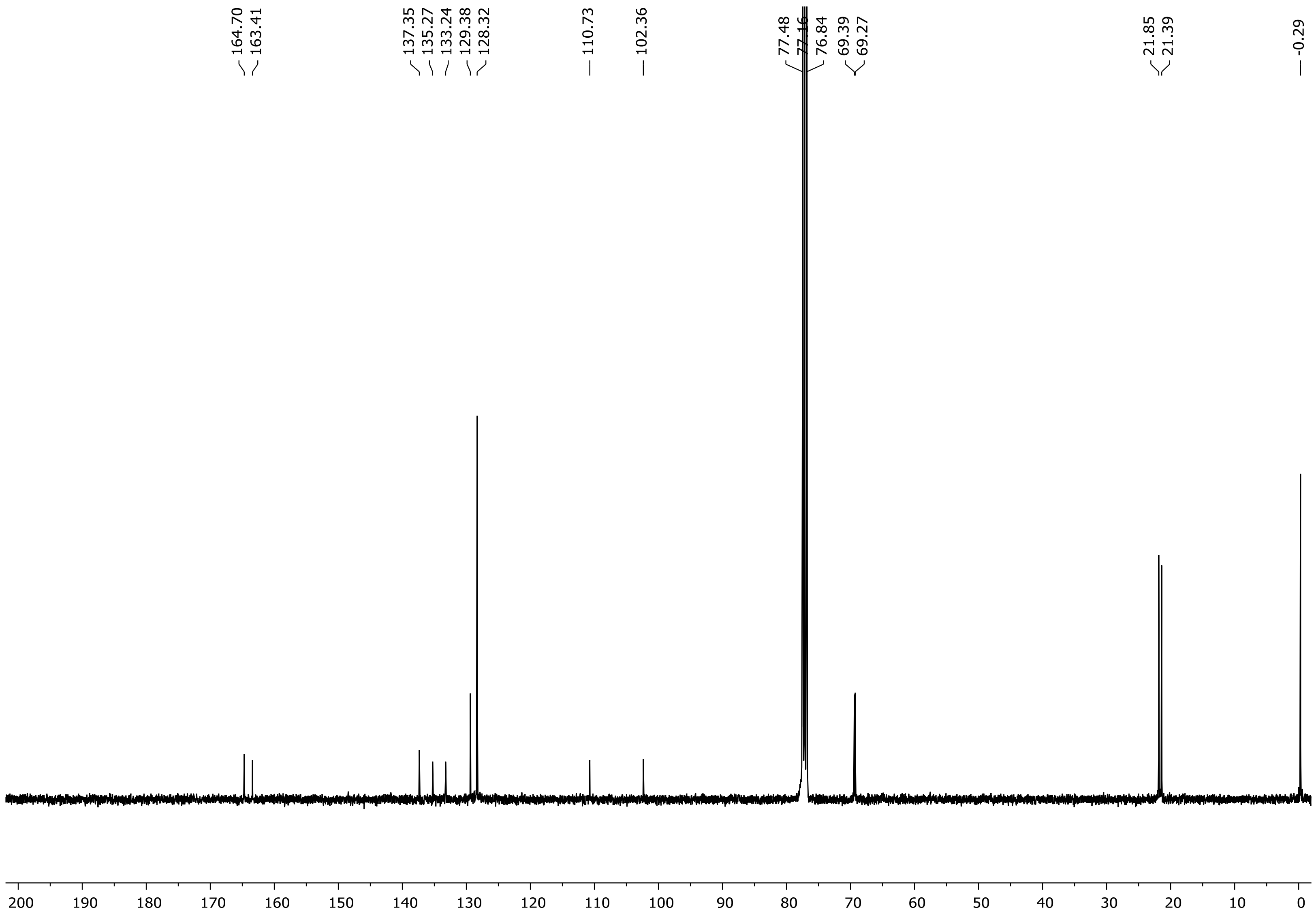

Figure S76: **^{1}H NMR** (400 MHz, $CDCl_3$, 298 K) spectrum of compound **3g**

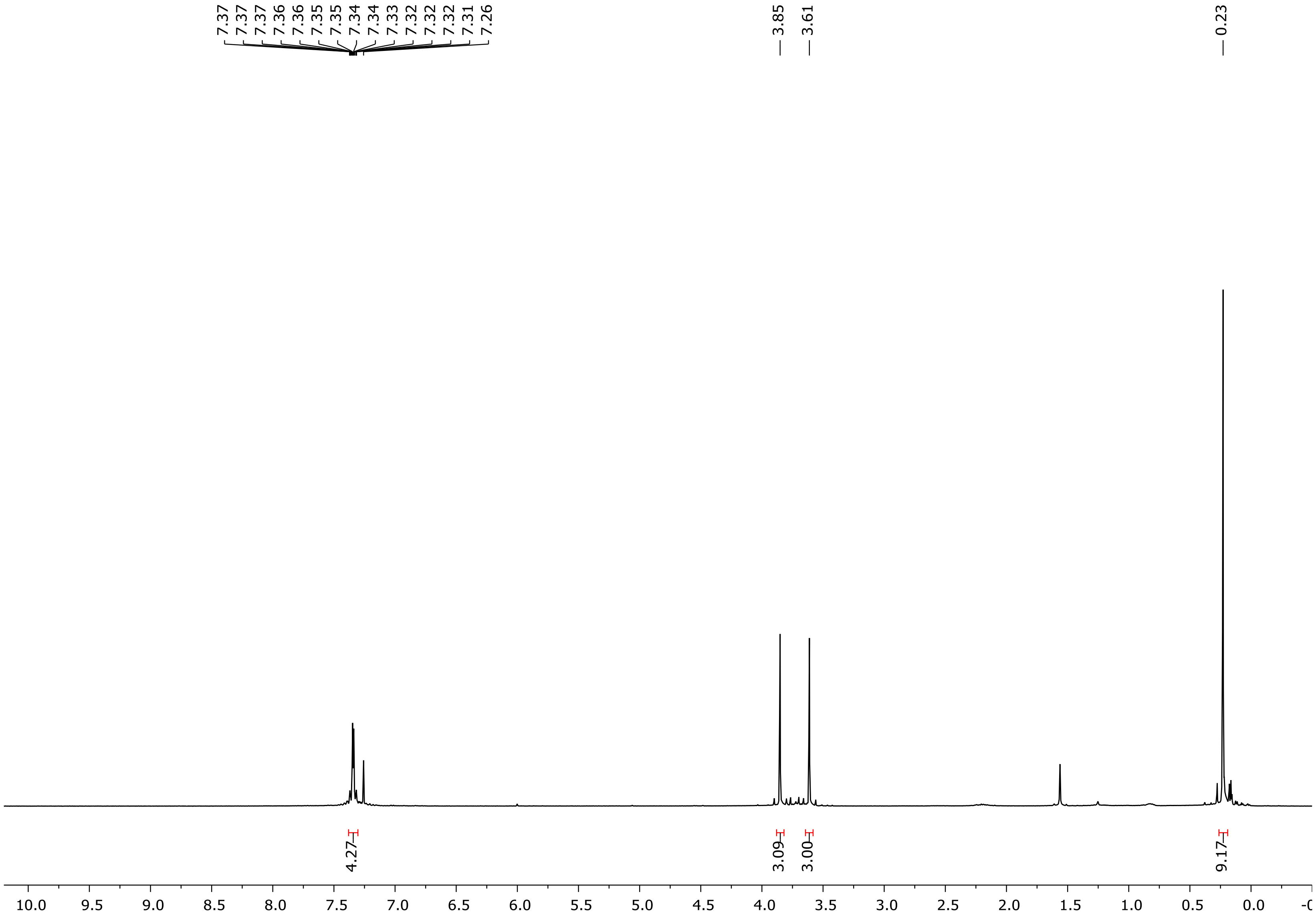

Figure S77: **^{13}C NMR** (101 MHz, $CDCl_3$, 298 K) spectrum of compound **3g**

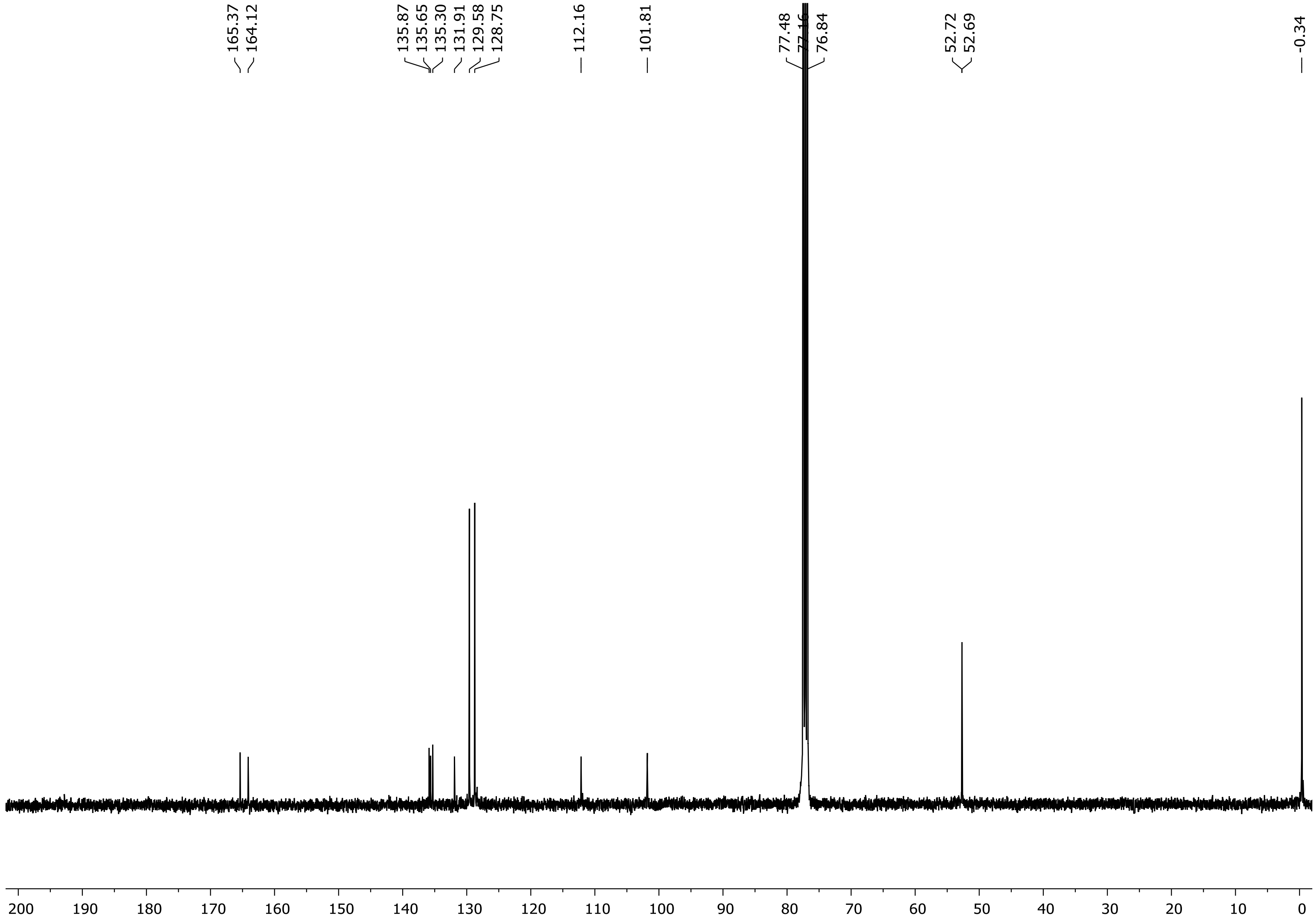

Figure S78: **^{1}H NMR** (400 MHz, $CDCl_3$, 298 K) spectrum of compound **3h**

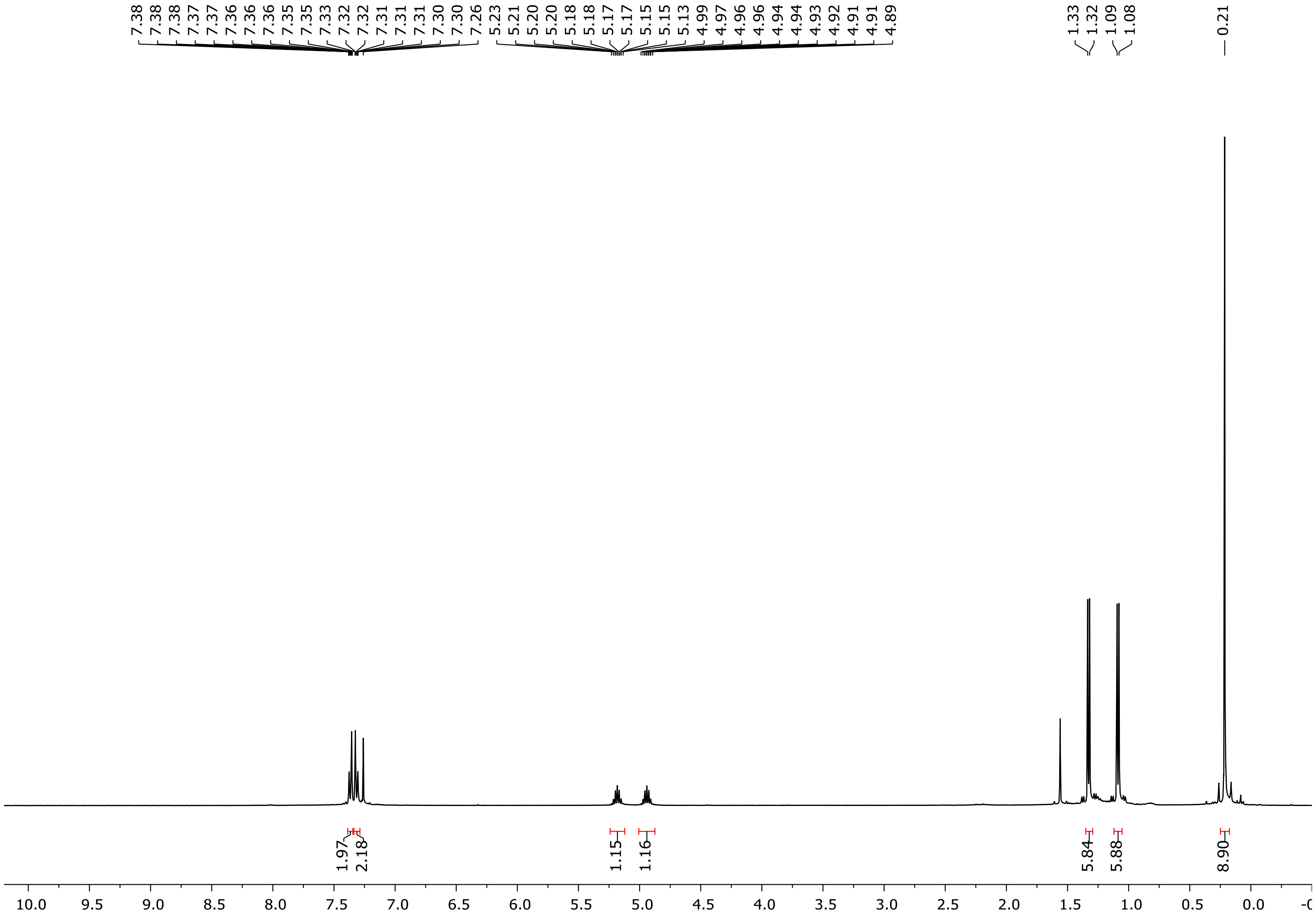

Figure S79: **^{13}C NMR** (101 MHz, $CDCl_3$, 298 K) spectrum of compound **3h**

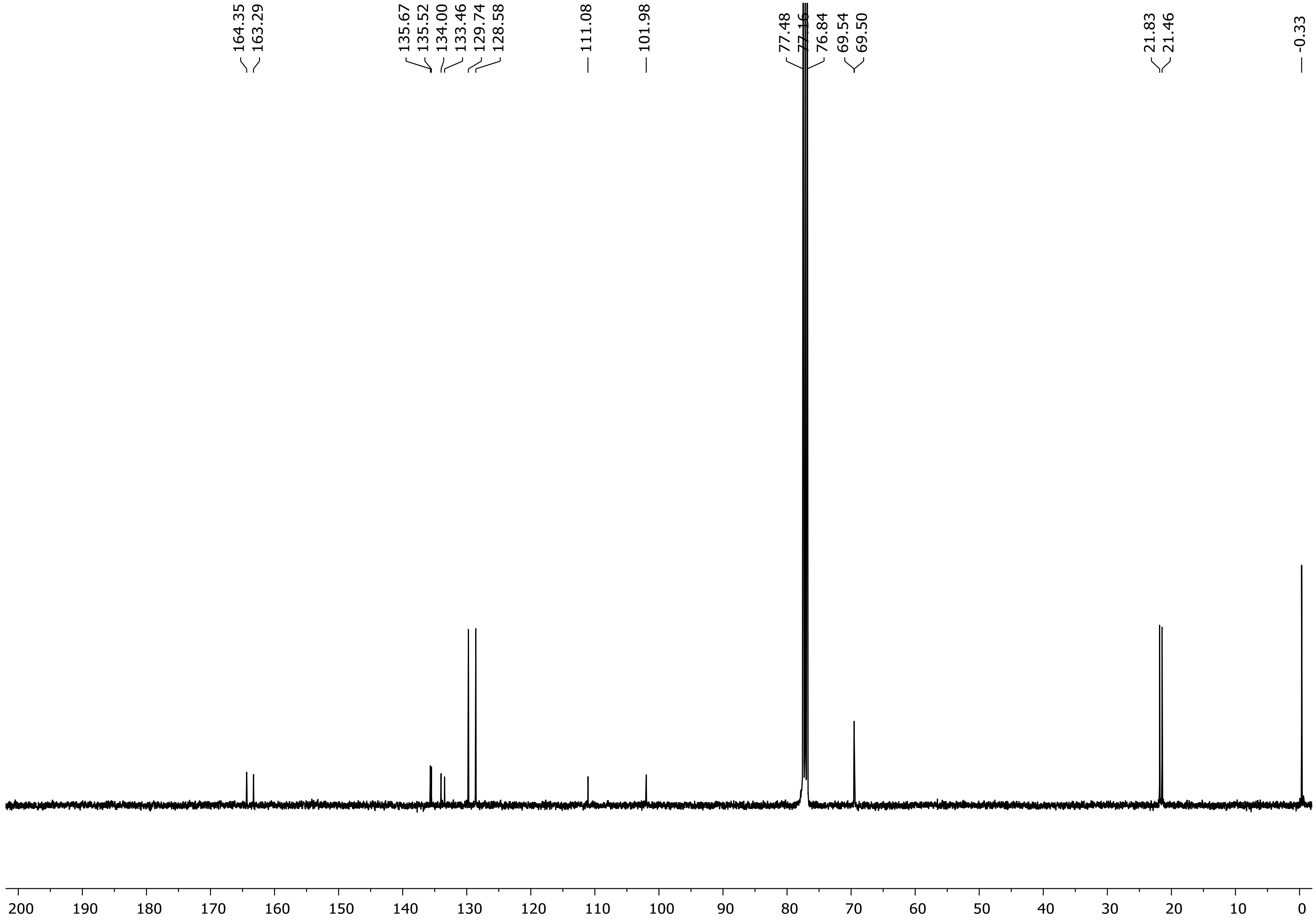

Figure S80: ^{1}**H NMR** (400 MHz, $CDCl_3$, 298 K) spectrum of compound **3i**

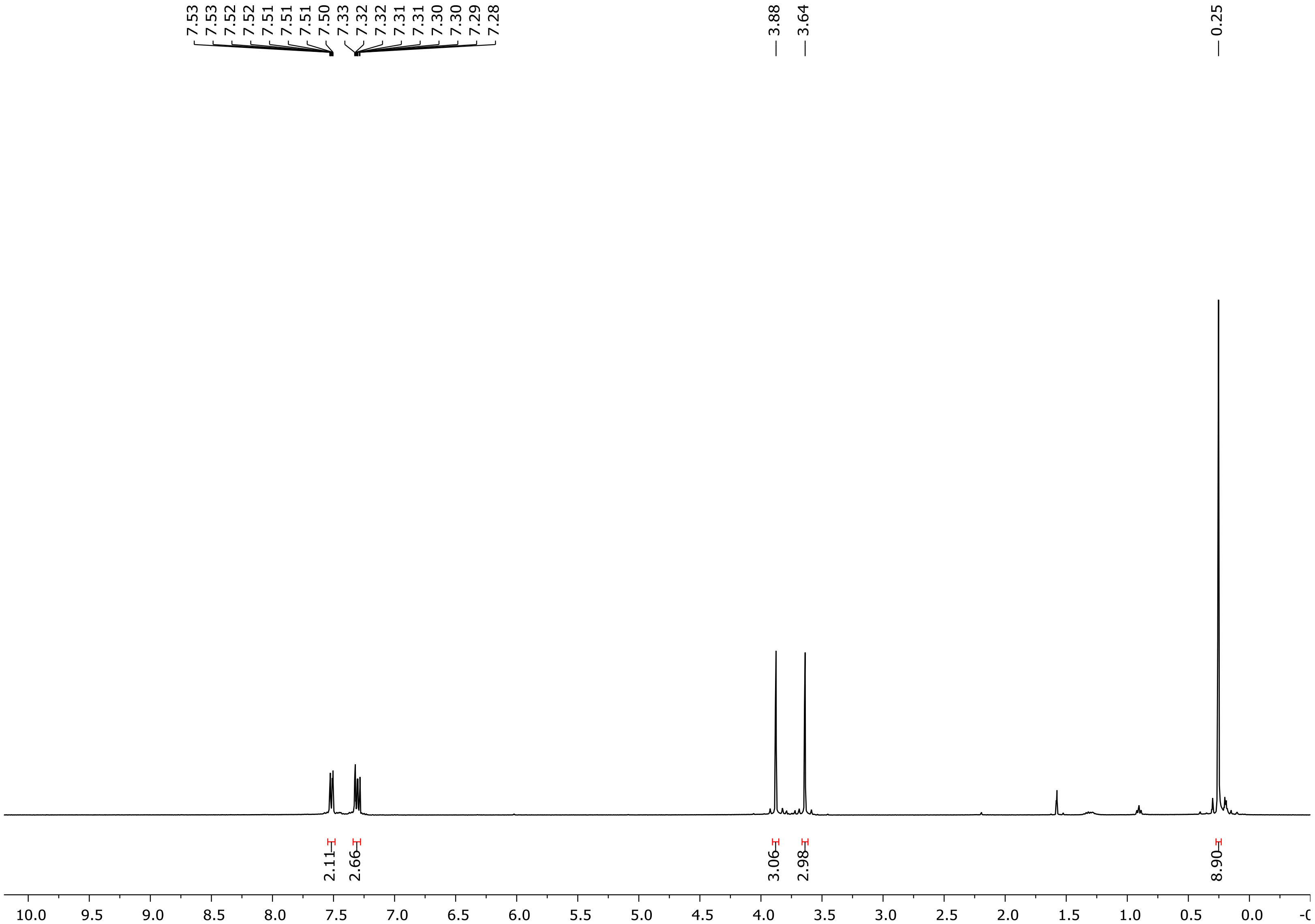

Figure S81: **^{13}C NMR** (101 MHz, $CDCl_3$, 298 K) spectrum of compound **3i**

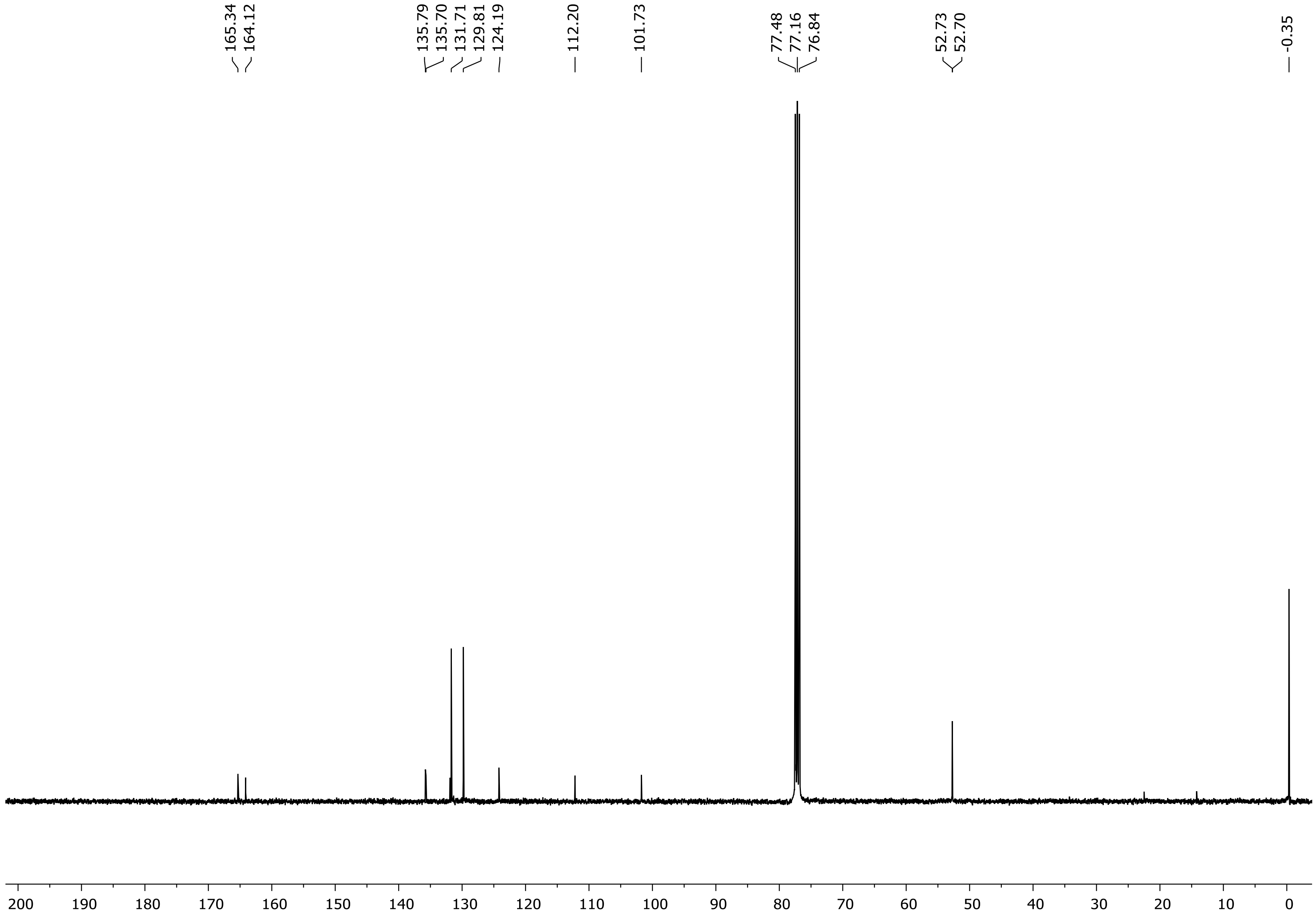

Figure S82: **¹H NMR** (500 MHz, $CDCl_3$, 298 K) spectrum of compound **3j**

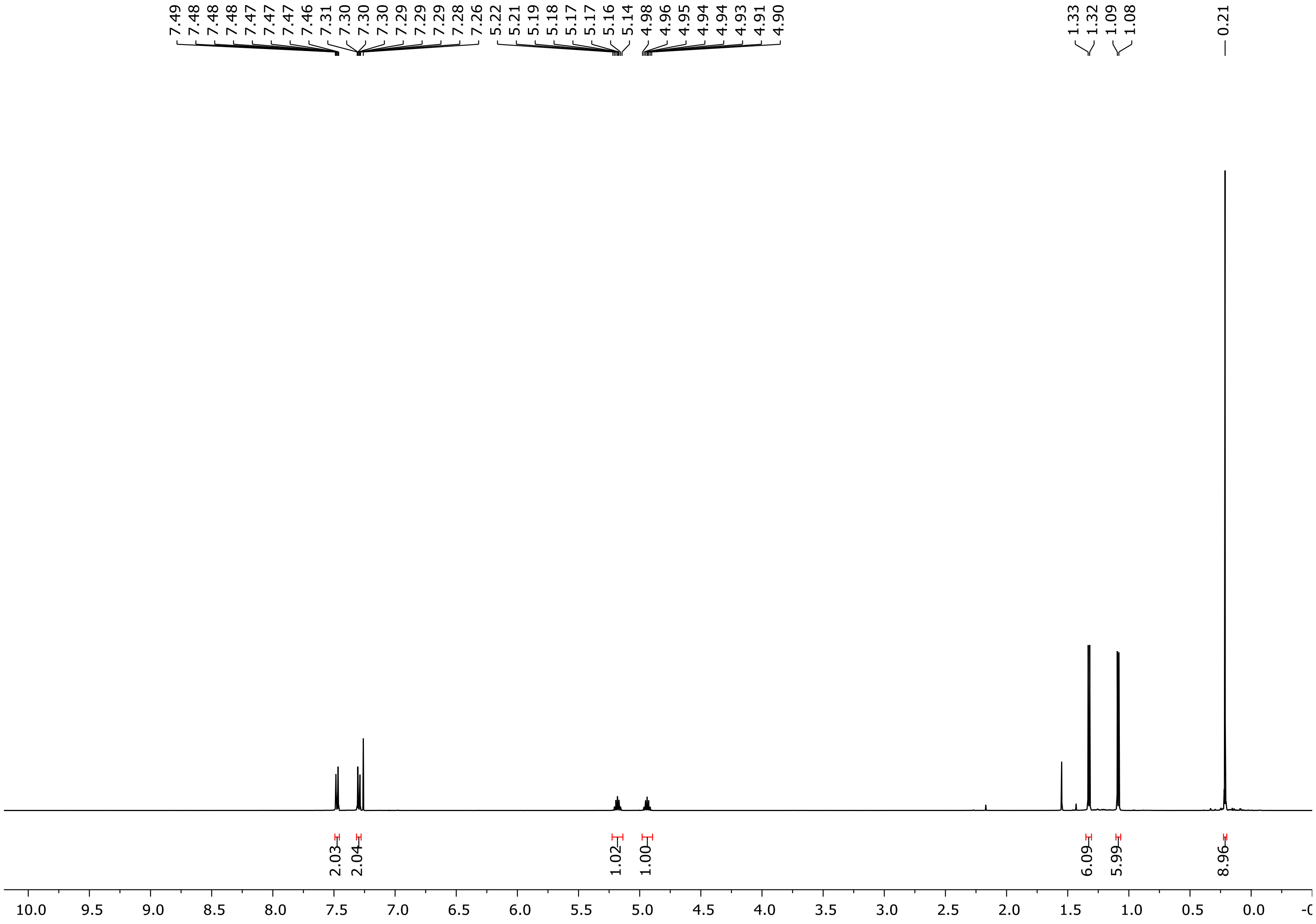

Figure S83: **^{13}C NMR** (126 MHz, $CDCl_3$, 298 K) spectrum of compound **3j**

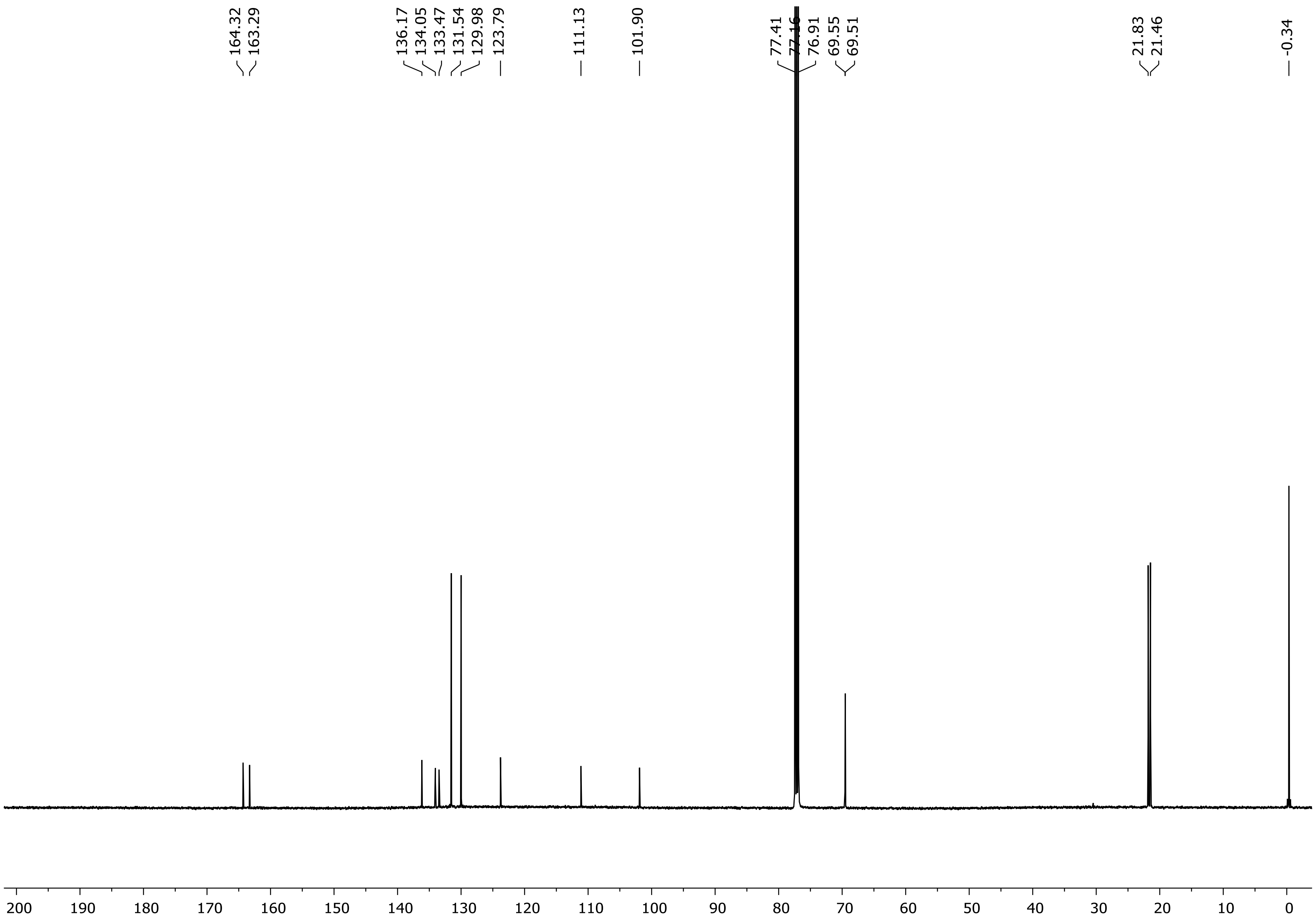

Figure S84: **^{1}H NMR** (400 MHz, $CDCl_3$, 298 K) spectrum of compound **3k**

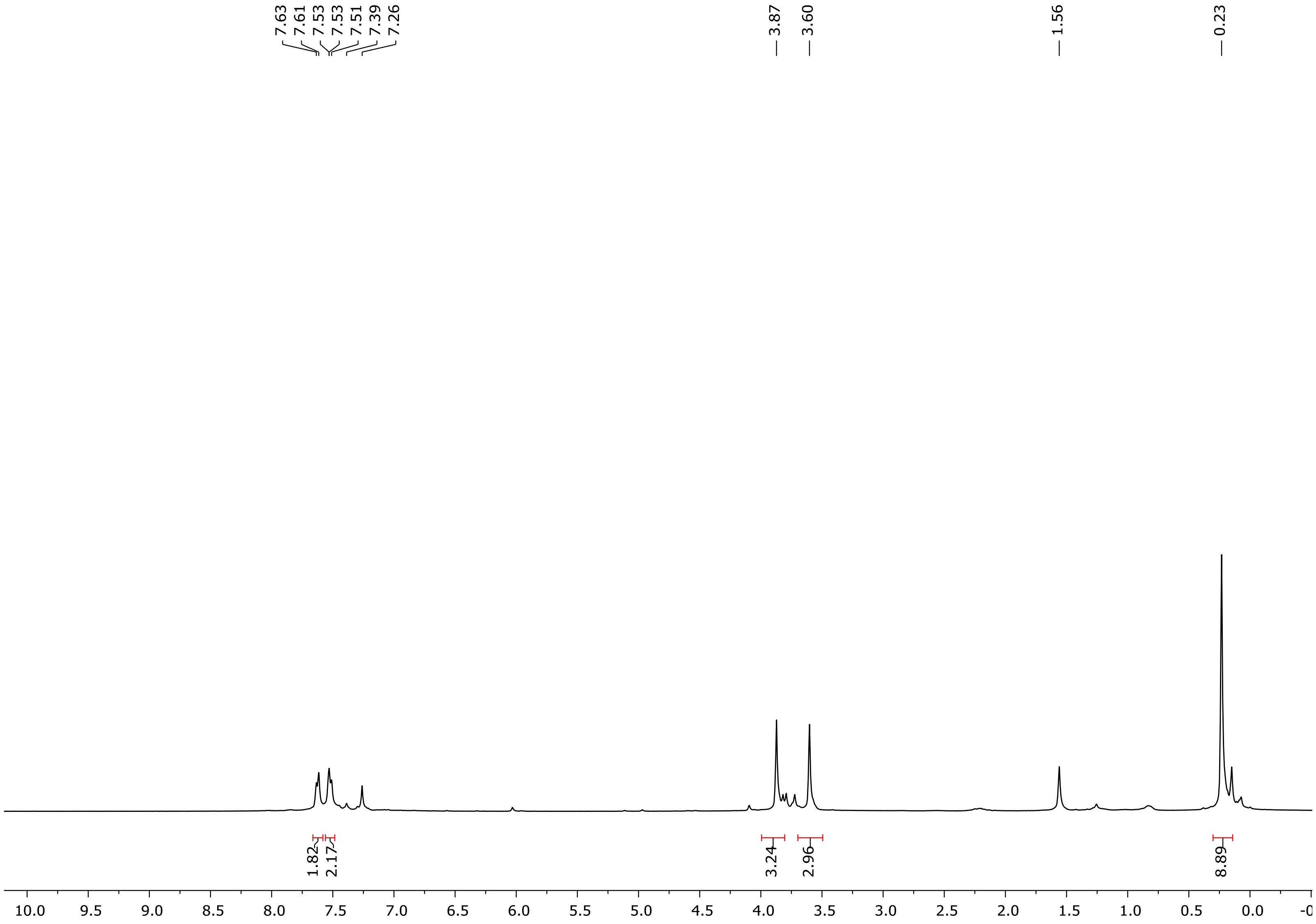

Figure S85: **^{13}C NMR** (101 MHz, $CDCl_3$, 298 K) spectrum of compound **3k**

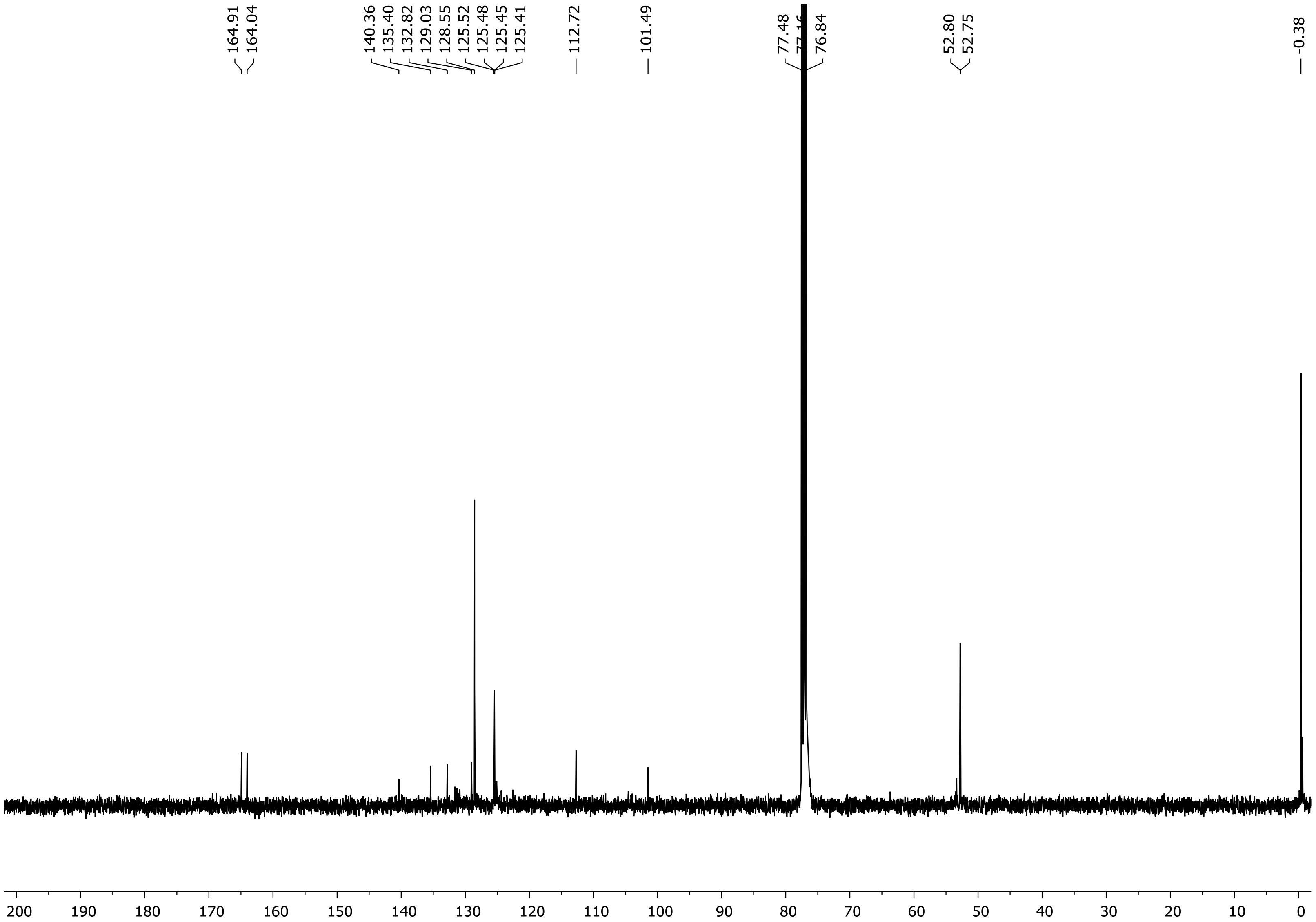

Figure S86: **^{19}F NMR** (376 MHz, $CDCl_3$, 298 K) spectrum of compound **3k**

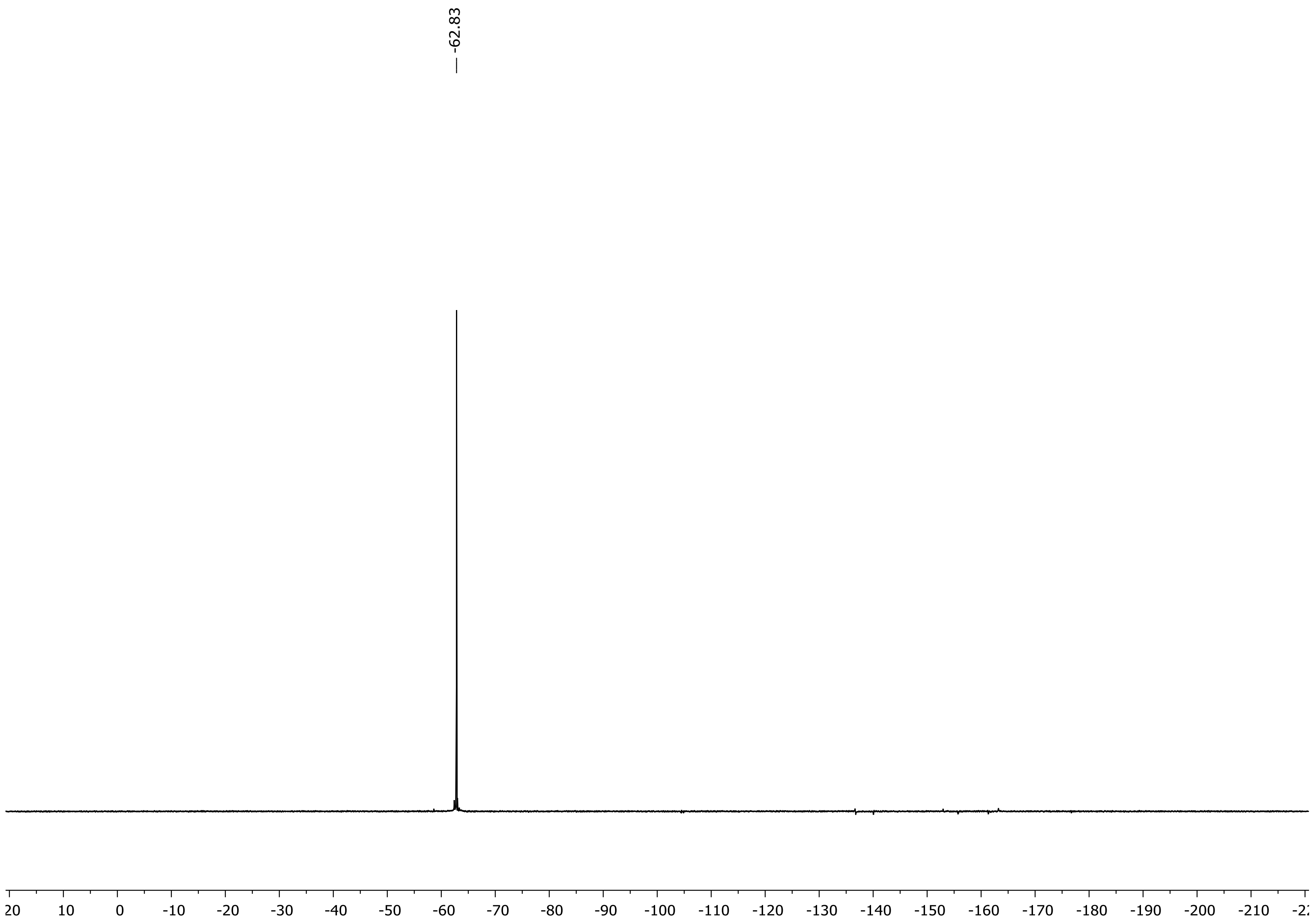

Figure S87: ^{1}**H NMR** (400 MHz, $CDCl_3$, 298 K) spectrum of compound **3l**

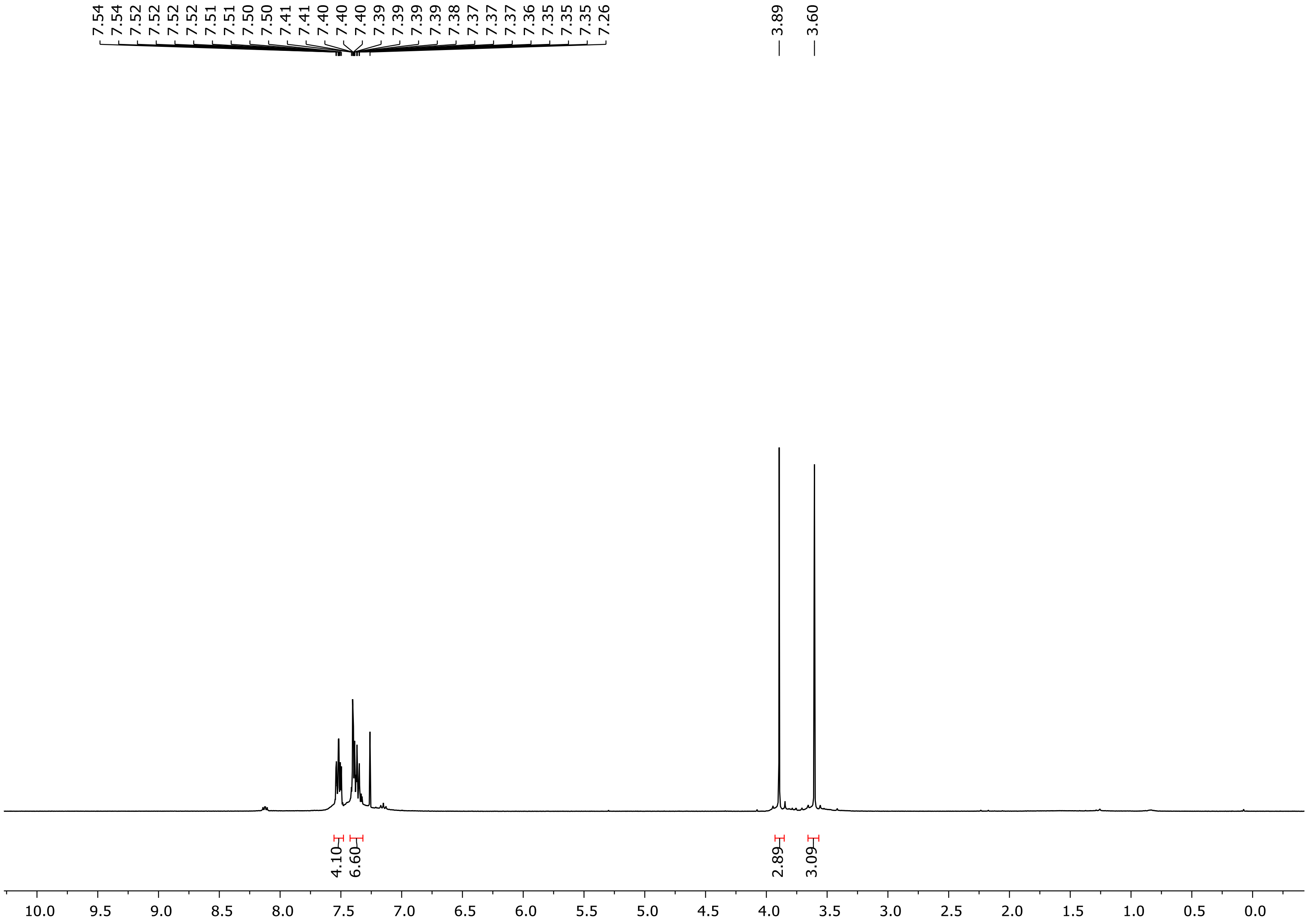

Figure S88: ^{13}**C NMR** (101 MHz, $CDCl_3$, 298 K) spectrum of compound **3l**

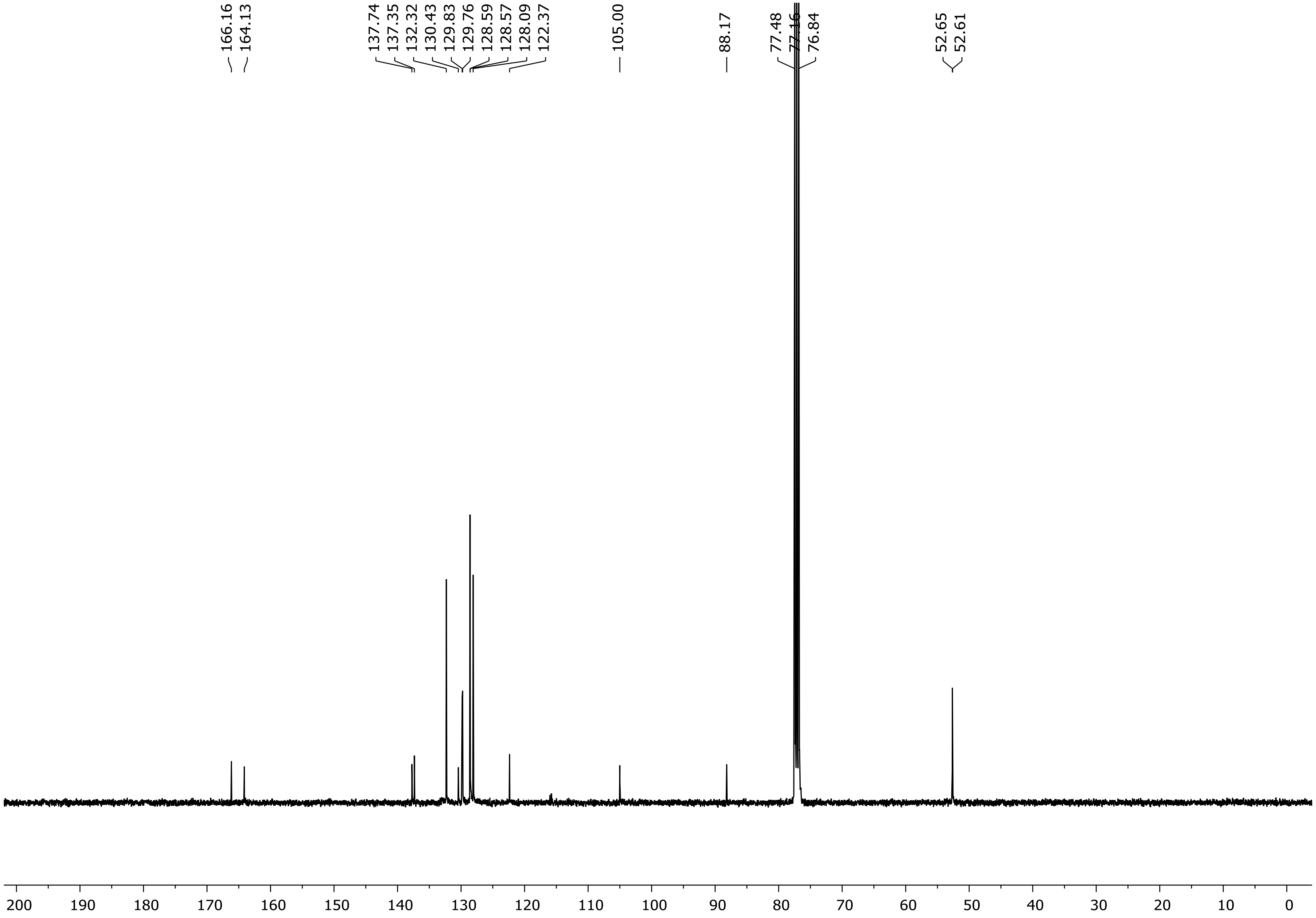

Figure S89: [1]**H NMR** (500 MHz, $CDCl_3$, 298 K) spectrum of compound **3m**

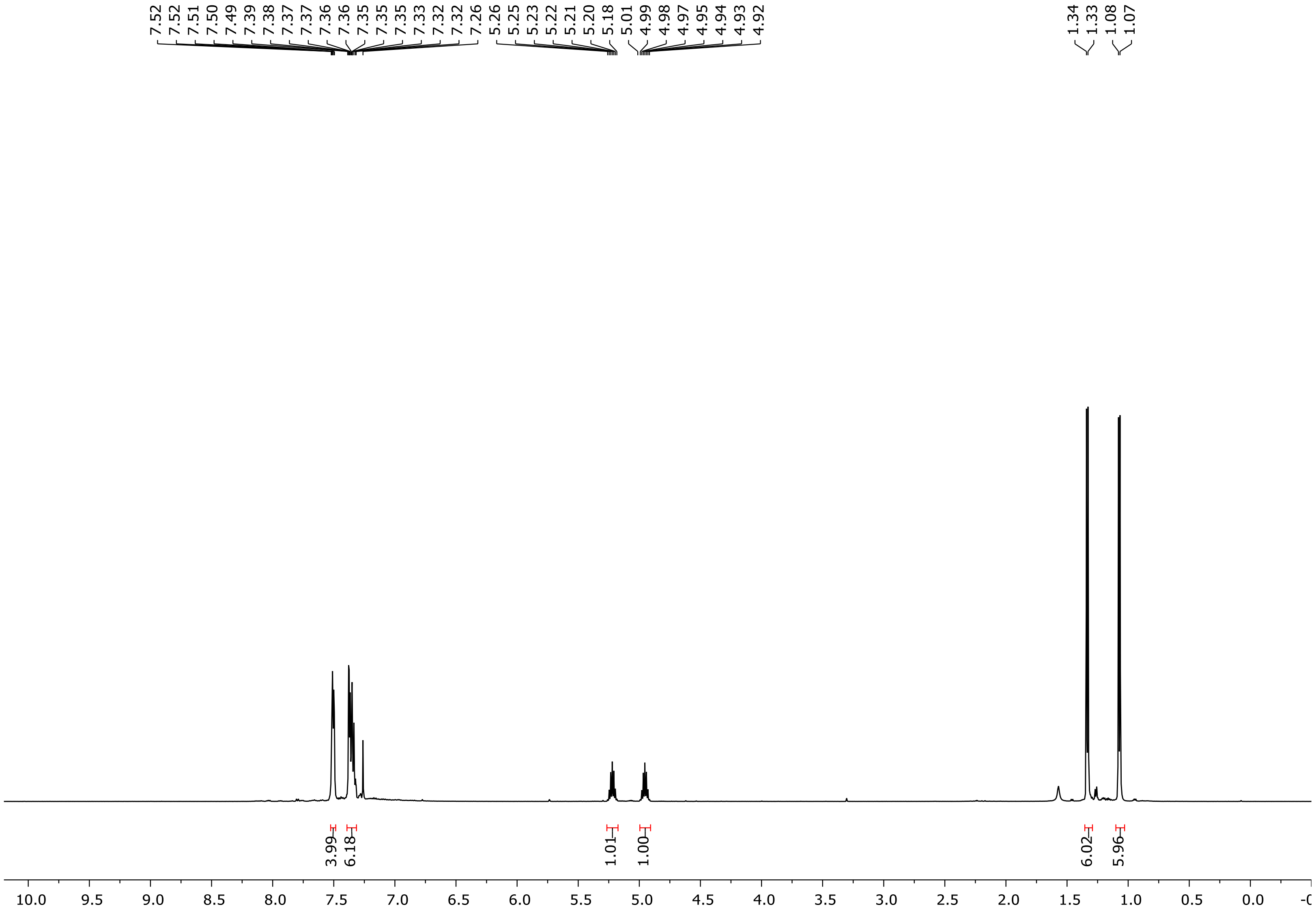

Figure S90: ^{13}**C NMR** (126 MHz, $CDCl_3$, 298 K) spectrum of compound **3m**

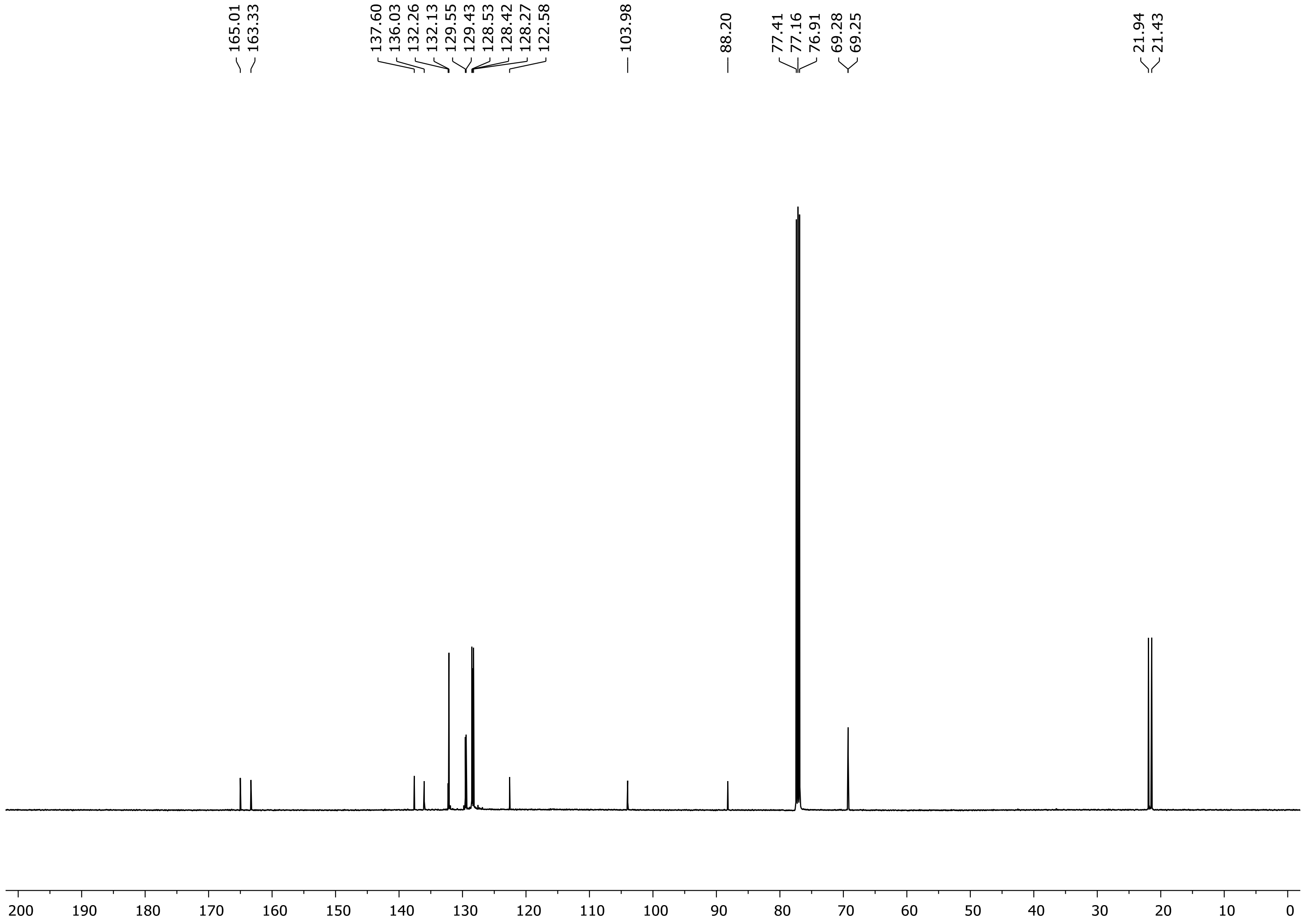

Figure S91: ^{1}**H NMR** (400 MHz, $CDCl_3$, 298 K) spectrum of compound **3n**

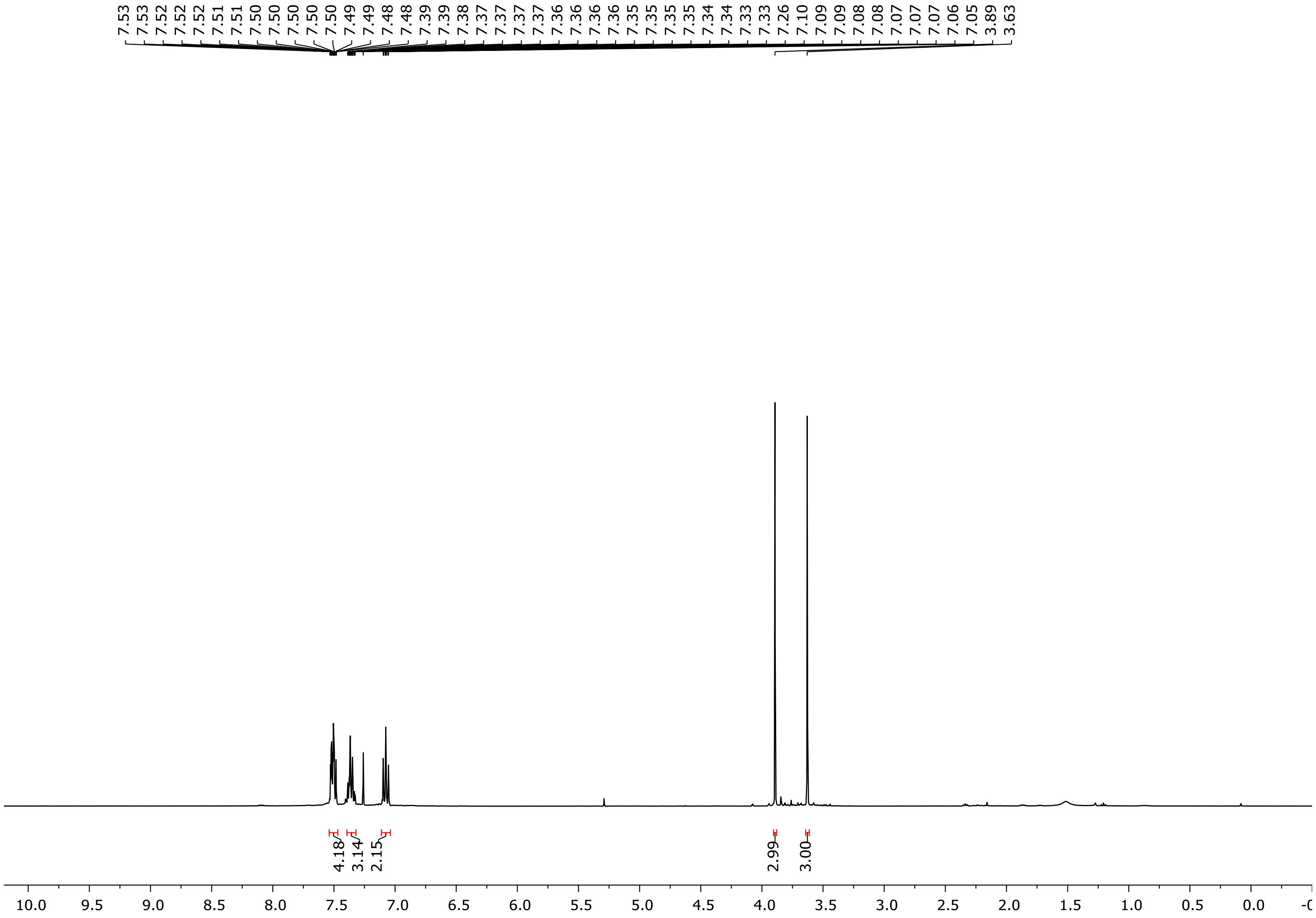

Figure S92: **^{13}C NMR** (101 MHz, $CDCl_3$, 298 K) spectrum of compound **3n**

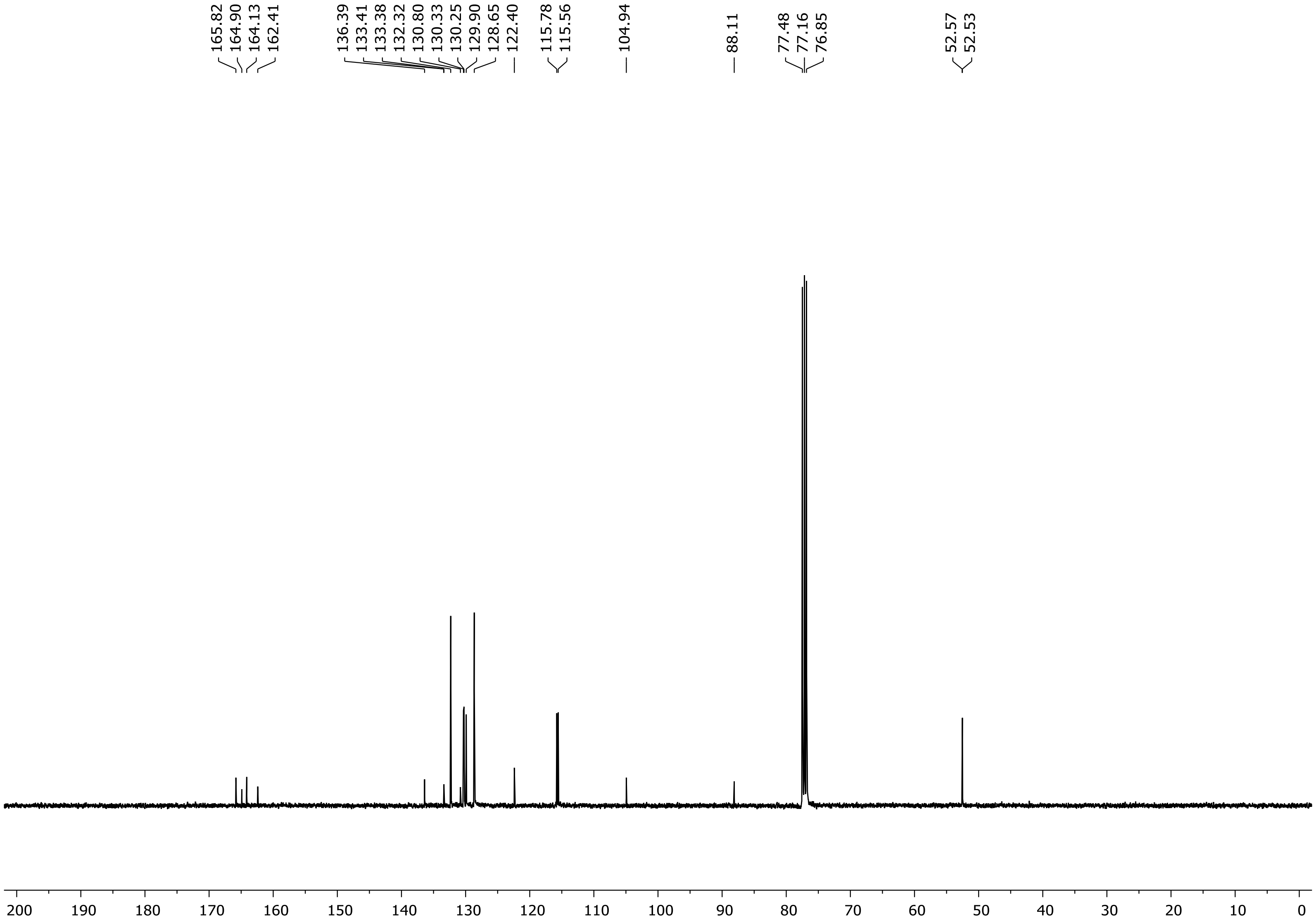

Figure S93: **^{19}F NMR** (376 MHz, $CDCl_3$, 298 K) spectrum of compound **3n**

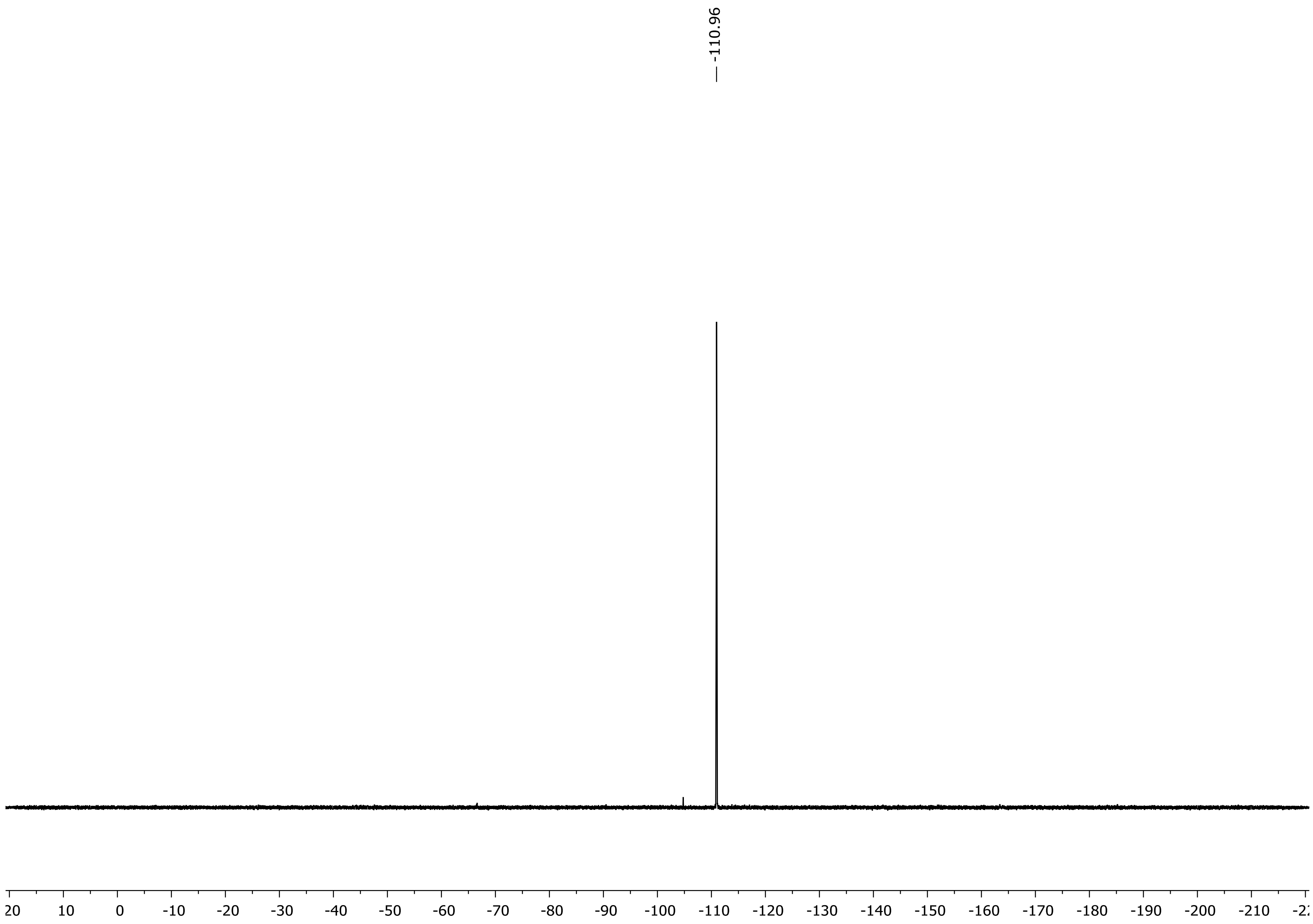

Figure S94: **^{1}H NMR** (500 MHz, $CDCl_3$, 298 K) spectrum of compound **3o**

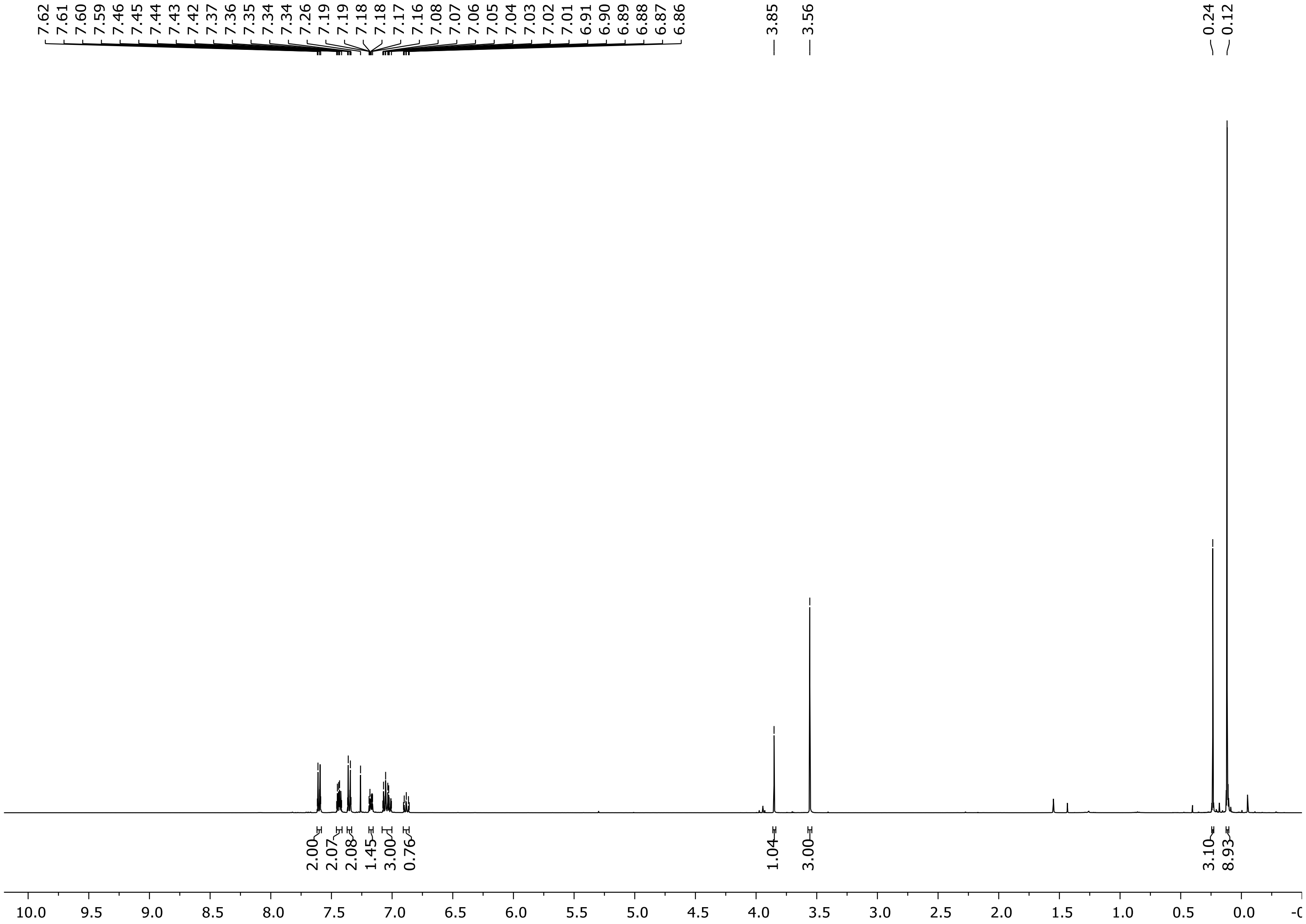

Figure S95: **^{13}C NMR** (126 MHz, $CDCl_3$, 298 K) spectrum of compound **3o**

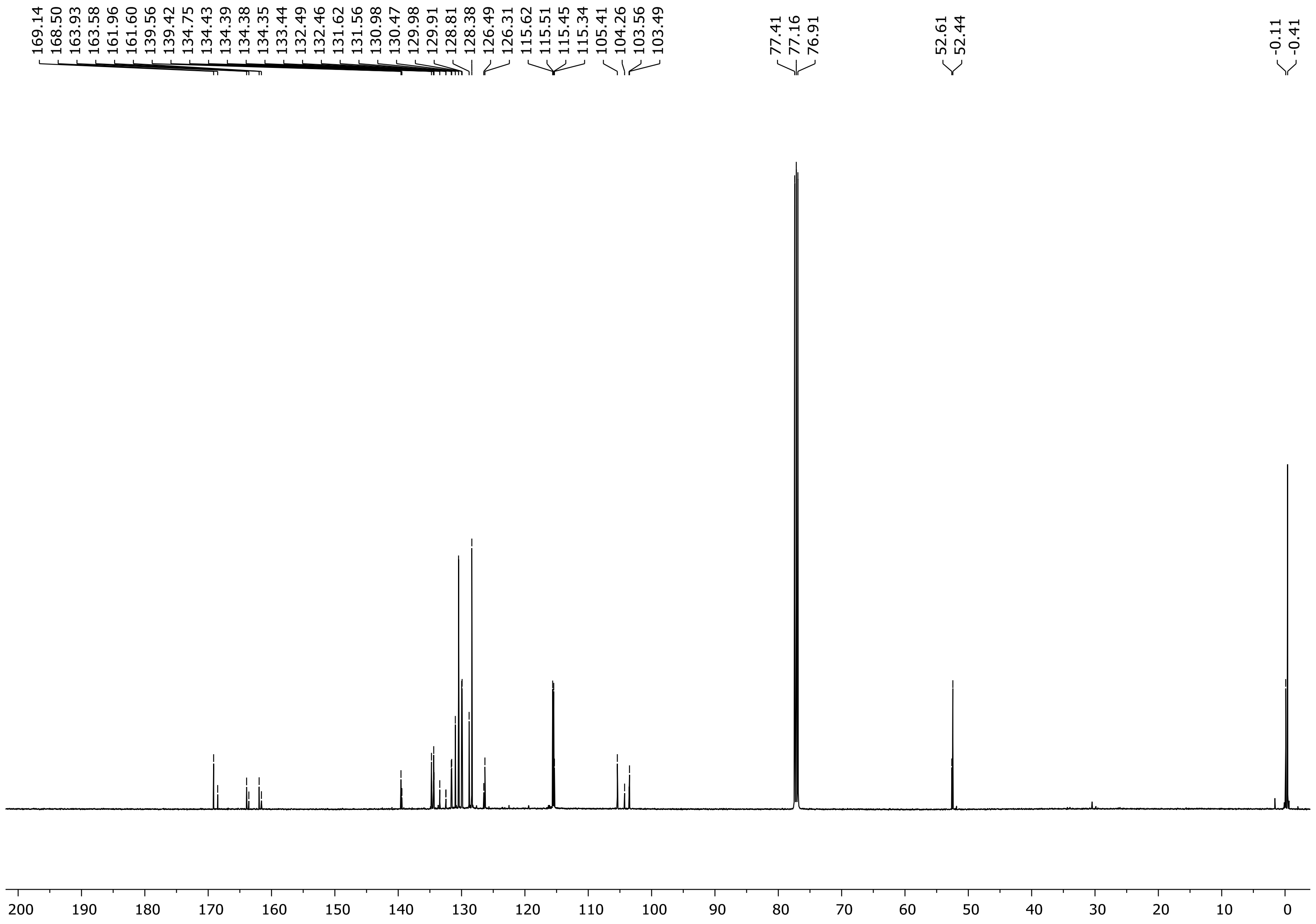

Figure S96: **^{19}F NMR** (471 MHz, $CDCl_3$, 298 K) spectrum of compound **3o**

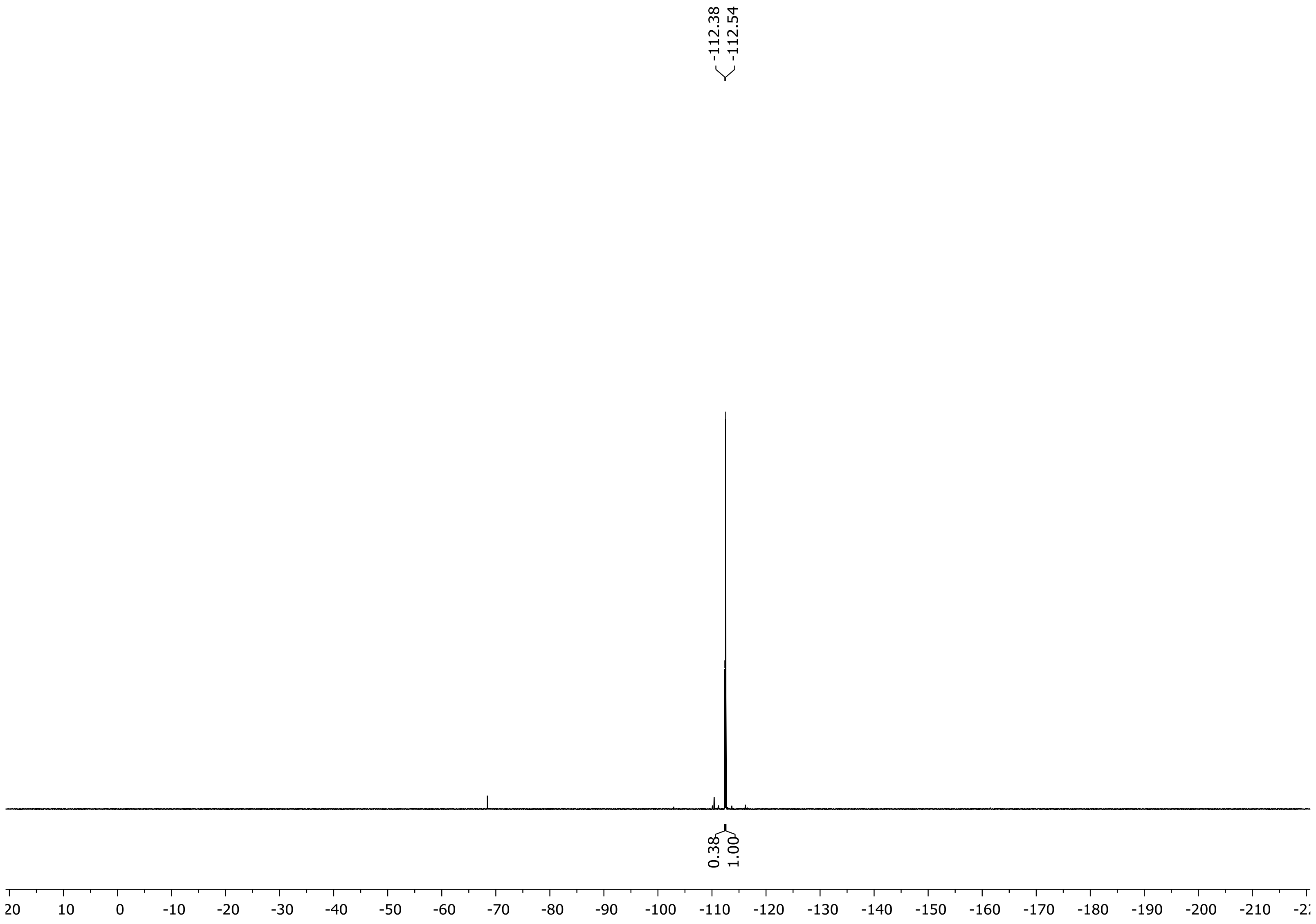

Figure S97: ^{1}**H NMR** (500 MHz, $CDCl_3$, 298 K) spectrum of compound **3p**

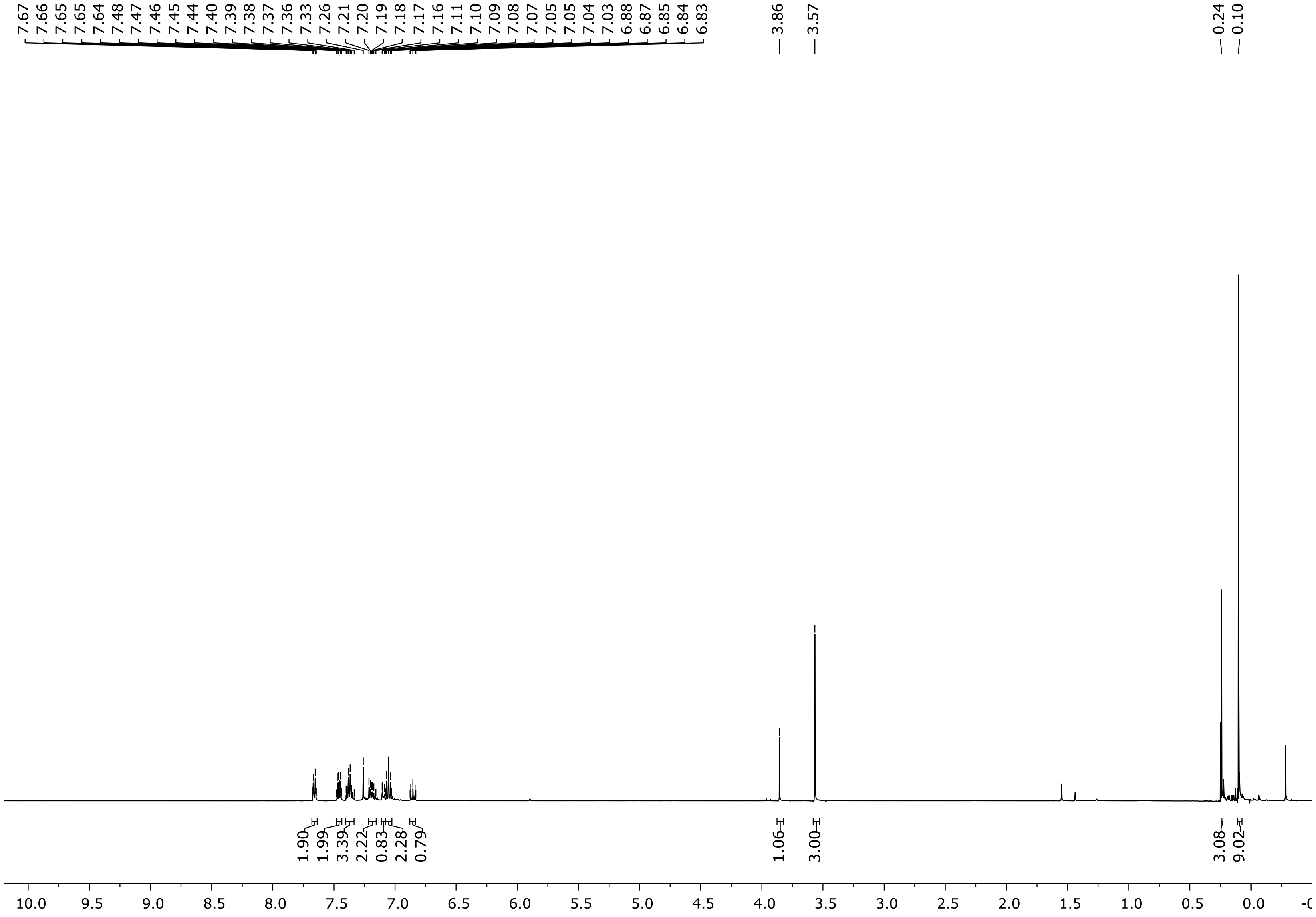

Figure S98: **^{13}C NMR** (126 MHz, $CDCl_3$, 298 K) spectrum of compound **3p**

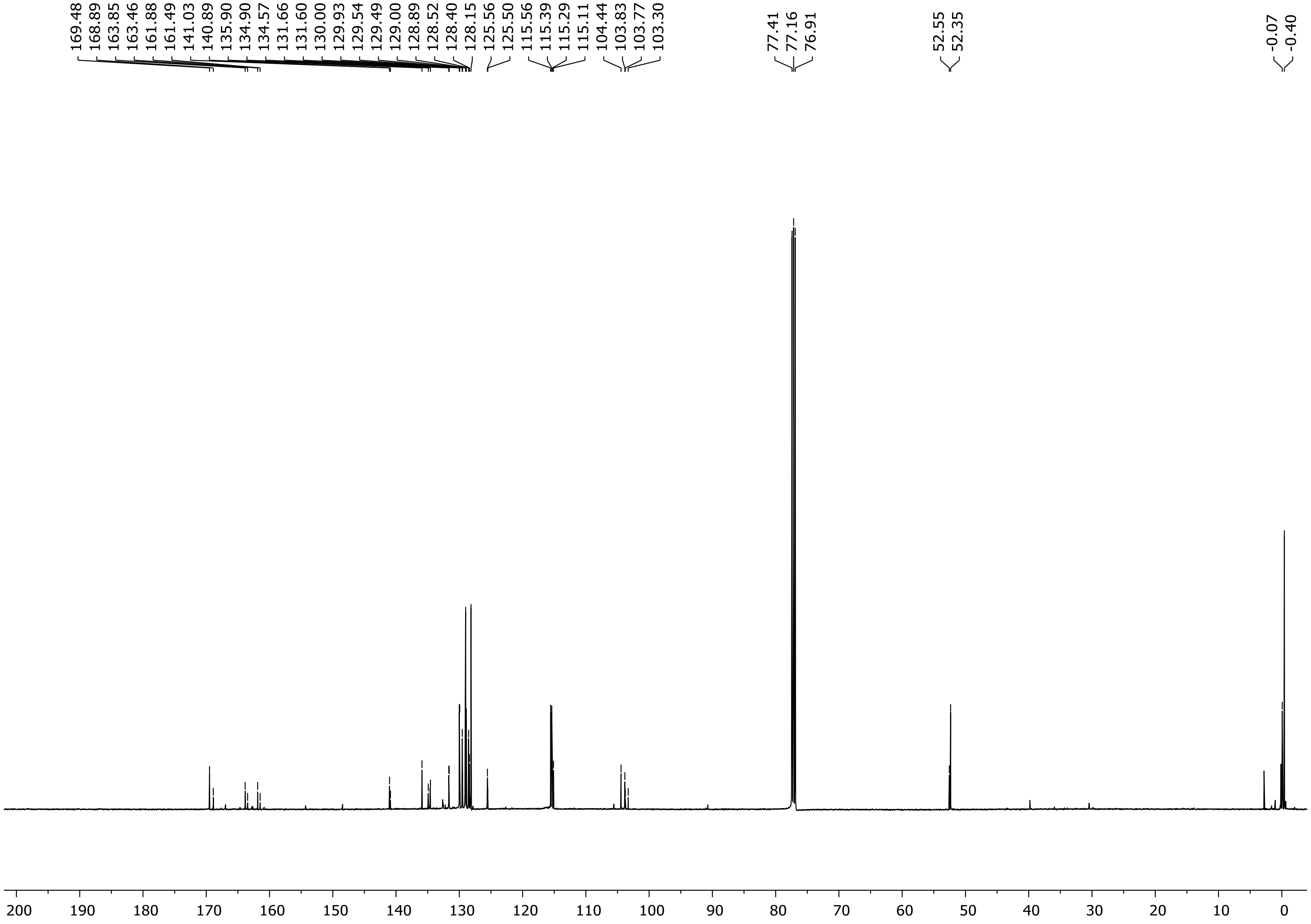

Figure S99: **^{1}H NMR** (500 MHz, $CDCl_3$, 298 K) spectrum of compound **3q**

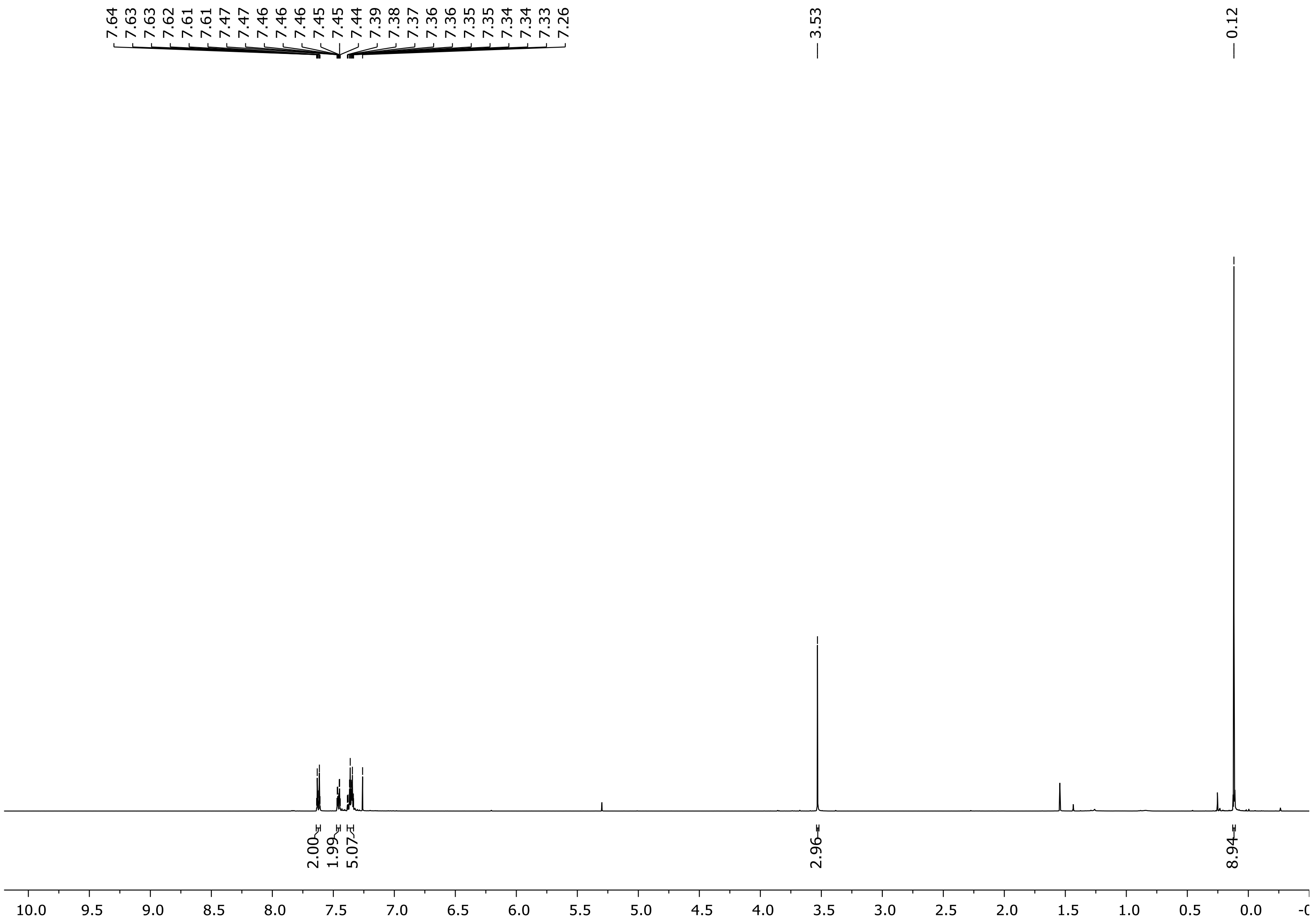

Figure S100: **^{13}C NMR** (126 MHz, $CDCl_3$, 298 K) spectrum of compound **3q**

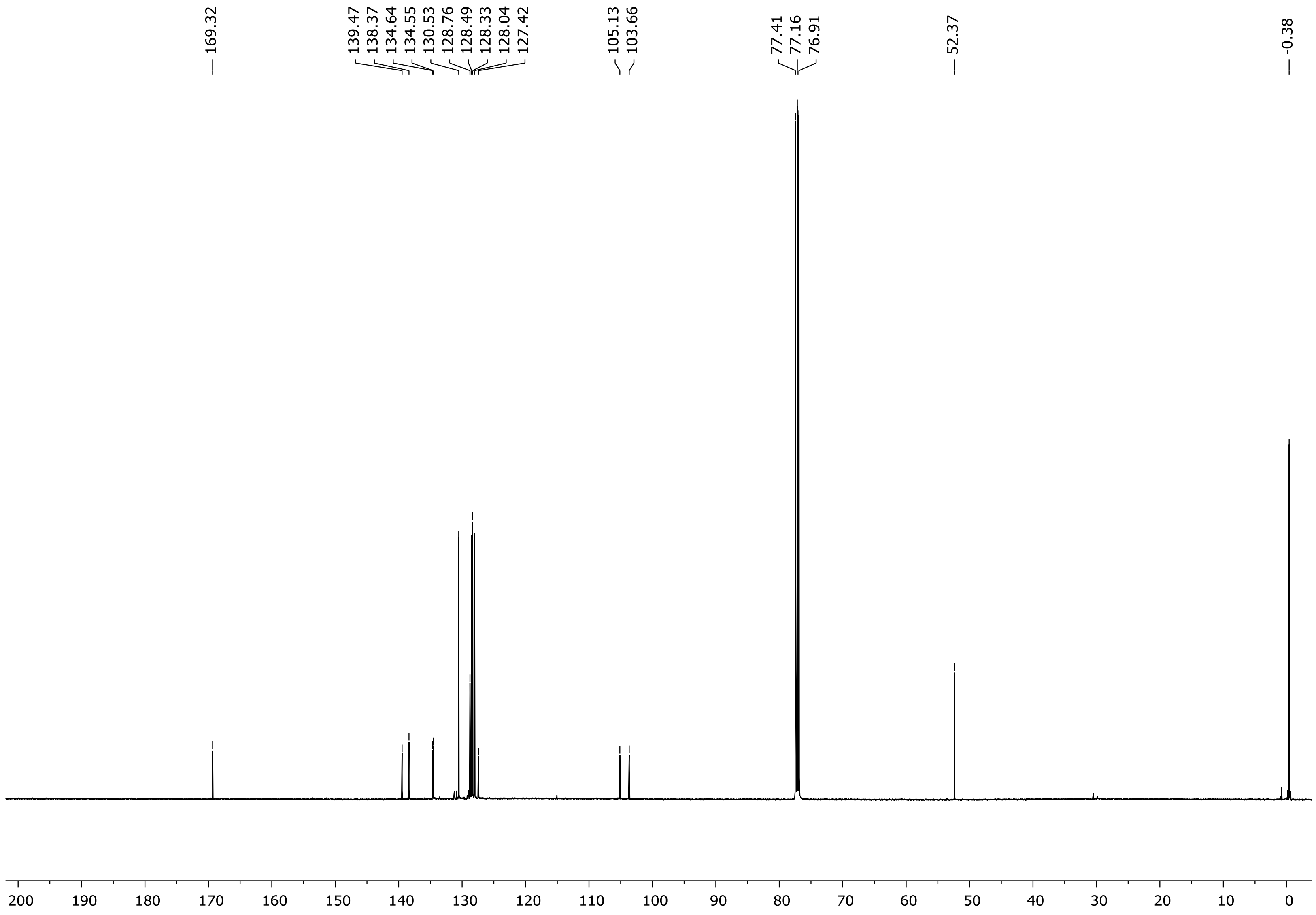

Figure S101: **^{1}H NMR** (500 MHz, $CDCl_3$, 298 K) spectrum of compound **3q'**

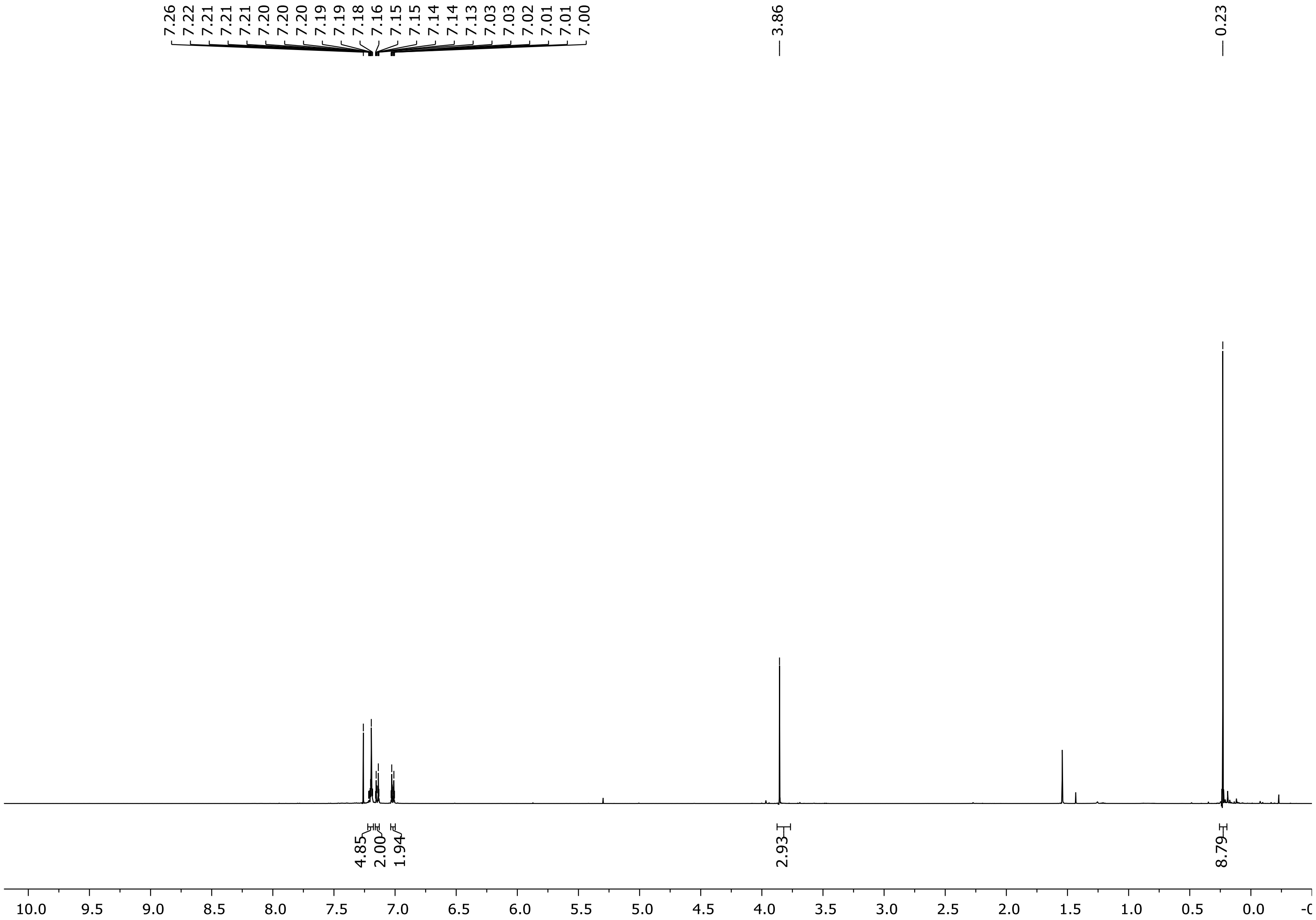

Figure S102: ^{13}**C NMR** (126 MHz, $CDCl_3$, 298 K) spectrum of compound **3q'**

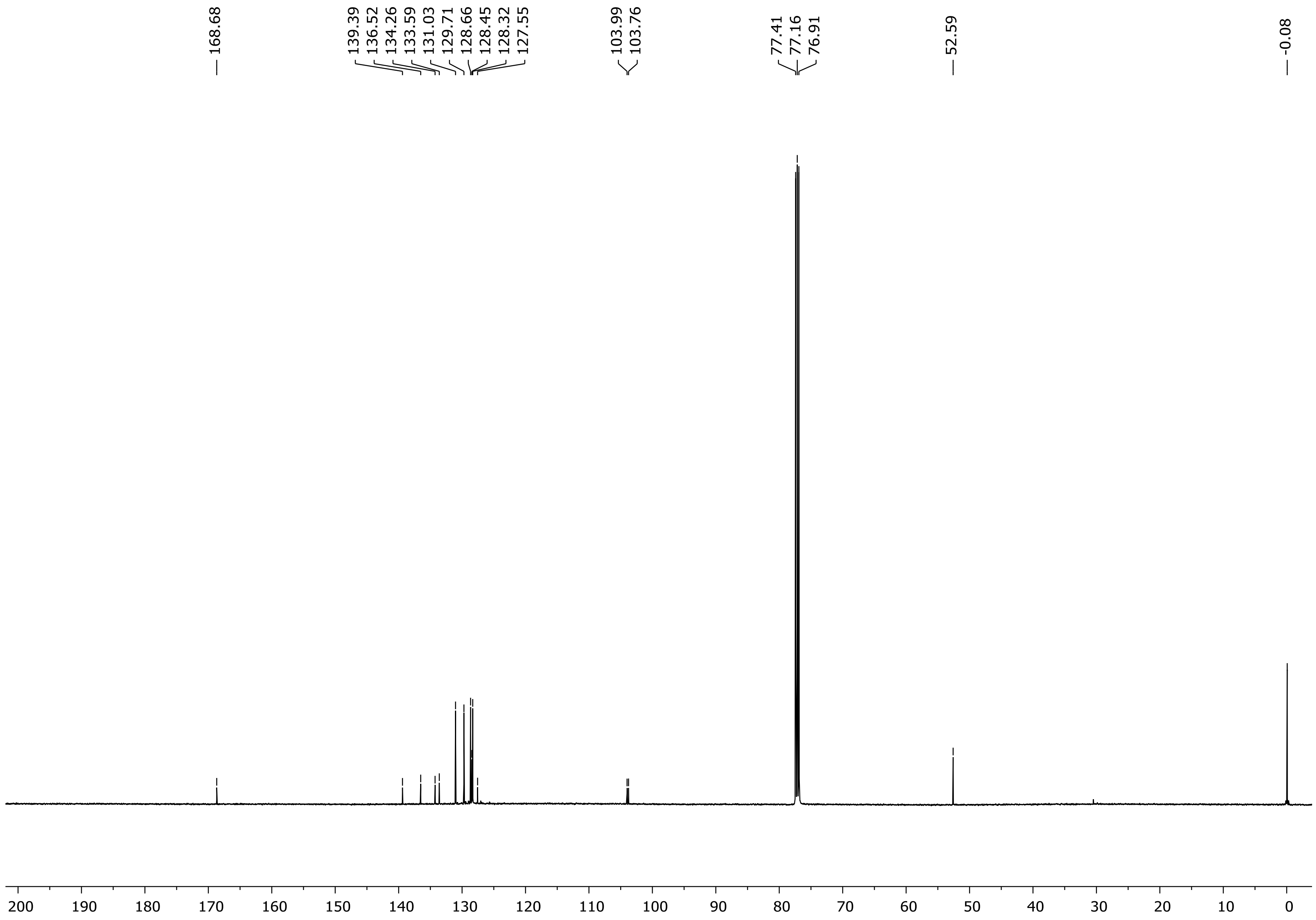

Figure S103: **^{1}H NMR** (500 MHz, $CDCl_3$, 298 K) spectrum of compound **3r**

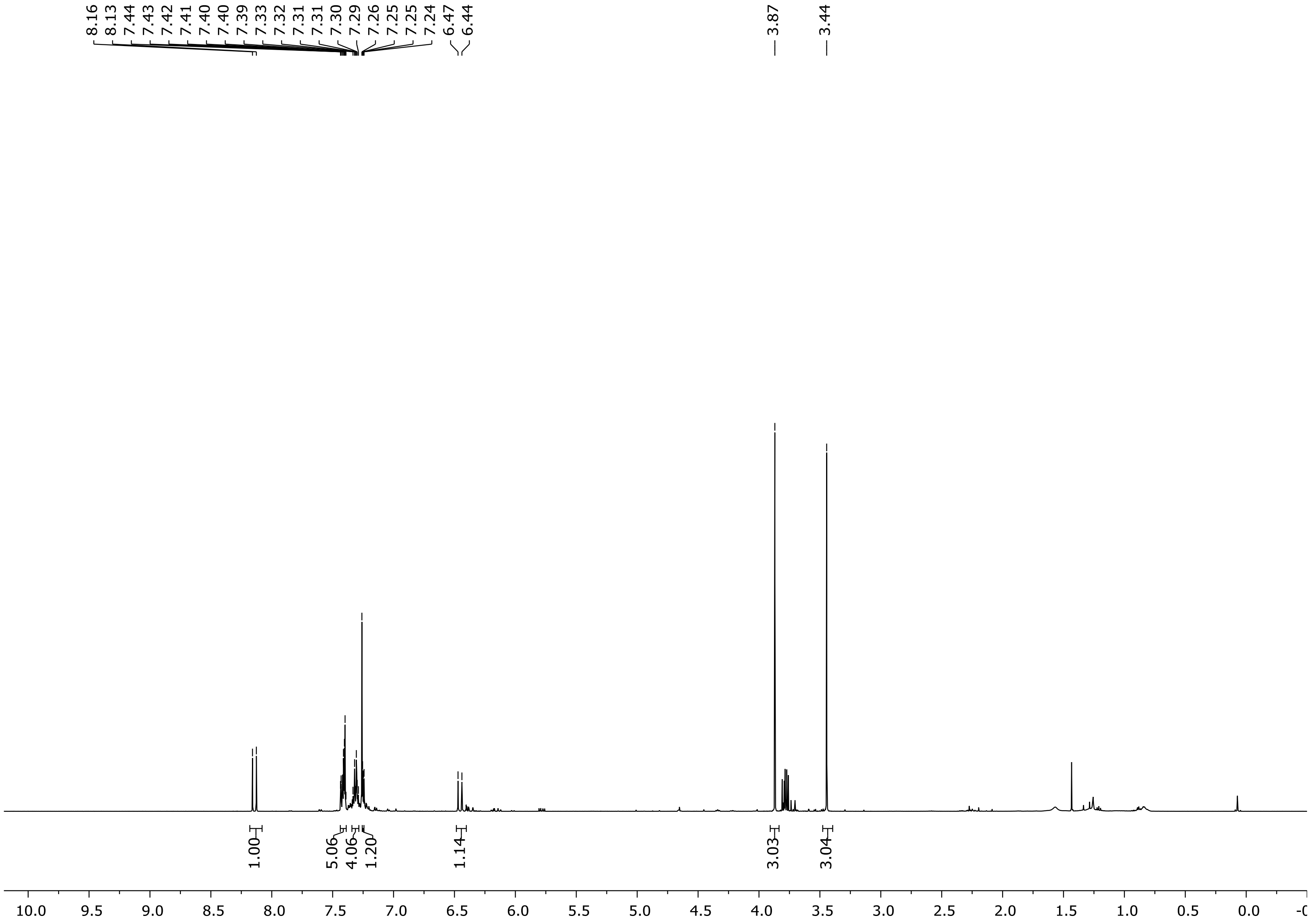

Figure S104: **^{13}C NMR** (126 MHz, $CDCl_3$, 298 K) spectrum of compound **3r**

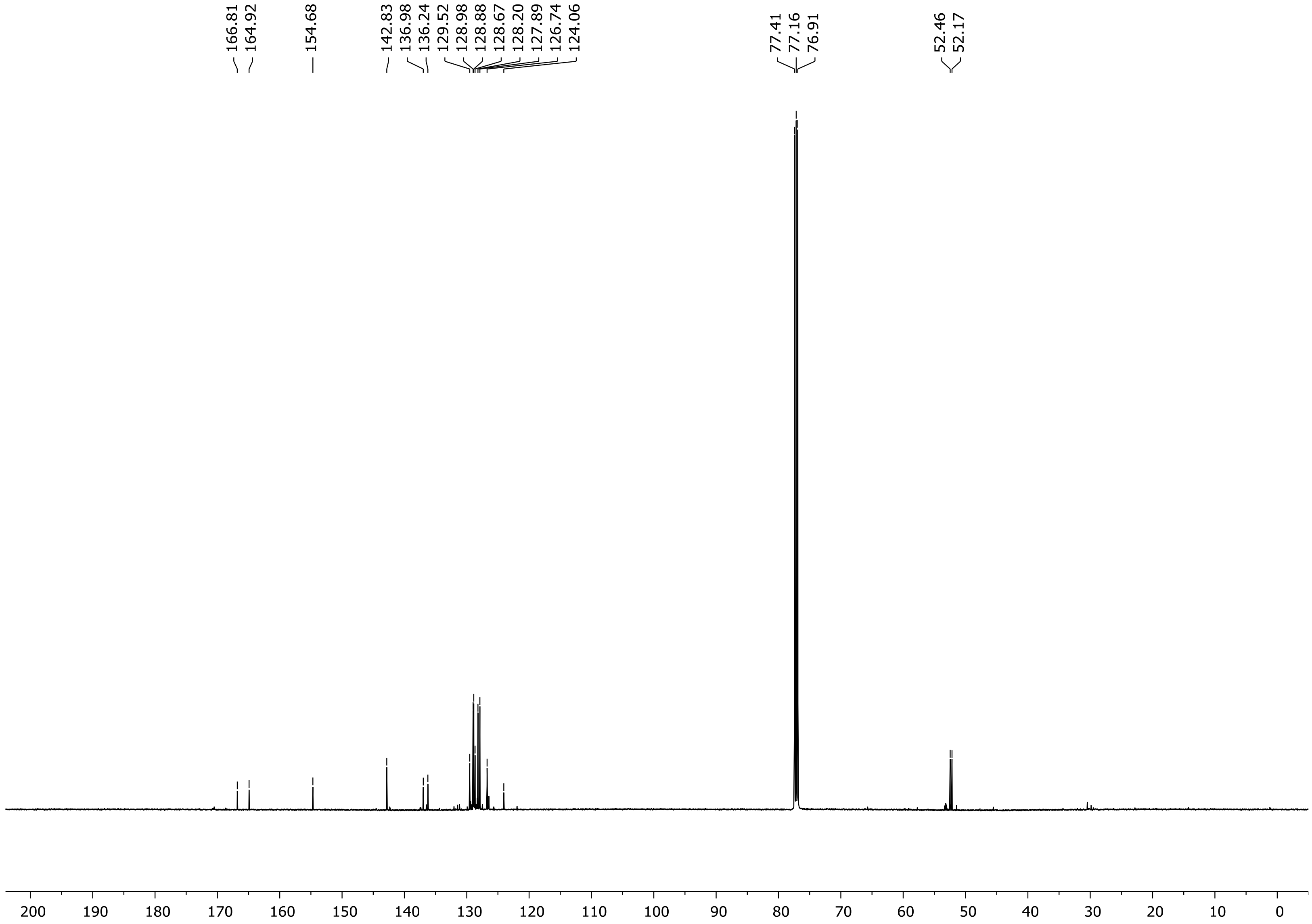

Figure S105: **^{1}H NMR** (500 MHz, $CDCl_3$, 298 K) spectrum of compound **3s**

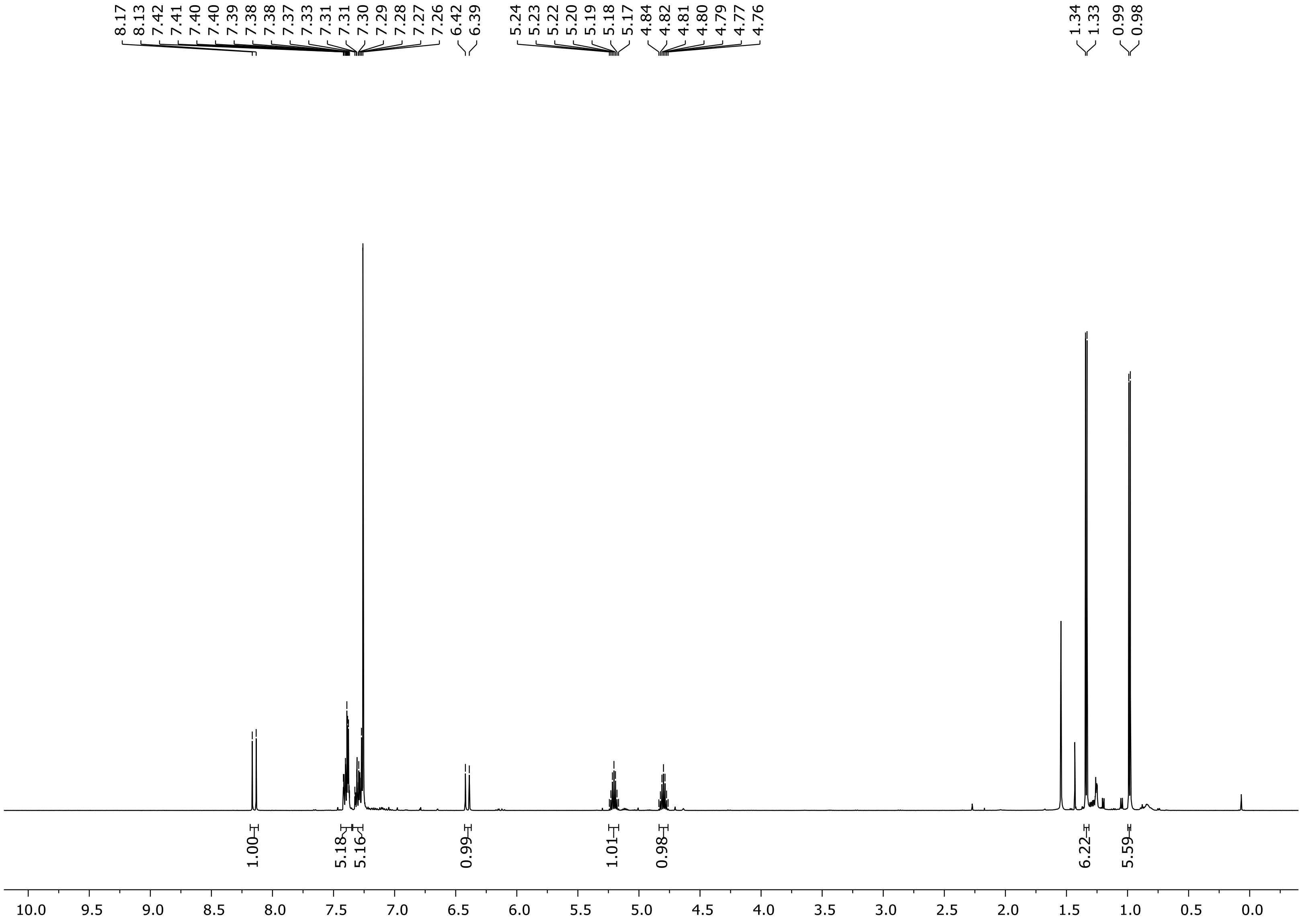

Figure S106: **^{13}C NMR** (126 MHz, $CDCl_3$, 298 K) spectrum of compound **3s**

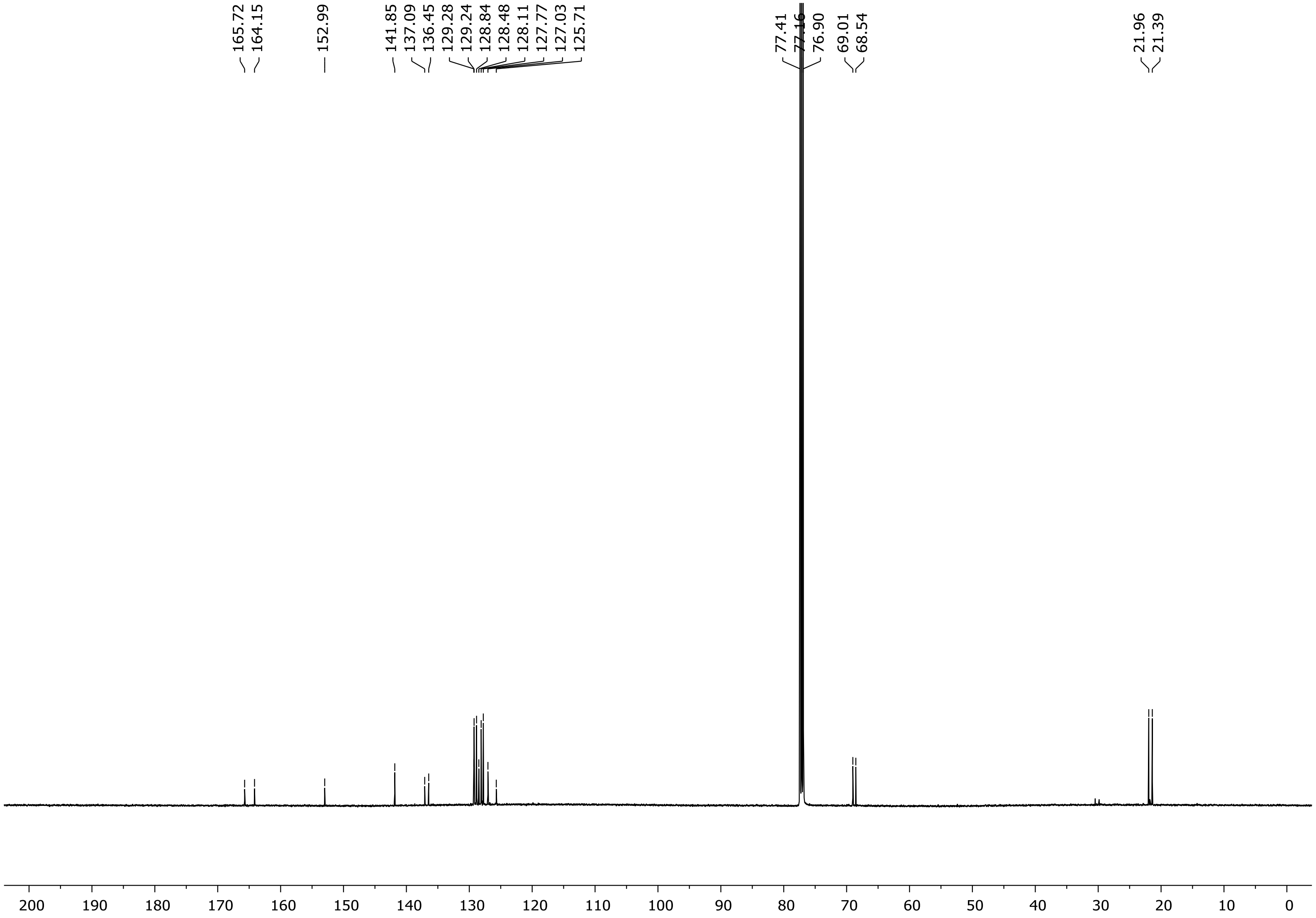

Figure S107: **¹H NMR** (500 MHz, $CDCl_3$, 298 K) spectrum of compound **4a**

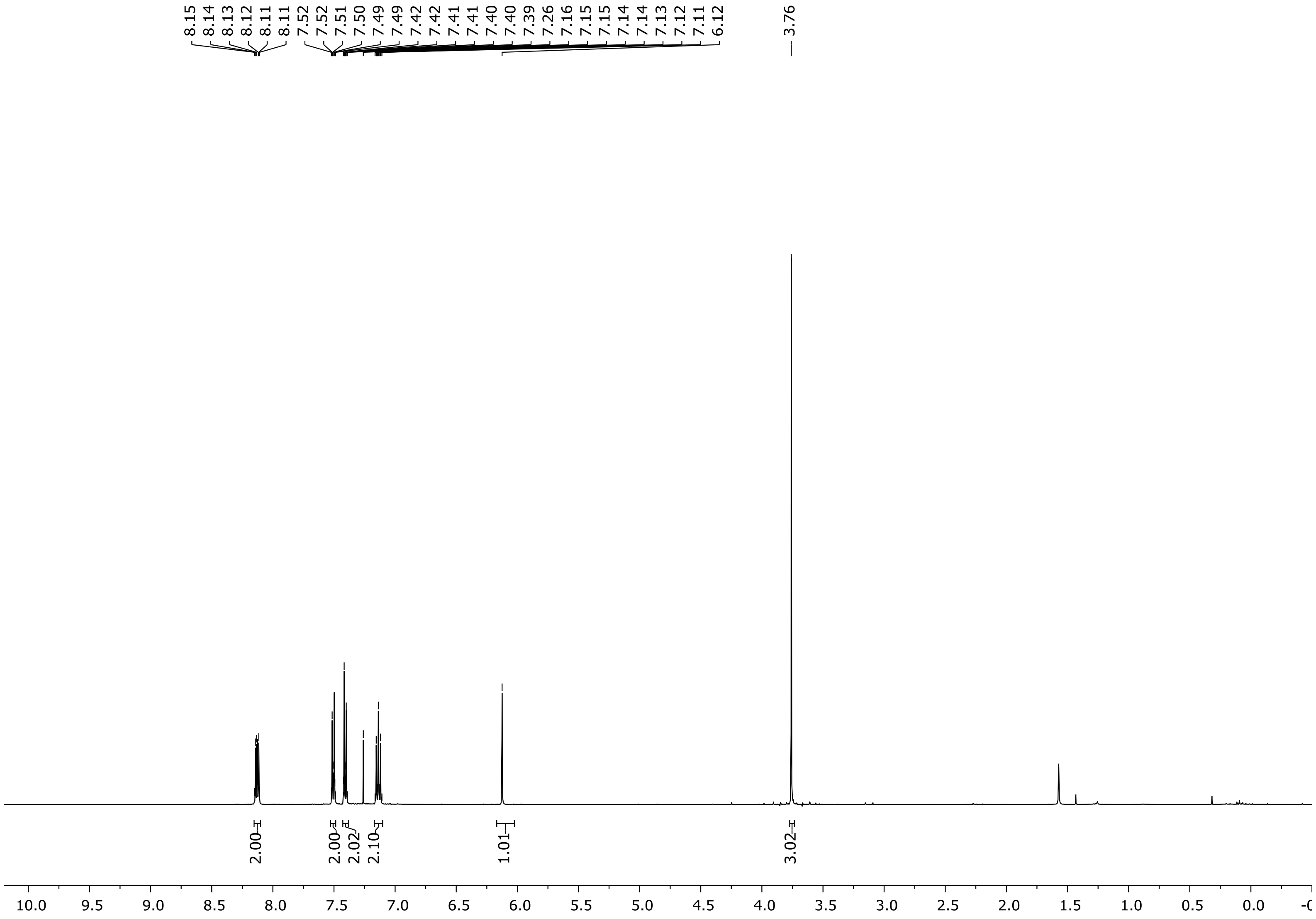

Figure S108: **^{13}C NMR** (126 MHz, $CDCl_3$, 298 K) spectrum of compound **4a**

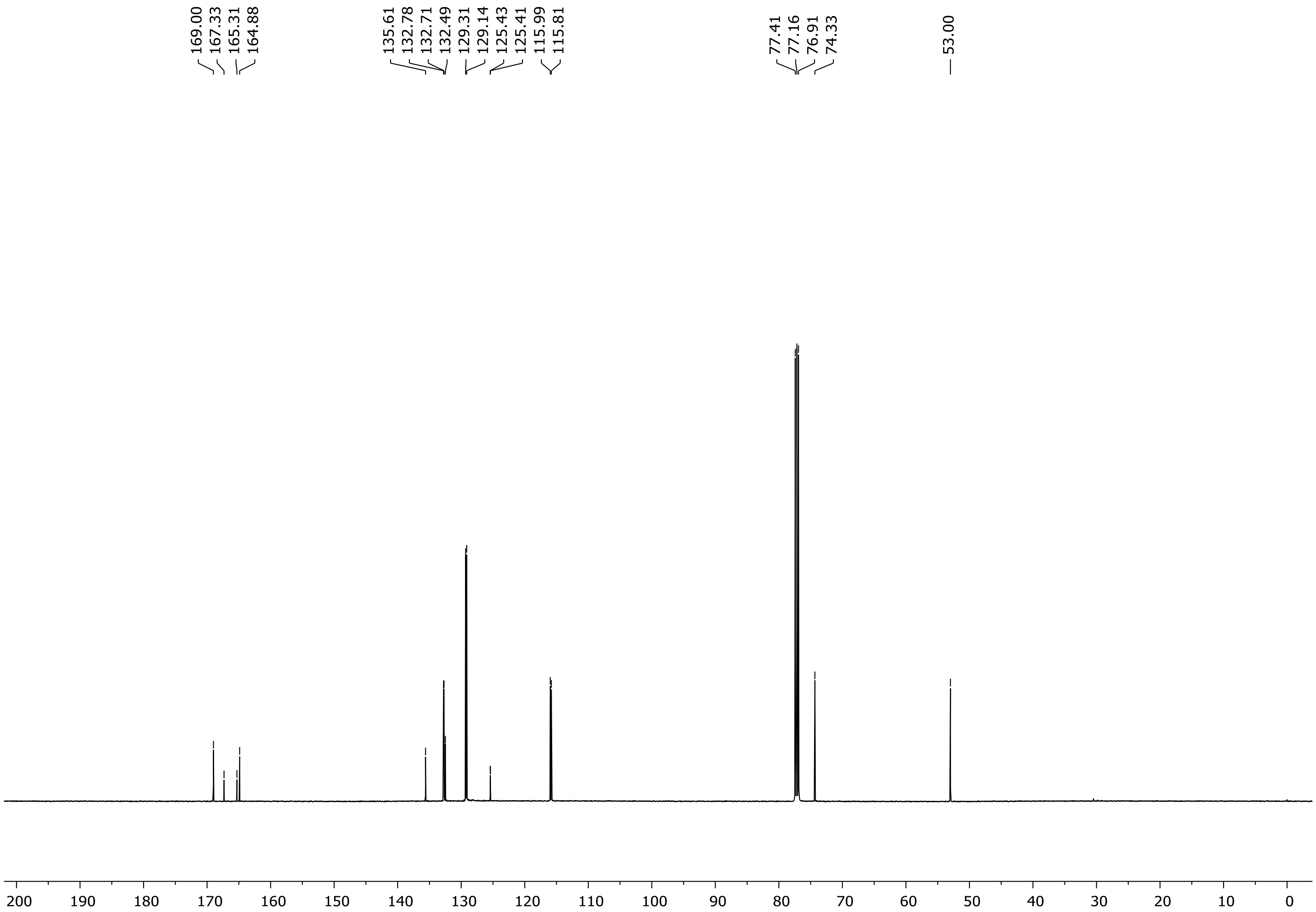

Figure S109: ^{19}**F NMR** (471 MHz, $CDCl_3$, 298 K) spectrum of compound **4a**

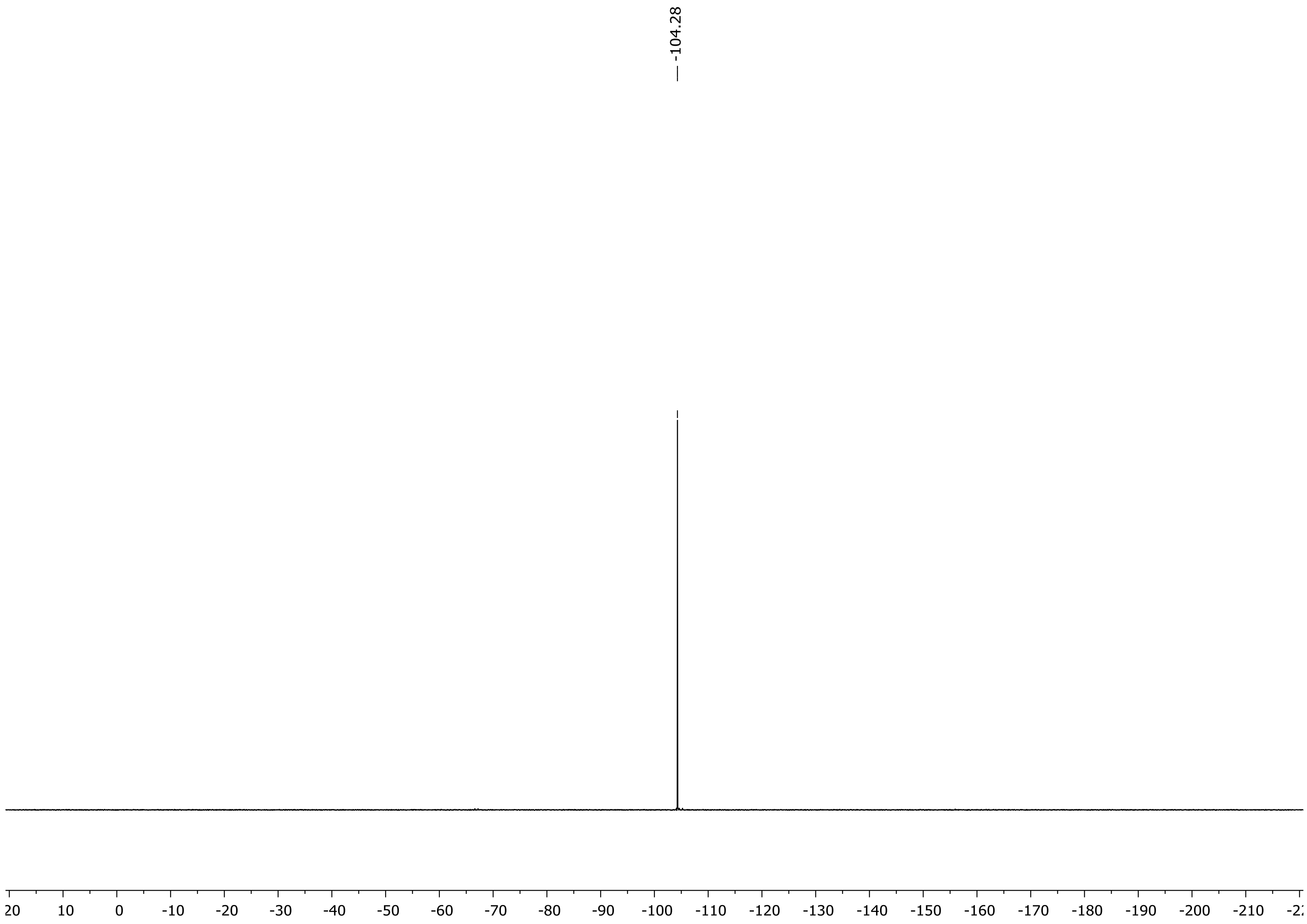

Figure S110: **^{1}H NMR** (500 MHz, $CDCl_3$, 298 K) spectrum of compound **4b**

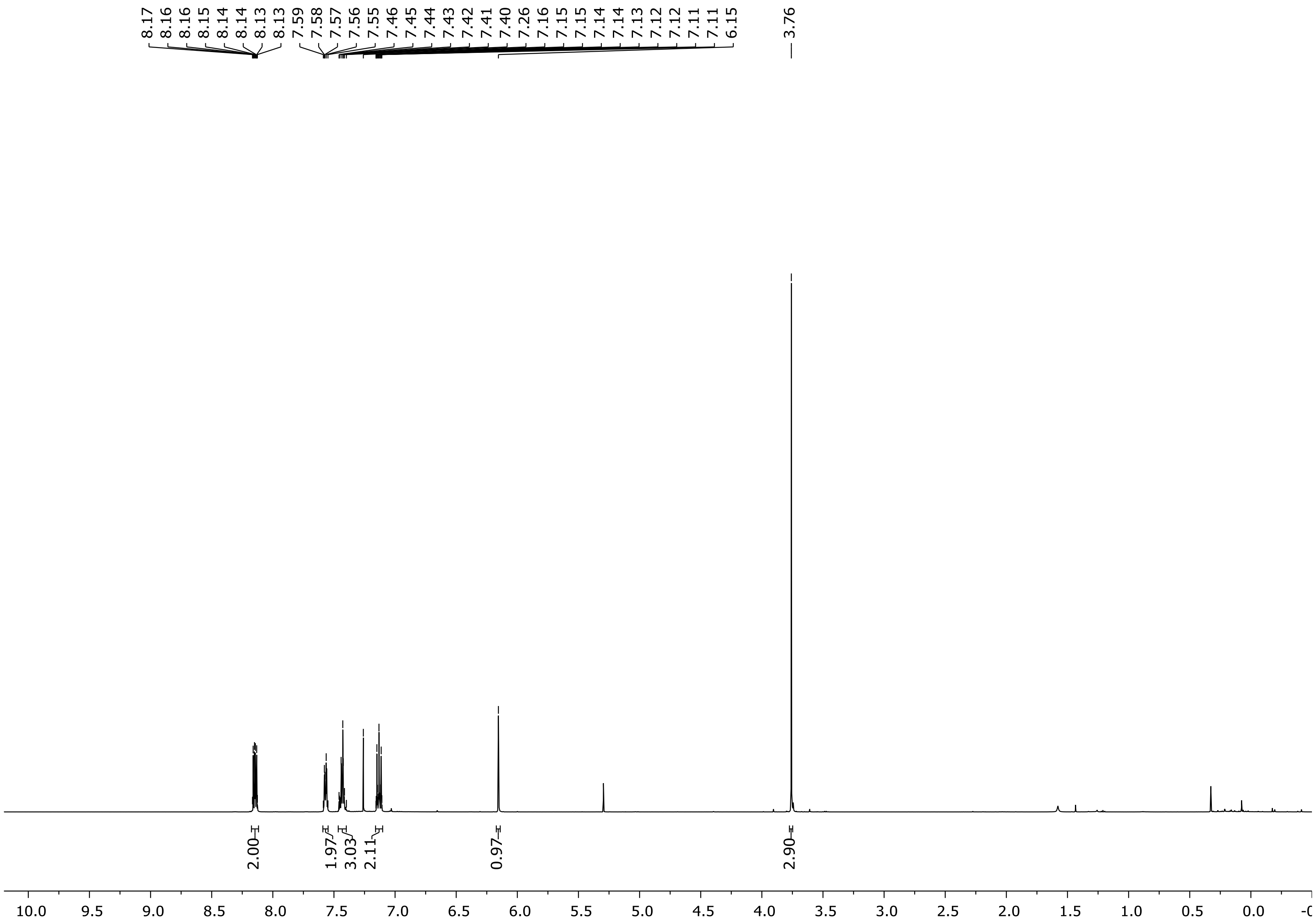

Figure S111: **^{13}C NMR** (126 MHz, $CDCl_3$, 298 K) spectrum of compound **4b**

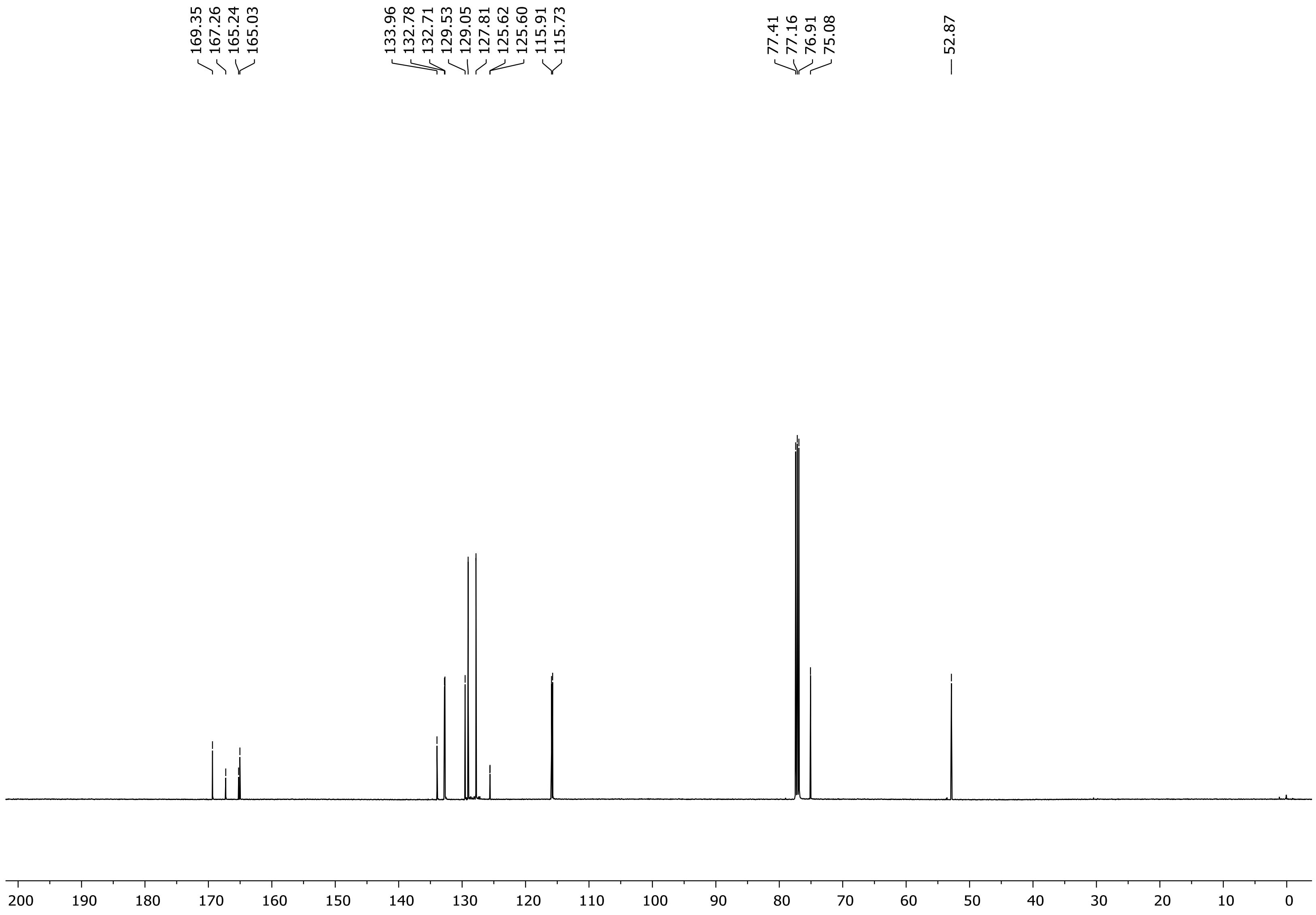

Figure S112: **^{19}F NMR** (471 MHz, $CDCl_3$, 298 K) spectrum of compound **4b**

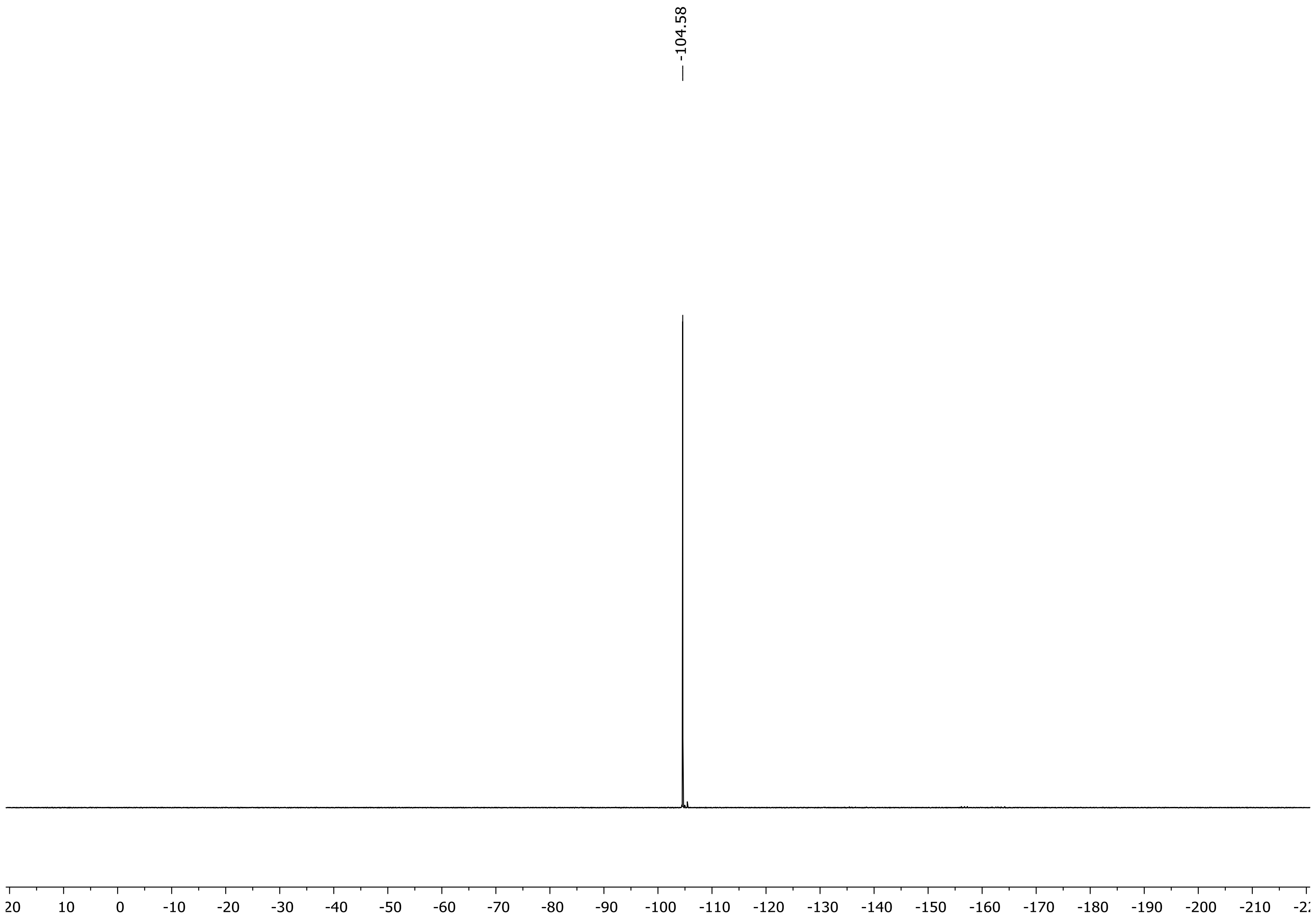

Figure S113: ^{1}**H NMR** (500 MHz, $CDCl_3$, 298 K) spectrum of compound **6a**

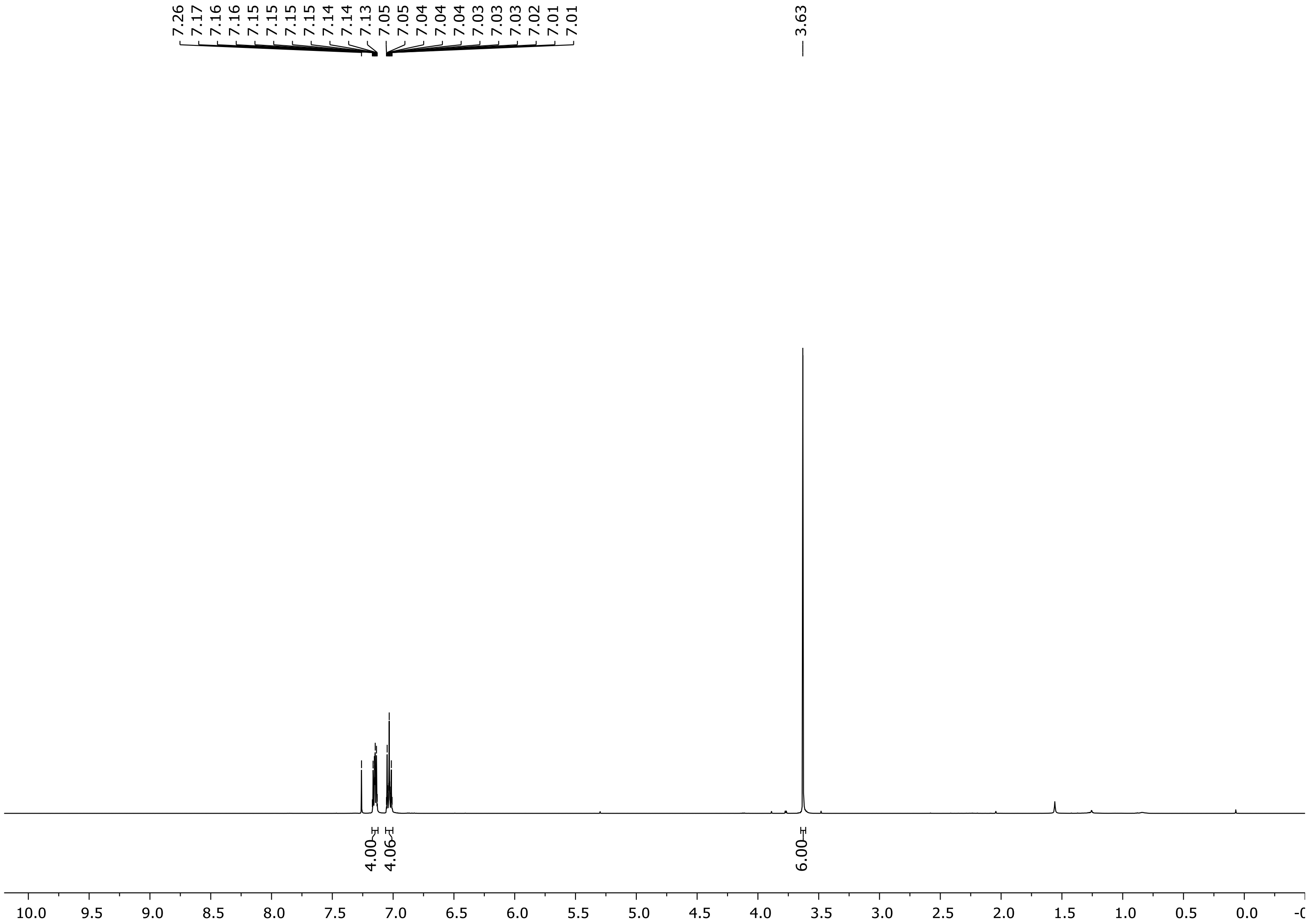

Figure S114: **^{13}C NMR** (126 MHz, $CDCl_3$, 298 K) spectrum of compound **6a**

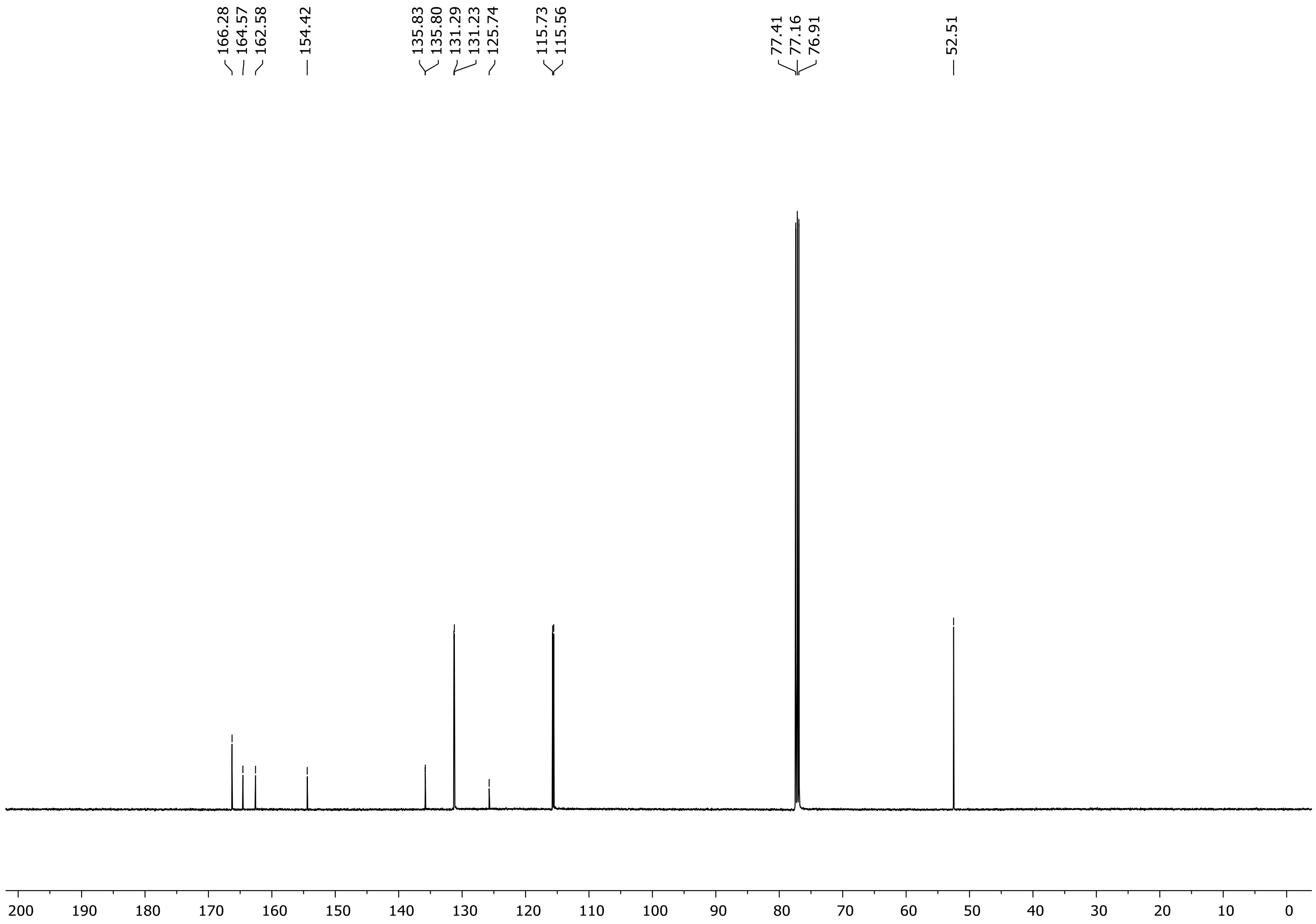

Figure S115: [19]**F NMR** (471 MHz, $CDCl_3$, 298 K) spectrum of compound **6a**

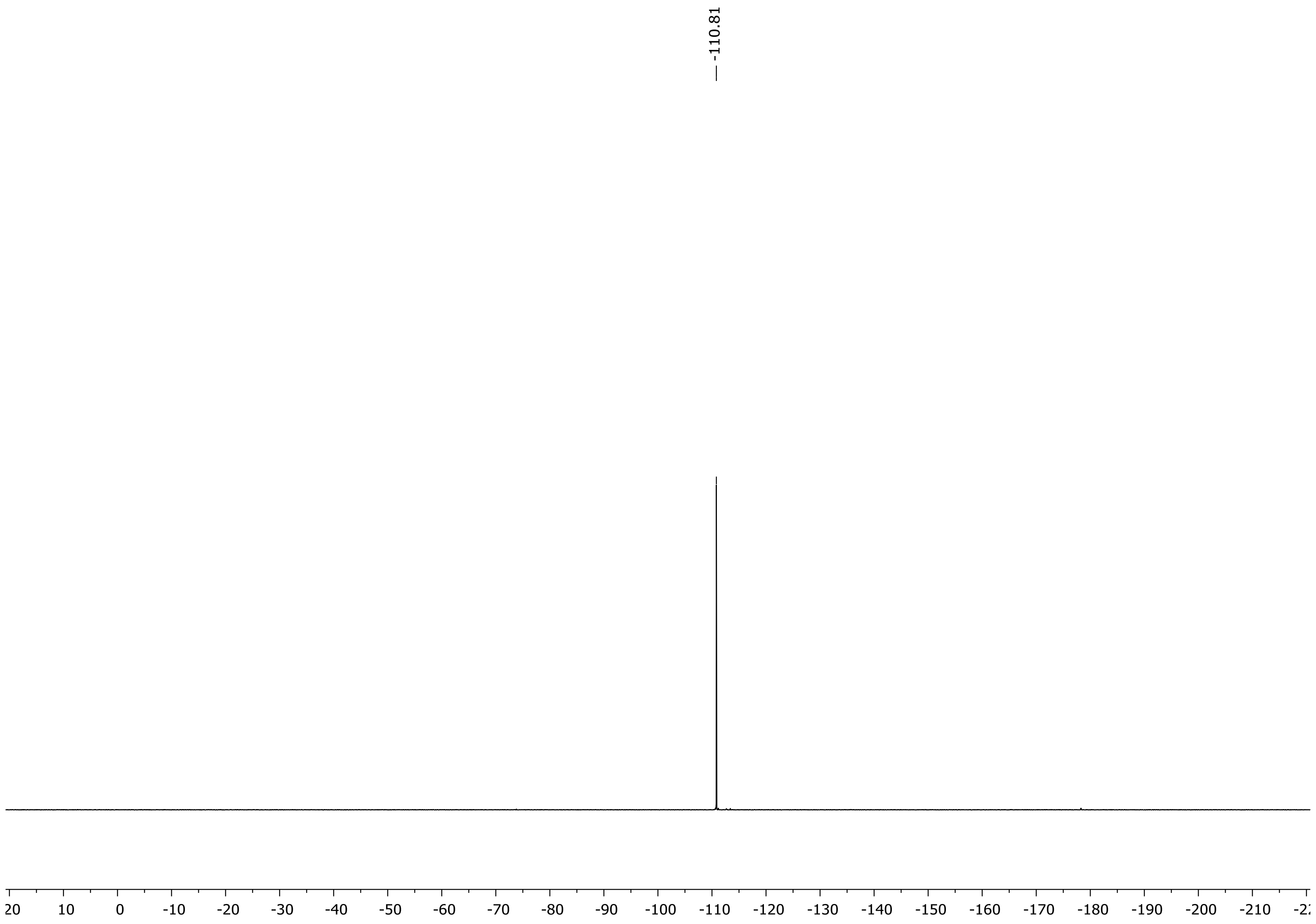

Figure S116: **^{1}H NMR** (500 MHz, $CDCl_3$, 298 K) spectrum of compound **6b**

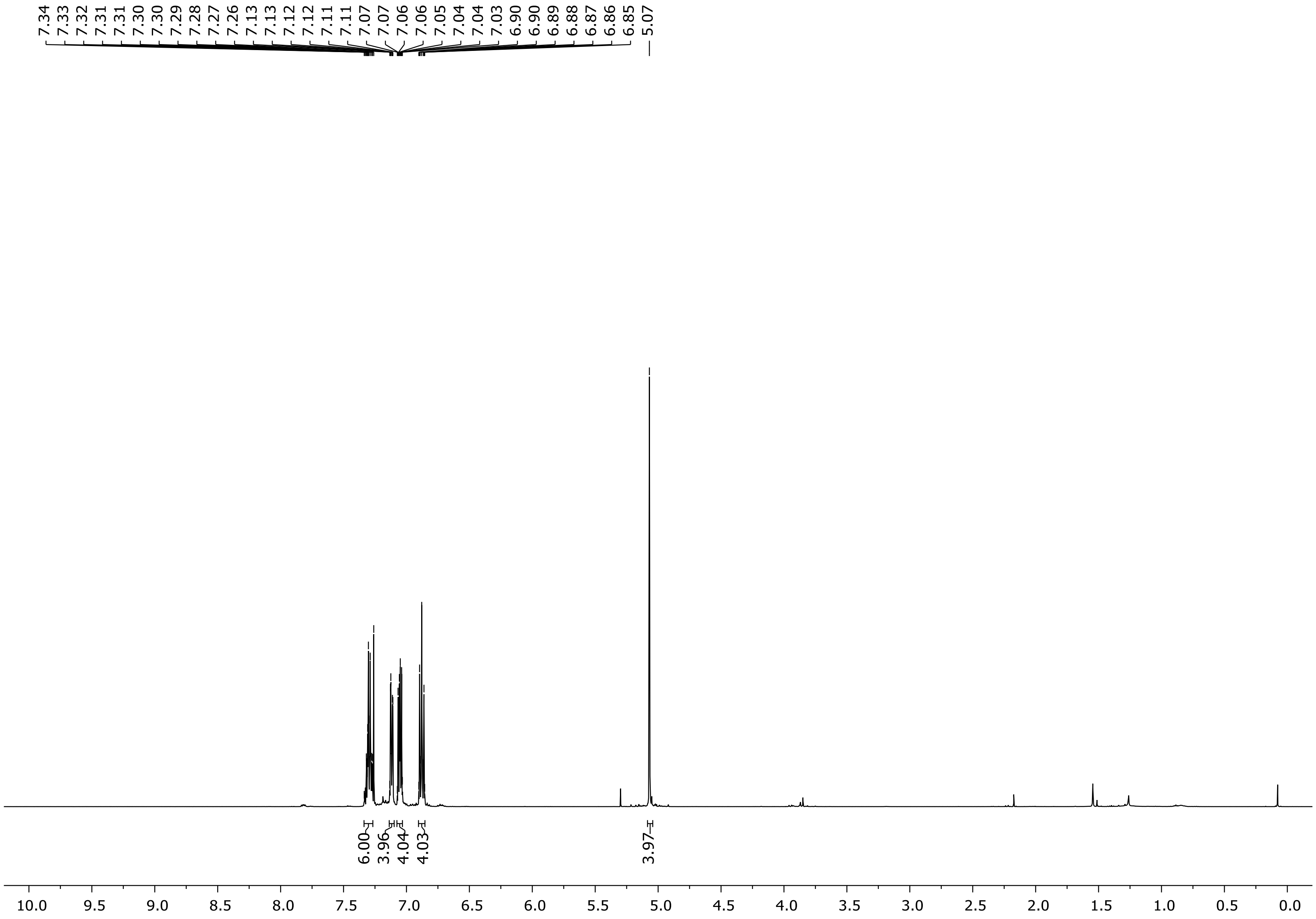

Figure S117: **^{13}C NMR** (126 MHz, $CDCl_3$, 298 K) spectrum of compound **6b**

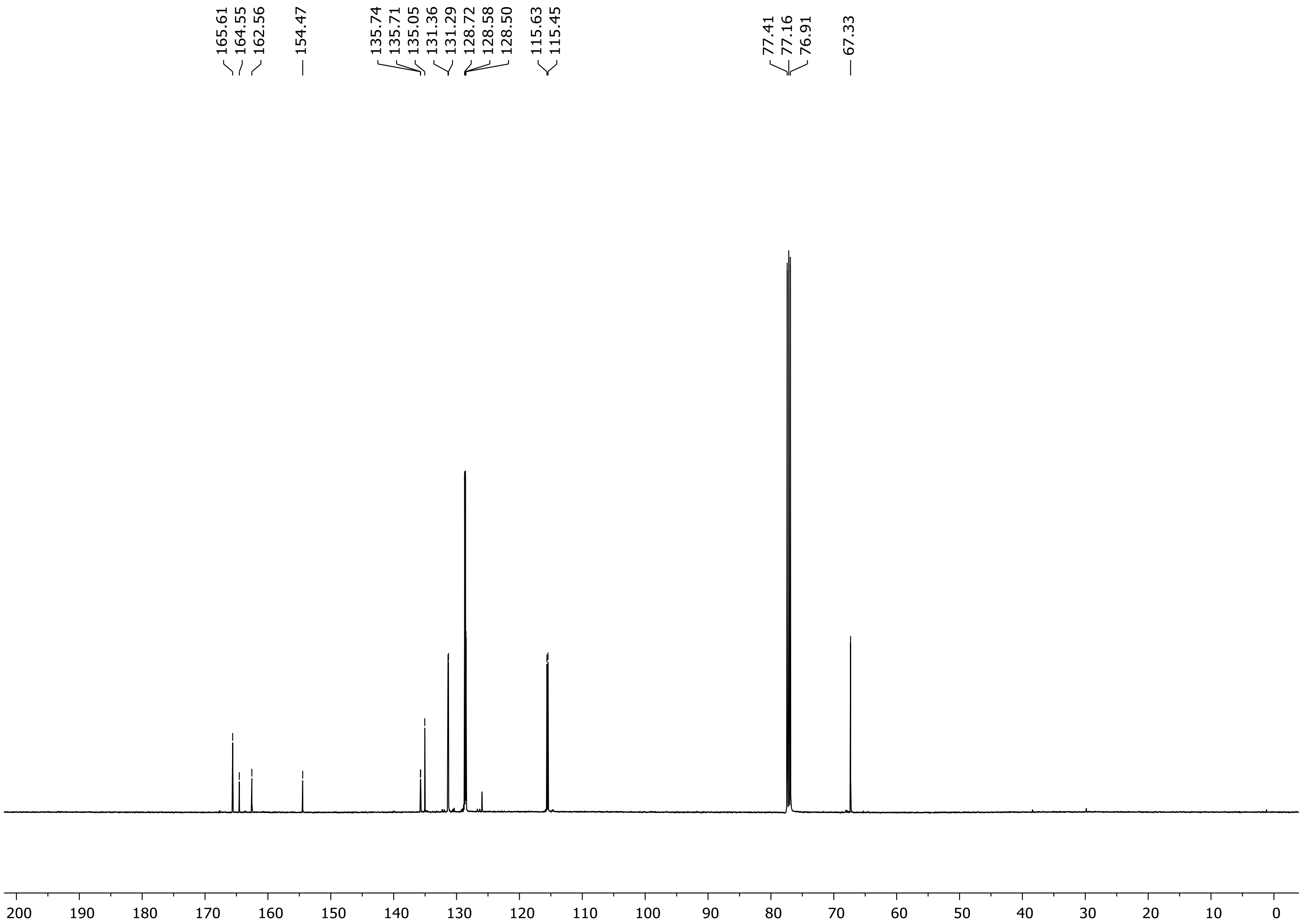

Figure S118: ^{19}**F NMR** (471 MHz, $CDCl_3$, 298 K) spectrum of compound **6b**

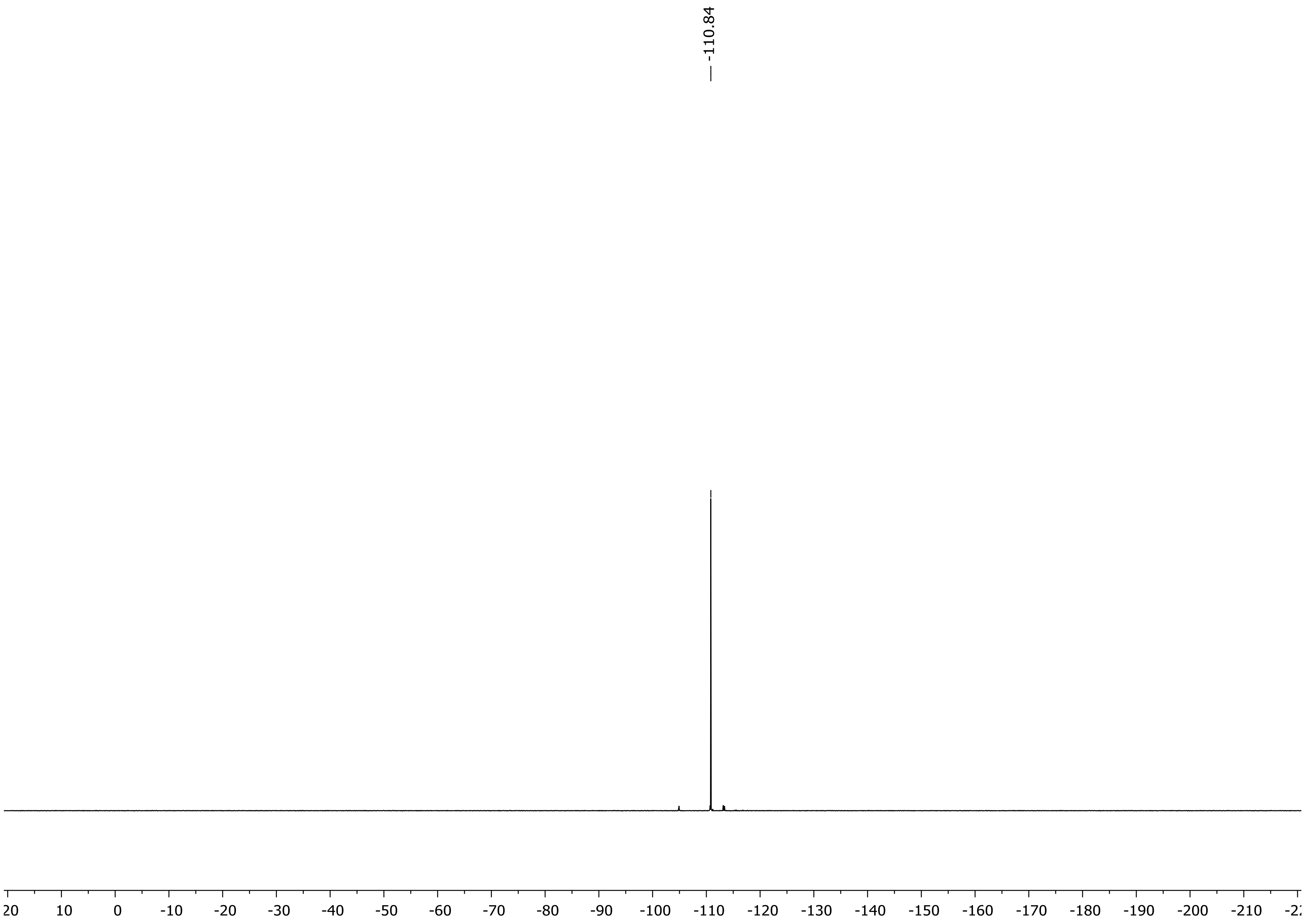

Figure S119: **^{1}H NMR** (500 MHz, $CDCl_3$, 298 K) spectrum of compound **6c**

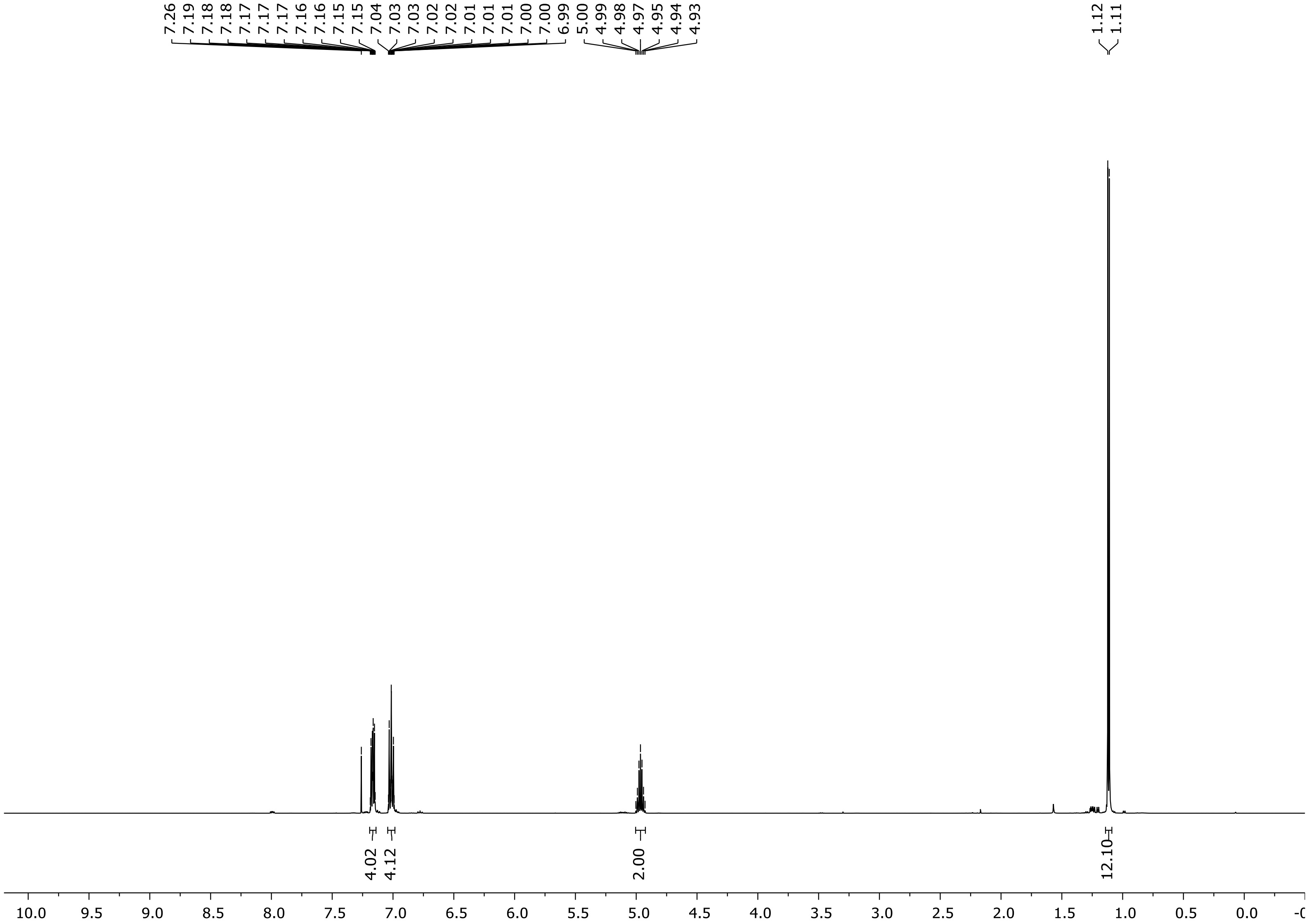

Figure S120: **^{13}C NMR** (126 MHz, $CDCl_3$, 298 K) spectrum of compound **6c**

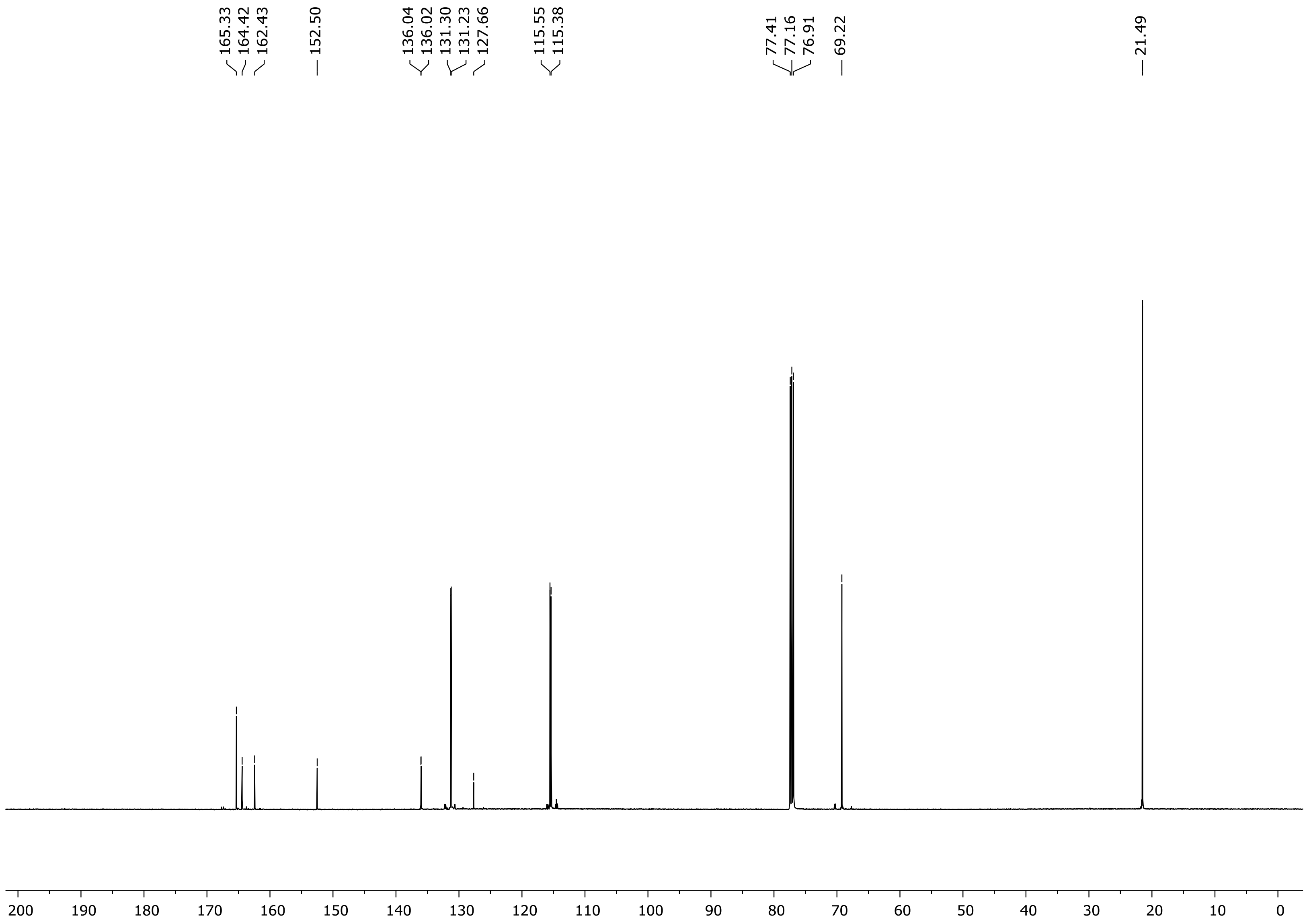

Figure S121: [19]**F NMR** (471 MHz, $CDCl_3$, 298 K) spectrum of compound **6c**

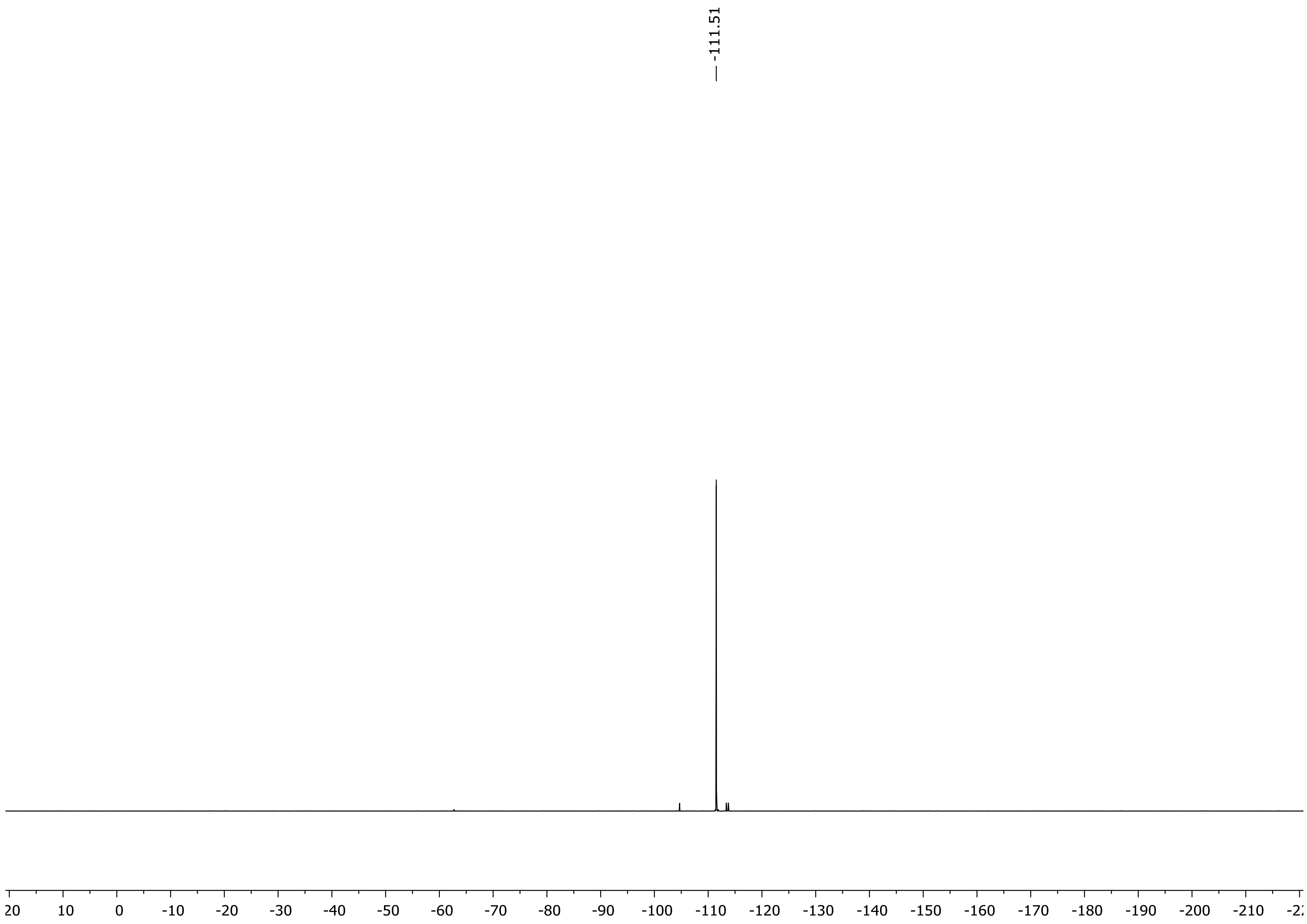

Figure S122: **^{1}H NMR** (500 MHz, $CDCl_3$, 298 K) spectrum of compound **6d**

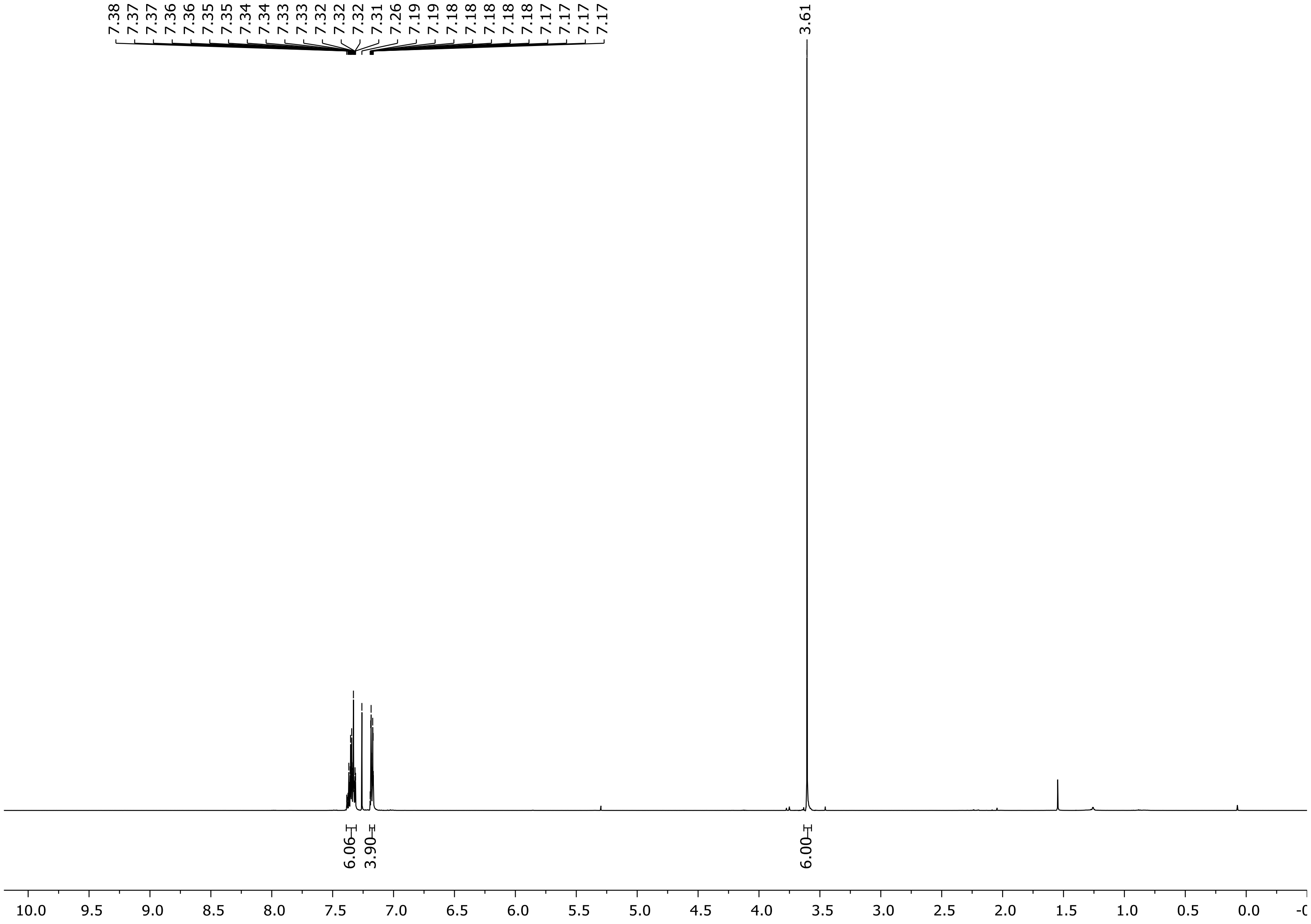

Figure S123: **^{13}C NMR** (126 MHz, $CDCl_3$, 298 K) spectrum of compound **6d**

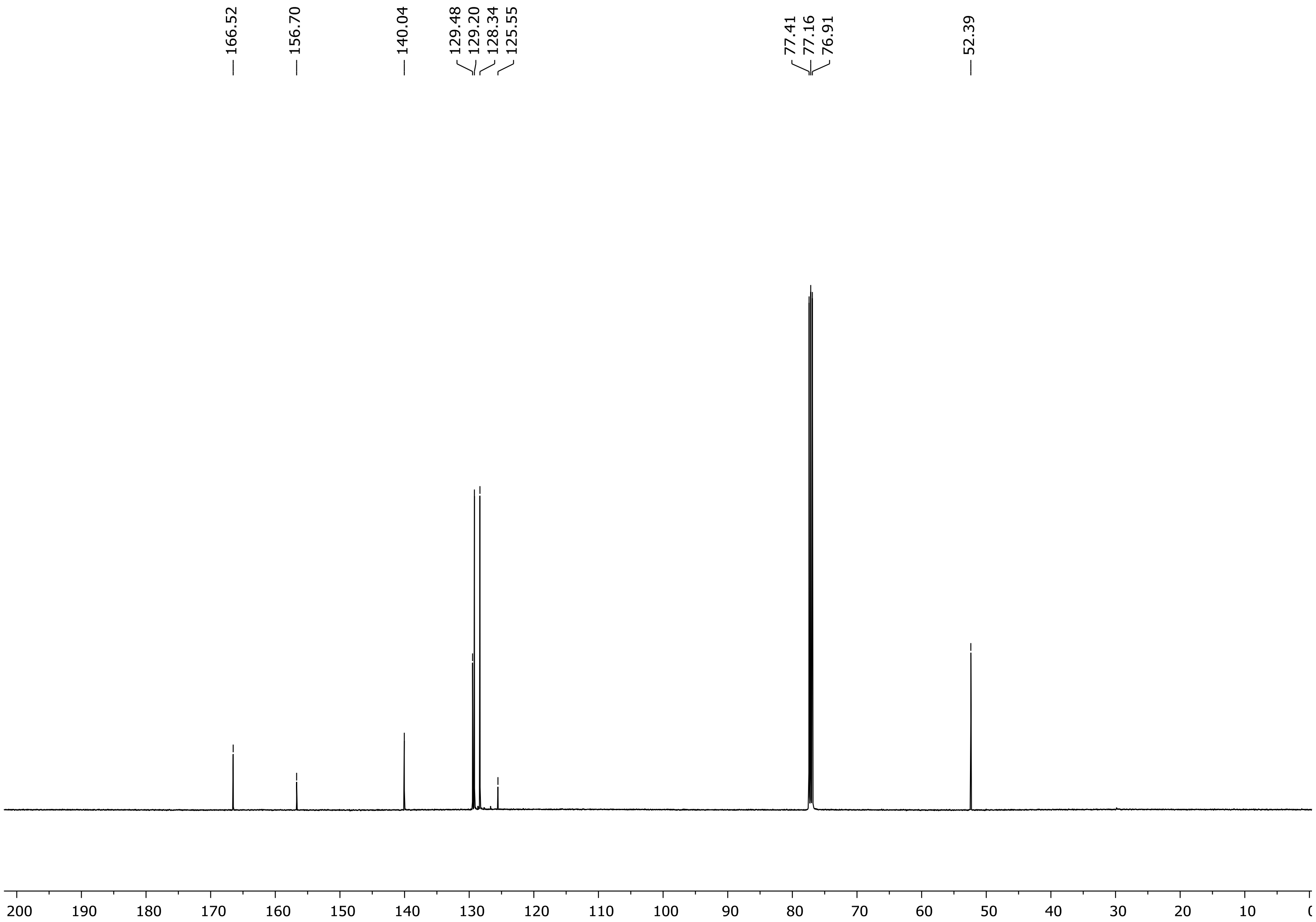

Figure S124: **^{1}H NMR** (500 MHz, $CDCl_3$, 298 K) spectrum of compound **6e**

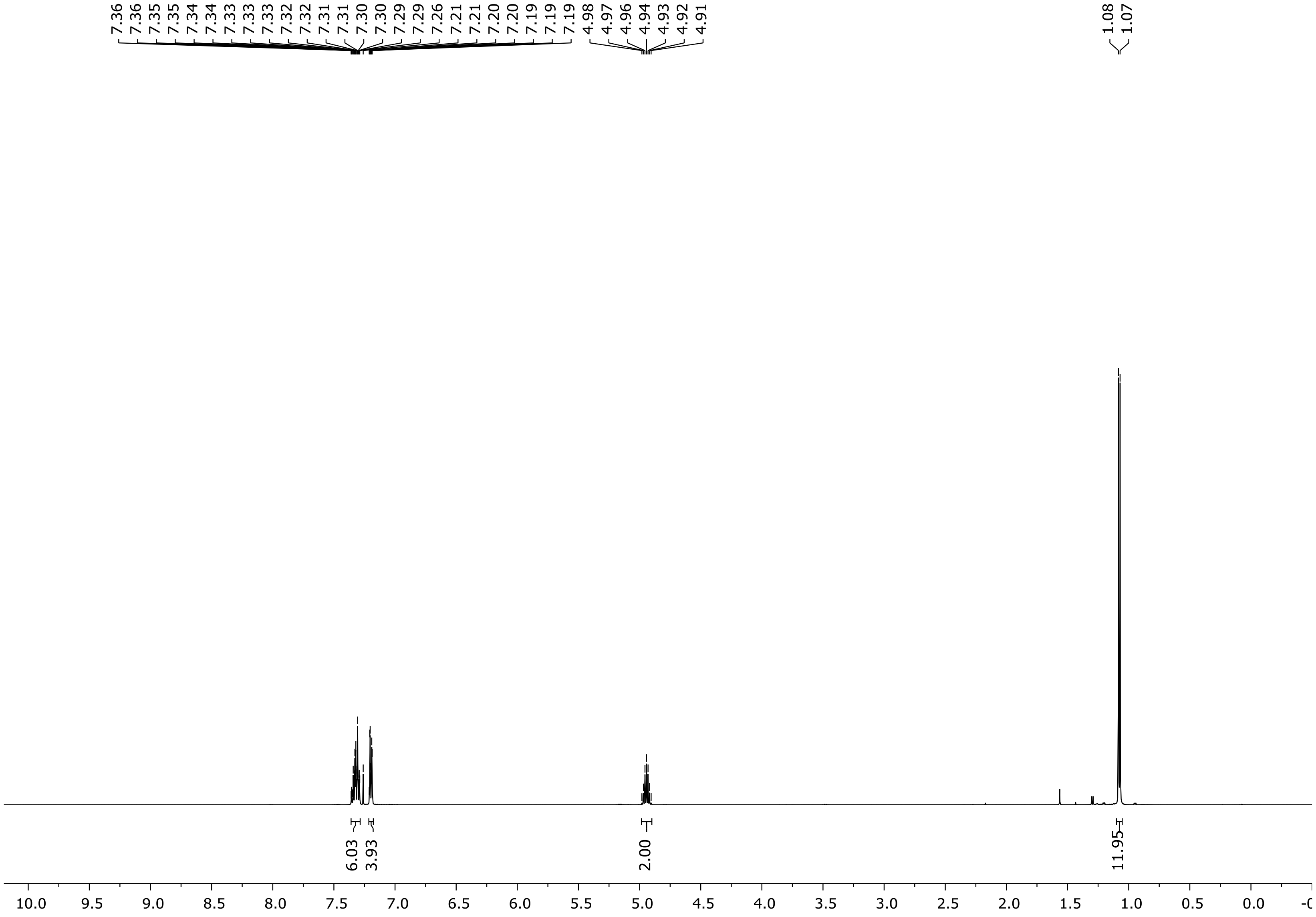

Figure S125: ^{13}**C NMR** (126 MHz, $CDCl_3$, 298 K) spectrum of compound **6e**

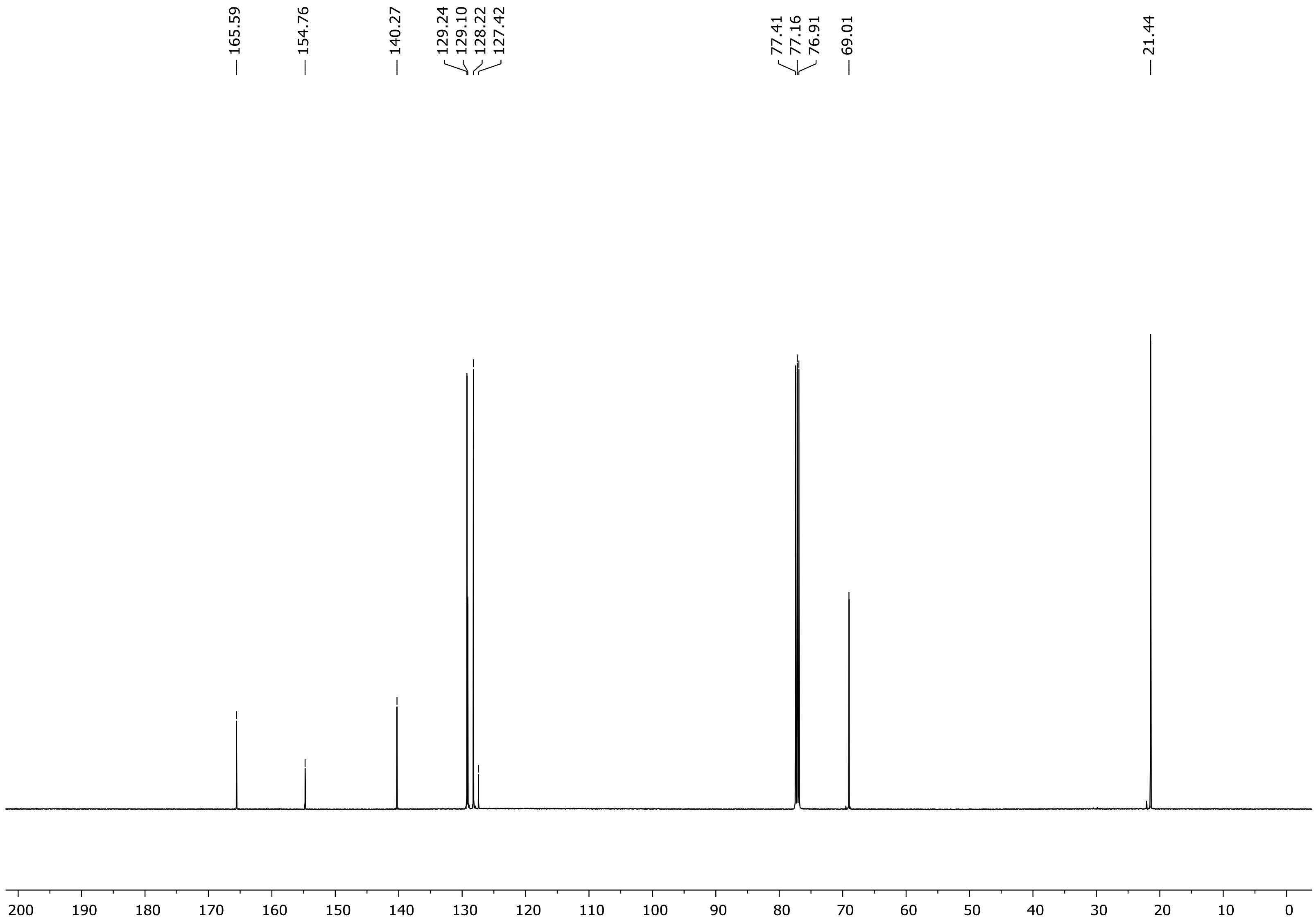

Figure S126: **^{1}H NMR** (500 MHz, $CDCl_3$, 298 K) spectrum of compound **6f**

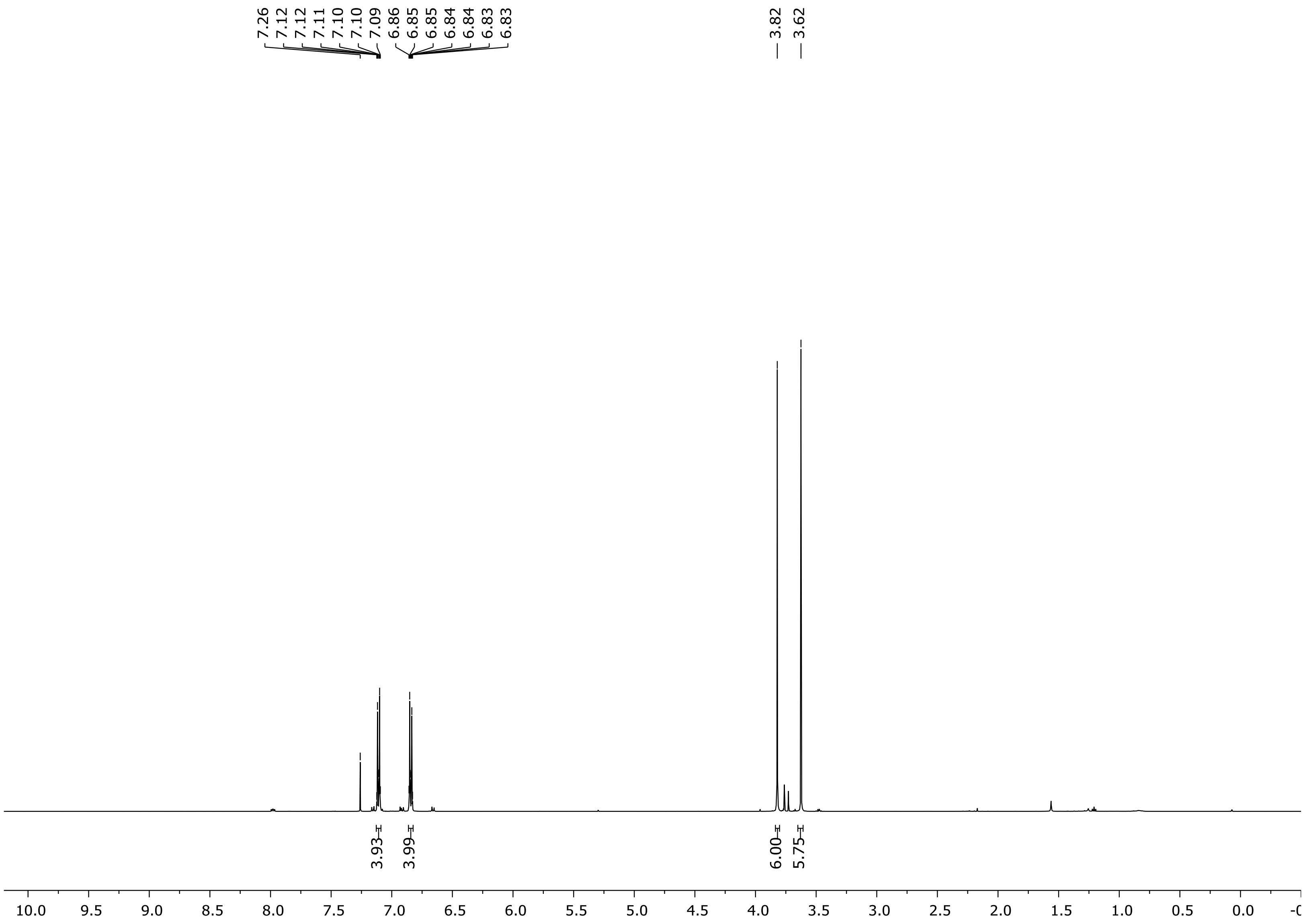

Figure S127: ^{13}**C NMR** (126 MHz, $CDCl_3$, 298 K) spectrum of compound **6f**

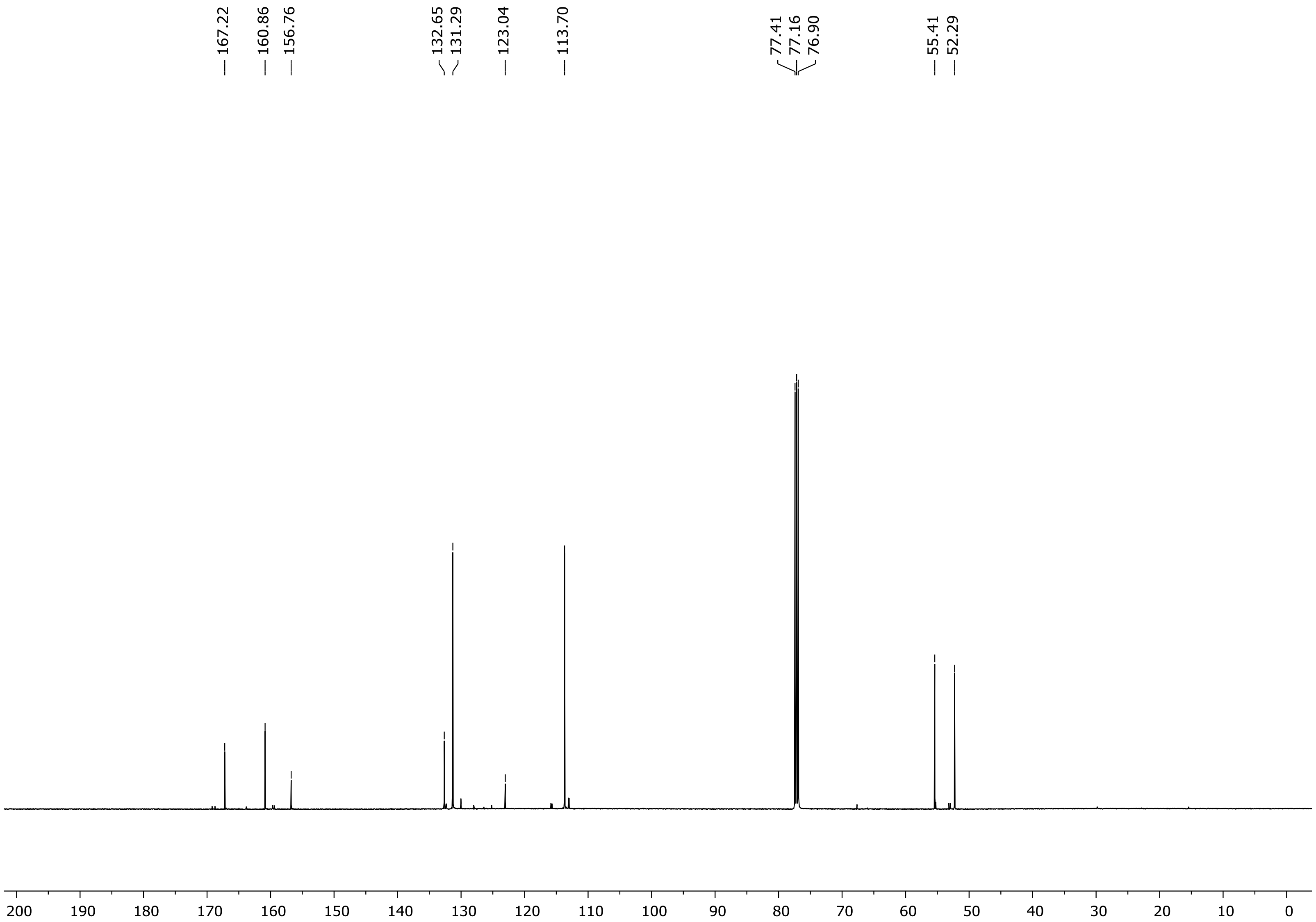

Figure S128: ^{1}**H NMR** (500 MHz, $CDCl_3$, 298 K) spectrum of compound **6g**

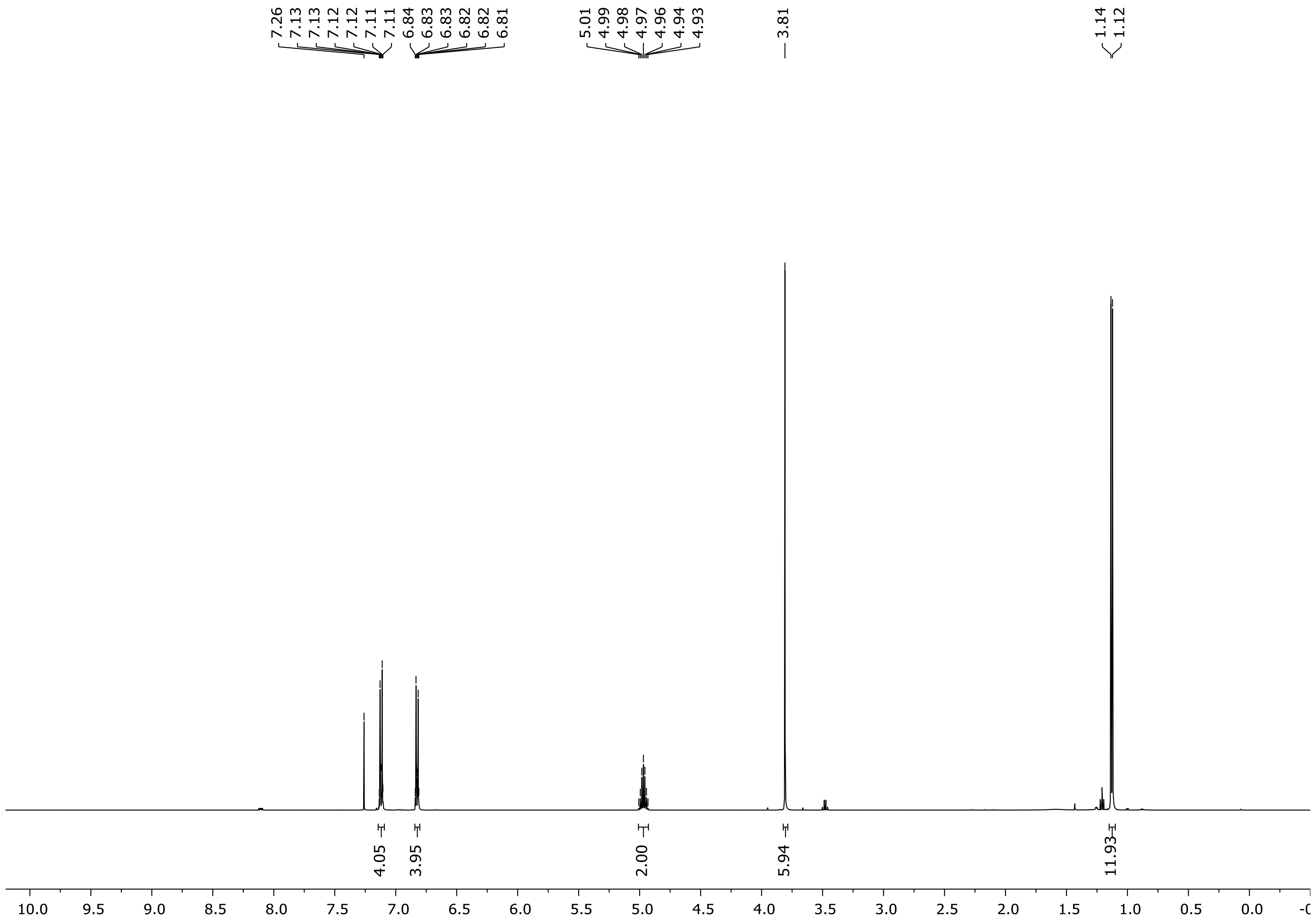

Figure S129: **^{13}C NMR** (126 MHz, $CDCl_3$, 298 K) spectrum of compound **6g**

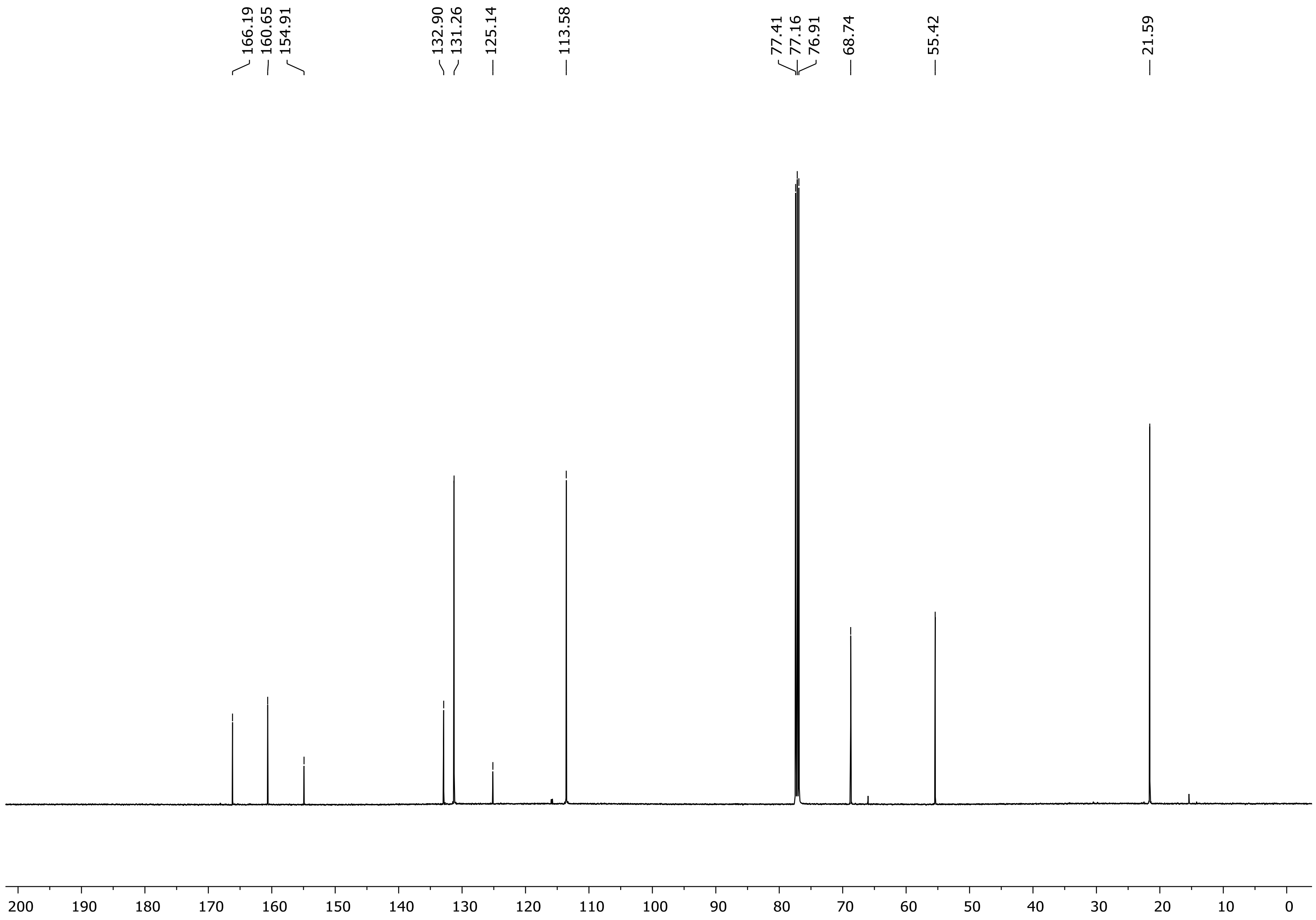

Figure S130: **^{1}H NMR** (500 MHz, $CDCl_3$, 298 K) spectrum of compound **6h**

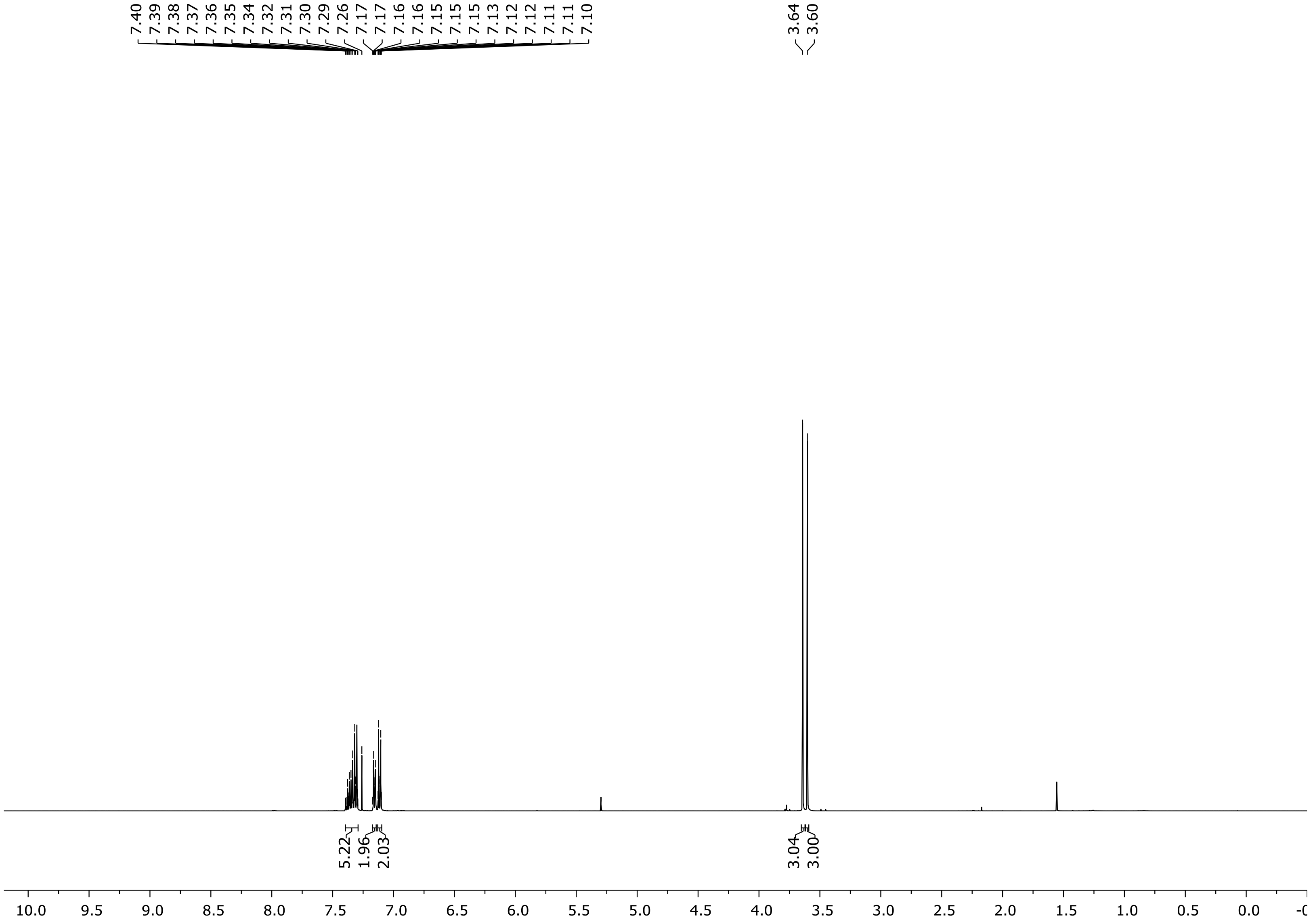

Figure S131: **^{13}C NMR** (126 MHz, $CDCl_3$, 298 K) spectrum of compound **6h**

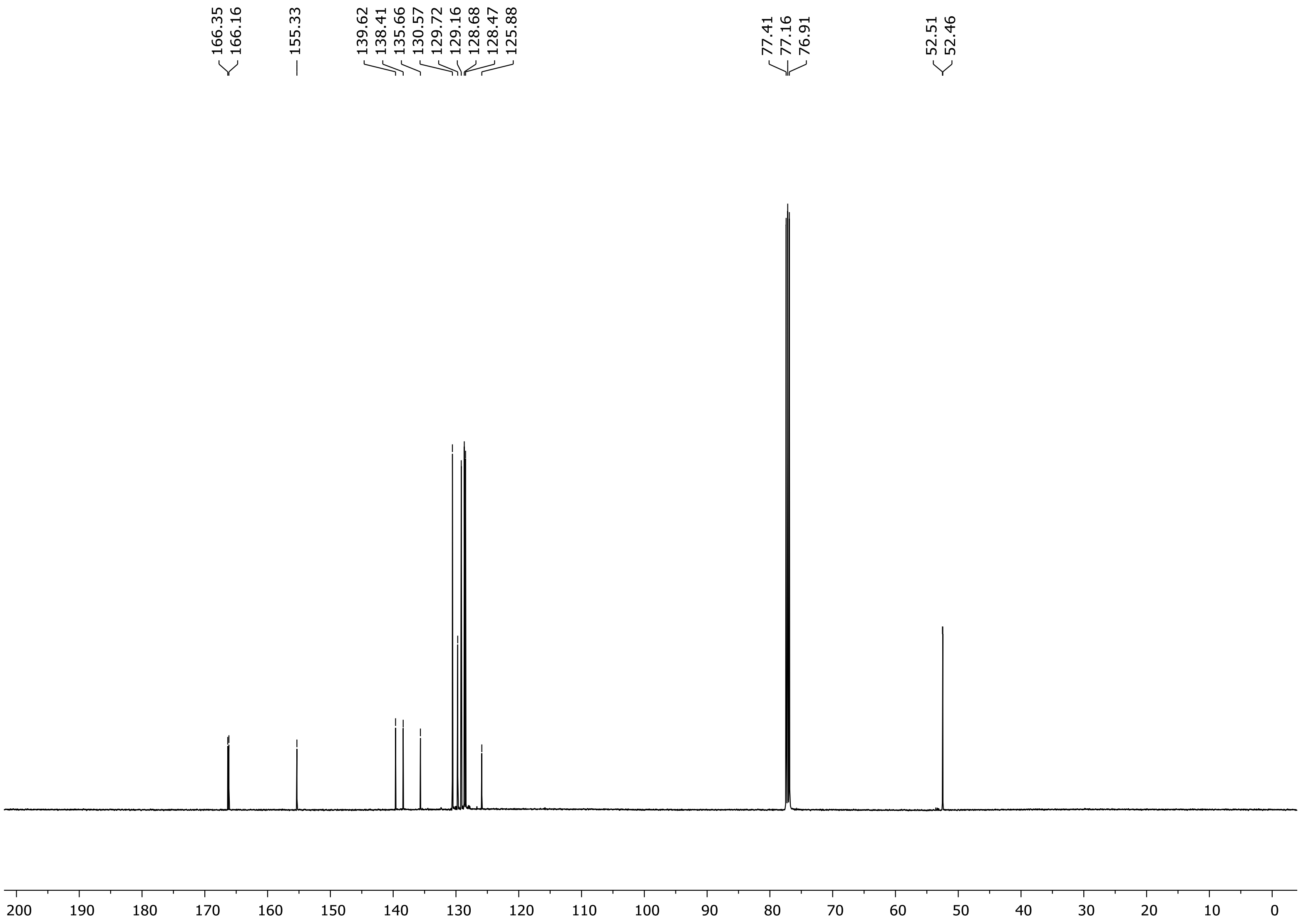

Figure S132: **^{1}H NMR** (500 MHz, $CDCl_3$, 298 K) spectrum of compound **6i**

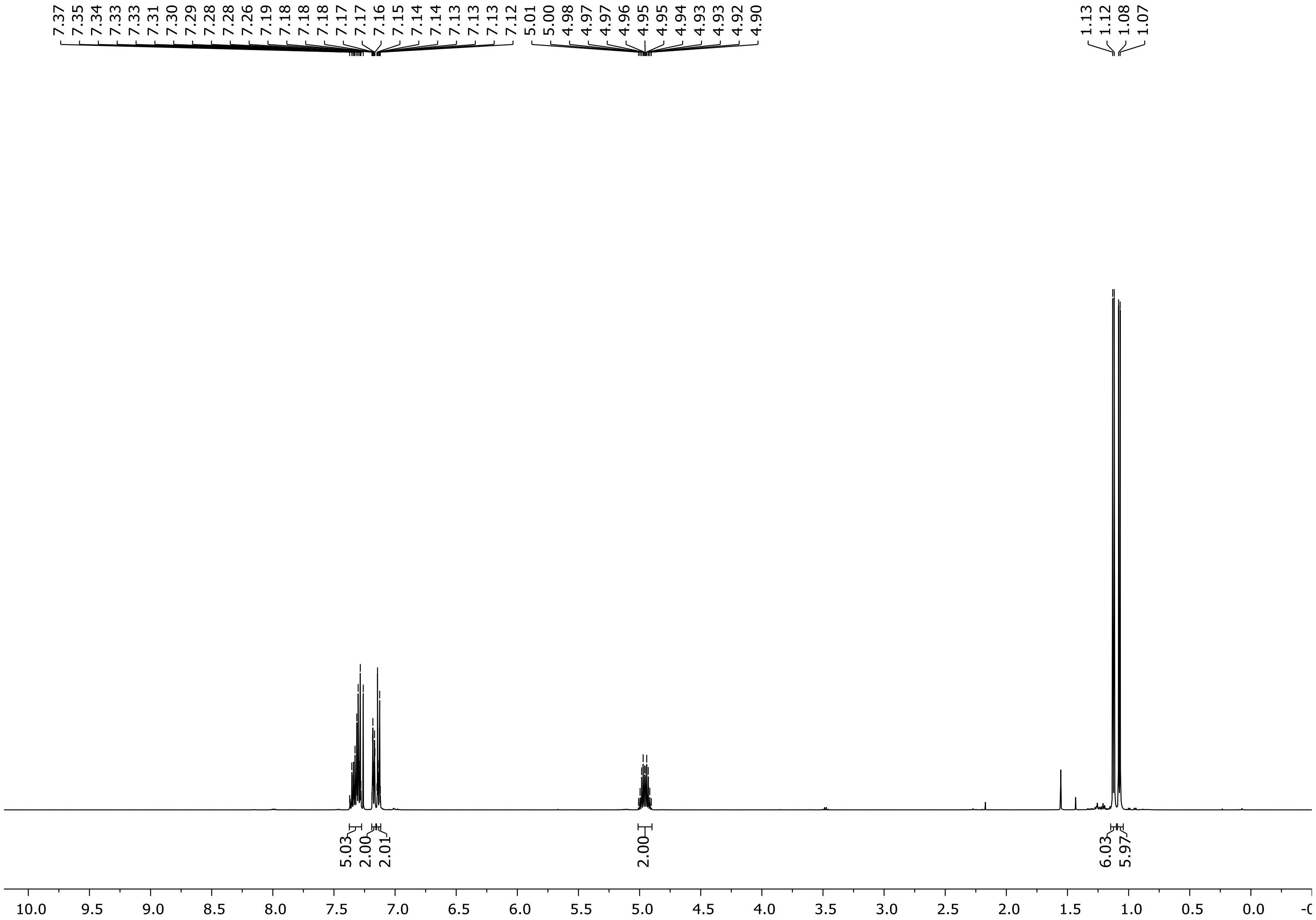

Figure S133: ^{13}**C NMR** (126 MHz, $CDCl_3$, 298 K) spectrum of compound **6i**

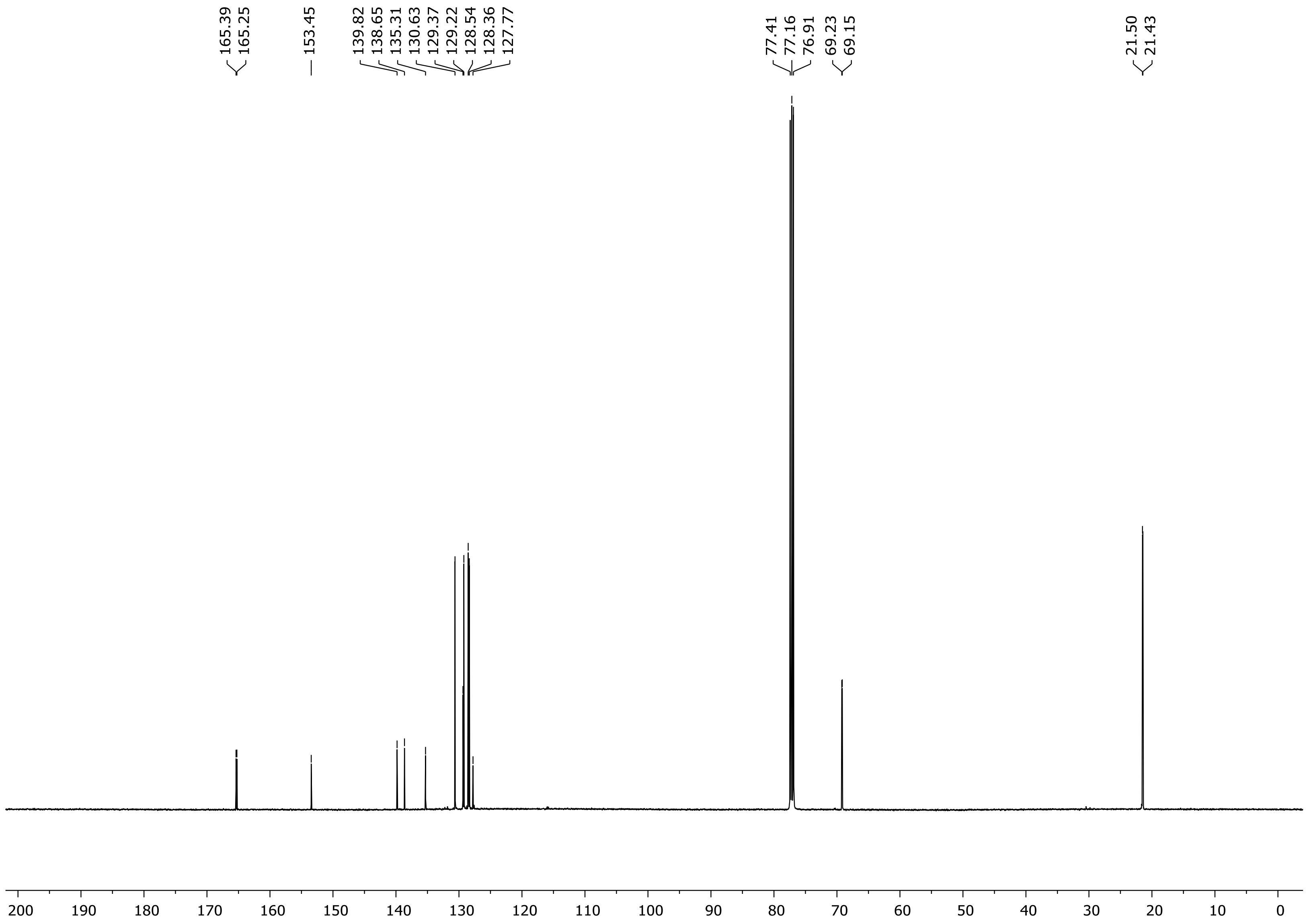

Figure S134: **¹H NMR** (500 MHz, $CDCl_3$, 298 K) spectrum of compound **6j**

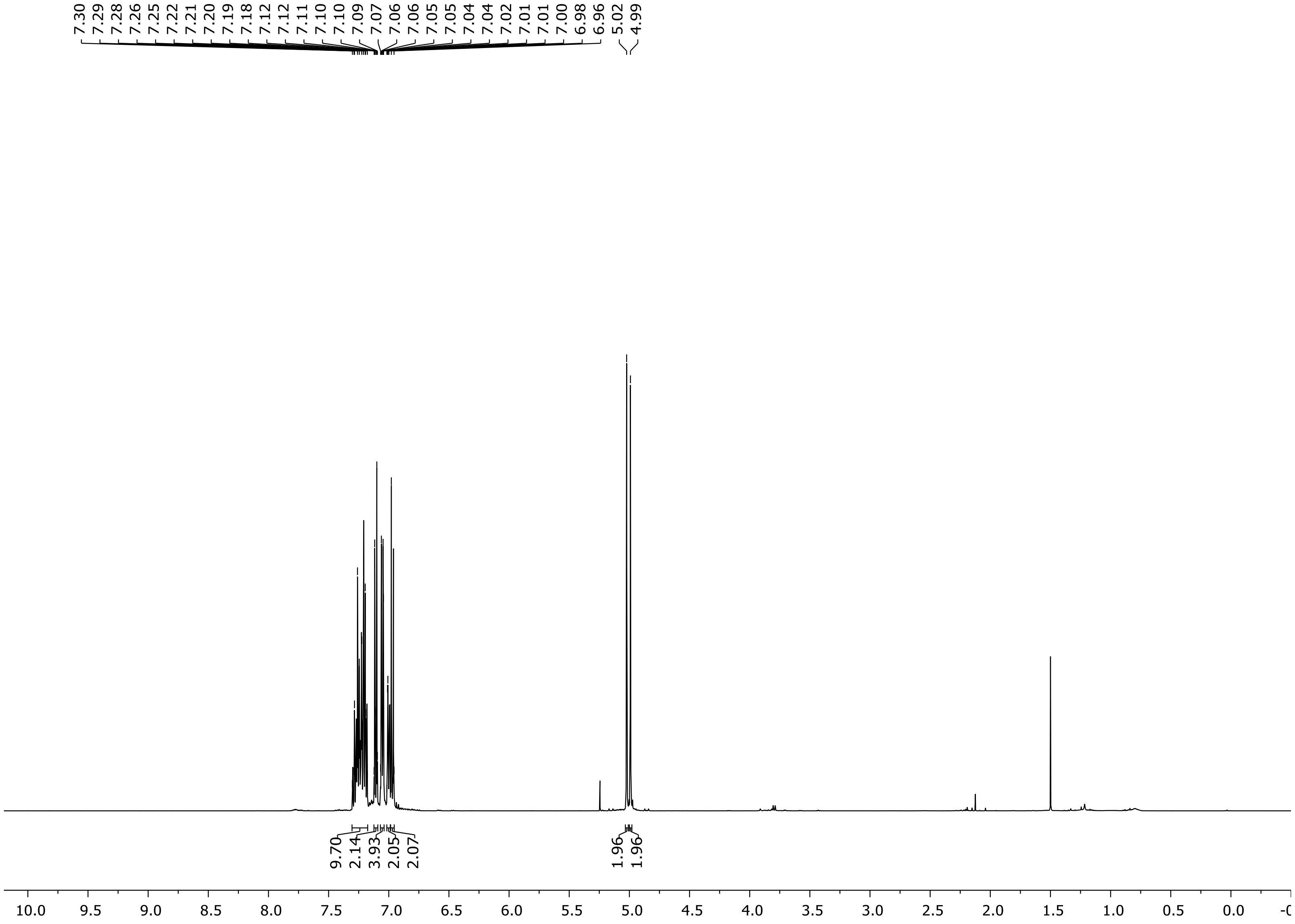

Figure S135: ^{13}**C NMR** (126 MHz, $CDCl_3$, 298 K) spectrum of compound **6j**

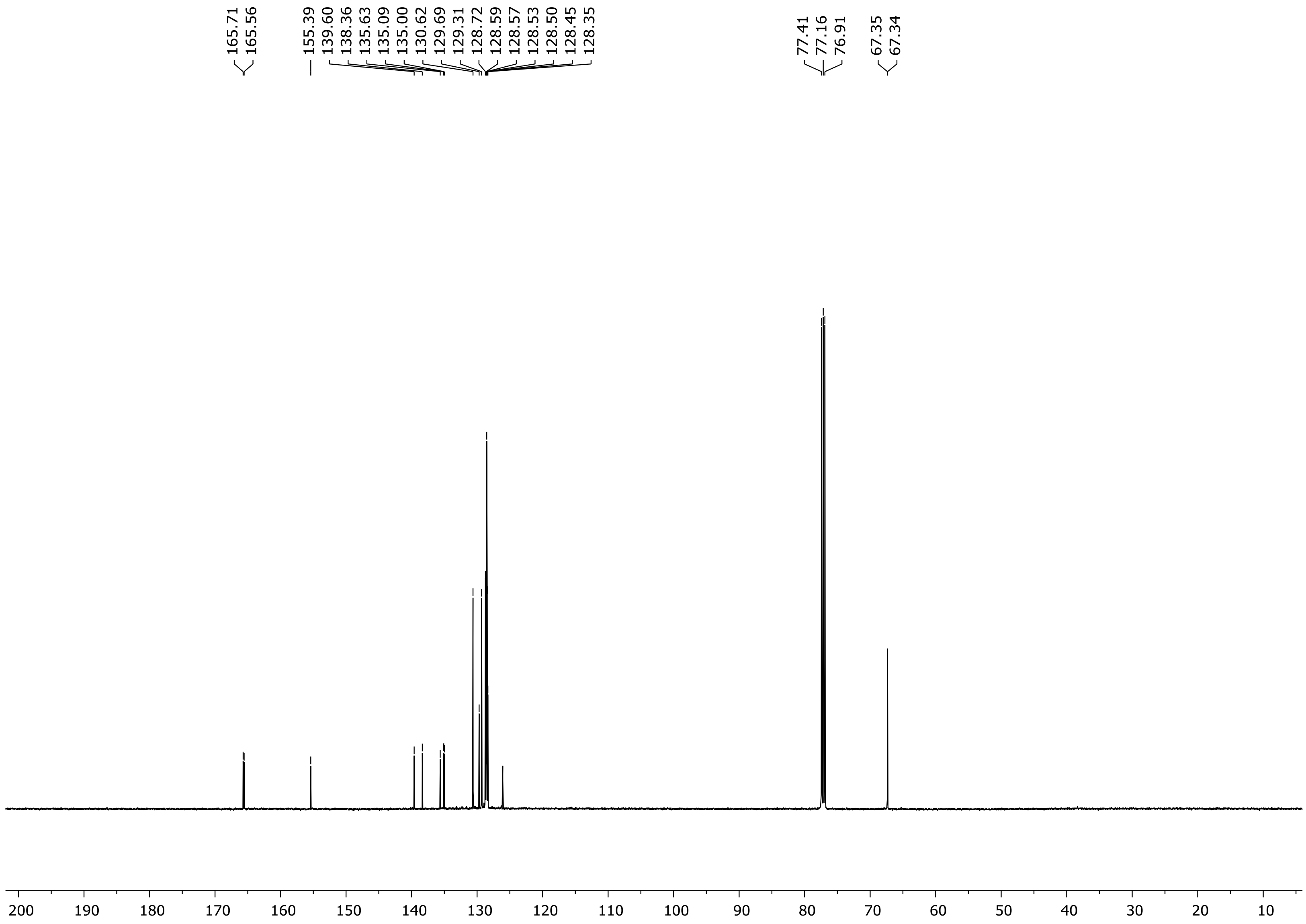

Figure S136: [1]**H NMR** (500 MHz, $CDCl_3$, 298 K) spectrum of compound **6k**

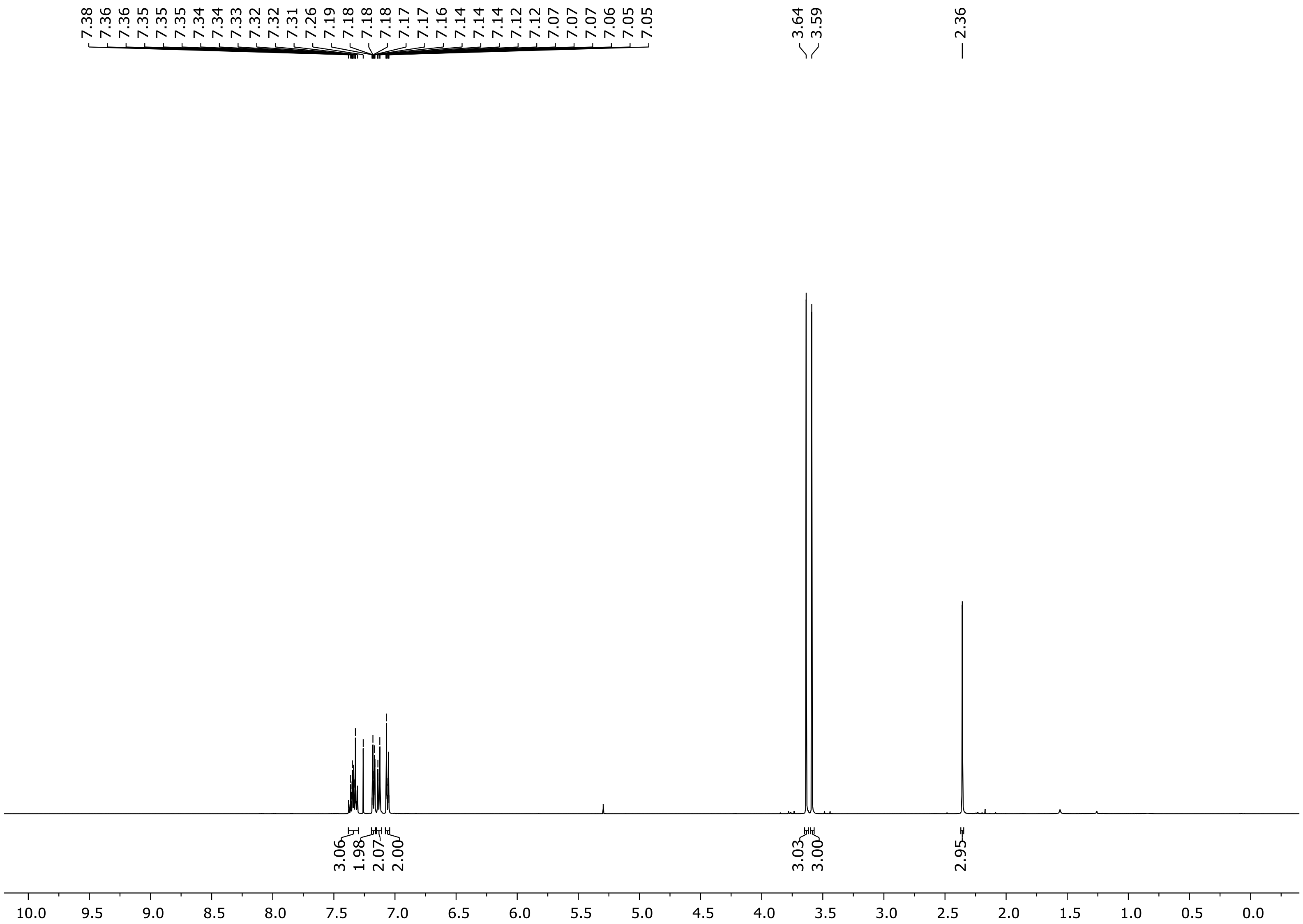

Figure S137: **^{13}C NMR** (126 MHz, $CDCl_3$, 298 K) spectrum of compound **6k**

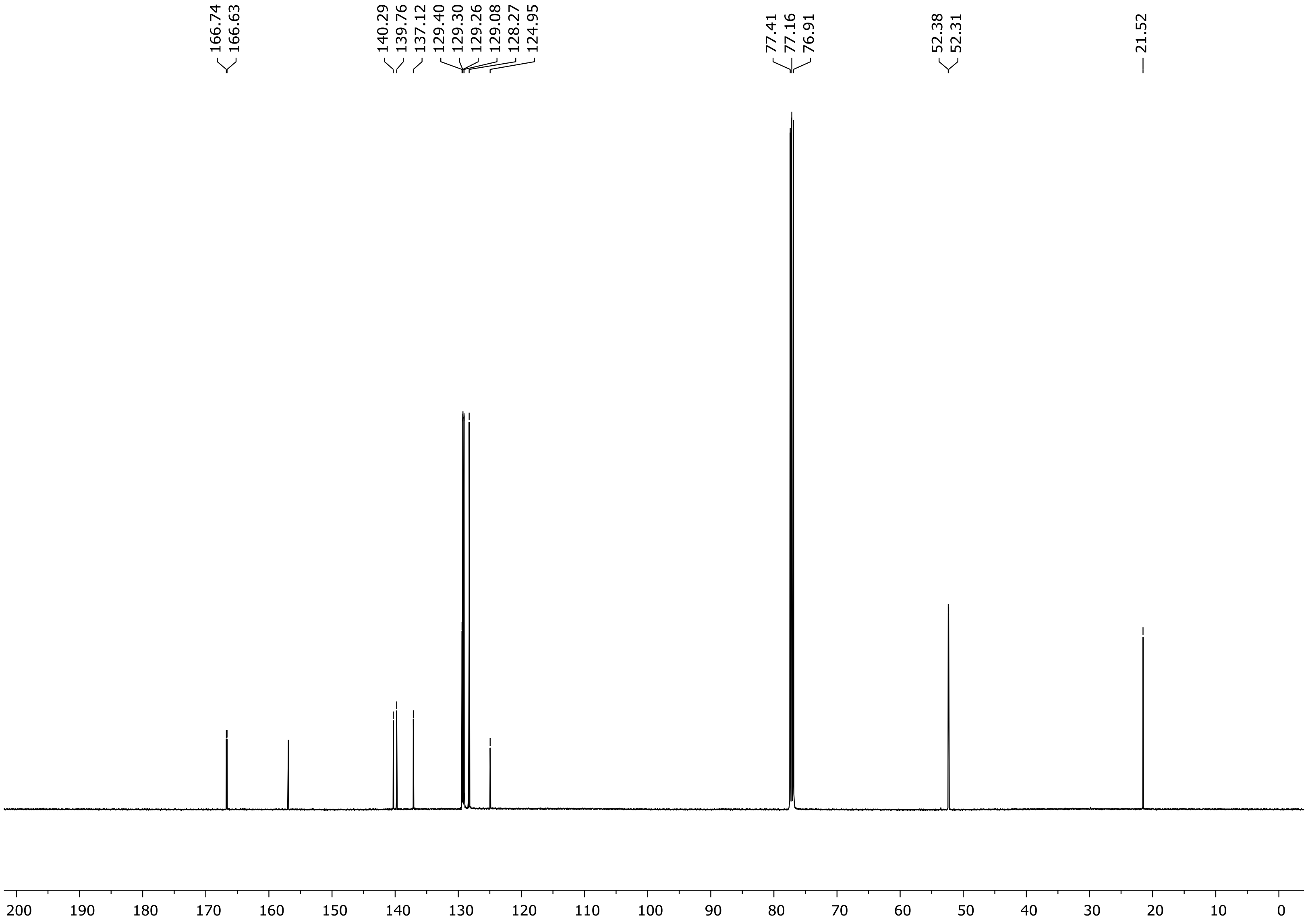

Figure S138: ^{1}**H NMR** (500 MHz, $CDCl_3$, 298 K) spectrum of compound **6l**

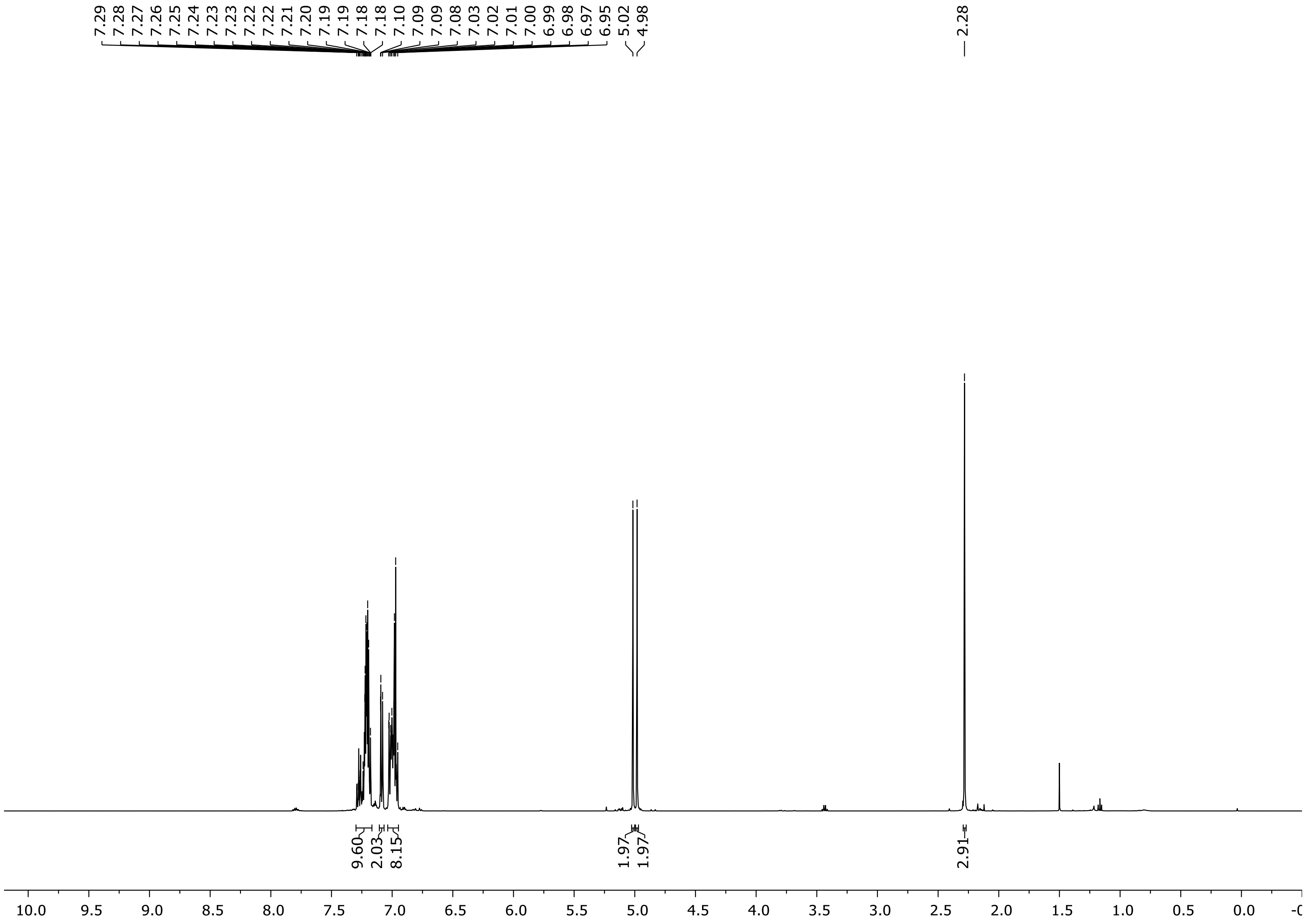

Figure S139: ^{13}**C NMR** (126 MHz, $CDCl_3$, 298 K) spectrum of compound **6l**

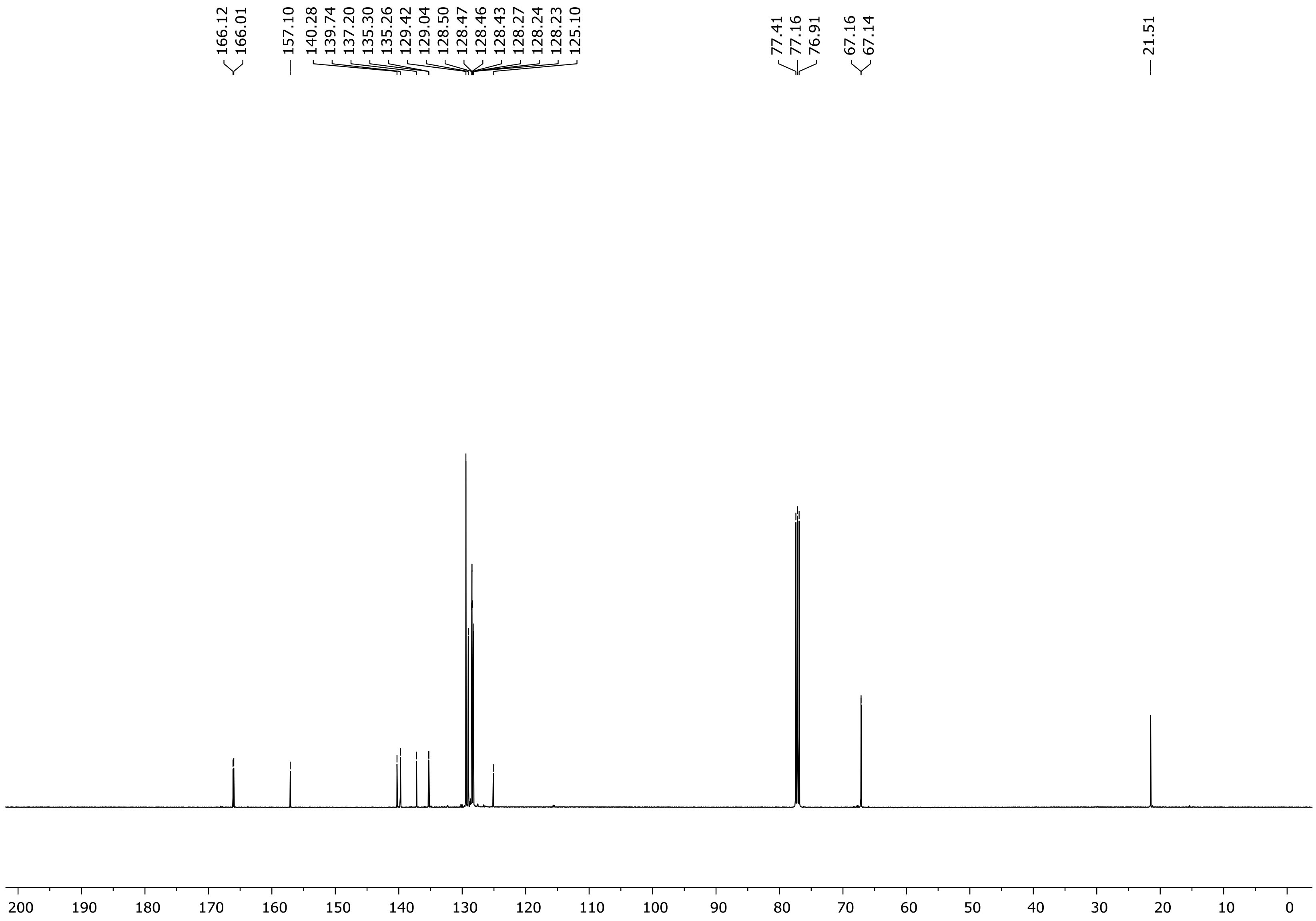

4. Crystallographic Data

4.1 Single crystal X-ray diffraction experimental

Single crystals of **3a**, **3n** and **6e** were grown in a fume hood by slow evaporation or vapor diffusion. Crystallographic studies were undertaken on single crystal mounted in paratone and studied on an Agilent SuperNova Dual Atlas three-circle diffractometer using Mo- or Cu-Kα radiation and a CCD detector. Measurements were taken at 190.00(10) K (**3a**, **3n**) or 200.01(10) K (**6e**) with temperatures maintained using an Oxford cryostream. Data were collected and integrated and data corrected for absorption using a numerical absorption correction based on Gaussian integration over a multifaceted crystal model within CrysAlisPro.[11] The structures were solved by direct methods and refined against F2 within SHELXL-2013.[12] The structures have been deposited with the Cambridge Structural Database (CCDC deposition numbers **1976457**, **1976537** and **1977547**). These can be obtained free of charge from the Cambridge Crystallographic Data Centre via www.ccdc.cam.ac.uk/data_request/cif.

4.2 Solid-state structures:

Figure S140: Solid-state structure of compound **3a**, thermal ellipsoids drawn at 50% probability level. H-atoms and TMS disorder have been omitted for clarity.

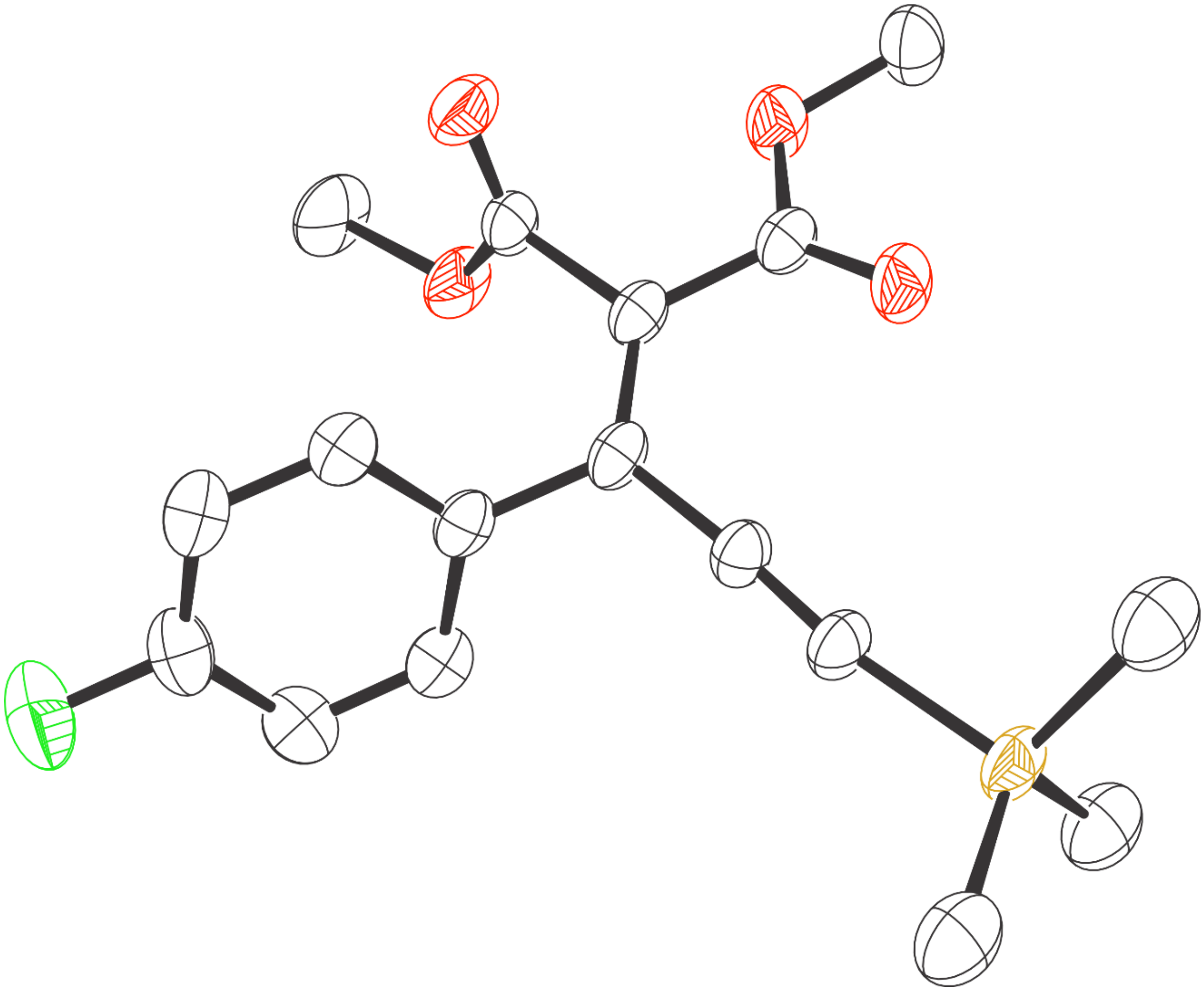

Figure S141: Solid-state structure of compound **3n**, thermal ellipsoids drawn at 50% probability level. H-atoms have been omitted for clarity.

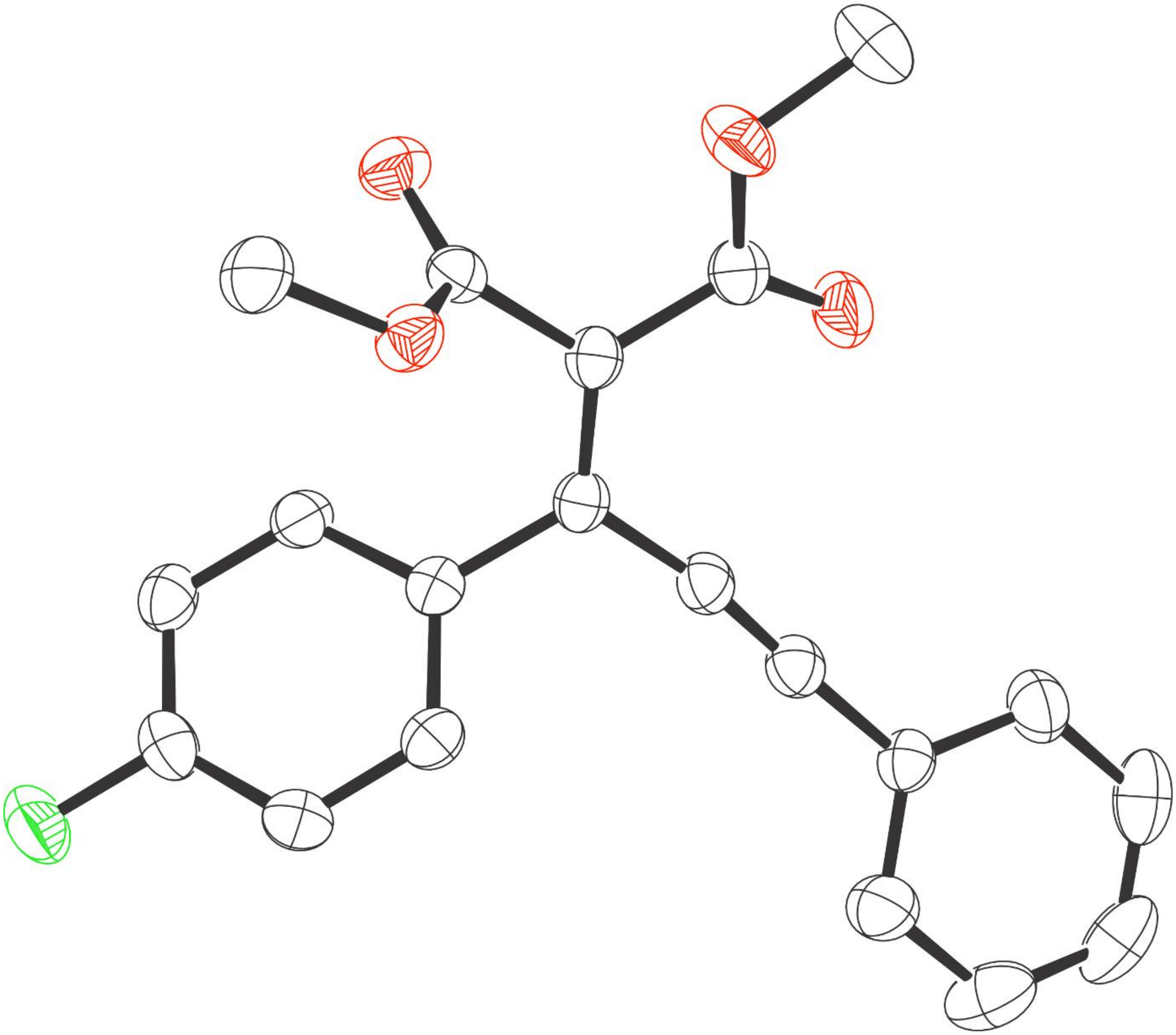

Figure S142: Solid-state structure of compound **6e**, thermal ellipsoids drawn at 50% probability level. Data collected at 200.01(10) K H-atoms have been omitted for clarity.

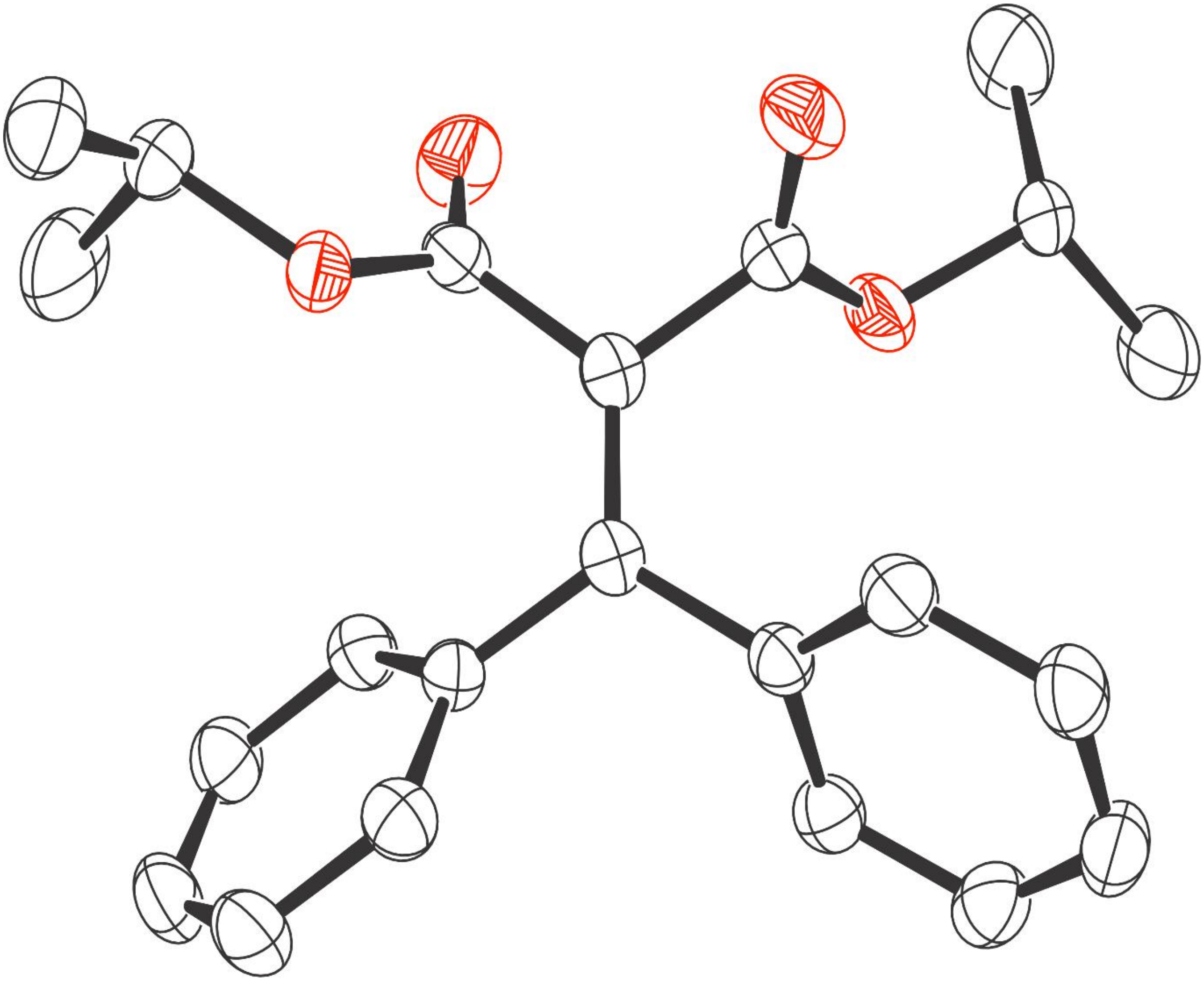

4.3 X-ray refinement data:

Table S1. Crystal data and structure refinement for compound **3a**.

Empirical formula	$C_{17}H_{19}FO_4Si$	
Formula weight	334.42	
Temperature	190(10) K	
Wavelength	0.71073 Å	
Crystal system	Triclinic	
Space group	*P*-1	
Unit cell dimensions	a = 5.9971(11) Å	α = 85.473(14)°.
	b = 10.4508(18) Å	β = 84.051(14)°.
	c = 14.670(2) Å	γ = 84.797(15)°.
Volume	908.4(3) $Å^3$	
Z	2	
Density (calculated)	1.223 Mg/m^3	
Absorption coefficient	0.154 mm^{-1}	
F(000)	352.0	
Crystal size	0.977 × 0.216 × 0.185 mm^3	
θ range for data collection	4.2060 to 28.3770°.	
Index ranges	$-6 \leq h \leq 8$, $-13 \leq k \leq 12$, $-14 \leq l \leq 19$	
Reflections collected	7611	
Independent reflections	4270 [R(int) = 0.0307]	
Completeness to θ = 29.659 °	83.0%	
Absorption correction	Gaussian	
Max. and min. transmission	1.000 and 0.763	
Refinement method	Full-matrix least-squares on F^2	
Data / restraints / parameters	4270 / 33 / 213	
Goodness-of-fit on F^2	0.875	
Final R indices [I>2σ(I)]	R1 = 0.0620, wR2 = 0.1615	
R indices (all data)	R1 = 0.0913, wR2 = 0.1975	
Extinction coefficient	n/a	
Largest diff. peak and hole	0.441 and -0.378 e.$Å^{-3}$	

Table S2. Crystal data and structure refinement for compound **3n**.

Empirical formula	$C_{20}H_{15}FO_4$	
Formula weight	338.32	
Temperature	190(10) K	
Wavelength	0.71073 Å	
Crystal system	Monoclinic	
Space group	$P2_1/c$	
Unit cell dimensions	a = 18.3342(11) Å	α = 90°.
	b = 5.7085(5) Å	β = 93.557(6)°.
	c = 16.2664(11) Å	γ = 90°.
Volume	1699.2(2) $Å^3$	
Z	4	
Density (calculated)	1.323 Mg/m^3	
Absorption coefficient	0.099 mm^{-1}	
F(000)	704	
Crystal size	0.535 × 0.186 × 0.053 mm^3	
θ range for data collection	4.2750 to 29.0220°.	
Index ranges	$-23 \le h \le 25, -7 \le k \le 6, -22 \le l \le 19$	
Reflections collected	9432	
Independent reflections	4101 [R(int) = 0.0251]	
Completeness to θ = 29.795 °	84.7%	
Absorption correction	Gaussian	
Max. and min. transmission	1.000 and 0.654	
Refinement method	Full-matrix least-squares on F^2	
Data / restraints / parameters	4095 / 0 / 227	
Goodness-of-fit on F^2	0.934	
Final R indices [I>2σ(I)]	R1 = 0.0490, wR2 = 0.1003	
R indices (all data)	R1 = 0.0743, wR2 = 0.1130	
Extinction coefficient	0.0043(6)	
Largest diff. peak and hole	0.248 and -0.184 e.$Å^{-3}$	

Table S3. Crystal data and structure refinement for compound **6e**.

Empirical formula	$C_{22}H_{24}O_4$	
Formula weight	352.41	
Temperature	200.01(10) K	
Wavelength	0.71073 Å	
Crystal system	Monoclinic	
Space group	$P2_1/c$	
Unit cell dimensions	a = 10.6266(5) Å	α = 90°.
	b = 10.1624(5) Å	β = 93.715(4)°.
	c = 18.5303(7) Å	γ = 90°.
Volume	1996.91(15) $Å^3$	
Z	4	
Density (calculated)	1.172 Mg/m^3	
Absorption coefficient	0.080 mm^{-1}	
F(000)	752.0	
Crystal size	0.249 × 0.226 × 0.209 mm^3	
θ range for data collection	3.8490 to 27.9840°.	
Index ranges	-13 ≤ h ≤ 11, -12 ≤ k ≤ 12, -23 ≤ l ≤ 22	
Reflections collected	10172	
Independent reflections	4337 [R(int) = 0.0250]	
Completeness to θ = 27.100 °	98.7%	
Absorption correction	Gaussian	
Max. and min. transmission	1.000 and 0.779	
Refinement method	Full-matrix least-squares on F^2	
Data / restraints / parameters	4337 / 114 / 270	
Goodness-of-fit on F^2	1.005	
Final R indices [I>2σ(I)]	R1 = 0.0434, wR2 = 0.0821	
R indices (all data)	R1 = 0.0638, wR2 = 0.0917	
Extinction coefficient	0.0169(9)	
Largest diff. peak and hole	0.220 and -0.190 e.$Å^{-3}$	

5. Computational Data

5.1 Additional energy profiles

Figure S143: The free energy profile for production of **I2** via coordination of $B(C_6F_5)_3$ to pendant oxygen of the carboxylate group vs production of **I2** via coordination of $B(C_6F_5)_3$ to the bonded oxygen of the carboxylate group.

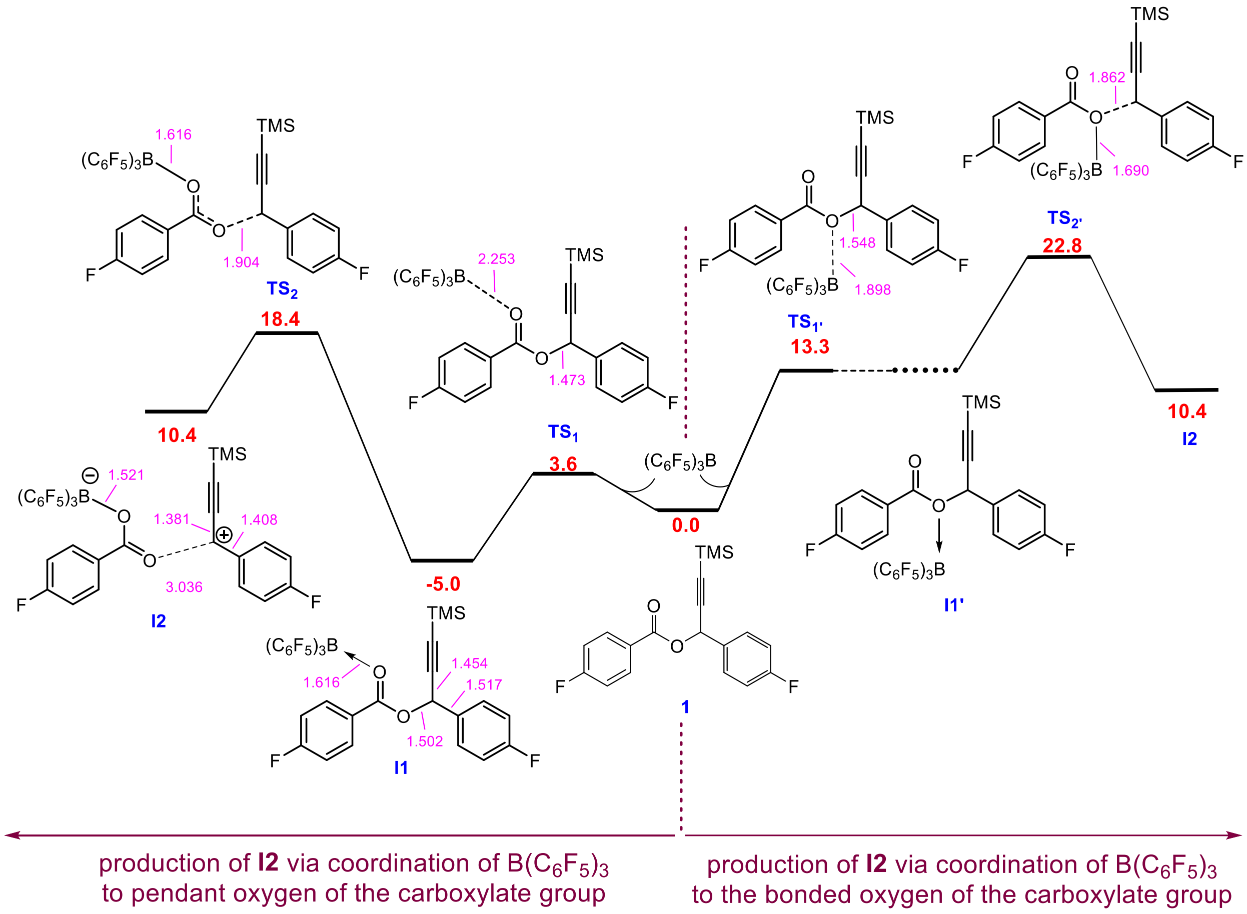

Figure S144: The free energy profile for coordination of the borane to the diazo compound calculated by SMD/M06-2X-D3/def2-TZVP//CPCM/B3LYP/6-31G(d). The relative free energies are given in kcal/mol.

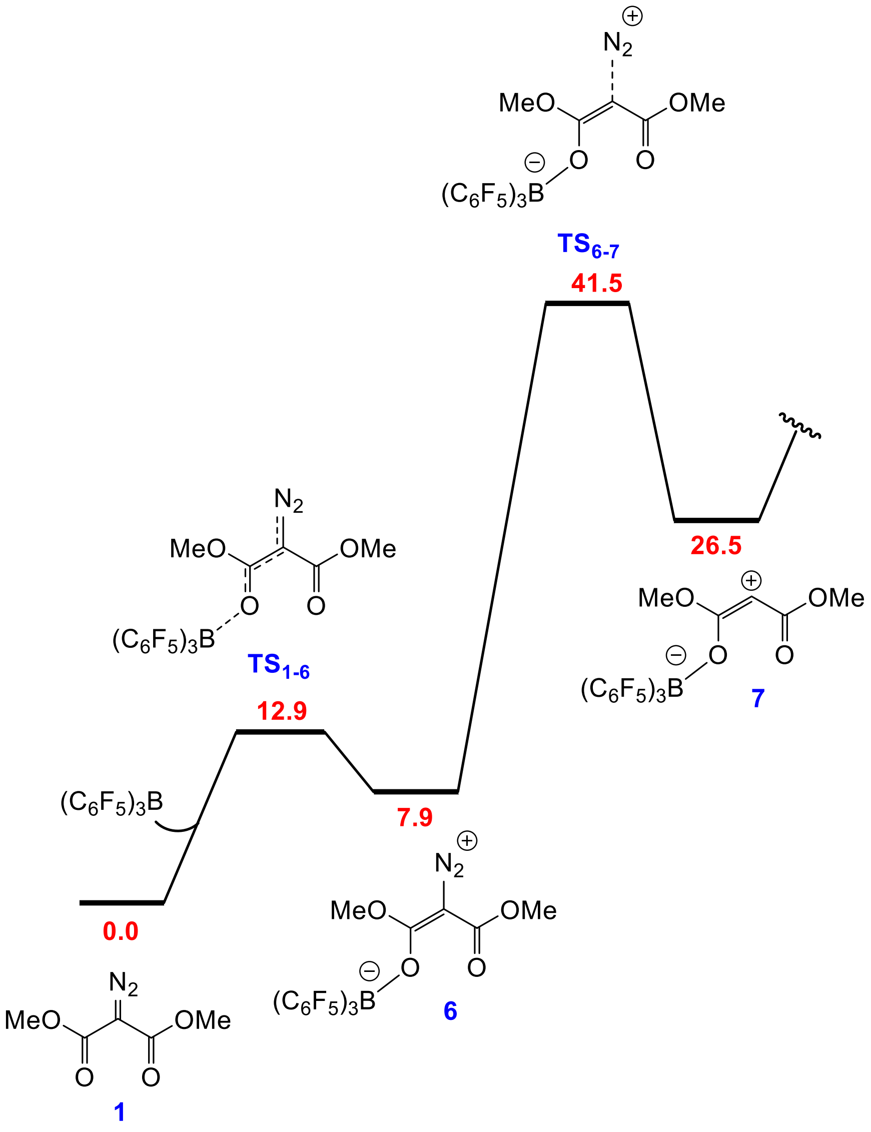

Figure S145: The free energy profile for the reaction calculated by SMD/M06-2X/def2-TZVP//CPCM/B3LYP/6-31G(d). The relative free energies are given in kcal/mol.

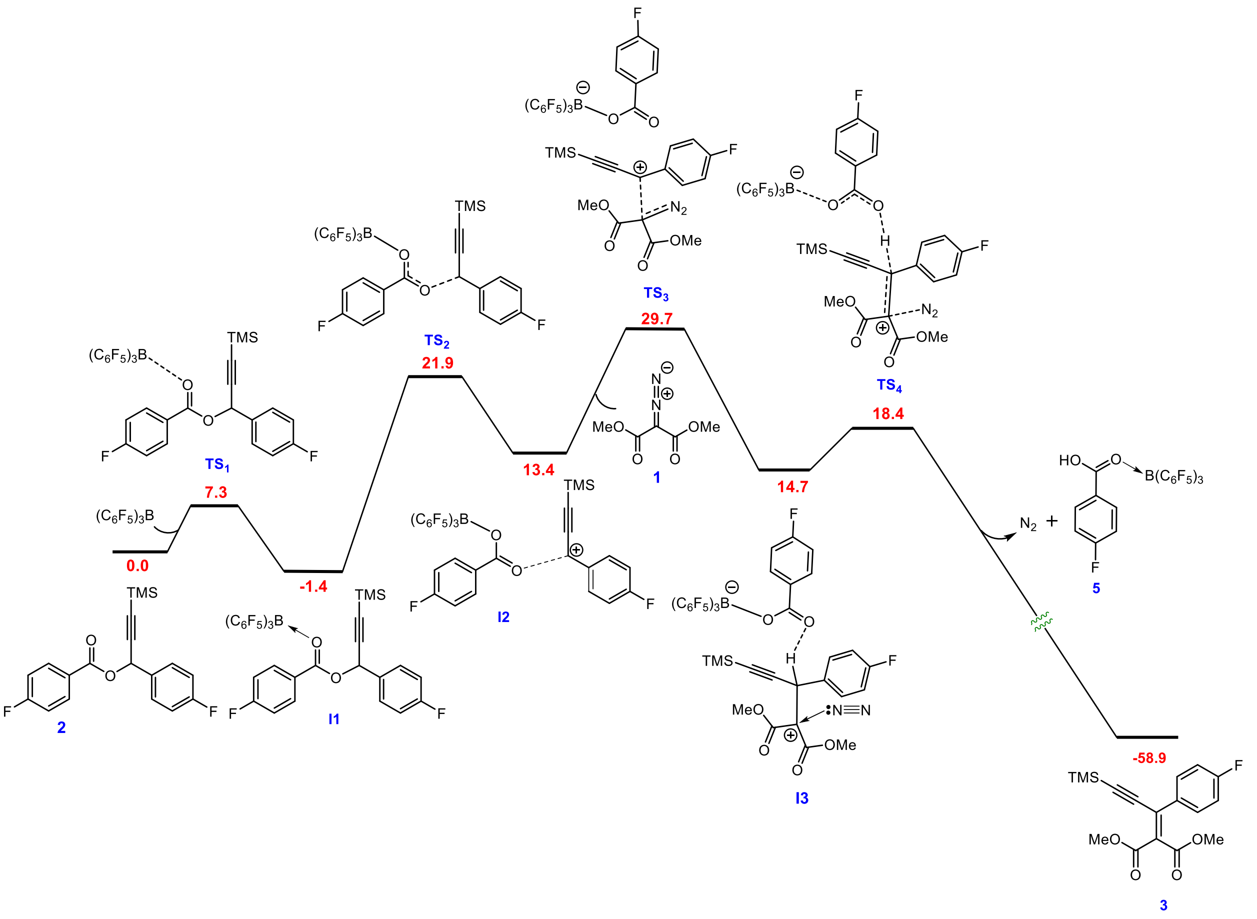

Figure S146: The free energy profile for the reaction calculated by SMD/wB97XD/def2-TZVP//CPCM/B3LYP/6-31G(d). The relative free energies are given in kcal/mol.

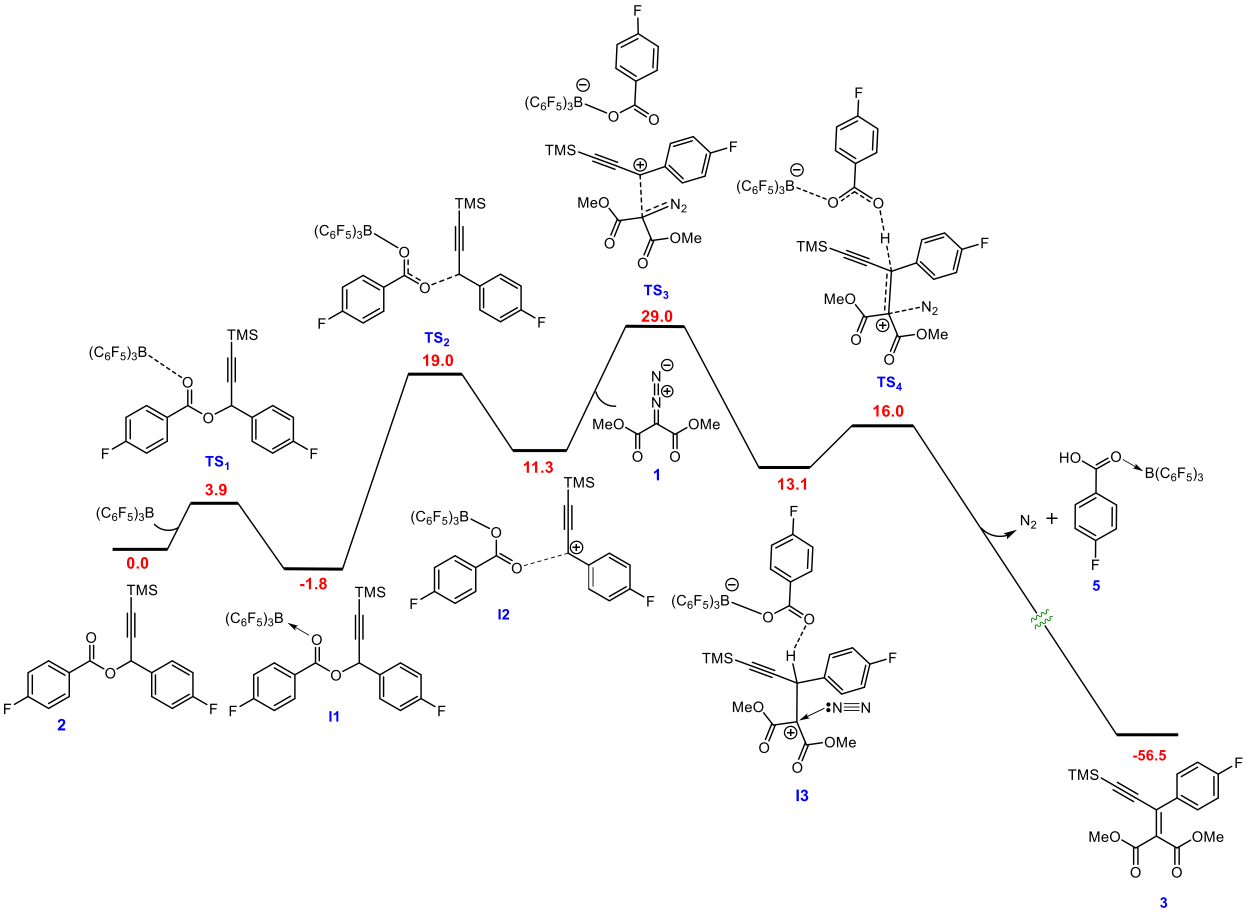

5.2 Cartesian coordinates and total energies for all the calculated structures in Toluene.

1

E(CPCM/B3LYP/6-31G(d)) = -604.494636 au
H(CPCM/B3LYP/6-31G(d)) = -604.361751 au
G(CPCM/B3LYP/6-31G(d)) = -604.413467 au
E(SMD/M06-2X-D3/def2-TZVP//CPCM/B3LYP/6-31G(d)) = -604.517252 au

C	1.30450500	-0.49667000	0.00004000
O	2.31461200	0.40359500	-0.00021000
O	1.45563300	-1.69866300	0.00011600
C	-0.00000500	0.19076600	0.00019200
N	-0.00000400	1.51178100	0.00026300
N	0.00002300	2.64319100	0.00031900
C	-1.30451600	-0.49665800	0.00012000
O	-1.45562700	-1.69865500	0.00031100
O	-2.31461400	0.40361100	-0.00015500
C	-3.64084600	-0.15626100	-0.00023400
H	-3.79688100	-0.76865400	0.89116100
H	-4.31496400	0.69992500	0.00012800
H	-3.79703900	-0.76799200	-0.89206300
C	3.64083500	-0.15625500	-0.00048000
H	3.79737200	-0.76775700	0.89144900
H	3.79656100	-0.76888300	-0.89177000
H	4.31495800	0.69992700	-0.00131000

2

E(CPCM/B3LYP/6-31G(d)) = -1374.461266 au
H(CPCM/B3LYP/6-31G(d)) = -1374.115932 au
G(CPCM/B3LYP/6-31G(d)) = -1374.197134 au
E(SMD/M06-2X-D3/def2-TZVP//CPCM/B3LYP/6-31G(d)) = -1374.480469 au

C	4.63645100	-1.31310900	1.33493600
C	3.30121400	-0.92344200	1.31052600
C	2.67601200	-0.59215500	0.09864500
C	3.40575100	-0.65527800	-1.09863400
C	4.74296800	-1.04402900	-1.08757400
C	5.33378900	-1.36597600	0.13094200
H	5.13913100	-1.57309900	2.26033500
H	2.72505200	-0.87016300	2.22785700
H	2.92549600	-0.40043800	-2.03604300
H	5.32722900	-1.10058100	-1.99977500
F	6.62750200	-1.74244500	0.14562900
C	1.24463400	-0.18312800	0.14552800
O	0.58511100	-0.12300500	1.16536400
O	0.76231000	0.11171100	-1.08581800
C	-0.63118300	0.55884900	-1.20865900
H	-0.72128800	0.63684900	-2.29706600
C	-1.57797600	-0.46015000	-0.75286700
C	-2.39358100	-1.31091400	-0.44539800

Si	-3.61105200	-2.61104600	0.05000700
C	-3.94522700	-2.44759300	1.90196600
H	-3.02547600	-2.57390800	2.48437600
H	-4.36566600	-1.46526000	2.14620000
H	-4.66077900	-3.21051200	2.23319400
C	-2.86147600	-4.30031100	-0.34252000
H	-3.55464600	-5.10249700	-0.06021500
H	-2.64679100	-4.40336700	-1.41232700
H	-1.92562100	-4.45751000	0.20576500
C	-5.20039100	-2.34297500	-0.93651900
H	-5.01928000	-2.41939300	-2.01476300
H	-5.95000300	-3.09768100	-0.66781300
H	-5.63269900	-1.35584400	-0.73652200
C	-0.83003900	1.94870300	-0.62087500
C	-0.33660700	3.04768300	-1.33697600
C	-1.48003600	2.16566100	0.59942800
C	-0.47955400	4.34527000	-0.84751600
H	0.16714000	2.89048300	-2.28735100
C	-1.63844100	3.45732100	1.09996500
H	-1.86025000	1.31904700	1.15967400
C	-1.13231300	4.52526800	0.36711700
H	-0.10429200	5.20518000	-1.39213900
H	-2.14145200	3.64249700	2.04323700
F	-1.28354700	5.77833800	0.84746100

3

E(CPCM/B3LYP/6-31G(d)) = -1349.448367 au
H(CPCM/B3LYP/6-31G(d)) = -1349.096995 au
G(CPCM/B3LYP/6-31G(d)) = -1349.185014 au
E(SMD/M06-2X-D3/def2-TZVP//CPCM/B3LYP/6-31G(d)) = -1349.460432 au

C	0.02575600	2.78978900	-0.09178000
O	-0.44379200	3.86999900	0.22685700
O	1.30241700	2.61534900	-0.46240000
C	-0.79301200	1.54876900	-0.09953800
C	-2.26647400	1.86986600	-0.05665100
O	-2.88289300	2.34779800	-0.98653700
O	-2.80195100	1.57843200	1.13773600
C	-4.21096700	1.85386900	1.27129100
H	-4.78299800	1.24661300	0.56515900
H	-4.46172500	1.58767700	2.29765700
H	-4.40997800	2.91182000	1.08596200
C	-0.33685600	0.26109800	-0.09561900
C	1.04325400	-0.09131000	-0.04156500
C	2.18530800	-0.52267100	0.02148100
Si	3.93408200	-1.12026800	0.09978700
C	4.10951200	-2.21795300	1.62766100
H	3.87987800	-1.66483900	2.54551200
H	3.43897500	-3.08336900	1.57753600
H	5.13681700	-2.59391000	1.71094000

C	5.07542300	0.37957600	0.22433100
H	6.12424200	0.06149900	0.27369400
H	4.96526700	1.03654100	-0.64613400
H	4.86113900	0.96965000	1.12267500
C	4.28939300	-2.10136800	-1.47469000
H	4.16341900	-1.48016200	-2.36868000
H	5.32117300	-2.47425500	-1.46739500
H	3.62060000	-2.96460000	-1.56669900
C	-1.26710200	-0.90993000	-0.13785400
C	-1.05618700	-2.00741800	0.71458600
C	-2.33408500	-0.95657200	-1.05127200
C	-1.90759600	-3.10823200	0.68728600
H	-0.22332800	-1.99214900	1.40999500
C	-3.18652600	-2.05897300	-1.09708300
H	-2.49155700	-0.13725400	-1.74398600
C	-2.96185600	-3.11440300	-0.22114500
H	-1.76308100	-3.95412400	1.35094000
H	-4.00631700	-2.11021400	-1.80566400
F	-3.78408400	-4.18288300	-0.25914200
C	2.12609100	3.79492500	-0.42463500
H	2.14496900	4.21643600	0.58333900
H	3.12040700	3.46171400	-0.72110900
H	1.74700100	4.54563100	-1.12255600

5

E(CPCM/B3LYP/6-31G(d)) = -2728.268218 au

H(CPCM/B3LYP/6-31G(d)) = -2727.966091 au

G(CPCM/B3LYP/6-31G(d)) = -2728.077861 au

E(SMD/M06-2X-D3/def2-TZVP//CPCM/B3LYP/6-31G(d)) = -2728.583837 au

B	-0.31193400	0.07159800	0.00389900
C	-0.77390000	-0.66435200	-1.38622400
C	-1.89097100	-0.30429700	-2.14421300
C	-0.10123000	-1.79925800	-1.84965200
C	-2.29380100	-0.98712200	-3.29099800
C	-0.46271800	-2.50573500	-2.99288100
C	-1.57311000	-2.09461300	-3.72232100
C	-0.75901000	1.63698800	0.15017300
C	-1.30748900	2.22978600	1.28968500
C	-0.52787000	2.52214000	-0.90878200
C	-1.63888800	3.58241100	1.36816100
C	-0.83903300	3.87719400	-0.87281400
C	-1.40452200	4.41334600	0.27974600
C	-0.71419200	-0.93835600	1.22790400
C	-2.07257500	-1.11096100	1.51329800
C	0.13554800	-1.77634800	1.94906000
C	-2.56331900	-2.00821500	2.45403100
C	-0.30966500	-2.69052500	2.90416000
C	-1.66941600	-2.80925600	3.15969600
F	0.96676500	-2.28560000	-1.16924000

F	0.23830700	-3.58065800	-3.38604600
F	-1.94505200	-2.76005900	-4.82264400
F	-3.37128700	-0.58374900	-3.98052600
F	-2.65323000	0.75075600	-1.79545600
F	0.03131900	2.06969900	-2.05192800
F	-0.59530800	4.66735100	-1.92962700
F	-1.70958200	5.71497100	0.34192600
F	-2.16572900	4.08956700	2.49355300
F	-1.53414200	1.51462700	2.40966000
F	-2.98400400	-0.37037200	0.84774800
F	-3.88015900	-2.11350600	2.68315100
F	-2.11541200	-3.68236900	4.07104300
F	0.56732200	-3.45765000	3.57056800
F	1.48013800	-1.74859100	1.76240500
O	1.27797700	0.13843900	-0.14573500
C	2.13852900	0.56471400	0.65601100
O	1.73700000	1.14292300	1.77027700
C	3.55968700	0.41895100	0.33482600
C	3.93416500	-0.55904900	-0.60664700
C	4.54042500	1.22841000	0.93747200
C	5.27238900	-0.73619900	-0.92991800
H	3.17072000	-1.18378000	-1.05578800
C	5.87992400	1.06345600	0.61023900
H	4.27193100	2.02114300	1.63067100
C	6.22195400	0.07907000	-0.31594700
H	5.58993800	-1.49008500	-1.64149100
H	6.65354400	1.68338500	1.04873900
F	7.51324700	-0.08590000	-0.62873300
H	2.47949000	1.32073700	2.37496000

I1

E(CPCM/B3LYP/6-31G(d)) = -3582.722318 au

H(CPCM/B3LYP/6-31G(d)) = -3582.191076 au

G(CPCM/B3LYP/6-31G(d)) = -3582.339782 au

E(SMD/M06-2X-D3/def2-TZVP//CPCM/B3LYP/6-31G(d)) = -3582.987890 au

C	3.22108900	-2.87038900	-1.95857200
C	2.12070600	-2.10973100	-1.58059000
C	1.68537000	-1.04053300	-2.38185800
C	2.37127500	-0.75071400	-3.57861600
C	3.48281900	-1.49266200	-3.95374900
C	3.88648100	-2.54602500	-3.13653200
H	3.56457300	-3.70296900	-1.35496500
H	1.60323200	-2.36569100	-0.66735600
H	2.03777600	0.06708800	-4.20582400
H	4.03306600	-1.27230500	-4.86169700
F	4.95377400	-3.27289100	-3.49960000
C	0.48244600	-0.25435600	-2.04203400
O	0.00535100	-0.02060600	-0.90524500
O	-0.11260300	0.20640100	-3.11980500

C	-1.39113500	0.99342200	-3.13280000
H	-1.47598700	1.13264100	-4.21529300
C	-2.49545100	0.15967400	-2.68468000
C	-3.42389800	-0.56150700	-2.37036200
Si	-4.80060000	-1.68024600	-1.82087700
C	-5.11809800	-1.39201600	0.01666200
H	-4.27987700	-1.75525100	0.61976200
H	-5.24878700	-0.32966000	0.24824100
H	-6.02242300	-1.92641000	0.33267500
C	-4.25139100	-3.45832900	-2.13296300
H	-5.04682500	-4.15672700	-1.84448400
H	-4.02332800	-3.62925700	-3.19114800
H	-3.35962300	-3.70385700	-1.54678500
C	-6.32804600	-1.24883800	-2.84482700
H	-6.14547000	-1.38830300	-3.91634300
H	-7.16995100	-1.89228500	-2.56125500
H	-6.63488800	-0.20861800	-2.68700300
C	-1.23558800	2.35567600	-2.48388100
C	-0.22187400	3.20710400	-2.94704700
C	-2.12232800	2.81342800	-1.50534900
C	-0.07800500	4.49017400	-2.42804900
H	0.46470700	2.86545300	-3.71679200
C	-2.00043400	4.10291900	-0.98670100
H	-2.91103800	2.16164600	-1.14626300
C	-0.97674900	4.91655600	-1.45464000
H	0.70524200	5.15842500	-2.76926800
H	-2.68182300	4.47519600	-0.22932600
F	-0.85147100	6.16271900	-0.95552300
B	0.51344800	-0.14419400	0.62427400
C	0.04280900	-1.59996700	1.21289400
C	0.25692300	-1.86052600	2.57111300
C	-0.70261000	-2.57694700	0.55140300
C	-0.20354400	-2.99412300	3.22954900
C	-1.19003600	-3.72686200	1.17227000
C	-0.94194400	-3.93899600	2.52169100
C	-0.38621700	0.97830600	1.41816500
C	0.09434100	1.91852800	2.33427700
C	-1.77745900	0.96865000	1.28661500
C	-0.72729600	2.80756300	3.02722400
C	-2.63323000	1.84297100	1.94695200
C	-2.10242700	2.77726300	2.82858700
C	2.11333900	0.18714600	0.60007000
C	3.14118200	-0.62981200	1.07408000
C	2.54775400	1.40761300	0.07099300
C	4.48775900	-0.26764000	1.05125900
C	3.87695500	1.80890900	0.02101500
C	4.86001700	0.96230700	0.52457300
F	-0.99383600	-2.46624600	-0.76733200

F	-1.90278300	-4.62523800	0.47158700
F	-1.40007900	-5.03758600	3.13267100
F	0.04656600	-3.18125900	4.53320300
F	0.94969200	-0.97001300	3.31185100
F	-2.36840400	0.07268000	0.46792600
F	-3.95873500	1.79921600	1.72347400
F	-2.90246500	3.63283300	3.47607400
F	-0.19824400	3.69120800	3.88684600
F	1.40922000	2.01479700	2.61497000
F	2.88630700	-1.86764500	1.55054300
F	5.42490300	-1.10650700	1.51705200
F	6.14742000	1.32342300	0.48803700
F	4.21813000	2.99668100	-0.50063400
F	1.64106900	2.27377400	-0.43474900

I2

E(CPCM/B3LYP/6-31G(d)) = -3582.707006 au

H(CPCM/B3LYP/6-31G(d)) = -3582.177614 au

G(CPCM/B3LYP/6-31G(d)) = -3582.333295 au

E(SMD/M06-2X-D3/def2-TZVP//CPCM/B3LYP/6-31G(d)) = -3582.954546au

C	-4.44509300	0.47061300	-2.74390700
C	-3.14592100	0.44834300	-2.24016200
C	-2.26600200	-0.59152200	-2.57119100
C	-2.71243100	-1.60977700	-3.43126200
C	-4.00953900	-1.60886400	-3.93266600
C	-4.85514100	-0.56145200	-3.57930300
H	-5.13377000	1.27165500	-2.49753100
H	-2.82806200	1.25084300	-1.59042900
H	-2.02402700	-2.40216500	-3.70195700
H	-4.36912000	-2.39471300	-4.58854500
F	-6.11300600	-0.54803700	-4.06546000
C	-0.83841700	-0.64915000	-2.11534800
O	-0.40675600	0.03517300	-1.08411200
O	-0.03769600	-1.33368400	-2.76961200
C	2.76872100	-1.51220600	-1.62588700
C	3.77958300	-0.57231000	-1.66979300
C	4.61263600	0.33368700	-1.69903900
Si	5.78660300	1.79484100	-1.72682300
C	6.97138000	1.59154800	-0.27820000
H	6.42719900	1.54701400	0.67048900
H	7.57025700	0.67931800	-0.37562000
H	7.66018300	2.44381400	-0.23456300
C	4.70349900	3.32601100	-1.57543500
H	5.32402500	4.22075900	-1.44601400
H	4.08812000	3.46418100	-2.47091300
H	4.03508400	3.24089000	-0.71326800
C	6.67794700	1.69815600	-3.38711000
H	5.97490300	1.75210300	-4.22486700
H	7.37151900	2.54353900	-3.47384200

H	7.25924000	0.77489600	-3.48238300
C	2.86161900	-2.79100500	-1.04467800
C	1.69448000	-3.61303000	-1.05468700
C	4.06007600	-3.27550400	-0.43926100
C	1.72036400	-4.86703900	-0.47834400
H	0.79110500	-3.22505500	-1.51285200
C	4.09035300	-4.52771400	0.13038800
H	4.94351300	-2.64545200	-0.43285100
C	2.91700100	-5.30096400	0.10102800
H	0.84895000	-5.51134000	-0.45814800
H	4.98076800	-4.93052800	0.59950800
F	2.95220300	-6.50769600	0.65559800
B	-0.92377500	0.44579900	0.28607500
C	-1.28983600	2.05528000	0.33268100
C	-1.68367800	2.63488000	1.54268100
C	-1.08861900	2.96958600	-0.70317800
C	-1.89242400	3.99602700	1.72705700
C	-1.28500600	4.34502100	-0.56465300
C	-1.68635500	4.86418600	0.65861200
C	0.40843600	0.31983700	1.28260700
C	0.43436900	-0.24279700	2.56076600
C	1.62189000	0.89808200	0.89157800
C	1.56810600	-0.27634500	3.37413100
C	2.77322500	0.89143800	1.67390600
C	2.75346300	0.29187800	2.92697100
C	-2.13824100	-0.58836400	0.70331300
C	-3.47185200	-0.26050600	0.94926300
C	-1.86875400	-1.95759700	0.78112800
C	-4.45547200	-1.19844900	1.26531600
C	-2.81210900	-2.93043700	1.08676000
C	-4.12482800	-2.54500200	1.33829800
F	-0.70719200	2.56351100	-1.93469400
F	-1.08347900	5.17008500	-1.60644400
F	-1.87811100	6.18259800	0.80897200
F	-2.27852600	4.48137000	2.91859100
F	-1.88078600	1.84463400	2.62239400
F	1.73049000	1.53448500	-0.29720300
F	3.90966900	1.47258900	1.23115800
F	3.85528200	0.27198500	3.69091100
F	1.52146000	-0.85128600	4.58710500
F	-0.66832500	-0.80124100	3.10151000
F	-3.90741000	1.01484400	0.85352200
F	-5.72185300	-0.80994800	1.48436100
F	-5.05633200	-3.46108600	1.63544400
F	-2.46944200	-4.22941900	1.14502800
F	-0.60915900	-2.40925900	0.54971700
H	1.79824900	-1.23885900	-2.07015500

I3

E(CPCM/B3LYP/6-31G(d)) = -4187.199604 au
H(CPCM/B3LYP/6-31G(d)) = -4186.535770 au
G(CPCM/B3LYP/6-31G(d)) = -4186.720819 au
E(SMD/M06-2X-D3/def2-TZVP//CPCM/B3LYP/6-31G(d)) = -4187.494414 au

C	-3.58740000	-1.37138500	-3.72579300
C	-2.65222200	-0.90486300	-2.80295100
C	-1.58057500	-1.71159500	-2.39748000
C	-1.45601500	-2.99933000	-2.94614500
C	-2.38960900	-3.48603800	-3.85537800
C	-3.44141900	-2.65675400	-4.23244200
H	-4.41864400	-0.75433000	-4.04993700
H	-2.76929800	0.09388200	-2.40675100
H	-0.61284200	-3.61257700	-2.64901400
H	-2.31161400	-4.48339900	-4.27535300
F	-4.34761400	-3.11649400	-5.12125000
C	-0.49282500	-1.25162700	-1.46767200
O	-0.67812900	-0.30285400	-0.58502800
O	0.62154400	-1.77895100	-1.58050400
C	3.16488600	-0.68864600	-0.32310700
C	3.36028300	0.71780200	-0.64049600
C	3.49008400	1.90343700	-0.88832700
Si	3.47820000	3.74014900	-1.17645300
C	4.11852000	4.55515000	0.40050600
H	3.50107900	4.26944900	1.25929400
H	5.15444200	4.26795600	0.61478300
H	4.08581300	5.64776800	0.30961900
C	1.70065700	4.25671300	-1.52401400
H	1.65725200	5.32365300	-1.77514800
H	1.25513200	3.69311500	-2.35026100
H	1.07748600	4.08587600	-0.64082500
C	4.60986800	4.10840500	-2.64353500
H	4.25674800	3.61040200	-3.55383200
H	4.63327300	5.18680100	-2.84234400
H	5.63896500	3.78233400	-2.45332000
C	2.92316600	-0.98513800	1.14754500
C	2.00174900	-1.98741500	1.49136100
C	3.57464500	-0.27061200	2.16418700
C	1.75762400	-2.29569200	2.82784400
H	1.44490900	-2.49388600	0.71030700
C	3.34546200	-0.57605700	3.50380900
H	4.23295200	0.55669000	1.91574500
C	2.44459700	-1.59132800	3.81067400
H	1.02668900	-3.04601600	3.10848100
H	3.83291400	-0.02844400	4.30314800
F	2.21636800	-1.88569300	5.10456200
B	-1.82558200	0.27873800	0.22135500
C	-2.35001900	1.70053800	-0.44086100
C	-3.32969500	2.45005500	0.21810900

C	-1.77200500	2.35069600	-1.53338300
C	-3.73424600	3.72234500	-0.16802000
C	-2.13906600	3.63052000	-1.95205200
C	-3.12570000	4.32458700	-1.26567900
C	-1.12378300	0.75409500	1.65743100
C	-1.63523000	0.55187800	2.94114300
C	0.06251900	1.49383800	1.62936100
C	-1.00822200	0.99862200	4.10505000
C	0.72593300	1.95256300	2.76279600
C	0.18828700	1.69852600	4.01856100
C	-2.95582000	-0.90116000	0.43644700
C	-4.28651800	-0.87756000	0.01987400
C	-2.57730200	-2.09205200	1.06454400
C	-5.18118600	-1.92874800	0.22183500
C	-3.42945800	-3.16721400	1.28591000
C	-4.75204000	-3.08288700	0.86306800
F	-0.80696000	1.76264600	-2.27759500
F	-1.53096500	4.19917200	-3.00910700
F	-3.49050600	5.55522200	-1.65479200
F	-4.68906900	4.38011800	0.51090100
F	-3.93495800	1.93443400	1.31132100
F	0.62880000	1.82698000	0.44746000
F	1.88654000	2.63242200	2.65580600
F	0.81363500	2.12505800	5.12662200
F	-1.55107600	0.75455200	5.31023300
F	-2.79885900	-0.10449100	3.13621600
F	-4.78794500	0.17900900	-0.65837500
F	-6.44934100	-1.83860100	-0.21250500
F	-5.59540700	-4.10565500	1.06302500
F	-2.99278100	-4.27851400	1.90389000
F	-1.30373300	-2.24702400	1.50440700
C	4.53511200	-1.26783900	-2.46031200
O	5.70541800	-0.70997600	-2.74117700
O	3.64348300	-1.54807500	-3.20934300
C	4.40249900	-1.53926800	-0.92687500
N	5.59759800	-1.07310200	-0.27212800
N	6.43279000	-0.62530200	0.30388000
C	4.23423300	-3.06251300	-0.63201800
O	3.36961000	-3.70043000	-1.16300100
O	5.12855400	-3.46759700	0.25950000
C	5.02527100	-4.85862500	0.68090900
H	5.12496200	-5.51185300	-0.18684600
H	5.84735700	-5.00066200	1.37909600
H	4.06217000	-5.01542000	1.16846000
C	5.92128000	-0.35465200	-4.13740200
H	5.86127700	-1.25144500	-4.75546300
H	5.16793400	0.37145000	-4.44575300
H	6.91941900	0.07724200	-4.16491000

H	2.31510400	-1.09537000	-0.91094700

N_2

E(CPCM/B3LYP/6-31G(d)) = -109.520971 au
H(CPCM/B3LYP/6-31G(d)) = -109.512065 au
G(CPCM/B3LYP/6-31G(d)) = -109.533819 au
E(SMD/M06-2X-D3/def2-TZVP//CPCM/B3LYP/6-31G(d)) = -109.529218 au

N	0.00000000	0.00000000	0.55258000
N	0.00000000	0.00000000	-0.55258000

TS_1

E(CPCM/B3LYP/6-31G(d)) = -3582.714535 au
H(CPCM/B3LYP/6-31G(d)) = -3582.184350 au
G(CPCM/B3LYP/6-31G(d)) = -3582.334293 au
E(SMD/M06-2X-D3/def2-TZVP//CPCM/B3LYP/6-31G(d)) = -3582.971851 au

C	3.64883600	-1.72097100	-2.51636600
C	2.40348800	-1.27974300	-2.07951900
C	1.81487000	-0.13608600	-2.64034400
C	2.49065700	0.56112800	-3.65685400
C	3.74348400	0.13977100	-4.08910700
C	4.30011000	-0.99744900	-3.51025900
H	4.11326700	-2.60963400	-2.10312600
H	1.87075400	-1.84325700	-1.32544100
H	2.03648500	1.43871000	-4.10173000
H	4.28712600	0.67168400	-4.86227100
F	5.50779600	-1.41385300	-3.93107700
C	0.47386800	0.30506400	-2.17488000
O	-0.02228100	0.07451800	-1.07659300
O	-0.16382700	0.98916500	-3.13890300
C	-1.54524200	1.46935900	-2.96256400
H	-1.73064800	1.86788400	-3.96676500
C	-2.47089500	0.36021200	-2.74667500
C	-3.26060700	-0.56085900	-2.64559000
Si	-4.41516200	-1.99687700	-2.43934500
C	-5.30591300	-1.80575400	-0.78656100
H	-4.59757900	-1.65755800	0.03383400
H	-5.98121500	-0.94227400	-0.80185700
H	-5.90728600	-2.69676000	-0.56826000
C	-3.39131400	-3.58181400	-2.49967100
H	-4.03526400	-4.45320500	-2.32706000
H	-2.92145500	-3.70537200	-3.48245000
H	-2.59702400	-3.59282000	-1.74687800
C	-5.65600900	-1.96607900	-3.86389400
H	-5.15469500	-2.05454500	-4.83442300
H	-6.36090600	-2.80190300	-3.77377100
H	-6.23815100	-1.03749500	-3.86776500
C	-1.63221000	2.62757700	-1.97663000
C	-0.70492700	3.67367800	-2.08191000
C	-2.66518100	2.72337800	-1.04028700

C	-0.79245800	4.79093300	-1.25551300
H	0.09723900	3.61455700	-2.81159000
C	-2.77389700	3.84264800	-0.21266500
H	-3.38975500	1.92045800	-0.95246900
C	-1.83218700	4.85600200	-0.33361200
H	-0.07677900	5.60360900	-1.32001200
H	-3.56895800	3.92994300	0.52050700
F	-1.92841000	5.93925400	0.46612400
B	0.59883700	-0.30961100	1.05440700
C	0.40030400	-1.88906100	1.27067400
C	0.65775500	-2.33578600	2.57060200
C	-0.10123500	-2.85836900	0.40473000
C	0.45478300	-3.64105200	2.99870300
C	-0.32562600	-4.17869100	0.79432900
C	-0.04887400	-4.57494100	2.09668600
C	-0.59640200	0.54065300	1.66347400
C	-0.41881600	1.56984800	2.59624400
C	-1.93047100	0.21869700	1.38300800
C	-1.47673800	2.25518900	3.18704700
C	-3.01138900	0.89240100	1.93855900
C	-2.78297200	1.92015700	2.84722900
C	2.04580600	0.30969200	0.88790300
C	3.23165300	-0.41455100	1.07810700
C	2.23436600	1.65256600	0.52159300
C	4.50104800	0.14401800	0.94879200
C	3.48283900	2.24100800	0.36909800
C	4.62811200	1.48069500	0.59175800
F	-0.40073300	-2.56994200	-0.87743700
F	-0.82214500	-5.06262500	-0.08427800
F	-0.26015900	-5.83827800	2.47995500
F	0.72923600	-4.00304400	4.25879200
F	1.13655300	-1.45140800	3.47410800
F	-2.22039400	-0.77355000	0.52365400
F	-4.26733500	0.57955000	1.58377100
F	-3.81105900	2.59145700	3.37397600
F	-1.24665900	3.23305100	4.07257500
F	0.81457000	1.93515400	2.98892600
F	3.20469800	-1.72172400	1.39251000
F	5.59509400	-0.60139700	1.15043900
F	5.83588700	2.02984200	0.45320300
F	3.59538600	3.52623300	0.00921300
F	1.17435300	2.43928200	0.27004400

TS_2

E(CPCM/B3LYP/6-31G(d)) = -3582.696387 au
H(CPCM/B3LYP/6-31G(d)) = -3582.170188 au
G(CPCM/B3LYP/6-31G(d)) = -3582.314871 au
E(SMD/M06-2X-D3/def2-TZVP//CPCM/B3LYP/6-31G(d)) = -3582.949627 au

C	3.55300400	-2.39098000	-2.23322900

C	2.38509100	-1.79329600	-1.77380900
C	1.78426200	-0.75226300	-2.50181000
C	2.37354700	-0.32403500	-3.70848200
C	3.55033000	-0.90189900	-4.16454900
C	4.11852400	-1.93196400	-3.41857300
H	4.02438900	-3.20013600	-1.68680900
H	1.94561600	-2.15381100	-0.85501500
H	1.91281700	0.47281100	-4.27960000
H	4.02695100	-0.57276900	-5.08117500
F	5.25012300	-2.50101600	-3.86008200
C	0.50979900	-0.14049900	-2.07511400
O	0.05974400	-0.02080600	-0.90992200
O	-0.18640800	0.29001500	-3.10366800
C	-1.91792800	1.07501200	-3.00521700
H	-2.06871400	1.25151200	-4.07502300
C	-2.88856900	0.08902000	-2.55673500
C	-3.70496900	-0.75713600	-2.24301100
Si	-4.90559100	-2.06453700	-1.69709100
C	-5.17224100	-1.90259300	0.16385000
H	-4.26876700	-2.18417600	0.71405800
H	-5.42312300	-0.87687600	0.45414700
H	-5.98784600	-2.56068400	0.48781100
C	-4.15467200	-3.74301700	-2.12068700
H	-4.84323500	-4.54863100	-1.83685200
H	-3.95567400	-3.83546800	-3.19438900
H	-3.21350800	-3.90042800	-1.58349900
C	-6.51990100	-1.78325000	-2.63653600
H	-6.37050500	-1.84965700	-3.72014200
H	-7.26157400	-2.54005300	-2.35296500
H	-6.94600500	-0.79834900	-2.41399700
C	-1.90328300	2.41505800	-2.29452200
C	-1.02533000	3.40781900	-2.75353400
C	-2.79441800	2.71221900	-1.25965700
C	-1.01835700	4.67383400	-2.17605500
H	-0.33748400	3.19094400	-3.56622100
C	-2.81004000	3.98184000	-0.68133100
H	-3.47888300	1.95043700	-0.90325800
C	-1.91772900	4.93862700	-1.14742300
H	-0.34053600	5.45043500	-2.51357000
H	-3.49710600	4.23028200	0.12036400
F	-1.92550800	6.16662600	-0.59092400
B	0.64851200	-0.14872800	0.59002900
C	0.38962400	-1.67752500	1.12201200
C	0.69618300	-1.97049600	2.45568500
C	-0.25812000	-2.70966800	0.44170400
C	0.41056100	-3.18192300	3.07332100
C	-0.57015300	-3.93895900	1.02208600
C	-0.23632900	-4.17925300	2.34809800

C	-0.34639800	0.81393900	1.47495500
C	0.05499500	1.76463800	2.41796500
C	-1.73021700	0.63474200	1.39823500
C	-0.83819000	2.51039400	3.18719500
C	-2.65689700	1.36309100	2.13552500
C	-2.20653700	2.31582500	3.04184100
C	2.19209800	0.38266800	0.51942300
C	3.33397400	-0.31872600	0.91023100
C	2.44701400	1.67100500	0.03632600
C	4.62270700	0.21118500	0.85259600
C	3.71242600	2.23872900	-0.04573300
C	4.81494400	1.50096200	0.37482600
F	-0.62070500	-2.57673100	-0.85713800
F	-1.19706100	-4.88730100	0.30537200
F	-0.52615200	-5.35353600	2.92006500
F	0.74119300	-3.39510800	4.35485300
F	1.30609200	-1.03429000	3.21276200
F	-2.24207600	-0.29047300	0.55906300
F	-3.97551800	1.16258300	1.96273300
F	-3.07623300	3.03321200	3.76314900
F	-0.38428200	3.41371900	4.06903700
F	1.35917300	2.01324100	2.65094400
F	3.25677600	-1.59921600	1.33283600
F	5.67702700	-0.52299500	1.23767800
F	6.04452800	2.02298100	0.30579200
F	3.87932000	3.48256000	-0.51939100
F	1.41767300	2.43789500	-0.38844000

TS_3

E(CPCM/B3LYP/6-31G(d)) = -4187.200417 au
H(CPCM/B3LYP/6-31G(d)) = -4186.537134 au
G(CPCM/B3LYP/6-31G(d)) = -4186.721294 au
E(SMD/M06-2X-D3/def2-TZVP//CPCM/B3LYP/6-31G(d)) = -4187.470976 au

C	-3.00284900	-1.13785900	-4.05561500
C	-2.25060100	-0.62912600	-2.99810800
C	-1.03776700	-1.22677100	-2.62815800
C	-0.58580800	-2.34439100	-3.35103100
C	-1.33360300	-2.87498500	-4.39695200
C	-2.53290200	-2.25550500	-4.73414600
H	-3.94048800	-0.68037700	-4.35259600
H	-2.62415300	0.23688900	-2.47082600
H	0.36306400	-2.79391300	-3.08182800
H	-1.00093800	-3.74590800	-4.95174100
F	-3.26086800	-2.75572400	-5.75353800
C	-0.13371800	-0.69326100	-1.55216400
O	-0.56374100	-0.05383400	-0.50420100
O	1.08754800	-0.87998200	-1.70358200
C	3.05356400	-0.66063600	-0.64360400
C	2.96722000	0.76065300	-0.67413400

C	2.97133800	1.98155100	-0.64312700
Si	2.93820900	3.84428300	-0.54858800
C	3.41397600	4.30519300	1.21750600
H	2.71014600	3.86929200	1.93434800
H	4.42159400	3.95647100	1.47148600
H	3.39486500	5.39457000	1.34453700
C	1.20770800	4.45492700	-0.96542000
H	1.21253800	5.54959300	-1.03756900
H	0.83568500	4.05125200	-1.91151300
H	0.50238800	4.16607400	-0.18096700
C	4.20914100	4.48695600	-1.78923000
H	3.95561400	4.18543300	-2.81191700
H	4.24054200	5.58301400	-1.76281600
H	5.21634100	4.11895400	-1.56308600
C	2.61144700	-1.41349100	0.54093800
C	2.36071100	-2.79567800	0.42298500
C	2.42683300	-0.78412500	1.78496900
C	1.94267600	-3.53470200	1.52051000
H	2.47744000	-3.28158400	-0.53974000
C	2.01736400	-1.51744700	2.89352100
H	2.57556900	0.28676700	1.87729100
C	1.78180400	-2.88016600	2.74128300
H	1.72139700	-4.59362900	1.44450400
H	1.86090000	-1.04941600	3.85892500
F	1.37931400	-3.59151300	3.80588800
B	-1.87845400	0.20665900	0.22030400
C	-2.55583100	1.62002000	-0.30325100
C	-3.67723800	2.13341900	0.35662800
C	-2.02241800	2.47423400	-1.27016900
C	-4.24202000	3.37492900	0.09017000
C	-2.54973300	3.73180900	-1.56686900
C	-3.66613200	4.18953500	-0.88088500
C	-1.41654300	0.52862800	1.78885700
C	-2.03698200	0.04687800	2.94460000
C	-0.35866900	1.40740600	2.03994900
C	-1.62271500	0.36396500	4.23886900
C	0.09550800	1.74327500	3.31167500
C	-0.54093800	1.21447200	4.42813400
C	-2.81409600	-1.14447400	0.11701000
C	-4.09968300	-1.24012000	-0.41485400
C	-2.29879800	-2.35744900	0.58467200
C	-4.82865300	-2.42798500	-0.47186300
C	-2.98373400	-3.56612200	0.54656000
C	-4.26890800	-3.60155700	0.01526700
F	-0.94462500	2.11863200	-2.00986100
F	-1.97608300	4.50383200	-2.50660600
F	-4.18662200	5.39457800	-1.15249600
F	-5.32391700	3.79990400	0.76262800

F	-4.27129700	1.39931100	1.32314200
F	0.29455600	2.00328900	1.01534000
F	1.15279000	2.56657900	3.47035400
F	-0.12059100	1.52348700	5.66340100
F	-2.26133000	-0.14724000	5.30408100
F	-3.10487200	-0.77430200	2.87505700
F	-4.71210500	-0.16461400	-0.95987100
F	-6.06035700	-2.44751200	-1.00717200
F	-4.95129800	-4.75390000	-0.03448800
F	-2.42262700	-4.69221400	1.01968100
F	-1.05649800	-2.39630500	1.12335700
C	5.45043700	-0.27600100	-1.99440300
O	6.17573600	0.83730200	-1.88985700
O	5.07770200	-0.80401700	-3.01200300
C	5.08665800	-0.83351000	-0.62679200
N	5.51506800	-0.05703700	0.40582200
N	5.70096900	0.63245200	1.26875500
C	5.34569600	-2.31179100	-0.41052700
O	5.12747400	-3.13812500	-1.26128700
O	5.78691100	-2.53658600	0.82824500
C	6.00341400	-3.92628900	1.18453400
H	6.71679300	-4.37964500	0.49447000
H	6.40205100	-3.89849900	2.19692300
H	5.05442200	-4.46422300	1.15423600
C	6.51979200	1.48147100	-3.14467600
H	7.11435900	0.80134100	-3.75685700
H	5.60950600	1.76606200	-3.67473900
H	7.09813000	2.35899800	-2.86218700
H	3.10247700	-1.18861800	-1.58621200

TS_4

E(CPCM/B3LYP/6-31G(d)) = -4187.194716 au
H(CPCM/B3LYP/6-31G(d)) = -4186.533166 au
G(CPCM/B3LYP/6-31G(d)) = -4186.718403 au
E(SMD/M06-2X-D3/def2-TZVP//CPCM/B3LYP/6-31G(d)) = -4187.486114 au

C	-3.37657300	-1.42750900	-3.78900400
C	-2.48645000	-0.92397000	-2.84187300
C	-1.39906200	-1.69070000	-2.40222100
C	-1.21049300	-2.97520400	-2.94047000
C	-2.09978100	-3.49840200	-3.87326900
C	-3.16945000	-2.70893500	-4.28455500
H	-4.21963000	-0.84220100	-4.14014500
H	-2.64964900	0.07152600	-2.45394000
H	-0.35551600	-3.55774800	-2.61657700
H	-1.97411000	-4.49394600	-4.28573100
F	-4.03187600	-3.20454300	-5.19625900
C	-0.35980900	-1.18303000	-1.44559600
O	-0.60578500	-0.26659500	-0.55147000
O	0.78570100	-1.65326200	-1.55328000

C	3.16536300	-0.61829000	-0.26701800
C	3.32135900	0.75872700	-0.71992100
C	3.41602100	1.91589200	-1.08790200
Si	3.33847600	3.70773300	-1.57595000
C	2.82864400	4.66692400	-0.03414800
H	1.96822400	4.19620800	0.45141400
H	3.64488600	4.70225900	0.69678200
H	2.56159700	5.69932600	-0.29011900
C	2.07676400	3.87644800	-2.96617000
H	2.39620200	3.32358900	-3.85734700
H	1.08986700	3.50224000	-2.67653600
H	1.96852900	4.93092300	-3.24949600
C	5.05264600	4.23898400	-2.16832600
H	5.37293800	3.66445900	-3.04514600
H	5.04263800	5.29869600	-2.45156900
H	5.80743400	4.11183800	-1.38380200
C	2.91670400	-0.78885600	1.23127400
C	2.02446500	-1.77813200	1.66974400
C	3.54713800	0.03588600	2.17240300
C	1.79063400	-1.96734300	3.03077300
H	1.48207500	-2.37684300	0.94641000
C	3.32608000	-0.14689900	3.53603900
H	4.18675900	0.84968800	1.84445700
C	2.45490300	-1.15308300	3.94069900
H	1.08356500	-2.71075600	3.38202300
H	3.79835700	0.48827000	4.27760700
F	2.23318300	-1.32849600	5.25799700
B	-1.81337600	0.24044200	0.23160100
C	-2.40431500	1.63530900	-0.42837900
C	-3.45991800	2.30428000	0.19978900
C	-1.82298200	2.34706000	-1.47893800
C	-3.93191900	3.55438500	-0.18230000
C	-2.25655100	3.60699000	-1.89277900
C	-3.31819700	4.21863900	-1.24076300
C	-1.18099700	0.74424300	1.68537400
C	-1.72692900	0.50992700	2.94943700
C	-0.04134600	1.55489400	1.69870100
C	-1.17395700	0.99517000	4.13490400
C	0.54692300	2.05500400	2.85580200
C	-0.02221400	1.76990000	4.09098900
C	-2.87224300	-1.01012400	0.39804100
C	-4.18529400	-1.06745200	-0.06893200
C	-2.44341900	-2.17964300	1.03392500
C	-5.01879100	-2.17410800	0.09426500
C	-3.23368600	-3.30779600	1.21790700
C	-4.54221000	-3.30355300	0.74595300
F	-0.78336500	1.83976600	-2.18403500
F	-1.64168000	4.23547300	-2.91114700

F	-3.74884700	5.42885400	-1.62496000
F	-4.95767800	4.13244900	0.46400300
F	-4.07428200	1.72839300	1.25672200
F	0.55032000	1.92094500	0.53814100
F	1.66013100	2.81070200	2.78869600
F	0.53161300	2.23746800	5.21994800
F	-1.74597000	0.71779400	5.31896700
F	-2.85407900	-0.21784100	3.10112000
F	-4.72614700	-0.04056100	-0.76173000
F	-6.27247000	-2.16071900	-0.38784500
F	-5.32614600	-4.37845300	0.90887300
F	-2.75099800	-4.39362900	1.84619600
F	-1.17943100	-2.25833900	1.51952500
C	4.51493200	-1.36735200	-2.33567300
O	5.69699400	-0.83709000	-2.61119800
O	3.66347300	-1.72841800	-3.10377400
C	4.25652800	-1.51566100	-0.82310400
N	5.73906200	-0.90103300	-0.10334300
N	6.49363900	-0.32625800	0.46166700
C	4.21152600	-2.98307600	-0.36178700
O	3.33632100	-3.69535500	-0.77986300
O	5.18102200	-3.28810400	0.48626300
C	5.16219100	-4.64872300	1.00650100
H	5.23338700	-5.35731600	0.18023400
H	6.03213800	-4.71133400	1.65664700
H	4.23927700	-4.80726800	1.56601800
C	5.98262300	-0.63391800	-4.02462300
H	5.95552200	-1.59280500	-4.54416600
H	5.24471200	0.04853900	-4.44864100
H	6.98010200	-0.20032200	-4.04908300
H	2.25456300	-1.05066500	-0.80218800

6

E(CPCM/B3LYP/6-31G(d)) = -2812.697172 au

H(CPCM/B3LYP/6-31G(d)) = -2812.379699 au

G(CPCM/B3LYP/6-31G(d)) = -2812.498315 au

E(SMD/M06-2X-D3/def2-TZVP//CPCM/B3LYP/6-31G(d)) = -2812.656965 au

O	-0.75849800	-0.60426900	-0.84885900
B	0.32990900	0.16895400	0.08905300
C	-0.42509200	0.30518900	1.53304400
C	-0.27942100	1.44281000	2.33328300
C	-1.23834700	-0.68390000	2.08853500
C	-0.91396000	1.61097300	3.56141900
C	-1.89825400	-0.55840400	3.30764900
C	-1.73749100	0.60380600	4.05218300
C	0.43531200	1.60110500	-0.69835300
C	1.62663500	2.24060800	-1.05235800
C	-0.72361100	2.30580300	-1.04636100
C	1.67657600	3.45883700	-1.72863200

C	-0.71784500	3.52109600	-1.72551400
C	0.49553000	4.10396100	-2.07305000
C	1.71845000	-0.68055100	0.11513600
C	2.44329200	-0.99350900	1.26553700
C	2.30539400	-1.11343500	-1.07764900
C	3.65107100	-1.69431800	1.24321100
C	3.49868400	-1.81857500	-1.14788200
C	4.18204900	-2.11152900	0.03017100
F	-1.41973600	-1.87261100	1.45376800
F	-2.67366500	-1.55118400	3.77064400
F	-2.35677100	0.74598500	5.23002800
F	-0.73110100	2.73050200	4.27636100
F	0.53047400	2.45034900	1.94847700
F	-1.93704200	1.82462500	-0.71232500
F	-1.86958400	4.13707300	-2.03650100
F	0.52498800	5.27342800	-2.72394400
F	2.85714300	4.01388600	-2.04396100
F	2.82697000	1.71111700	-0.73636700
F	2.01322100	-0.62041400	2.48818500
F	4.29961300	-1.96746700	2.38561900
F	5.33562700	-2.78945900	-0.00939700
F	3.99828700	-2.21689400	-2.32861400
F	1.68734200	-0.85144200	-2.25246000
C	-1.01419000	-1.79400100	-1.12376900
O	-0.12512000	-2.73265100	-0.89333200
C	-0.46918200	-4.12689800	-0.69794000
H	-1.47376800	-4.21799900	-0.28160700
H	-0.37789300	-4.66632400	-1.64228200
H	0.26638600	-4.49898800	0.01368200
C	-2.29773700	-2.08400700	-1.74978100
C	-3.53188400	-1.28439100	-1.50645400
O	-3.65134900	-0.49122800	-0.60378900
O	-4.47789700	-1.60691800	-2.40262100
C	-5.75043600	-0.94269700	-2.23222600
H	-6.38624800	-1.33754600	-3.02327200
H	-6.16627800	-1.17164500	-1.24871600
H	-5.62574400	0.13693600	-2.33741200
N	-2.36684900	-3.06373200	-2.63697200
N	-2.45718500	-3.88112100	-3.41212200

$TS_{1\text{-}6}$

E(CPCM/B3LYP/6-31G(d)) = -2812.692674 au

H(CPCM/B3LYP/6-31G(d)) = -2812.375464 au

G(CPCM/B3LYP/6-31G(d)) = -2812.496454 au

E(SMD/M06-2X-D3/def2-TZVP//CPCM/B3LYP/6-31G(d)) = -2812.652522 au

O	-0.62655200	0.24738200	1.20500700
B	0.60455100	-0.36056900	-0.49006100
C	-0.54050300	-0.12314100	-1.57149400
C	-0.95068400	-1.11120100	-2.47386900

C	-1.20347100	1.10249600	-1.70270800
C	-1.95030800	-0.91306200	-3.42330800
C	-2.21580800	1.33659500	-2.62459100
C	-2.59219900	0.31797100	-3.49529400
C	0.82979400	-1.83073900	0.07444000
C	2.09516500	-2.40952000	0.23292300
C	-0.24548600	-2.65380000	0.44278400
C	2.29368900	-3.69321500	0.73559000
C	-0.08535500	-3.93434800	0.96056000
C	1.19536900	-4.45899000	1.10826100
C	1.78523700	0.69930100	-0.39064600
C	2.19572800	1.48866000	-1.47085300
C	2.50964500	0.89250700	0.79279000
C	3.23795800	2.41032900	-1.39131900
C	3.54396700	1.81115200	0.91613500
C	3.91321200	2.57590300	-0.18790900
F	-0.87335400	2.14247900	-0.90605000
F	-2.82279200	2.53038300	-2.68881100
F	-3.55702500	0.52496000	-4.39589100
F	-2.29519000	-1.89624300	-4.26562200
F	-0.36467300	-2.32302400	-2.47940700
F	-1.50949600	-2.22644600	0.30076900
F	-1.15074900	-4.67065400	1.30737700
F	1.36608500	-5.69055600	1.59703800
F	3.53124600	-4.19304100	0.86032700
F	3.21039500	-1.74405900	-0.12321800
F	1.60273600	1.37506900	-2.67441600
F	3.59197500	3.13446900	-2.46247300
F	4.91061000	3.45982800	-0.08988300
F	4.19074600	1.96594900	2.08059300
F	2.20642400	0.17937600	1.89082500
C	-0.88530500	1.28395500	1.80143600
O	0.03461300	2.24943800	1.90083600
C	-0.30843100	3.64548400	2.03355000
H	-1.29381900	3.84924300	1.60868700
H	-0.26856000	3.94788500	3.08270400
H	0.45268000	4.18519100	1.46992400
C	-2.19194200	1.44762200	2.46414800
C	-3.42124800	0.74930500	2.01327200
O	-3.52063700	0.15369400	0.96498400
O	-4.40777900	0.90179900	2.91741700
C	-5.67288800	0.30494600	2.56361900
H	-6.34054000	0.53620900	3.39268400
H	-6.04848300	0.73620100	1.63290200
H	-5.56112900	-0.77529900	2.44746600
N	-2.26203400	2.14654800	3.58161400
N	-2.33072900	2.73334200	4.54759700

$TS_{6\text{-}7}$

E(CPCM/B3LYP/6-31G(d)) = -2812.675082 au
H(CPCM/B3LYP/6-31G(d)) = -2812.360811 au
G(CPCM/B3LYP/6-31G(d)) = -2812.483944 au
E(SMD/M06-2X-D3/def2-TZVP//CPCM/B3LYP/6-31G(d)) = -2812.945107 au

O	-1.02547000	0.71226900	0.41531600
B	0.25612700	-0.09160100	-0.12617600
C	1.22040700	-0.58900900	1.10048000
C	2.33792800	-1.36541000	0.77632400
C	1.15626000	-0.21136000	2.44049700
C	3.29993200	-1.76910500	1.69411700
C	2.09428200	-0.59385200	3.39924100
C	3.17744100	-1.37747800	3.02436200
C	1.09237600	1.05919000	-0.93266800
C	1.71930000	0.87802000	-2.16723600
C	1.32094400	2.30496700	-0.33879700
C	2.48155300	1.86297900	-2.79241300
C	2.06947000	3.31802000	-0.93121900
C	2.65524500	3.09468400	-2.17241500
C	-0.43301400	-1.25934600	-1.04000100
C	-0.48846100	-2.61519300	-0.70644100
C	-1.12191100	-0.92511800	-2.21183100
C	-1.12639000	-3.57616700	-1.48932400
C	-1.77314700	-1.84994600	-3.02133700
C	-1.77334600	-3.19231300	-2.65725700
F	0.13989500	0.56168900	2.90529300
F	1.95836200	-0.20363700	4.67648700
F	4.09031200	-1.75283700	3.92897500
F	4.34153600	-2.52223300	1.31193700
F	2.51637000	-1.76247700	-0.50190700
F	0.82103300	2.57210300	0.88671300
F	2.24263100	4.49603500	-0.31111300
F	3.38396000	4.05268400	-2.75983700
F	3.04990300	1.63069600	-3.98610700
F	1.61347100	-0.29041100	-2.83243400
F	0.06917000	-3.07668400	0.43202500
F	-1.13725900	-4.86448000	-1.11203600
F	-2.39791300	-4.10026800	-3.41668900
F	-2.40687100	-1.45920900	-4.13803700
F	-1.18505100	0.36114300	-2.61528700
C	-1.89087000	0.34025100	1.25200600
O	-1.90480500	-0.91252500	1.64240900
C	-2.36976700	-1.33768000	2.94986500
H	-2.40356200	-0.48876600	3.63297700
H	-3.34932500	-1.80374300	2.84426600
H	-1.63897300	-2.07592500	3.27868000
C	-2.74940000	1.34207500	1.81496800
C	-3.21980500	2.51318500	1.13335200
O	-2.28120100	3.27406400	0.92804900

O	-4.49819100	2.71350800	0.88101100
C	-4.82696800	4.00498800	0.29743800
H	-5.91244700	4.00919800	0.22157900
H	-4.47736600	4.80699700	0.94978600
H	-4.36794600	4.09225900	-0.68869500
N	-4.65786700	0.26663900	2.02313400
N	-5.69384500	0.09081400	2.36484900

7

E(CPCM/B3LYP/6-31G(d)) = -2703.159102 au

H(CPCM/B3LYP/6-31G(d)) = -2702.854352 au

G(CPCM/B3LYP/6-31G(d)) = -2702.973519 au

E(SMD/M06-2X-D3/def2-TZVP//CPCM/B3LYP/6-31G(d)) = -2703.421369 au

O	-0.78741400	0.24306000	1.26880700
B	0.14394500	0.01032700	-0.01975400
C	1.36101300	-1.02554500	0.33231700
C	2.25573200	-1.35345300	-0.69110300
C	1.71291300	-1.52554500	1.58548000
C	3.38912900	-2.13738400	-0.51501200
C	2.83907600	-2.31746100	1.81076600
C	3.68538000	-2.62621500	0.75423700
C	0.82837800	1.48089800	-0.23973400
C	0.99803300	2.11079200	-1.47458800
C	1.39750900	2.15975900	0.84303300
C	1.64488100	3.33560500	-1.62964200
C	2.04616800	3.38635900	0.73344400
C	2.16982400	3.98182500	-0.51701200
C	-0.91979100	-0.45603700	-1.17213600
C	-0.99059100	-1.72252000	-1.75686900
C	-1.92166700	0.42775300	-1.58946400
C	-1.94624400	-2.07977700	-2.70797800
C	-2.89538300	0.11341300	-2.53131400
C	-2.90646500	-1.15597000	-3.09985200
F	0.96031000	-1.26886000	2.68372800
F	3.11111600	-2.77642400	3.04298000
F	4.77168700	-3.38394400	0.95264300
F	4.19784300	-2.42059200	-1.54719700
F	2.02388500	-0.89431900	-1.94026700
F	1.35308100	1.61989800	2.08044500
F	2.56203900	3.98915300	1.81650800
F	2.79306700	5.16004400	-0.64799300
F	1.76569800	3.89400900	-2.84436900
F	0.53260500	1.54929700	-2.60917800
F	-0.13053800	-2.70168100	-1.41074200
F	-1.95643300	-3.31607500	-3.23134100
F	-3.83658200	-1.48522300	-4.00475500
F	-3.82384700	1.01536300	-2.88792700
F	-1.98193900	1.67205000	-1.06771500
C	-1.49477300	-0.59881400	1.87320800

O	-1.53969300	-1.85070100	1.47752400
C	-2.32466000	-2.80798400	2.23937400
H	-2.00189200	-2.79913600	3.28365600
H	-3.38866000	-2.57587000	2.15193300
H	-2.11337100	-3.77181400	1.78085000
C	-2.17522900	-0.24105900	3.07970700
C	-3.29053300	0.57876800	3.19545500
O	-2.61332000	1.63075900	3.16770800
O	-4.55685300	0.37444300	3.35390600
C	-5.38731900	1.56621400	3.55546000
H	-6.38399600	1.17541000	3.74465000
H	-5.01448200	2.12776600	4.41273000
H	-5.36928100	2.17551700	2.65134500

TS1′

E(CPCM/B3LYP/6-31G(d)) = -3582.685130 au

H(CPCM/B3LYP/6-31G(d)) = -3582.155656 au

G(CPCM/B3LYP/6-31G(d)) = -3582.303775 au

E(SMD/M06-2X-D3/def2-TZVP//CPCM/B3LYP/6-31G(d)) = -3582.947571 au

C	-0.89287700	1.23455900	-1.58825300
O	-0.18895800	1.66534600	-2.45812800
O	-0.29463000	0.85411000	-0.31918400
C	0.56856700	2.00683700	0.24734600
H	0.65766100	1.67653800	1.27893000
C	1.88872800	2.05557100	-0.35201800
C	3.03213600	2.14056800	-0.76249100
Si	4.76013400	2.21382500	-1.43256300
C	5.29556600	4.02482600	-1.45964400
H	4.67335600	4.61322000	-2.14343100
H	5.22849700	4.47942600	-0.46478500
H	6.33602800	4.10986000	-1.79666400
C	4.73702800	1.49114300	-3.17523800
H	5.76095900	1.34640200	-3.54146400
H	4.22708500	0.52331200	-3.20393600
H	4.22331900	2.16295900	-3.87235200
C	5.87104600	1.20420800	-0.28812700
H	5.54362500	0.15976900	-0.23533600
H	6.90568100	1.21388800	-0.65231700
H	5.86962600	1.60909500	0.73021500
C	-0.21105500	3.30333900	0.22071300
C	-1.19373400	3.52427100	1.19847000
C	0.04376900	4.30124000	-0.72987400
C	-1.91626100	4.71406900	1.22791500
H	-1.40570900	2.75923800	1.93942100
C	-0.66848700	5.49807100	-0.71135800
H	0.80812700	4.14055400	-1.48206300
C	-1.63774400	5.68230500	0.26858100
H	-2.67662100	4.90008500	1.97862900

H	-0.48104400	6.28135200	-1.43787100
F	-2.32760200	6.83916500	0.29363900
B	-0.00747900	-0.87898700	0.39874400
C	-1.34017500	-1.74086100	0.05368300
C	-1.68817500	-1.99689500	-1.27953200
C	-2.05200600	-2.51187800	0.98292200
C	-2.68229300	-2.88108400	-1.67936700
C	-3.04962500	-3.41843800	0.62625100
C	-3.36794600	-3.60907800	-0.71215000
C	0.26300900	-0.45773400	1.94062900
C	1.52445800	-0.50507100	2.55792600
C	-0.75585500	0.00817800	2.78392900
C	1.74454900	-0.17715000	3.89555300
C	-0.57907900	0.35813500	4.11634500
C	0.68687800	0.25626500	4.68412900
C	1.22286900	-1.65407300	-0.33963500
C	1.58820600	-2.87564200	0.24432100
C	1.88287000	-1.37388500	-1.53459900
C	2.54694300	-3.73712900	-0.27147700
C	2.85517200	-2.21002900	-2.08632400
C	3.19606500	-3.39719200	-1.45457900
F	-1.02299900	-1.36552900	-2.27329200
F	-2.96257400	-3.05569300	-2.97740700
F	-4.31058900	-4.48725500	-1.06519200
F	-3.68738900	-4.12450400	1.56946400
F	-1.78347100	-2.45423100	2.29998900
F	-2.01102200	0.17378900	2.30862400
F	-1.60944500	0.80328700	4.84680800
F	0.88346100	0.58722900	5.96126300
F	2.97516200	-0.25661400	4.41567200
F	2.62792700	-0.85445000	1.87468100
F	1.61182100	-0.26376000	-2.23826300
F	3.46323700	-1.86610200	-3.23460300
F	4.12569600	-4.20535200	-1.97592200
F	2.84751000	-4.88337600	0.35582300
F	0.98813900	-3.26496800	1.39304700
C	-2.36394700	1.16949400	-1.65417700
C	-3.19237200	1.05871000	-0.52804900
C	-2.94108000	1.33631700	-2.92849400
C	-4.57620200	1.12971700	-0.66474700
H	-2.76107100	0.93458300	0.45383700
C	-4.31911700	1.39655200	-3.07679200
H	-2.29347200	1.42491800	-3.79344300
C	-5.11435300	1.30316600	-1.93545600
H	-5.23442300	1.06147800	0.19443800
H	-4.78337900	1.52444200	-4.04847300
F	-6.44862800	1.39127700	-2.06996200

TS2′

E(CPCM/B3LYP/6-31G(d)) = -3582.681792 au
H(CPCM/B3LYP/6-31G(d)) = -3582.153075 au
G(CPCM/B3LYP/6-31G(d)) = -3582.302007 au
E(SMD/M06-2X-D3/def2-TZVP//CPCM/B3LYP/6-31G(d)) = -3582.940794 au

C	0.38355800	1.00411500	-0.87898900
O	1.20598200	0.08560100	-0.95235000
O	-0.83010100	0.92836900	-0.40107700
C	3.26938300	0.22161800	0.84076800
H	2.31995400	-0.12110300	1.24183500
C	4.09132700	-0.76452400	0.32187400
C	4.75169800	-1.71356200	-0.09619500
Si	5.66266300	-3.23518600	-0.70256100
C	7.37052700	-3.18064900	0.09435900
H	7.30523700	-3.21406700	1.18736500
H	7.95398900	-4.05032900	-0.23197900
H	7.92145700	-2.27790500	-0.19041500
C	5.75797000	-3.05373800	-2.57685000
H	6.27881500	-3.92195500	-2.99908200
H	4.75966400	-3.01077200	-3.02519200
H	6.30719300	-2.15299200	-2.87043700
C	4.66662000	-4.75086300	-0.17113400
H	5.27837400	-5.41985200	0.44443400
H	3.77970700	-4.47845200	0.40890100
H	4.32788400	-5.31644700	-1.04624300
C	3.57761300	1.59043400	0.95389700
C	2.61033500	2.45863100	1.54225900
C	4.81862400	2.12663100	0.50133400
C	2.87648700	3.80603800	1.68286000
H	1.65623800	2.05576000	1.86874200
C	5.08711800	3.47008300	0.63910500
H	5.54955900	1.46265400	0.05154500
C	4.10856100	4.28667600	1.22800300
H	2.16095700	4.49190000	2.12120300
H	6.02055100	3.91141300	0.30881100
F	4.37107600	5.58278300	1.35947000
B	-1.75720200	-0.20802100	0.02011900
C	-2.94596600	-0.08947000	-1.12352800
C	-2.59163300	-0.26417200	-2.46570300
C	-4.27353000	0.29391600	-0.93311800
C	-3.46273100	-0.10646900	-3.53603900
C	-5.18693100	0.46348200	-1.97627900
C	-4.78142700	0.26212700	-3.28768300
C	-2.25999200	0.11123400	1.55974600
C	-3.17404100	-0.75240500	2.16993800
C	-1.78665200	1.12356100	2.39435500
C	-3.61213800	-0.62987100	3.48318400
C	-2.19457800	1.28595000	3.71895100
C	-3.11538500	0.40517600	4.26966900

C	-1.00808200	-1.68399700	0.08169400
C	-1.36900800	-2.84332300	-0.60780000
C	0.08513900	-1.85584700	0.93064300
C	-0.66442500	-4.04650300	-0.52261700
C	0.83080500	-3.02083600	1.03586900
C	0.45200500	-4.13542200	0.29735200
F	-1.32401600	-0.61934600	-2.77965800
F	-3.04857800	-0.30183400	-4.79983600
F	-5.64393700	0.42425000	-4.30149700
F	-6.45307100	0.83337800	-1.71795800
F	-4.76837300	0.54800200	0.29859100
F	-0.86494500	2.02960000	1.97223600
F	-1.69421500	2.28538700	4.46817000
F	-3.51789400	0.54725000	5.54105500
F	-4.50172800	-1.49396800	3.99887400
F	-3.69164700	-1.77754600	1.45709700
F	0.50358800	-0.81995000	1.71223200
F	1.92743100	-3.07317000	1.82052000
F	1.16218800	-5.27199500	0.37441700
F	-1.05854600	-5.11809600	-1.22952600
F	-2.44139000	-2.87028300	-1.42441400
C	0.73726600	2.38854000	-1.34092800
C	-0.05838000	3.50010800	-1.02211500
C	1.89969500	2.57071500	-2.10503100
C	0.30296800	4.77501900	-1.45052800
H	-0.95092800	3.35686600	-0.42481300
C	2.26824600	3.83746200	-2.54965400
H	2.50242900	1.70330700	-2.35150000
C	1.46103500	4.91915200	-2.20929300
H	-0.29470900	5.64830400	-1.21086600
H	3.15587400	3.99758000	-3.15282200
F	1.81482800	6.15141900	-2.62989000

5.3 Computational details

Gaussian 16[13] was used to fully optimize all the structures reported in this paper at the B3LYP level[14] of density functional theory (DFT) using the CPCM solvation model [15] in toluene. The 6-31G(d) basis set[16] was chosen for all atoms. Frequency calculations were carried out at the same level of theory as those for the structural optimization. Transition structures were located using the Berny algorithm and intrinsic reaction coordinate (IRC) calculations[17] were used to confirm the connectivity between transition structures and minima. To further refine the energies obtained from the CPCM/B3LYP/6-31G(d) calculations and to consider dispersive interactions,[18] we carried out single-point energy calculations using the M06-2X-D3 functional method[19] for

all of the structures with a larger basis set def2-TZVP[20] and the SMD solvation model[21] in toluene. All thermodynamic data were calculated at the standard state (298.15 K and 1 atm). To estimate the corresponding Gibbs free energies, entropy corrections were calculated at the B3LYP level and added to the single-point potential energies. To adjust relative free energies of all stationary points from 1 atm to 1 M standard state, the conversion factor of 1.89 kcal/mol were employed. To further confirm the accuracy of the results obtained from SMD/M06-2X-D3/def2-TZVP//CPCM/B3LYP/6-31G(d), other single-point calculations were carried out using the SMD/M06-2X/def2-TZVP and SMD/wB97XD[22]/def2-TZVP levels of theory. The energy profiles obtained by these additional single point calculations are depicted in Figures S143 and S144. The overall activation free energy of the reaction is calculated to be 29.2, 31.1 and 31.8 kcal/mol using single point calculations obtained by M06-2X-D3, M06-2X, and wB97XD, respectively. These results indicate that the functional dependence is essentially insignificant.

6. References:

[1] M. Santi, D. M. C. Ould, J. Wenz, Y. Soltani, R. L. Melen, T. Wirth, *Angew. Chem. Int. Ed*. **2019**, *58*, 7861–7865.

[2] J. R. Lawson, L. C. Wilkins, R. L. Melen, *Chem. Eur. J.* **2017**, *23*, 10997–11000.

[3] R. K. Harris, E. D. Becker, S. M. Cabral De Menezes, R. Goodfellow, P. Granger, *Magn. Reson. Chem.* **2002**, *40*, 489–505.

[4] X. Wang, K. Nozaki, *J. Am. Chem. Soc.* **2018**, *140*, 15635–15640.

[5] S. Racine, F. de Nanteuil, E. Serrano, J. Waser, *Angew. Chem. Int. Ed*. **2014**, *53*, 8484–8487.

[6] A.-C. Chany, L. B. Marx, J. W. Burton, *Org. Biomol. Chem.* **2015**, *13*, 9190–9193.

[7] J. Hao, Y. Xu, Z. Xu, Z. Zhang, W. Yang, *Org. Lett*. **2018**, *20*, 7888−7892.

[8] M. T. La, H.-K. Kim, *Tetrahedron. Lett.* **2018**, *59*, 1855–1859.

[9] W. Zhao, P. K. Yan, A. T. Radosevich, *J. Am. Chem. Soc*. **2015**, *137*, 616–619.

[10] J.-L. Shi, Q. Luo, W. Yu, B. Wang, Z.-J. Shi, J. Wang, *Chem. Commun.* **2019**, *55*, 4047–4050.

[11] CrysAlisPro, Agilent Technoligies, Version 1.171.37.33 (release 27-03-2014 CrysAlis171 .NET).

[12] SHELXL-2013, G.M. SHeldrick, University of Göttingen, Germany (2013).

[13] Gaussian 16, Revision B.01, M. J. Frisch, G. W. Trucks, H. B. Schlegel, G. E. Scuseria, M. A. Robb, J. R. Cheeseman, G. Scalmani, V. Barone, G. A. Petersson, H. Nakatsuji, X. Li, M. Caricato, A. V. Marenich, J. Bloino, B. G. Janesko, R. Gomperts, B. Mennucci, H. P. Hratchian, J. V. Ortiz, A. F. Izmaylov, J. L. Sonnenberg, D. Williams-Young, F. Ding, F. Lipparini, F. Egidi, J. Goings, B. Peng, A. Petrone, T. Henderson, D. Ranasinghe, V. G. Zakrzewski, J. Gao, N. Rega, G. Zheng, W. Liang, M. Hada, M. Ehara, K. Toyota, R. Fukuda, J. Hasegawa, M. Ishida, T. Nakajima, Y. Honda, O. Kitao, H. Nakai, T. Vreven, K. Throssell, J. A. Montgomery, Jr., J. E. Peralta, F. Ogliaro, M. J. Bearpark, J. J. Heyd, E. N. Brothers, K. N. Kudin, V. N. Staroverov, T. A. Keith, R. Kobayashi, J. Normand, K. Raghavachari, A. P. Rendell, J. C. Burant, S. S. Iyengar, J. Tomasi, M. Cossi, J. M. Millam, M. Klene, C. Adamo, R. Cammi, J. W. Ochterski, R. L. Martin, K. Morokuma, O. Farkas, J. B. Foresman, and D. J. Fox, Gaussian, Inc., Wallingford CT, **2016**.

[14] (a) C. T. Lee, W. T. Yang, R. G. Parr, *Phys. Rev. B.* **1988**, *37*, 785−789; (b) B. Miehlich, A. Savin, H. Stoll, H. Preuss, *Chem. Phys. Lett.* **1989**, 157, 200−206; (c) A. D. J. Becke, *Chem. Phys.* **1993**, *98*, 5648−5652.

[15] V. Barone, M. J. Cossi, *Phys. Chem. A.* **1998**, *102*, 1995−2001.

[16] P. C. Hariharan, J. A. Pople, *Theor. Chim. Acta J. A.* **1973**, *28*, 213−222.

[17] (a) K. J. Fukui, *Phys. Chem.* **1970**, *74*, 4161–4163; (b) K. Fukui, *Acc. Chem. Res.* **1981**, 14, 363−368.

[18] S. Grimme, J. Antony, S. Ehrlich, H. J. Krieg, *Chem. Phys.* **2010**, *132*, 154104–154124.

[19] Y. Zhao, D. G. Truhlar, *Acc. Chem. Res.* **2008**, *41*, 157−167.

[20] F. Weigend, F. Furche and R. J. Ahlrichs, *Chem. Phys.* **2003**, *119*, 12753–12762.

[21] A. V. Marenich, C. J. Cramer, D. G. J. Truhlar, *Phys. Chem. B.* **2009**, *113*, 6378−6396.

[22] J.-D. Chai, M. Head-Gordon, *Phys. Chem. Chem. Phys.* **2008**, *10*, 6615–6620.
